# Safety, Immunogenicity, and Efficacy of the NVX-CoV2373 COVID-19 Vaccine in Adolescents

**DOI:** 10.1001/jamanetworkopen.2023.9135

**Published:** 2023-04-03

**Authors:** Germán Áñez, Lisa M. Dunkle, Cynthia L. Gay, Karen L. Kotloff, Jeffrey M. Adelglass, Brandon Essink, James D. Campbell, Shane Cloney-Clark, Mingzhu Zhu, Joyce S. Plested, Pavitra Roychoudhury, Alexander L. Greninger, Nita Patel, Alice McGarry, Wayne Woo, Cho Iksung, Gregory M. Glenn, Filip Dubovsky, 2019nCoV-301-Pediatric Expansion Study Group

**Affiliations:** Novavax, Inc, Gaithersburg, Maryland; Now with Vaccines Clinical Research, Global Clinical Development, Merck Research Laboratories, North Wales, Pennsylvania; Novavax, Inc, Gaithersburg, Maryland; Division of Infectious Diseases, University of North Carolina School of Medicine, Chapel Hill; Department of Pediatrics, Center for Vaccine Development and Global Health, University of Maryland School of Medicine, Baltimore; Research Your Health, Plano, Texas; Meridian Clinical Research, Omaha, Nebraska; Department of Pediatrics, Center for Vaccine Development and Global Health, University of Maryland School of Medicine, Baltimore; Novavax, Inc, Gaithersburg, Maryland; Novavax, Inc, Gaithersburg, Maryland; Novavax, Inc, Gaithersburg, Maryland; Department of Laboratory Medicine and Pathology, University of Washington, Seattle; Department of Laboratory Medicine and Pathology, University of Washington, Seattle; Novavax, Inc, Gaithersburg, Maryland; Novavax, Inc, Gaithersburg, Maryland; Novavax, Inc, Gaithersburg, Maryland; Novavax, Inc, Gaithersburg, Maryland; Novavax, Inc, Gaithersburg, Maryland; Novavax, Inc, Gaithersburg, Maryland

## Abstract

**IMPORTANCE:**

Greater than 20% of cases and 0.4% of deaths from COVID-19 occur in children. Following demonstration of the safety and efficacy of the adjuvanted, recombinant spike protein vaccine NVX-CoV2373 in adults, the PREVENT-19 trial immediately expanded to adolescents.

**OBJECTIVE:**

To evaluate the safety, immunogenicity, and efficacy of NVX-CoV2373 in adolescents.

**DESIGN, SETTING, AND PARTICIPANTS:**

The NVX-CoV2373 vaccine was evaluated in adolescents aged 12 to 17 years in an expansion of PREVENT-19, a phase 3, randomized, observer-blinded, placebo-controlled multicenter clinical trial in the US. Participants were enrolled from April 26 to June 5, 2021, and the study is ongoing. A blinded crossover was implemented after 2 months of safety follow-up to offer active vaccine to all participants. Key exclusion criteria included known previous laboratory-confirmed SARS-CoV-2 infection or known immunosuppression. Of 2304 participants assessed for eligibility, 57 were excluded and 2247 were randomized.

**INTERVENTIONS:**

Participants were randomized 2:1 to 2 intramuscular injections of NVX-CoV2373 or placebo, 21 days apart.

**MAIN OUTCOMES AND MEASURES:**

Serologic noninferiority of neutralizing antibody responses compared with those in young adults (aged 18–25 years) in PREVENT-19, protective efficacy against laboratory-confirmed COVID-19, and assessment of reactogenicity and safety.

**RESULTS:**

Among 2232 participants (1487 NVX-CoV2373 and 745 placebo recipients), the mean (SD) age was 13.8 (1.4) years, 1172 (52.5%) were male, 1660 (74.4%) were White individuals, and 359 (16.1%) had had a previous SARS-CoV-2 infection at baseline. After vaccination, the ratio of neutralizing antibody geometric mean titers in adolescents compared with those in young adults was 1.5 (95% CI, 1.3–1.7). Twenty mild COVID-19 cases occurred after a median of 64 (IQR, 57–69) days of follow-up, including 6 among NVX-CoV2373 recipients (incidence, 2.90 [95% CI, 1.31–6.46] cases per 100 person-years) and 14 among placebo recipients (incidence, 14.20 [95% CI, 8.42–23.93] cases per 100 person-years), yielding a vaccine efficacy of 79.5% (95% CI, 46.8%−92.1%). Vaccine efficacy for the Delta variant (the only viral variant identified by sequencing [n = 11]) was 82.0% (95% CI, 32.4%−95.2%). Reactogenicity was largely mild to moderate and transient, with a trend toward greater frequency after the second dose of NVX-CoV2373. Serious adverse events were rare and balanced between treatments. No adverse events led to study discontinuation.

**CONCLUSIONS AND RELEVANCE:**

The findings of this randomized clinical trial indicate that NVX-CoV2373 is safe, immunogenic, and efficacious in preventing COVID-19, including the predominant Delta variant, in adolescents.

**TRIAL REGISTRATION:**

ClinicalTrials.gov Identifier: NCT04611802

## Introduction

Optimal control of COVID-19 as it moves into an endemic state requires that vaccination be extended to all ages to minimize disease overall and, especially, to reduce the social and mental health effects on children and adolescents.^1^ Spike (S) protein–based vaccines for SARS-CoV-2 using messenger RNA (mRNA) technology are authorized or approved for use in adolescents and younger children in the US and elsewhere.^2^ NVX-CoV2373 (Novavax, Inc), a recombinant S protein vaccine coformulated with a saponin-based adjuvant (Matrix-M), is also authorized for emergency use in adults 18 years or older and recently for adolescents aged 12 to 17 years in the US and numerous other countries and regions.^3–11^ Approval of the vaccine has been based on safety, immunogenicity, and protective efficacy against symptomatic COVID-19,^12–14^ which has added another choice based on a different technology platform to the available mRNA vaccines.

We describe herein the data supporting safety, immunogenicity, and efficacy in adolescents aged 12 through 17 years in the PREVENT-19 trial. This analysis covered the precrossover, placebo-controlled period from April 26 to September 27, 2021, during which a predominance of the SARS-CoV-2 Delta variant was reported in the US (eFigure 1 in Supplement 1).

## Methods

### Trial Design, Participants, Procedures, and Oversight

PREVENT-19 is a phase 3, randomized, observer-blinded, placebo-controlled clinical trial initially conducted in adults in the US and Mexico evaluating the safety, immunogenicity, and efficacy of NVX-CoV2373.^13^ After the primary objective for adults was achieved,^13^ the pediatric expansion enrolled adolescents at 73 clinical sites in the US from April 26 to June 5, 2021. This study was reviewed and approved by the WIRB-Copernicus Group Institutional Review Board. The protocol, amendments, and overall oversight were approved by the Institutional Review Board. Parents or guardians of participants provided written informed consent while participants provided assent before enrollment and randomization. This study followed the Consolidated Standards of Reporting Trials (CONSORT) reporting guideline for randomized clinical trials.

Healthy adolescents aged 12 through 17 years or those with stable chronic medical conditions (as determined by the investigator based on review of overall health status, vital signs, medical history, and physical examination results), including chronic pulmonary, kidney, or cardiovascular disease; type 1 or 2 diabetes; or well-controlled HIV infection (defined as undetectable HIV RNA [<50 copies/mL] and CD4 count >200/μL for at least 1 year [to convert CD4 count to ×10^9^/L, multiply by 0.001]) that did not necessitate substantive changes in medications in the 2 months prior to enrollment and who were not currently undergoing workup of undiagnosed illness that could lead to diagnosis of a new condition were eligible for participation. Key exclusion criteria included known previous laboratory-confirmed SARS-CoV-2 infection or known immunosuppression. Additional details regarding trial design, conduct, oversight, and analyses are provided in the eMethods and eTable 1 in Supplement 1 and the trial protocol and statistical analysis plan (Supplement 2). Race and ethnicity were self-reported by the parents and participants as important parameters because the aim was to characterize the vaccine in a population that reflected the demographic composition of the US, and because certain minority populations had reported increased risk for COVID-19, hospitalization, and death.

Participants were allocated without age stratification in a 2:1 ratio to receive two 0.5-mL intramuscular injections of either NVX-CoV2373 (5 μg recombinant SARS-CoV-2 S plus 50 μg Matrix-M adjuvant) or normal saline placebo 21 days apart. Randomization used a web-based interactive system. Site personnel who managed study vaccine logistics and preparation had no subsequent role in participant assessment.

Trial data were available to all authors, who vouched for its accuracy and completeness and for fidelity to the trial protocol. The trial is ongoing, and investigators, Novavax, the clinical team, and the participants remain blinded to participant-level initial treatment assignments. Safety and efficacy were monitored through the placebo-controlled portion of the trial with regular reviews of unblinded data by the National Institute of Allergy and Infectious Diseases of the National Institutes of Health– sponsored data and safety monitoring board (eAppendix in Supplement 1).^15^

### Safety Assessments

Solicited local and systemic adverse events were collected via electronic diary for 7 days following each injection. Participants were assessed for all unsolicited adverse events from the first dose through 28 days after the second dose (day 49); serious adverse events, adverse events of special interest, and medically attended adverse events related to vaccination are to be collected from the first dose until the end of the study, which will occur approximately 2 years after enrollment. This report only includes data until the precrossover, placebo-controlled part of the study (April to September 2021).

### Immunogenicity Assessments

Day 0 (baseline) and day 35 serum samples were tested for neutralizing antibodies specific to SARS-CoV-2, measured with a validated microneutralization assay that defined titers as the inverse serum dilution that yielded 50% viral inhibition. The assay used wild-type virus strain SARS-CoV-2 hCoV-19/ Australia/VIC01/2020 (GenBank MT007544.1) (360biolabs)^16^ and has a lower limit of quantitation of 20. Additional immunogenicity end points included serum anti–SARS-CoV-2 S protein IgG antibody levels^17^ and human angiotensin-converting enzyme 2 (hACE2) receptor–binding inhibition antibodies to SARS-CoV-2 S protein.^18^ Both were validated enzyme-linked immunosorbent assays conducted at Novavax Clinical Immunology Laboratory using reagents based on the prototype Wuhan strain. Additionally, in post hoc analyses, anti-S IgG and hACE2 receptor–binding inhibition antibodies were measured against viral variants using reagents based on the SARS-CoV-2 Alpha, Beta, Delta, Gamma, Mu, and Omicron variants (fit-for-purpose assays conducted at Novavax Vaccine Immunology Laboratory). Details of these assays and results are provided in the eMethods in Supplement 1.

Prior exposure to SARS-CoV-2 was determined by the presence of serum antinucleoprotein antibodies (University of Washington, Seattle), using an anti–SARS-CoV-2 assay (Elecsys; F Hoffmann–La Roche Ltd),^19^ and/or positive results of SARS-CoV-2 reverse transcriptase–polymerase chain reaction (RT-PCR) on nasal swabs collected at baseline (University of Washington, Seattle) using a quantitative SARS-CoV-2 assay (RealTi*m*e; Abbott Laboratories).^13^

### Efficacy Assessments

The efficacy of NVX-CoV2373 in preventing the first episode of RT-PCR–confirmed symptomatic mild, moderate, or severe COVID-19 (according to US Food and Drug Administration [FDA] criteria)^20^ (eTable 2 in Supplement 1) with onset at least 7 days after the second injection in the per-protocol population of efficacy was summarized descriptively. Symptoms of suspected COVID-19 (eTable 3 in Supplement 1) were reported by participants’ parents or guardians as soon as possible after onset or during weekly calls. When prespecified symptoms were reported, participants were instructed to undergo in-clinic medical evaluation, which included collection of nasal swabs for RT-PCR. End point COVID-19 cases were confirmed by positive results of nasal swab RT-PCR at the central laboratory. Whole-genome sequencing and clade and lineage assignment were performed on RT-PCR–positive samples with sufficient viral RNA load (eMethods in Supplement 1). Severity of COVID-19 protocol-defined end points was assessed by investigators and study physicians according to protocol-specified criteria, and severe cases were confirmed through review by the external independent end point review committee blinded to treatment assignment. As implemented earlier for adult participants in PREVENT-19,^13^ a blinded crossover (participants originally randomized to placebo were offered NVX-CoV2373 and vice versa) was implemented for adolescent participants after a median follow-up of 71 (IQR, 65–77) days had been attained. The intention was to offer all participants active vaccine as soon as possible without compromising FDA-required placebo-controlled safety follow-up.

## Statistical Analysis

### Safety Analysis

Safety data from all participants who received at least 1 dose of study treatment were summarized descriptively. Severity and duration of solicited local and systemic adverse events, reported daily for 7 days by participants’ guardians in electronic diaries, were assessed (according to FDA criteria for severity^21^) after each injection. Unsolicited adverse events were coded by preferred term and system organ class using the Medical Dictionary for Regulatory Activities, version 24.0, and summarized by severity and investigator-assessed relationship to study vaccine.

### Immunogenicity Analysis

The per-protocol immunogenicity set included participants without prior exposure to SARS-CoV-2 who had a baseline serum sample and at least 1 serum sample result available after the full primary vaccination series with no protocol violations or events considered likely to impact immune response at the study visit in question (eg, RT-PCR–positive swabs, SARS-CoV-2 seropositivity, or receipt of other COVID-19 vaccine outside the study). The primary assessment of effectiveness was based on a formal analysis of noninferiority of the neutralizing antibody response in a randomly selected subset of adolescents at day 35 compared with that in a similarly selected per-protocol immunogenicity subset of young adult participants aged 18 to 25 years in this study.^13^

For all assays, the geometric mean titers (GMTs) at each study visit (ie, at day 0 [baseline] and at day 35) and the geometric mean fold rise (GMFR) with 95% CI compared with baseline (day 0) were calculated by treatment group at each postvaccination study visit. The 95% CI was calculated based on the *t* distribution of the log-transformed values for GMTs or GMFR, then back-transformed to the original scale for presentation. Serologic response was defined as the proportion of participants with at least a 4-fold increase between days 0 and 35. The 95% CI was calculated using the exact Clopper-Pearson method.^22^

The study was powered for the demonstration of serologic effectiveness; further details are presented in the eMethods in Supplement 1. The primary noninferiority effectiveness objective required meeting 3 criteria: (1) lower bound of 2-sided 95% CI for the ratio of GMTs (ie, for those aged 12–17 years to those aged 18–25 years) greater than 0.67, (2) point estimate of the ratio of GMTs at least 0.82 (estimated as the square root of 2/3), and (3) lower bound of the 2-sided 95% CI for difference of the serologic response (the serologic response for those aged 12–17 years divided by the serologic response for those aged 18–25 years) at least −10%.

### Efficacy Analysis

Participants who (1) had no evidence of prior SARS-CoV-2 infection at baseline or to at least 7 days after the second injection, (2) received both injections of assigned treatment, and (3) had no major protocol deviations were included in all protective efficacy analyses. Vaccine efficacy was defined as (1 − RR) × 100, where RR is the relative risk of end point incidence rates between the 2 treatment groups. The estimated RR and 2-sided 95% CI were derived using Poisson regression with robust error variance. All data analyses were performed using SAS, version 9.4 (SAS Institute Inc).

## Results

### Participants

**Figure 1.**
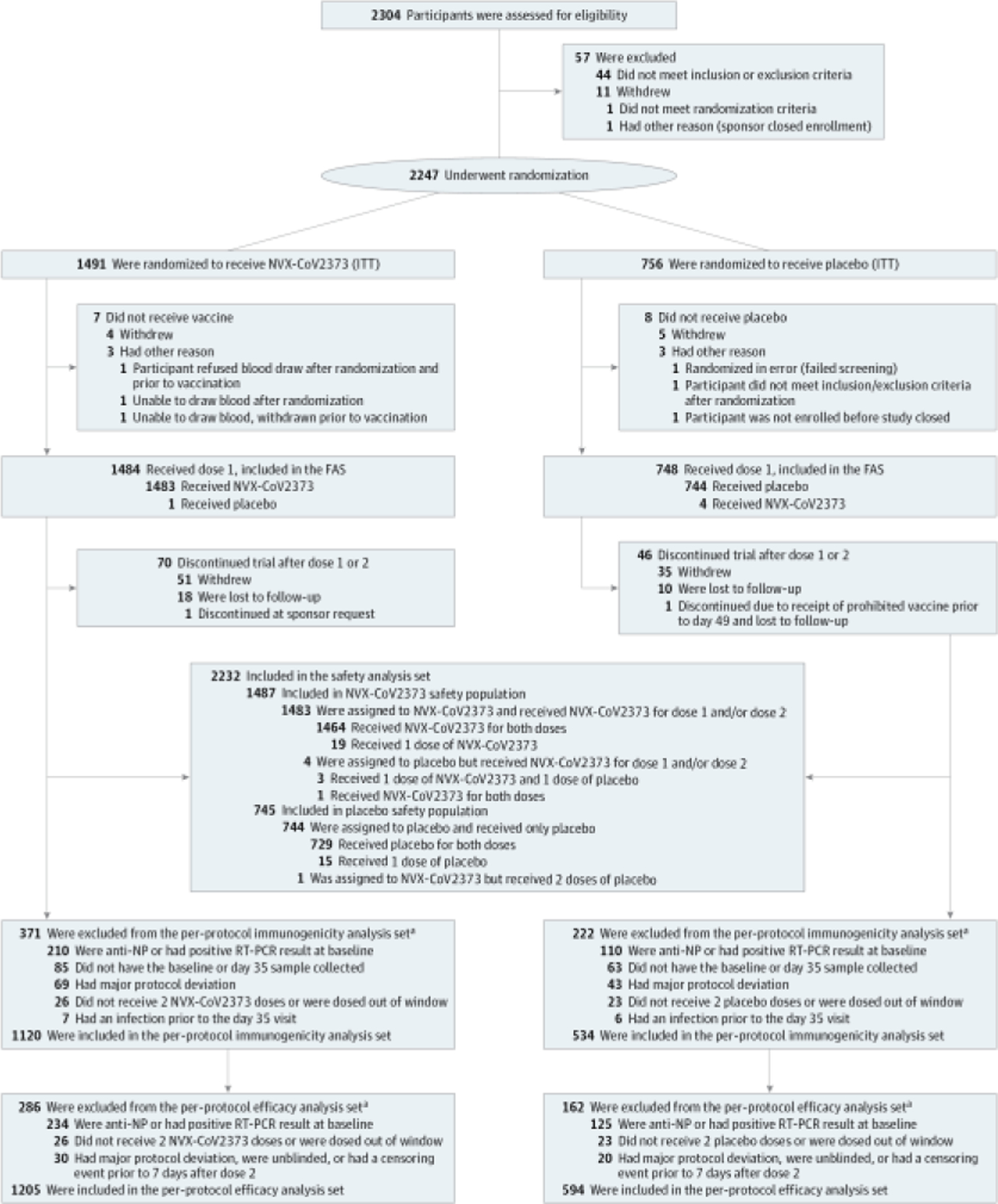
Trial Disposition The full analysis set (FAS) included all participants who were randomly assigned to treatment and received at least 1 dose, regardless of protocol violations or missing data, and are analyzed according to the trial vaccine group as randomized. ITT indicates intention to treat; NP, nucleoprotein; and RT-PCR, reverse transcriptase–polymerase chain reaction. ^a^ Participants could have more than 1 reason for exclusion.

A total of 2304 participants were screened and 2247 were randomized between April 26 and June 5, 2021 (Figure 1). The safety analysis set included 2232 participants who received at least 1 dose of NVX-CoV2373 (n = 1487) or placebo (n = 745). A total of 1799 participants (80.1% of all randomized) were included in the per-protocol efficacy population and 1654 (73.6%) in the per-protocol immunogenicity population based on evidence of previous SARS-CoV-2 infection at baseline (234 [15.7%] vs 125 [16.8%] in active vs placebo groups, respectively) and/or other exclusionary criteria (Figure 1 and Table 1). The baseline demographic characteristics of the safety analysis set were well balanced between treatment groups: 1060 (47.5%) self-identified as female and 1172 (52.5%) as male; 310 (13.9%) identified as African American or Black, 46 (2.1%) as American Indian or Alaska Native, 412 (18.5%) as Hispanic or Latino, and 1660 (74.4%) as White; and 359 (16.1%) had previous SARS-CoV-2 infection at baseline. The mean (SD) age was 13.8 (1.4) years; 1498 (67.1%) were aged 12 to 14 years (Table 1). There were no major differences between the demographic characteristics of the safety analysis set and per-protocol populations (efficacy and immunogenicity analysis sets) (eTables 4 and 5 in Supplement 1). The median duration of safety follow-up after second vaccination was 71 (IQR, 65–77) days and was similar between treatment groups (NVX-CoV2373: 71 [IQR, 65–77] days; placebo: 71 [IQR, 64–77] days) (eTable 6 in Supplement 1). There were 18 participants in the NVX-CoV2373 group and 10 in the placebo group who were lost to follow-up and for whom day 35 immunogenicity analyses and RT-PCR–positive COVID-19 assessments could not be made.

### Safety

#### Reactogenicity

**Figure 2.**
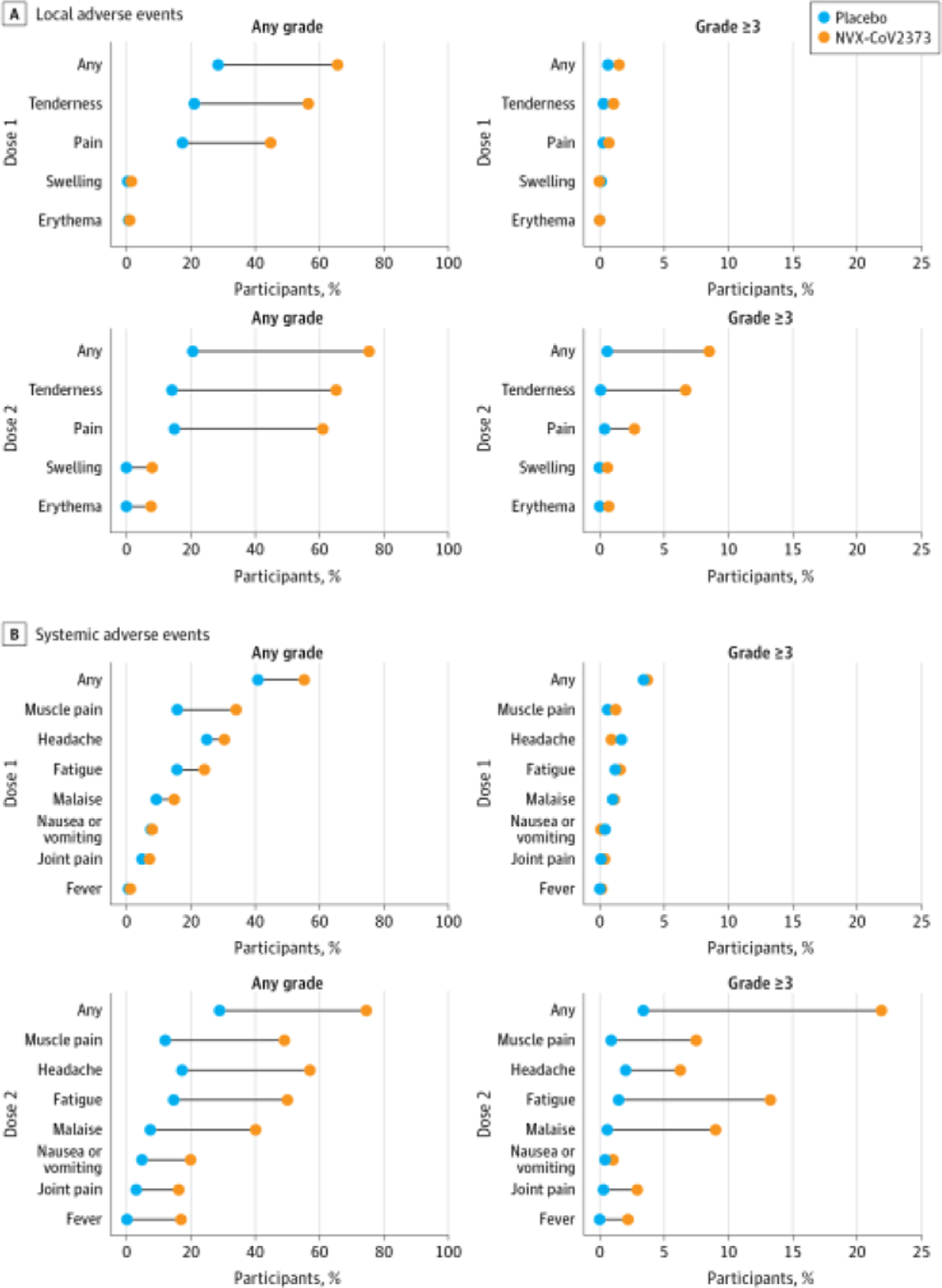
Solicited Local and Systemic Adverse Events The percentage of participants in each treatment group with solicited local (A) and systemic (B) adverse events during the 7 days after each vaccination is plotted by US Food and Drug Administration toxicity grade, as any (mild, moderate, severe, or potentially life-threatening) or as grade 3 or higher (severe or potentially life-threatening).^21^

Solicited local and systemic adverse events were predominantly mild to moderate in severity and self-limited, although more frequent in NVX-CoV2373 recipients and more common after the second injection. After each dose, the most frequently reported solicited local adverse events were injection site pain (NVX-CoV2373: 648 [44.8%] after dose 1 and 850 [61.0%] after dose 2; placebo: 126 [17.4%] after dose 1 and 102 [14.9%] after dose 2) and tenderness (NVX-CoV2373: 817 [56.4%] in dose 1 and 909 [65.2%] in dose 2; placebo: 153 [21.1%] in dose 1 and 97 [14.1%] in dose 2). The median duration of these events was 2 days or less (range, 1–7 days; IQR, 1–3 days) (eTable 7 in Supplement 1). Severe (≥grade 3) local reactions occurred after dose 1 among 22 (1.5%) in the NVX-CoV2373 group vs 5 (0.7%) in the placebo group and after dose 2 among 118 (8.5%) in the NVX-CoV2373 group vs 4 (0.6%) in the placebo group (Figure 2 and eTable 8 in Supplement 1).

The most common solicited systemic adverse events were headache (NVX-CoV2373: 440 [30.4%] after dose 1 and 793 [56.9%] after dose 2; placebo: 181 [24.9%] after dose 1 and 119 [17.3%] after dose 2), fatigue (NVX-CoV2373: 350 [24.2%] after dose 1 and 695 [49.9%] after dose 2; placebo: 113 [15.6%] after dose 1 and 100 [14.6%] after dose 2), myalgia (NVX-CoV2373: 492 [34.0%] after dose 1 and 683 [49.0%] after dose 2; placebo: 114 [15.7%] after dose 1 and 82 [12.0%] after dose 2), and malaise (NVX-CoV2373: 215 [14.8%] after dose 1 and 560 [40.2%] after dose 2; placebo: 67 [9.2%] after dose 1 and 51 [7.4%] after dose 2). These adverse events were also detected more frequently among NVX-CoV2373 recipients and after the second injection, with a median duration of 2 days or less (range, 1–7 days; IQR, 1–2 days) (eTable 9 in Supplement 1). Fever of any severity occurred in 235 recipients (16.9%) in the NVX-CoV2373 group after the second dose. Severe systemic reactions (grade ≥3), most commonly fatigue, occurred after dose 1 in 54 (3.7%) in the NVX-CoV2373 group vs 25 (3.4%) in the placebo group and after dose 2 among 306 (22.0%) in the NVX-CoV2373 group vs 23 (3.4%) in the placebo group (Figure 2 and eTable 10 in Supplement 1). Similar reactogenicity rates occurred in the age subgroups at 12 to 14 and 15 to 17 years (eFigure 2 in Supplement 1).

#### Unsolicited Adverse Events

Unsolicited adverse events occurred with similar frequency in vaccine and placebo recipients (236 [15.9%] and 116 [15.6%], respectively). Reports of medically attended, serious, and severe adverse events were balanced across treatment groups (eTable 11 in Supplement 1). There were no safety events that triggered prespecified pause rules. No episodes of anaphylaxis, vaccine-enhanced COVID-19, Guillain Barré syndrome,^23^ thrombosis with thrombocytopenia syndrome,^24^ or myocarditis and/or pericarditis^25^ were observed (eTables 12 and 13 in Supplement 1). There were no deaths or adverse events of special interest among adolescent trial participants, including multisystem inflammatory syndrome in children.

#### Immunogenicity

The ratio of neutralizing antibody response to SARS-CoV-2 wild-type virus at day 35 for previously unexposed adolescents compared with that observed in similarly unexposed adult PREVENT-19 participants aged 18 to 25 years met all criteria for noninferiority. The GMT ratio point estimate and lower bound of 95% CI was 1.5 (95% CI, 1.3–1.7), and the lower bound of 95% CI of the serologic response difference was −1.0 (95% CI, −2.8 to 0.2) (Table 2).

Neutralizing antibody GMTs and serologic response were markedly higher in vaccine than placebo groups in all age subgroups (eFigure 3 in the Supplement 1). Day 35 serum IgG levels against S proteins of wild-type and more recent variants tested post hoc also demonstrated high antibody levels against all tested variants, including the Omicron subvariants BA.1, BA.2, and BA.5, while hACE2 receptor–binding inhibition antibody results were generally comparable to the IgG levels, albeit with a trend for lower titers for the Omicron subvariants (eFigures 4-7 in Supplement 1).

#### Efficacy

**Figure 3.**
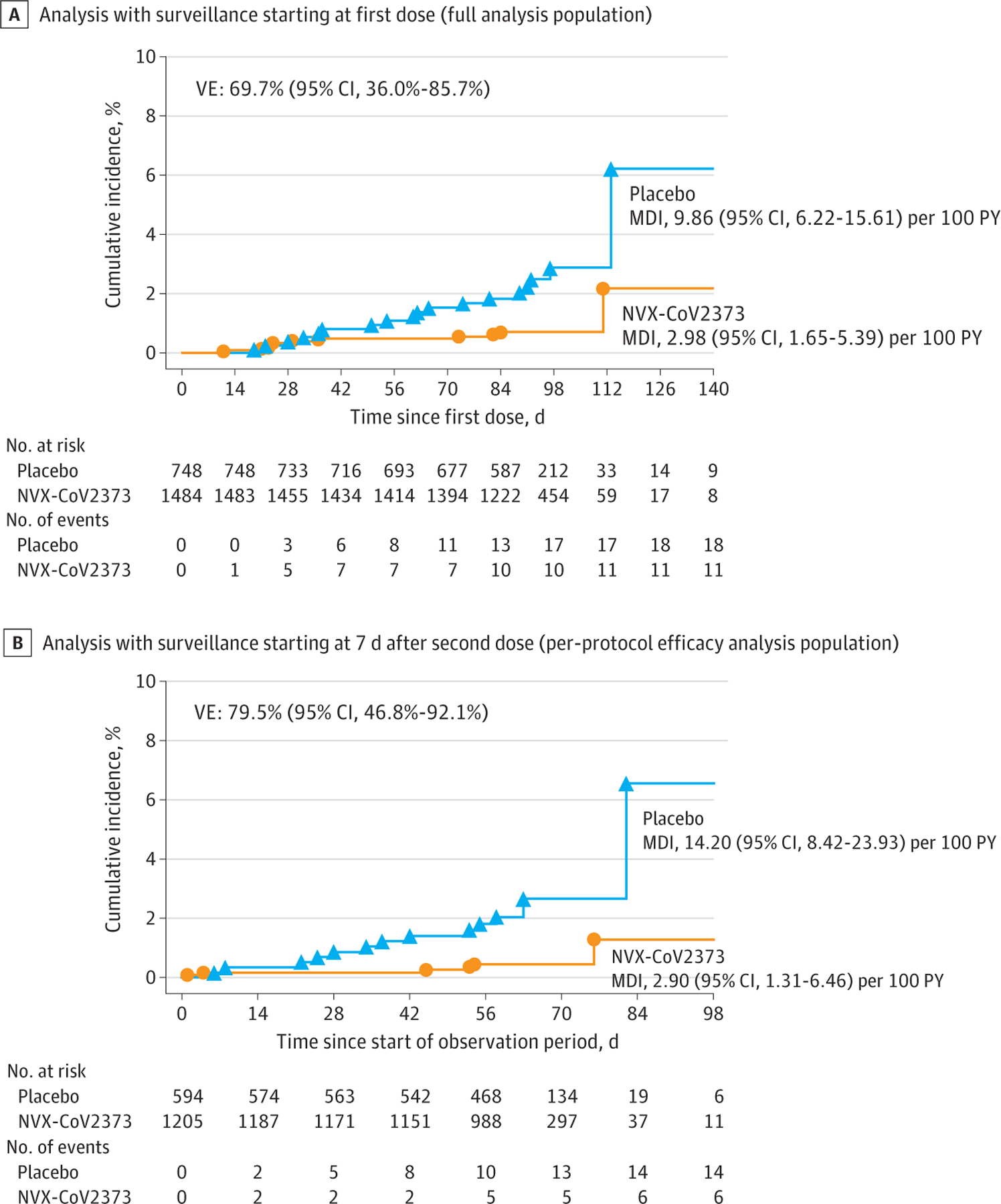
Cumulative Incidence Plot of Overall Efficacy of NVX-CoV2373 Against Symptomatic COVID-19 Prospective surveillance of COVID-19 illness in the full analysis population started from the first dose of NVX-CoV2373 or placebo. The per-protocol symptomatic COVID-19 cases were defined as beginning at least 7 days after the second dose (ie, day 28) through approximately 3 to 4 months of follow-up (the implementation of blinded crossover), unblinding or receipt of emergency use authorization vaccine. MDI indicates mean disease incidence; PY, person-years; and VE, vaccine efficacy.

In the full analysis set, the incidence of COVID-19 was 9.86 (95% CI, 6.22–15.61) cases per 100 person-years in the placebo group and 2.98 (95% CI, 1.65–5.39) cases per 100 person-years in the NVX-CoV2373 group (eTable 14 in Supplement 1), with cumulative incidence curves separating after day 21 (Figure 3A). Among the 1799 participants in the per-protocol efficacy population followed up through September 27, 2021 (median surveillance time, 64 [IQR, 57–69; range, 1–135] days) (eTable 15 in Supplement 1), 20 COVID-19 cases occurred overall (incidence, 14.20 [95% CI, 8.42–23.93] cases per 100 person-years in placebo recipients and 2.90 [95% CI, 1.31–6.46] cases per 100 person-years in vaccine recipients) (Figure 3B). The 6 cases in NVX-CoV2373 recipients and 14 in placebo recipients yielded a vaccine efficacy of 79.5% (95% CI, 46.8%−92.1%) (eTable 16 in Supplement 1). All cases were mild in severity; thus, vaccine efficacy against moderate-to-severe COVID-19 could not be established. Nasal swabs from 11 of 20 end point cases (55.0%), 3 in vaccine and 8 in placebo recipients, yielded sequencing results. All 11 cases were identified as the Delta variant, yielding a vaccine efficacy of 82.0% (95% CI, 32.4%−95.2%) (eTable 17 in Supplement 1).

## Discussion

The expansion of the ongoing PREVENT-19 trial into more than 2200 racially and ethnically diverse adolescents in the US demonstrates that NVX-CoV2373 appears safe and effective (as determined by predefined immunogenicity criteria). Neutralizing antibody responses on postvaccination day 35 were noninferior for both GMFR and serologic response to those observed in young adults from PREVENT-19, in whom a high degree of protective efficacy (90.4% [95% CI, 82.9%−94.6%]) was demonstrated.^13^ Furthermore, protective efficacy of 79.5% (95% CI, 46.8%−92.1%) was demonstrated in the adolescents in a period with predominant circulation of the Delta variant.

The high short-term vaccine efficacy of NVX-CoV2373 for the prevention of COVID-19 in adolescents aged 12 through 17 years corroborated the earlier results from the adult portion of the study.^13^ These vaccine efficacy results were also consistent with those observed for mRNA vaccines in this age group. However, unlike phase 3 trials characterizing the efficacy of mRNA vaccines in adolescents,^26,27^ vaccine efficacy in this pediatric expansion was established during a period of almost exclusive circulation of the Delta variant, the only variant detected in all cases that yielded sequencing results (vaccine efficacy against Delta, 82.0% [95% CI, 32.4%−95.2%]). Even though vaccine efficacy was specified as a descriptive analysis in the pediatric expansion, the results recapitulate the high vaccine efficacy observed for NVX-CoV2373 largely against early viral variants during earlier phase 3 trials in adults,^12,13^ which suggests that the vaccine may elicit broadly protective immunity.

No safety concerns were identified during the follow-up period reported herein (median, 71 [IQR, 65–77] days after dose 2, with >84% of participants followed up for at least 60 days for this analysis). Reactogenicity was mild to moderate in severity, self-limited, and, as expected, more frequent and more severe after the second vaccination. By contrast, similar rates of unsolicited adverse events (including serious or severe adverse events) were observed between vaccine and placebo recipients. However, given the sample size, this study did not have the power to identify rare adverse events, such as myocarditis following vaccination.

Neutralizing antibodies and anti–S-binding IgG antibodies at day 35 (ie, 14 days after the second vaccine dose) have been correlated with vaccine efficacy for NVX-CoV2373.^28^ High levels of humoral responses at day 35 were observed in adolescents (as determined by both anti–S-binding IgG antibodies and functional microneutralization and hACE2 receptor–binding inhibition assays) against prototype virus as well as against more recent Alpha, Beta, Delta, Gamma, Mu, and Omicron variants, including Omicron subvariants BA.1, BA.2, and BA.5 (eFigures 3-7 in Supplement 1), which were 2 to 4 times higher than those observed in PREVENT-19 adult participants (G.A, S.C.-C., M.Z, et al; unpublished data, December 2022).

### Limitations

This study has limitations, including its short period of time (median surveillance time: 64 [IQR, 57–69] days) (eTable 14 in Supplement 1) during which the vaccine efficacy of the primary series of 2 doses of vaccine 21 days apart was evaluated. Placebo-controlled follow-up was limited by early implementation of the blinded crossover to ensure retention of this age group that provided active vaccination for all participants when other vaccines became available under emergency use authorizations,^13^ which overall limited the ability to assess the efficacy of the vaccine against a larger number of viral variants.

Notably, the protective efficacy was assessed during the predominant circulation of the Delta variant, which was later replaced by the Omicron variant. However, the post hoc analyses of immune responses against the more recent Omicron subvariants support potential effectiveness against a broad distribution of future variants (eFigures 6 and 7 in Supplement 1). The effect of an NVX-CoV2373 booster dose given 5 to 6 months after the primary series is being assessed for all PREVENT-19 participants, including those exposed to Omicron.^29,30^

Although the study was not powered for the assessment of vaccine efficacy, another limitation of the study was the low number of cases that were accrued in each group due to the implementation of the blinded crossover, which made the 95% CIs appear wide. However, the lower bound of the vaccine efficacy 95% CI was over the threshold of 30% established by the US FDA to grant emergency use authorization to COVID-19 vaccines.^20^ Another limitation of the study was the fact that, in all COVID-19 studies conducted in 2020 to 2021, the per-protocol efficacy population excluded participants seropositive at baseline, which was in line with the serostatus of the US population at the time the study was initiated and was the population for which the regulators primarily wanted to understand the efficacy of the vaccine.^20^

## Conclusions

In this randomized clinical trial, NVX-CoV2373 was safe, immunogenic, and efficacious in preventing COVID-19, including the predominant Delta variant, in adolescents. NVX-CoV2373 is currently authorized for emergency use in the US among adults and adolescents 12 years or older.^11,14^ The vaccine is expected to increase uptake in adolescents, more than 22% of whom have not yet received a full vaccination regimen with mRNA vaccines.^31^ A favorable safety profile, convenient storage and transportation requirements, and induction of broad, cross-reactive immune responses with the potential to provide protection against new variants suggest that NVX-CoV2373 offers an important choice for vaccination of younger individuals in the fight against the current COVID-19 pandemic worldwide.

## Supplementary Material

Supplement 1 Supplementary Online Content SUPPLEMENT 1.
**eAppendix.** Data and Safety Monitoring Board (DSMB) Members List
**eMethods.** Data Accrual and Analysis
**eFigure 1.** Circulating Variant Strains of SARS-CoV-2 During Study Conduct
**eTable 1.** Primary and Secondary Objectives and End Points Addressed in This Manuscript, Protocol Version 8.0
**eTable 2.** End Point Definitions of COVID-19 Severity
**eTable 3.** Symptoms Suggestive of COVID-19
**eTable 4.** Baseline Demographic Characteristics of the Per-Protocol Efficacy Analysis Set
**eTable 5.** Baseline Demographic Characteristics of the Per-Protocol Immunogenicity Analysis Set
**eTable 6.** Duration (Days) of Safety Follow-up Post Dose 2 Precrossover (Safety Analysis Set)
**eTable 7.** Duration (Days) of Solicited Local Adverse Events Within 7 Days After Dose 1 and Dose 2 in All Participants (Safety Analysis Set)
**eTable 8.** Summary of Solicited Local Adverse Events Within 7 Days After Dose 1 and Dose 2 in All Participants (Safety Analysis Set)
**eTable 9.** Duration (Days) of Solicited Systemic Adverse Events Within 7 Days After Dose 1 and Dose 2 in All Participants (Safety Analysis Set)
**eTable 10.** Summary of Solicited Systemic Adverse Events Within 7 Days After Dose 1 and Dose 2 by Age Group (Safety Analysis Set)
**eFigure 2.** Solicited Local and Systemic Adverse Events by Age Subgroup
**eTable 11.** Overall Summary of Treatment-Emergent Adverse Events Reported Between Start of First Vaccination and Blinded Crossover or Early Withdrawal (Safety Analysis Set)
**eTable 12.** Overall Summary of Unsolicited Adverse Events by System Organ Class and Preferred Term Reported Within 49 Days After First Vaccination in at Least 0.5% of All Adolescent Participants in Any Study Vaccine Group by Age Strata (Safety Analysis Set)
**eTable 13.** Overall Summary of Unsolicited Serious Adverse Events From Start of First Vaccination to Blinded Crossover Dose in All Adolescent Participants in Any Study Vaccine Group by Age Strata (Safety Analysis Set)
**eFigure 3.** Box Plot of Neutralizing Antibody Titers for SARS-CoV-2 Wild-Type Virus at Specified Time Points in Baseline Serologically Negative/PCR-Negative Adolescent Participants
**eFigure 4.** Box Plot of Serum IgG Antibody Concentrations to SARS-CoV-2 S Protein in Baseline Serologically Negative/PCR-Negative Adolescent Participants 12 to <18 Years of Age (PP-IMM Analysis Set)
**eFigure 5.** Box Plot of hACE2 Inhibition Antibodies to SARS-CoV-2 S Protein in Baseline Serologically Negative/ PCR-Negative Adolescent Participants 12 to <18 Years of Age (PP-IMM Analysis Set)
**eFigure 6.** Serum IgG Antibody Concentrations to SARS-CoV-2 S Protein From Different Variants in Baseline Serologically Negative/PCR-Negative Adolescent Participants 12 to <18 Years of Age
**eFigure 7.** hACE2 Inhibition Antibodies to SARS-CoV-2 S Protein From Different Variants in Baseline Serologically Negative/PCR-Negative Adolescent Participants 12 to <18 Years of Age
**eTable 14.** Vaccine Efficacy Against RT-PCR–Confirmed Symptomatic Mild, Moderate, or Severe COVID-19 From First Injection Due to Any SARS-CoV-2 Variant in Adolescent Participants (Full Analysis Set)
**eTable 15.** Duration of Surveillance Time (Days) for Primary Efficacy End Point (Per-Protocol Efficacy Analysis Set)
**eTable 16.** Vaccine Efficacy Against RT-PCR–Confirmed Symptomatic Mild, Moderate, or Severe COVID-19 at Least 7 Days After Second Vaccination Due to Any SARS-CoV-2 Variant in Adolescent Participants Not Previously Exposed to SARS-CoV-2 (PP-EFF Analysis Set)
**eTable 17.** Vaccine Efficacy Against RT-PCR–Confirmed Symptomatic Mild, Moderate, or Severe COVID-19 at Least 7 Days After Second Vaccination Due to the SARS-CoV-2 Delta Variant in Adolescent Participants Not Previously Exposed to SARS-CoV-2 (PP-EFF Analysis Set)
eReferences

Supplement 2 Trial Protocol and Statistical Analysis Plan SUPPLEMENT 2.
Trial Protocol and Statistical Analysis Plan

Supplement 3 Nonauthor Collaborators. 2019nCoV-301−Pediatric Expansion Study Group SUPPLEMENT 3.
**Nonauthor Collaborators.** 2019nCoV-301–Pediatric Expansion Study Group

Supplement 4 Data Sharing Statement SUPPLEMENT 4.
Data Sharing Statement

## Figures and Tables

**Table 1.** Demographic and Baseline Characteristics (Safety Analysis Set Population)

Characteristic	Participant group^a^		
	NVX-CoV2373 (n = 1487)	Placebo (n = 745)	All (N = 2232)
Age, y			
Mean (SD)	13.9 (1.4)	13.8 (1.4)	13.8 (1.4)
Median (range)	14 (12–17)	14 (12–17)	14 (12–17)
Age group, y			
12–14	998 (67.1)	500 (67.1)	1498 (67.1)
15–17	489 (32.9)	245 (32.9)	734 (32.9)
Sex			
Male	756 (50.8)	416 (55.8)	1172 (52.5)
Female	731 (49.2)	329 (44.2)	1060 (47.5)
Race			
African American or Black	202 (13.6)	108 (14.5)	310 (13.9)
American Indian or Alaska Native	32 (2.2)	14 (1.9)	46 (2.1)
Asian	43 (2.9)	34 (4.6)	77 (3.4)
Native Hawaiian or other Pacific Islander	3 (0.2)	2 (0.3)	5 (0.2)
White	1115 (75.0)	545 (73.2)	1660 (74.4)
Multiracial	82 (5.5)	37 (5.0)	119 (5.3)
Not reported	10 (0.7)	5 (0.7)	15 (0.7)
Ethnicity			
Hispanic or Latino	274 (18.4)	138 (18.5)	412 (18.5)
Not Hispanic or Latino	1208 (81.2)	607 (81.5)	1815 (81.3)
Not reported	2 (0.1)	0	2 (0.1)
Unknown	3 (0.2)	0	3 (0.1)
BMI category^b^			
Underweight (<18.0)	40 (2.7)	28 (3.8)	68 (3.0)
Normal (18.0–24.9)	771 (51.8)	417 (56.0)	1188 (53.2)
Overweight	(25.0–29.9)	270 (18.2)	107 (14.4)
Obesity (≥30.0)	406 (27.3)	193 (25.9)	599 (26.8)
Previous SARS-CoV-2 infection status^c^			
Positive	234 (15.7)	125 (16.8)	359 (16.1)
Negative	1252 (84.2)	620 (83.2)	1872 (83.9)
Missing	1 (0.1)	0	1 (0.04)

Abbreviation: BMI, body mass index (calculated as weight in kilograms divided by height in meters squared).

a Unless otherwise indicated, data are expressed as No. (%) of patients. Percentages have been rounded and may not total 100. Percentages are based on the safety analysis set within each treatment and overall.

b Classified (using sex- and age-specific percentiles) as underweight, less than the 5th percentile; healthy, within the 5th percentile and up to the 85th percentile; overweight, within the 85th percentile to less than the 95th percentile; and obesity, equal to or greater than the 95th percentile.

c Indicates either antinucleoprotein or reverse transcriptase–polymerase chain reaction positive findings at baseline.

**Table 2.** Neutralizing Antibody Response in Adolescents Compared With Young Adults in the PREVENT-19 Trial^a^

Age, y	No. of participants	Geometric mean (95% CI)^b^		Serologic response at day 35, % (95% CI)^c^	Difference in serologic response (95% CI)^c^
		Titer at day 35	Titer ratio		
12–17	390	3860 (3423 to 4352)	1.5 (1.3 to 1.7)	98.7 (97.0 to 99.6)	−1.0 (−2.8 to −0.2)
18–25	416	2634 (2398 to 2904)	NA	99.8 (98.7 to 100)	NA

Abbreviation: NA, not applicable.

a Neutralizing antibody titers of adolescents (aged 12–17 years) were compared with those from adult participants (aged 18–25 y). All participants in either age group were part of the per-protocol analysis set (ie, SARS-CoV-2–unexposed participants who had a baseline and ≥1 serum sample result available after full primary vaccination) and had no major protocol violations that were considered clinically relevant to impact immune response at the corresponding study visit (eg, reverse transcriptase–polymerase chain reaction–positive swabs or seropositivity for SARS-CoV-2 prior to the visit in question). Data source: validated microneutralization assay conducted by 360biolabs.^16^

b Calculated based on the *t* distribution of the log-transformed values for geometric means or geometric mean fold rise, then back transformed to the original scale for presentation. Assay results below the lower limit of quantitation (20) were assigned a value of 10 (0.5 times the lower limit of quantitation). The noninferiority criterion was met since the lower bound of the 2-sided 95% CI for the geometric mean ratio was greater than 0.67, and the point estimate was equal to or greater than 0.82.

c Defined as a percentage of participants with a value 4-fold or greater difference between day 35 and day 0. The 95% CI was calculated using the exact Clopper-Pearson method.^22^ The noninferiority criterion was met since the lower bound of the 2-sided 95% CI for the difference of serologic response was greater than −10%.

## Key Points

### Question

Is NVX-CoV2373 safe, immunogenic, and effective in adolescents aged 12 to 17 years?

### Findings

In this phase 3 randomized clinical trial including 2247 adolescents, neutralizing antibody responses were noninferior compared with those of young adults aged 18 to 25 years (effectiveness). Vaccine efficacy was 79.5%, and reactogenicity was mostly mild to moderate and transient; no safety concerns were identified.

### Meaning

These findings indicate that NVX-CoV2373 was safe, immunogenic, and efficacious in preventing COVID-19 in adolescents.
