# Supplementary material for: Safety, Immunogenicity, and Efficacy of the NVX-CoV2373 COVID-19 Vaccine in Adolescents: A Randomized Clinical Trial
Source: JAMA Netw Open. 2023 Apr 26;6(4):e239135. doi: 10.1001/jamanetworkopen.2023.9135 (PMC10536880; doi:10.1001/jamanetworkopen.2023.9135)
Supplement: Supplement 3. — Nonauthor Collaborators. 2019nCoV-301−Pediatric Expansion Study Group [file jamanetwopen-e239135-s003.pdf]

Supplemental Online Content: Nonauthor Collaborators
*First name, last name, and suffix (if applicable) are required and will appear in PubMed.

*Group Name(s): 2019nCoV-301–Pediatric Expansion Study Group										
***First Name and Middle Initial(s)**	***Last Name**	***Suffix (eg, Jr, III)**	Academic Degrees	Institution	Location (city, state/province, country)	Role or Contribution, eg, chair, principal investigator	Group (if more than 1 Group listed in the byline) and/or Subgroup (eg, Steering Committee)			
James	Andersen		MD	Accel Research Sites	Lakeland, FL	Principal investigator	2019nCoV-301 Pediatric Expansion Study Group			
Szheckera	Fearon		MSN, FNP-C	Accel Research Sites	Lakeland, FL	Study team	2019nCoV-301 Pediatric Expansion Study Group			
Rosa	Negron		MD	Accel Research Sites	Lakeland, FL	Study team	2019nCoV-301 Pediatric Expansion Study Group			
Amy	Medina		ADN, BS	Accel Research Sites	Lakeland, FL	Study team	2019nCoV-301 Pediatric Expansion Study Group			
Colleen	Figueroa			Accel Research Sites	Lakeland, FL	Study team	2019nCoV-301 Pediatric Expansion Study Group			
Courtney	Smith			Accel Research Sites	Lakeland, FL	Study team	2019nCoV-301 Pediatric Expansion Study Group			
Bruce	Rankin		DO	Accel Research Sites	DeLand, FL	Study team	2019nCoV-301 Pediatric Expansion Study Group			
John M.	Hill		MD	Accel Research Sites	DeLand, FL	Study team	2019nCoV-301 Pediatric Expansion Study Group			
Steven	Shinn		MD	Accel Research Sites	DeLand, FL	Study team	2019nCoV-301 Pediatric Expansion Study Group			
Vivek	Rajasekhar		DO	Accel Research Sites	DeLand, FL	Study team	2019nCoV-301 Pediatric Expansion Study Group			
Marshall	Nash		MD	Accel Research Sites	DeLand, FL	Study team	2019nCoV-301 Pediatric Expansion Study Group			
Ashraf	Affan		MD	Accel Research Sites	DeLand, FL	Study team	2019nCoV-301 Pediatric Expansion Study Group			
Armando	Acevedo		MD	Acevedo Clinical Research Associates	Miami, FL	Principal investigator	2019nCoV-301 Pediatric Expansion Study Group			
Alina	Monteagudo Can		MD	Acevedo Clinical Research Associates	Miami, FL	Study team	2019nCoV-301 Pediatric Expansion Study Group			
Hector	Rodriguez		MD	Acevedo Clinical Research Associates	Miami, FL	Study team	2019nCoV-301 Pediatric Expansion Study Group			
Israel	Zagales			Acevedo Clinical Research Associates	Miami, FL	Study team	2019nCoV-301 Pediatric Expansion Study Group			
Christine	Prieto			Acevedo Clinical Research Associates	Miami, FL	Study team	2019nCoV-301 Pediatric Expansion Study Group			
Lizz	Hernandez Diaz			Acevedo Clinical Research Associates	Miami, FL	Study team	2019nCoV-301 Pediatric Expansion Study Group			
Max	Hale		MD	Alabama Clinical Therapeutics	Birmingham, AL	Principal investigator	2019nCoV-301 Pediatric Expansion Study Group			
Patrick	Farr		MD	Alabama Clinical Therapeutics	Birmingham, AL	Study team	2019nCoV-301 Pediatric Expansion Study Group			
Liesel	French		MD	Alabama Clinical Therapeutics	Birmingham, AL	Study team	2019nCoV-301 Pediatric Expansion Study Group			
Teresa	Goldsmith		MD	Alabama Clinical Therapeutics	Birmingham, AL	Study team	2019nCoV-301 Pediatric Expansion Study Group			
James	Warmack		MD	Alabama Clinical Therapeutics	Birmingham, AL	Study team	2019nCoV-301 Pediatric Expansion Study Group			
Bailey	Murphy			Alabama Clinical Therapeutics	Birmingham, AL	Study team	2019nCoV-301 Pediatric Expansion Study Group			
Robyn	Hartvickson		MD	Alliance for Multispecialty Research	Newton, KS	Principal investigator	2019nCoV-301 Pediatric Expansion Study Group			
Brooke	Dunlavy		MD	Alliance for Multispecialty Research	Newton, KS	Study team	2019nCoV-301 Pediatric Expansion Study Group			
Richard	Glover		MD	Alliance for Multispecialty Research	Newton, KS	Study team	2019nCoV-301 Pediatric Expansion Study Group			
Amber	Grant		MD	Alliance for Multispecialty Research	Newton, KS	Study team	2019nCoV-301 Pediatric Expansion Study Group			
Troy	Holderman		MD	Alliance for Multispecialty Research	Newton, KS	Study team	2019nCoV-301 Pediatric Expansion Study Group			
Stacy	Slechta		MD	Alliance for Multispecialty Research	Newton, KS	Study team	2019nCoV-301 Pediatric Expansion Study Group			
Terry	Poling		MD	Alliance for Multispecialty Research	Wichita, KS	Principal investigator	2019nCoV-301 Pediatric Expansion Study Group			
Terry	Klein			Alliance for Multispecialty Research	Wichita, KS	Study team	2019nCoV-301 Pediatric Expansion Study Group			
Thomas	Klein			Alliance for Multispecialty Research	Wichita, KS	Study team	2019nCoV-301 Pediatric Expansion Study Group			
Tracy	Klein			Alliance for Multispecialty Research	Wichita, KS	Study team	2019nCoV-301 Pediatric Expansion Study Group			
Sarah	Pinkham			Alliance for Multispecialty Research	Wichita, KS	Study team	2019nCoV-301 Pediatric Expansion Study Group			
Shannen	Lassiter			Alliance for Multispecialty Research	Wichita, KS	Study team	2019nCoV-301 Pediatric Expansion Study Group			
Imad	Jandali		MD	Asclepes Research Centers	Spring Hill, FL	Principal investigator	2019nCoV-301 Pediatric Expansion Study Group			
Maryam	Belavilas		MD	Asclepes Research Centers	Spring Hill, FL	Study team	2019nCoV-301 Pediatric Expansion Study Group			
David	Daniels			Asclepes Research Centers	Spring Hill, FL	Study team	2019nCoV-301 Pediatric Expansion Study Group			
Katie	Leonard			Asclepes Research Centers	Spring Hill, FL	Study team	2019nCoV-301 Pediatric Expansion Study Group			
Abigail	Vetter			Asclepes Research Centers	Spring Hill, FL	Study team	2019nCoV-301 Pediatric Expansion Study Group			
Toni	Rich			Asclepes Research Centers	Spring Hill, FL	Study team	2019nCoV-301 Pediatric Expansion Study Group			

Supplemental Online Content: Nonauthor Collaborators
*First name, last name, and suffix (if applicable) are required and will appear in PubMed.

*First Name and Middle Initial(s)	*Last Name	*Suffix (eg, Jr, III)	Academic Degrees	Institution	Location (city, state/province, country)	Role or Contribution, eg, chair, principal investigator	Group (if more than 1 Group listed in the byline) and/or Subgroup (eg, Steering Committee)			
Samir	Arora		MD	Aventiv Research	Columbus, OH	Principal investigator	2019nCoV-301 Pediatric Expansion Study Group			
Grazia	Cannon		MD	Aventiv Research	Columbus, OH	Study team	2019nCoV-301 Pediatric Expansion Study Group			
Sridhar	Guduri		MD	Aventiv Research	Columbus, OH	Study team	2019nCoV-301 Pediatric Expansion Study Group			
Alandra	Lingel		MD	Aventiv Research	Columbus, OH	Study team	2019nCoV-301 Pediatric Expansion Study Group			
Veronica	Moore		MD	Aventiv Research	Columbus, OH	Study team	2019nCoV-301 Pediatric Expansion Study Group			
Sarah	Wilcox		MD	Aventiv Research	Columbus, OH	Study team	2019nCoV-301 Pediatric Expansion Study Group			
Richard	Gorman		MD	Biomedical Advanced Research and Development Authority (BARDA)	Washington, DC	Study team	2019nCoV-301 Pediatric Expansion Study Group			
Gary	Horwith		MD	Biomedical Advanced Research and Development Authority (BARDA)	Washington, DC	Study team	2019nCoV-301 Pediatric Expansion Study Group			
Robin	Mason		MS, MBA	Biomedical Advanced Research and Development Authority (BARDA)	Washington, DC	Study team	2019nCoV-301 Pediatric Expansion Study Group			
Laurence	Chu		MD	Benchmark Research	Austin, TX	Principal investigator	2019nCoV-301 Pediatric Expansion Study Group			
Michelle	Listz			Benchmark Research	Austin, TX	Study team	2019nCoV-301 Pediatric Expansion Study Group			
Lamar	Box			Benchmark Research	Austin, TX	Study team	2019nCoV-301 Pediatric Expansion Study Group			
Cindy	Duran			Benchmark Research	Austin, TX	Study team	2019nCoV-301 Pediatric Expansion Study Group			
Isaiah	Knight			Benchmark Research	Austin, TX	Study team	2019nCoV-301 Pediatric Expansion Study Group			
Katherine	Davis			Benchmark Research	Austin, TX	Study team	2019nCoV-301 Pediatric Expansion Study Group			
William	Seger		MD	Benchmark Research	Fort Worth, TX	Principal investigator	2019nCoV-301 Pediatric Expansion Study Group			
John	Villegas			Benchmark Research	Fort Worth, TX	Study team	2019nCoV-301 Pediatric Expansion Study Group			
Ben	Seger			Benchmark Research	Fort Worth, TX	Study team	2019nCoV-301 Pediatric Expansion Study Group			
Virginia	Loudermilk			Benchmark Research	Fort Worth, TX	Study team	2019nCoV-301 Pediatric Expansion Study Group			
Ruth	Reyes			Benchmark Research	Fort Worth, TX	Study team	2019nCoV-301 Pediatric Expansion Study Group			
Anthony	Kim			Benchmark Research	Fort Worth, TX	Study team	2019nCoV-301 Pediatric Expansion Study Group			
Wendy C	Daly		MD	Brownsboro Park Pediatrics	Louisville, KY	Principal investigator	2019nCoV-301 Pediatric Expansion Study Group			
Rebecca	Becherer		MD	Brownsboro Park Pediatrics	Louisville, KY	Study team	2019nCoV-301 Pediatric Expansion Study Group			
Denver	Cornett		MD	Brownsboro Park Pediatrics	Louisville, KY	Study team	2019nCoV-301 Pediatric Expansion Study Group			
Karen	Dick		MD	Brownsboro Park Pediatrics	Louisville, KY	Study team	2019nCoV-301 Pediatric Expansion Study Group			
Kimberly	Downs		MD	Brownsboro Park Pediatrics	Louisville, KY	Study team	2019nCoV-301 Pediatric Expansion Study Group			
Pamela	Hall		MD	Brownsboro Park Pediatrics	Louisville, KY	Study team	2019nCoV-301 Pediatric Expansion Study Group			
Donald M.	Brandon		MD	California Research Foundation	San Diego, CA	Principal investigator	2019nCoV-301 Pediatric Expansion Study Group			
William B.	Davis		MD	California Research Foundation	San Diego, CA	Study team	2019nCoV-301 Pediatric Expansion Study Group			
Daniel T.	Lawler		MD	California Research Foundation	San Diego, CA	Study team	2019nCoV-301 Pediatric Expansion Study Group			
Cindy	Stevens		MD	California Research Foundation	San Diego, CA	Study team	2019nCoV-301 Pediatric Expansion Study Group			
Karl	Walter		MD	California Research Foundation	San Diego, CA	Study team	2019nCoV-301 Pediatric Expansion Study Group			
Michelle	Rios			California Research Foundation	San Diego, CA	Study team	2019nCoV-301 Pediatric Expansion Study Group			
Howard	Schwartz		MD	Cenexel RCA	Hollywood, FL	Principal investigator	2019nCoV-301 Pediatric Expansion Study Group			
Nelia	Sanchez-Crespo			Cenexel RCA	Hollywood, FL	Study team	2019nCoV-301 Pediatric Expansion Study Group			
Thelma	Beltron			Cenexel RCA	Hollywood, FL	Study team	2019nCoV-301 Pediatric Expansion Study Group			
Jennifer	Schwartz			Cenexel RCA	Hollywood, FL	Study team	2019nCoV-301 Pediatric Expansion Study Group			

Supplemental Online Content: Nonauthor Collaborators
*First name, last name, and suffix (if applicable) are required and will appear in PubMed.

*First Name and Middle Initial(s)	*Last Name	*Suffix (eg, Jr, III)	Academic Degrees	Institution	Location (city, state/province, country)	Role or Contribution, eg, chair, principal investigator	Group (if more than 1 Group listed in the byline) and/or Subgroup (eg, Steering Committee)			
Patricia	Balebona			Cenexel RCA	Hollywood, FL	Study team	2019nCoV-301 Pediatric Expansion Study Group			
Beatriz	Rivera			Cenexel RCA	Hollywood, FL	Study team	2019nCoV-301 Pediatric Expansion Study Group			
Barbara	Garcia			Cenexel RCA	Hollywood, FL	Study team	2019nCoV-301 Pediatric Expansion Study Group			
Christine B.	Turley		MD	Charlotte-Mecklenburg Hospital Auth	Charlotte, NC	Principal investigator	2019nCoV-301 Pediatric Expansion Study Group			
Andrew	McWilliams		MD	Charlotte-Mecklenburg Hospital Auth	Charlotte, NC	Study team	2019nCoV-301 Pediatric Expansion Study Group			
Tiffany	Esinhart		PA-C	Charlotte-Mecklenburg Hospital Auth	Charlotte, NC	Study team	2019nCoV-301 Pediatric Expansion Study Group			
Natasha	Montoya		APRN	Charlotte-Mecklenburg Hospital Auth	Charlotte, NC	Study team	2019nCoV-301 Pediatric Expansion Study Group			
Shamika	Huskey		FNP	Charlotte-Mecklenburg Hospital Auth	Charlotte, NC	Study team	2019nCoV-301 Pediatric Expansion Study Group			
Leena	Paul		FNP	Charlotte-Mecklenburg Hospital Auth	Charlotte, NC	Study team	2019nCoV-301 Pediatric Expansion Study Group			
Michael E.	Dever		MD	Clinical Neuroscience Solutions	Orlando, FL	Principal investigator	2019nCoV-301 Pediatric Expansion Study Group			
Mitul	Shah		MD	Clinical Neuroscience Solutions	Orlando, FL	Study team	2019nCoV-301 Pediatric Expansion Study Group			
Michael	Delgado		MD	Clinical Neuroscience Solutions	Orlando, FL	Study team	2019nCoV-301 Pediatric Expansion Study Group			
Tameika Scott	Scott		DrPH	Clinical Neuroscience Solutions	Orlando, FL	Study team	2019nCoV-301 Pediatric Expansion Study Group			
Patricia	Brown		PhD	Clinical Neuroscience Solutions	Orlando, FL	Study team	2019nCoV-301 Pediatric Expansion Study Group			
Americo	Padilla		MD	Clinical Neuroscience Solutions	Orlando, FL	Study team	2019nCoV-301 Pediatric Expansion Study Group			
Lisa S.	Usdan		MD	Clinical Neuroscience Solutions	Memphis, TN	Principal investigator	2019nCoV-301 Pediatric Expansion Study Group			
Lora J.	McGill		MD	Clinical Neuroscience Solutions	Memphis, TN	Study team	2019nCoV-301 Pediatric Expansion Study Group			
Valerie K.	Arnold		MD	Clinical Neuroscience Solutions	Memphis, TN	Study team	2019nCoV-301 Pediatric Expansion Study Group			
Carolyn	Scatamacchia		MSN, NP-C	Clinical Neuroscience Solutions	Memphis, TN	Study team	2019nCoV-301 Pediatric Expansion Study Group			
Codi	Anthony		DNP, APRN, PMH	Clinical Neuroscience Solutions	Memphis, TN	Study team	2019nCoV-301 Pediatric Expansion Study Group			
Robyn	Presley			Clinical Neuroscience Solutions	Memphis, TN	Study team	2019nCoV-301 Pediatric Expansion Study Group			
Stephan	Sharp		MD	Clinical Research Associates	Nashville, TN	Principal investigator	2019nCoV-301 Pediatric Expansion Study Group			
Michael	Caldwell		MD	Clinical Research Associates	Nashville, TN	Study team	2019nCoV-301 Pediatric Expansion Study Group			
Linda	Schipani		RN	Clinical Research Associates	Nashville, TN	Study team	2019nCoV-301 Pediatric Expansion Study Group			
Allison	Ulrich		RN	Clinical Research Associates	Nashville, TN	Study team	2019nCoV-301 Pediatric Expansion Study Group			
Wendy	Tidwell			Clinical Research Associates	Nashville, TN	Study team	2019nCoV-301 Pediatric Expansion Study Group			
Stacy Lynn	Cox		APN, RN	Clinical Research Associates	Nashville, TN	Study team	2019nCoV-301 Pediatric Expansion Study Group			
Michael	Levin		MD	Clinical Research Center of Nevada	Las Vegas, NV	Principal investigator	2019nCoV-301 Pediatric Expansion Study Group			
Julia	Gass			Clinical Research Center of Nevada	Las Vegas, NV	Study team	2019nCoV-301 Pediatric Expansion Study Group			
Marcy	Kulic		MD	Clinical Research Center of Nevada	Las Vegas, NV	Study team	2019nCoV-301 Pediatric Expansion Study Group			
Eduardo	Rodriguez			Clinical Research Center of Nevada	Las Vegas, NV	Study team	2019nCoV-301 Pediatric Expansion Study Group			
Jessica	Corea			Clinical Research Center of Nevada	Las Vegas, NV	Study team	2019nCoV-301 Pediatric Expansion Study Group			
Sierra	Dansbee			Clinical Research Center of Nevada	Las Vegas, NV	Study team	2019nCoV-301 Pediatric Expansion Study Group			
John	Delgado		MD	Clinical Research Institute of Souther	Medford, OR	Principal investigator	2019nCoV-301 Pediatric Expansion Study Group			
Jaleh	Ostovar		NP	Clinical Research Institute of Souther	Medford, OR	Study team	2019nCoV-301 Pediatric Expansion Study Group			
Audrey	Kuehl			Clinical Research Institute of Souther	Medford, OR	Study team	2019nCoV-301 Pediatric Expansion Study Group			
Sarah	Smiley			Clinical Research Institute of Souther	Medford, OR	Study team	2019nCoV-301 Pediatric Expansion Study Group			
Danuel	Hamlin			Clinical Research Institute of Souther	Medford, OR	Study team	2019nCoV-301 Pediatric Expansion Study Group			
Ben	Taucher			Clinical Research Institute of Souther	Medford, OR	Study team	2019nCoV-301 Pediatric Expansion Study Group			
Cayce	Tangeman		MD	Coastal Carolina Research Center	North Charleston, SC	Principal investigator	2019nCoV-301 Pediatric Expansion Study Group			
Yvonne	Davis		MD	Coastal Carolina Research Center	North Charleston, SC	Study team	2019nCoV-301 Pediatric Expansion Study Group			
Vanessa	Armetta			Coastal Carolina Research Center	North Charleston, SC	Study team	2019nCoV-301 Pediatric Expansion Study Group			
Mary	Love			Coastal Carolina Research Center	North Charleston, SC	Study team	2019nCoV-301 Pediatric Expansion Study Group			

Supplemental Online Content: Nonauthor Collaborators
*First name, last name, and suffix (if applicable) are required and will appear in PubMed.

*First Name and Middle Initial(s)	*Last Name	*Suffix (eg, Jr, III)	Academic Degrees	Institution	Location (city, state/province, country)	Role or Contribution, eg, chair, principal investigator	Group (if more than 1 Group listed in the byline) and/or Subgroup (eg, Steering Committee)			
David	Summers		RN	Coastal Carolina Research Center	North Charleston, SC	Study team	2019nCoV-301 Pediatric Expansion Study Group			
J. Bruce	Etheridge		MD	Coastal Carolina Research Center	North Charleston, SC	Study team	2019nCoV-301 Pediatric Expansion Study Group			
Teresita	Salazar		MD	Coast Clinical Research	Bellflower, CA	Principal investigator	2019nCoV-301 Pediatric Expansion Study Group			
Femina	David		MD	Coast Clinical Research	Bellflower, CA	Study team	2019nCoV-301 Pediatric Expansion Study Group			
Filipinas	Vitug			Coast Clinical Research	Bellflower, CA	Study team	2019nCoV-301 Pediatric Expansion Study Group			
Amiel	Guevarra			Coast Clinical Research	Bellflower, CA	Study team	2019nCoV-301 Pediatric Expansion Study Group			
Noemi	Ramirez			Coast Clinical Research	Bellflower, CA	Study team	2019nCoV-301 Pediatric Expansion Study Group			
Ronald	Ackerman		MD	Comprehensive Clinical Research	West Palm Beach, FL	Principal investigator	2019nCoV-301 Pediatric Expansion Study Group			
Jamie	Ackerman			Comprehensive Clinical Research	West Palm Beach, FL	Study team	2019nCoV-301 Pediatric Expansion Study Group			
Florida	Aristy		APRN	Comprehensive Clinical Research	West Palm Beach, FL	Study team	2019nCoV-301 Pediatric Expansion Study Group			
Lawrence	Corey		MD	COVID-19 Prevention Network (CoVP	Seattle, WA	Study team	2019nCoV-301 Pediatric Expansion Study Group			
Kathleen M	Neuzil		MD, MPH	COVID-19 Prevention Network (CoVP	Seattle, WA	Study team	2019nCoV-301 Pediatric Expansion Study Group			
Huub G	Gelderblom		MD, PhD	COVID-19 Prevention Network (CoVP	Seattle, WA	Study team	2019nCoV-301 Pediatric Expansion Study Group			
Nzeera	Ketter		MD	COVID-19 Prevention Network (CoVP	Seattle, WA	Study team	2019nCoV-301 Pediatric Expansion Study Group			
Carrie	Sopher			COVID-19 Prevention Network (CoVP	Seattle, WA	Study team	2019nCoV-301 Pediatric Expansion Study Group			
Vicki E.	Miller		MD	DM Clinical Research	Tomball, TX	Principal investigator	2019nCoV-301 Pediatric Expansion Study Group			
Amy	Starr			DM Clinical Research	Tomball, TX	Study team	2019nCoV-301 Pediatric Expansion Study Group			
Sonia	Guerrero			DM Clinical Research	Tomball, TX	Study team	2019nCoV-301 Pediatric Expansion Study Group			
Madiha	Baig			DM Clinical Research	Tomball, TX	Study team	2019nCoV-301 Pediatric Expansion Study Group			
Maryam	Jamil			DM Clinical Research	Tomball, TX	Study team	2019nCoV-301 Pediatric Expansion Study Group			
Husain	Motiwala			DM Clinical Research	Tomball, TX	Study team	2019nCoV-301 Pediatric Expansion Study Group			
Khozema	Palanpurwala		MD	DM Clinical Research - Pediatric Healt	Houston, TX	Principal investigator	2019nCoV-301 Pediatric Expansion Study Group			
Monica	Murry		MD	DM Clinical Research - Pediatric Healt	Houston, TX	Study team	2019nCoV-301 Pediatric Expansion Study Group			
Amy Starr, MD,	Starr		MD	DM Clinical Research - Pediatric Healt	Houston, TX	Study team	2019nCoV-301 Pediatric Expansion Study Group			
Meghan	Tonti		MD	DM Clinical Research - Pediatric Healt	Houston, TX	Study team	2019nCoV-301 Pediatric Expansion Study Group			
Rebecca	Wischnewsky		MD	DM Clinical Research - Pediatric Healt	Houston, TX	Study team	2019nCoV-301 Pediatric Expansion Study Group			
Earl	Martin		MD	DM Clinical Research - Pediatric Healt	Houston, TX	Study team	2019nCoV-301 Pediatric Expansion Study Group			
Yogesh K.	Paliwal		MD	Empire Clinical Research	Pomona, CA	Principal investigator	2019nCoV-301 Pediatric Expansion Study Group			
Amit	Paliwal		MD	Empire Clinical Research	Pomona, CA	Study team	2019nCoV-301 Pediatric Expansion Study Group			
Sarah	Gordon		MS	Empire Clinical Research	Pomona, CA	Study team	2019nCoV-301 Pediatric Expansion Study Group			
Krystle	Edwards			Empire Clinical Research	Pomona, CA	Study team	2019nCoV-301 Pediatric Expansion Study Group			
Cynthia	Montano-Pereira			Empire Clinical Research	Pomona, CA	Study team	2019nCoV-301 Pediatric Expansion Study Group			
Michael	Campos		MD	Empire Clinical Research	Pomona, CA	Study team	2019nCoV-301 Pediatric Expansion Study Group			
George H.	Freeman		MD	Health Research of Hampton Roads	Newport News, VA	Principal investigator	2019nCoV-301 Pediatric Expansion Study Group			
Esther Laverne	Harmon		ANP	Health Research of Hampton Roads	Newport News, VA	Study team	2019nCoV-301 Pediatric Expansion Study Group			
Marshall A.	Cross		MD	Health Research of Hampton Roads	Newport News, VA	Study team	2019nCoV-301 Pediatric Expansion Study Group			
Kacie	Sales		BSN, RN	Health Research of Hampton Roads	Newport News, VA	Study team	2019nCoV-301 Pediatric Expansion Study Group			
Catherine Q.	Gular		PharmD	Health Research of Hampton Roads	Newport News, VA	Study team	2019nCoV-301 Pediatric Expansion Study Group			
Joseph	Ley		MD	Holston Medical Group	Kingsport, TN	Principal investigator	2019nCoV-301 Pediatric Expansion Study Group			
Amanda	Donoho		MD	Holston Medical Group	Kingsport, TN	Study team	2019nCoV-301 Pediatric Expansion Study Group			
Kimberley	Hunt		MD	Holston Medical Group	Kingsport, TN	Study team	2019nCoV-301 Pediatric Expansion Study Group			
Donald	Lewis		MD	Holston Medical Group	Kingsport, TN	Study team	2019nCoV-301 Pediatric Expansion Study Group			
Stephanie	Tipton		MD	Holston Medical Group	Kingsport, TN	Study team	2019nCoV-301 Pediatric Expansion Study Group			

*First name, last name, and suffix (if applicable) are required and will appear in PubMed.

***First Name and Middle Initial(s)**	***Last Name**	***Suffix (eg, Jr, III)**	Academic Degrees	Institution	Location (city, state/province, country)	Role or Contribution, eg, chair, principal investigator	Group (if more than 1 Group listed in the byline) and/or Subgroup (eg, Steering Committee)			
Emily	Whitaker		MD	Holston Medical Group	Kingsport, TN	Study team	2019nCoV-301 Pediatric Expansion Study Group			
Jeffry	Jacqmein		MD	Jacksonville Center for Clinical Resear	Jacksonville, FL	Principal investigator	2019nCoV-301 Pediatric Expansion Study Group			
Maggie	Bowers		PA-C	Jacksonville Center for Clinical Resear	Jacksonville, FL	Study team	2019nCoV-301 Pediatric Expansion Study Group			
Dawn	Robison		APRN-C	Jacksonville Center for Clinical Resear	Jacksonville, FL	Study team	2019nCoV-301 Pediatric Expansion Study Group			
Victoria	Mosteller		MD	Jacksonville Center for Clinical Resear	Jacksonville, FL	Study team	2019nCoV-301 Pediatric Expansion Study Group			
Janet	Garvey		DNP	Jacksonville Center for Clinical Resear	Jacksonville, FL	Study team	2019nCoV-301 Pediatric Expansion Study Group			
Carlos	Fierro		MD	Johnson County Clin-Trials	Lenexa, KS	Principal investigator	2019nCoV-301 Pediatric Expansion Study Group			
Mary	Easley		BSN, RN	Johnson County Clin-Trials	Lenexa, KS	Study team	2019nCoV-301 Pediatric Expansion Study Group			
Amy Thompson, MD,	Thompson		MD	Johnson County Clin-Trials	Lenexa, KS	Study team	2019nCoV-301 Pediatric Expansion Study Group			
Heather	Barker			Johnson County Clin-Trials	Lenexa, KS	Study team	2019nCoV-301 Pediatric Expansion Study Group			
Mazen	Zari			Johnson County Clin-Trials	Lenexa, KS	Study team	2019nCoV-301 Pediatric Expansion Study Group			
Karol	Moore			Johnson County Clin-Trials	Lenexa, KS	Study team	2019nCoV-301 Pediatric Expansion Study Group			
Daniel	Finn		MD	Kentucky Pediatric/Adult Research	Bardstown, KY	Principal investigator	2019nCoV-301 Pediatric Expansion Study Group			
Lindsay	Blackman		MD	Kentucky Pediatric/Adult Research	Bardstown, KY	Study team	2019nCoV-301 Pediatric Expansion Study Group			
Stanley	Block		MD	Kentucky Pediatric/Adult Research	Bardstown, KY	Study team	2019nCoV-301 Pediatric Expansion Study Group			
Christal	Denton		MD	Kentucky Pediatric/Adult Research	Bardstown, KY	Study team	2019nCoV-301 Pediatric Expansion Study Group			
Martha	Osborn		MD	Kentucky Pediatric/Adult Research	Bardstown, KY	Study team	2019nCoV-301 Pediatric Expansion Study Group			
Robert	Smith		MD	Kentucky Pediatric/Adult Research	Bardstown, KY	Study team	2019nCoV-301 Pediatric Expansion Study Group			
Carl P.	Griffin		MD	Lynn Health Science Institute	Oklahoma City, OK	Principal investigator	2019nCoV-301 Pediatric Expansion Study Group			
William	Schnitz		MD	Lynn Health Science Institute	Oklahoma City, OK	Study team	2019nCoV-301 Pediatric Expansion Study Group			
Raymond	Cornelison		MD	Lynn Health Science Institute	Oklahoma City, OK	Study team	2019nCoV-301 Pediatric Expansion Study Group			
Linda	Lopez			Lynn Health Science Institute	Oklahoma City, OK	Study team	2019nCoV-301 Pediatric Expansion Study Group			
Kim	Hamilton			Lynn Health Science Institute	Oklahoma City, OK	Study team	2019nCoV-301 Pediatric Expansion Study Group			
Kim	Calloway			Lynn Health Science Institute	Oklahoma City, OK	Study team	2019nCoV-301 Pediatric Expansion Study Group			
David B.	Musante		MD	Triangle Orthopaedic Associates, PA	Durham, NC	Principal investigator	2019nCoV-301 Pediatric Expansion Study Group			
William P.	Silver		MD	Triangle Orthopaedic Associates, PA	Durham, NC	Study team	2019nCoV-301 Pediatric Expansion Study Group			
Linda R.	Belhorn		MD	Triangle Orthopaedic Associates, PA	Durham, NC	Study team	2019nCoV-301 Pediatric Expansion Study Group			
Nicholas A.	Viens		MD	Triangle Orthopaedic Associates, PA	Durham, NC	Study team	2019nCoV-301 Pediatric Expansion Study Group			
David	Dellaero		MD	Triangle Orthopaedic Associates, PA	Durham, NC	Study team	2019nCoV-301 Pediatric Expansion Study Group			
Elizabeth	Wilkers		MD	Triangle Orthopaedic Associates, PA	Durham, NC	Study team	2019nCoV-301 Pediatric Expansion Study Group			
Robert	Jeanfreau		MD	MedPharmics	Metairie, LA	Principal investigator	2019nCoV-301 Pediatric Expansion Study Group			
Nicki	Johnson			MedPharmics	Metairie, LA	Study team	2019nCoV-301 Pediatric Expansion Study Group			
Estafania	Bazan			MedPharmics	Metairie, LA	Study team	2019nCoV-301 Pediatric Expansion Study Group			
Davilyn	Roys			MedPharmics	Metairie, LA	Study team	2019nCoV-301 Pediatric Expansion Study Group			
Steven	Darden			MedPharmics	Metairie, LA	Study team	2019nCoV-301 Pediatric Expansion Study Group			
Susan	Jeanfreau		MD	MedPharmics	Metairie, LA	Study team	2019nCoV-301 Pediatric Expansion Study Group			
Paul G.	Matherne		MD	MedPharmics	Gulfport, MS	Principal investigator	2019nCoV-301 Pediatric Expansion Study Group			
Amy	Caldwell		RN	MedPharmics	Gulfport, MS	Study team	2019nCoV-301 Pediatric Expansion Study Group			
Jessica	Stahl		RN	MedPharmics	Gulfport, MS	Study team	2019nCoV-301 Pediatric Expansion Study Group			
Nicole	Guttierrez		RN	MedPharmics	Gulfport, MS	Study team	2019nCoV-301 Pediatric Expansion Study Group			
Cassandra	Beeks		LPN	MedPharmics	Gulfport, MS	Study team	2019nCoV-301 Pediatric Expansion Study Group			
Frank	Eder		MD	Meridian Clinical Research	Binghamton, NY	Principal investigator	2019nCoV-301 Pediatric Expansion Study Group			
Ryan	Little		MD	Meridian Clinical Research	Binghamton, NY	Study team	2019nCoV-301 Pediatric Expansion Study Group			

Supplemental Online Content: Nonauthor Collaborators
*First name, last name, and suffix (if applicable) are required and will appear in PubMed.

*First Name and Middle Initial(s)	*Last Name	*Suffix (eg, Jr, III)	Academic Degrees	Institution	Location (city, state/province, country)	Role or Contribution, eg, chair, principal investigator	Group (if more than 1 Group listed in the byline) and/or Subgroup (eg, Steering Committee)			
Susan	Owen		MD	Meridian Clinical Research	Binghamton, NY	Study team	2019nCoV-301 Pediatric Expansion Study Group			
Heather	Shaw		MD	Meridian Clinical Research	Binghamton, NY	Study team	2019nCoV-301 Pediatric Expansion Study Group			
John	Tarbox		MD	Meridian Clinical Research	Binghamton, NY	Study team	2019nCoV-301 Pediatric Expansion Study Group			
Victoia	Engler			Meridian Clinical Research	Binghamton, NY	Study team	2019nCoV-301 Pediatric Expansion Study Group			
Roni	Gray		APRN	Meridian Clinical Research	Omaha, NE	Study team	2019nCoV-301 Pediatric Expansion Study Group			
Fritz	Raiser		DO	Meridian Clinical Research	Omaha, NE	Study team	2019nCoV-301 Pediatric Expansion Study Group			
Christine	Wilson			Meridian Clinical Research	Omaha, NE	Study team	2019nCoV-301 Pediatric Expansion Study Group			
Tiffany	Nemecek			Meridian Clinical Research	Omaha, NE	Study team	2019nCoV-301 Pediatric Expansion Study Group			
Hannah	Harrington		MPH	Meridian Clinical Research	Omaha, NE	Study team	2019nCoV-301 Pediatric Expansion Study Group			
Charles	Harper		MD	Meridian Clinical Research	Norfolk, NE	Principal investigator	2019nCoV-301 Pediatric Expansion Study Group			
Torie	Johnson			Meridian Clinical Research	Norfolk, NE	Study team	2019nCoV-301 Pediatric Expansion Study Group			
Chelsie	Nutsch		NP	Meridian Clinical Research	Norfolk, NE	Study team	2019nCoV-301 Pediatric Expansion Study Group			
Sally	Eppenbach		NP	Meridian Clinical Research	Norfolk, NE	Study team	2019nCoV-301 Pediatric Expansion Study Group			
Wendell	Lewis		NP	Meridian Clinical Research	Norfolk, NE	Study team	2019nCoV-301 Pediatric Expansion Study Group			
Katlyn	Mace			Meridian Clinical Research	Norfolk, NE	Study team	2019nCoV-301 Pediatric Expansion Study Group			
Brannon C	Perilloux		MD	Meridian Clinical Research	Baton Rouge, LA	Principal investigator	2019nCoV-301 Pediatric Expansion Study Group			
Christopher	Dedon		MD	Meridian Clinical Research	Baton Rouge, LA	Study team	2019nCoV-301 Pediatric Expansion Study Group			
Lori	Cook			Meridian Clinical Research	Baton Rouge, LA	Study team	2019nCoV-301 Pediatric Expansion Study Group			
Zedlitz	Zedlitz			Meridian Clinical Research	Baton Rouge, LA	Study team	2019nCoV-301 Pediatric Expansion Study Group			
Vasavi	Srinivasan			Meridian Clinical Research	Baton Rouge, LA	Study team	2019nCoV-301 Pediatric Expansion Study Group			
Paige	Melner			Meridian Clinical Research	Baton Rouge, LA	Study team	2019nCoV-301 Pediatric Expansion Study Group			
Joanna	Sextor		MD	Meridian Clinical Research	Washington, DC	Principal investigator	2019nCoV-301 Pediatric Expansion Study Group			
Jessica	Long		MD	Meridian Clinical Research	Washington, DC	Study team	2019nCoV-301 Pediatric Expansion Study Group			
Francis	Palumbo		MD	Meridian Clinical Research	Washington, DC	Study team	2019nCoV-301 Pediatric Expansion Study Group			
Devon	Myers			Meridian Clinical Research	Washington, DC	Study team	2019nCoV-301 Pediatric Expansion Study Group			
Caroline	Vleck		MD	Meridian Clinical Research	Washington, DC	Study team	2019nCoV-301 Pediatric Expansion Study Group			
Peter	Warfield		Dr	Meridian Clinical Research	Washington, DC	Study team	2019nCoV-301 Pediatric Expansion Study Group			
Tatiana	Beresnev		MD	National Institute of Allergy and Infectious Diseases (NIAID)/National Institutes of Health (NIH)	Bethesda, MD	Study team	2019nCoV-301 Pediatric Expansion Study Group			
Maryam	Jahromi		MD	National Institute of Allergy and Infectious Diseases (NIAID)/National Institutes of Health (NIH)	Bethesda, MD	Study team	2019nCoV-301 Pediatric Expansion Study Group			
Mary A.	Marovich		MD	National Institute of Allergy and Infectious Diseases (NIAID)/National Institutes of Health (NIH)	Bethesda, MD	Study team	2019nCoV-301 Pediatric Expansion Study Group			
Julia	Hutter		MD	National Institute of Allergy and Infectious Diseases (NIAID)/National Institutes of Health (NIH)	Bethesda, MD	Study team	2019nCoV-301 Pediatric Expansion Study Group			

Supplemental Online Content: Nonauthor Collaborators
*First name, last name, and suffix (if applicable) are required and will appear in PubMed.

***First Name and Middle Initial(s)**	***Last Name**	***Suffix (eg, Jr, III)**	Academic Degrees	Institution	Location (city, state/province, country)	Role or Contribution, eg, chair, principal investigator	Group (if more than 1 Group listed in the byline) and/or Subgroup (eg, Steering Committee)			
Martha	Nason		PhD	National Institute of Allergy and Infectious Diseases (NIAID)/National Institutes of Health (NIH)	Bethesda, MD	Study team	2019nCoV-301 Pediatric Expansion Study Group			
Mark	Leibowitz		MD	National Research Institute	Los Angeles, CA	Principal investigator	2019nCoV-301 Pediatric Expansion Study Group			
Fernanda	Morales			National Research Institute	Los Angeles, CA	Study team	2019nCoV-301 Pediatric Expansion Study Group			
Mike	Delgado			National Research Institute	Los Angeles, CA	Study team	2019nCoV-301 Pediatric Expansion Study Group			
Rosario	Sanchez			National Research Institute	Los Angeles, CA	Study team	2019nCoV-301 Pediatric Expansion Study Group			
Norma	Vega			National Research Institute	Los Angeles, CA	Study team	2019nCoV-301 Pediatric Expansion Study Group			
Gary	Albert			Novavax, Inc.	Gaithersburg, MD	Study Sponsor	2019nCoV-301 Pediatric Expansion Study Group			
Erin	Coston			Novavax, Inc.	Gaithersburg, MD	Study Sponsor	2019nCoV-301 Pediatric Expansion Study Group			
Chinar	Desai			Novavax, Inc.	Gaithersburg, MD	Study Sponsor	2019nCoV-301 Pediatric Expansion Study Group			
Haoua	Dunbar			Novavax, Inc.	Gaithersburg, MD	Study Sponsor	2019nCoV-301 Pediatric Expansion Study Group			
Mark	Eickhoff			Novavax, Inc.	Gaithersburg, MD	Study Sponsor	2019nCoV-301 Pediatric Expansion Study Group			
Renee	Entzminger-Sneed			Novavax, Inc.	Gaithersburg, MD	Study Sponsor	2019nCoV-301 Pediatric Expansion Study Group			
Jenina	Garcia			Novavax, Inc.	Gaithersburg, MD	Study Sponsor	2019nCoV-301 Pediatric Expansion Study Group			
Margaret	Kautz			Novavax, Inc.	Gaithersburg, MD	Study Sponsor	2019nCoV-301 Pediatric Expansion Study Group			
Angela	Lee			Novavax, Inc.	Gaithersburg, MD	Study Sponsor	2019nCoV-301 Pediatric Expansion Study Group			
Maggie	Lewis			Novavax, Inc.	Gaithersburg, MD	Study Sponsor	2019nCoV-301 Pediatric Expansion Study Group			
Patrick	Newingham			Novavax, Inc.	Gaithersburg, MD	Study Sponsor	2019nCoV-301 Pediatric Expansion Study Group			
Patty	Price-Abbott			Novavax, Inc.	Gaithersburg, MD	Study Sponsor	2019nCoV-301 Pediatric Expansion Study Group			
Patty	Reed			Novavax, Inc.	Gaithersburg, MD	Study Sponsor	2019nCoV-301 Pediatric Expansion Study Group			
Kimberly	Cerenze Short			Novavax, Inc.	Gaithersburg, MD	Study Sponsor	2019nCoV-301 Pediatric Expansion Study Group			
Diana	Vegas			Novavax, Inc.	Gaithersburg, MD	Study Sponsor	2019nCoV-301 Pediatric Expansion Study Group			
Bethanie	Wilkinson		PhD	Novavax, Inc.	Gaithersburg, MD	Study Sponsor	2019nCoV-301 Pediatric Expansion Study Group			
Katherine	Smith		MD	Novavax, Inc.	Gaithersburg, MD	Study Sponsor	2019nCoV-301 Pediatric Expansion Study Group			
David L.	Fried		MD	Velocity Clinical Research	Warwick, RI	Principal investigator	2019nCoV-301 Pediatric Expansion Study Group			
Lynne A.	Haughey		MSN, FNP	Velocity Clinical Research	Warwick, RI	Study team	2019nCoV-301 Pediatric Expansion Study Group			
Ariana C.	Stanton		PA-C	Velocity Clinical Research	Warwick, RI	Study team	2019nCoV-301 Pediatric Expansion Study Group			
Monica	Freeman			Velocity Clinical Research	Warwick, RI	Study team	2019nCoV-301 Pediatric Expansion Study Group			
Jacqueline	DiFazio			Velocity Clinical Research	Warwick, RI	Study team	2019nCoV-301 Pediatric Expansion Study Group			
Courtney	Langlois			Velocity Clinical Research	Warwick, RI	Study team	2019nCoV-301 Pediatric Expansion Study Group			
Rosario	Retino		MD	Orange County Research Institute	Ontario, CA	Principal investigator	2019nCoV-301 Pediatric Expansion Study Group			
Alexis	Deniz		MD	Orange County Research Institute	Ontario, CA	Study team	2019nCoV-301 Pediatric Expansion Study Group			
Wendy	Paiva		MD	Orange County Research Institute	Ontario, CA	Study team	2019nCoV-301 Pediatric Expansion Study Group			
Leonel	Pajarillaga		MD	Orange County Research Institute	Ontario, CA	Study team	2019nCoV-301 Pediatric Expansion Study Group			
Lisa	Pyio		MD	Orange County Research Institute	Ontario, CA	Study team	2019nCoV-301 Pediatric Expansion Study Group			
Lendy	Torres		Dr	Orange County Research Institute	Ontario, CA	Study team	2019nCoV-301 Pediatric Expansion Study Group			
Paul	Wisman		MD	Pediatric Research of Charlottesville	Charlottesville, VA	Principal investigator	2019nCoV-301 Pediatric Expansion Study Group			
Carlos	Armengol		MD	Pediatric Research of Charlottesville	Charlottesville, VA	Study team	2019nCoV-301 Pediatric Expansion Study Group			

*First name, last name, and suffix (if applicable) are required and will appear in PubMed.

***First Name and Middle Initial(s)**	***Last Name**	***Suffix (eg, Jr, III)**	Academic Degrees	Institution	Location (city, state/province, country)	Role or Contribution, eg, chair, principal investigator	Group (if more than 1 Group listed in the byline) and/or Subgroup (eg, Steering Committee)			
Gemia	Bouber		MD	Pediatric Research of Charlottesville	Charlottesville, VA	Study team	2019nCoV-301 Pediatric Expansion Study Group			
Peggy	Bressler		MD	Pediatric Research of Charlottesville	Charlottesville, VA	Study team	2019nCoV-301 Pediatric Expansion Study Group			
Alaina	Brown		MD	Pediatric Research of Charlottesville	Charlottesville, VA	Study team	2019nCoV-301 Pediatric Expansion Study Group			
Candyce	Dorsey		Dr	Pediatric Research of Charlottesville	Charlottesville, VA	Study team	2019nCoV-301 Pediatric Expansion Study Group			
Bernard	Grunstra		MD	PMG Research of Bristol	Bristol, TN	Principal investigator	2019nCoV-301 Pediatric Expansion Study Group			
Amy Dye,	Dye			PMG Research of Bristol	Bristol, TN	Study team	2019nCoV-301 Pediatric Expansion Study Group			
Shelby	Gilmer Olds			PMG Research of Bristol	Bristol, TN	Study team	2019nCoV-301 Pediatric Expansion Study Group			
Joshua	Bullen			PMG Research of Bristol	Bristol, TN	Study team	2019nCoV-301 Pediatric Expansion Study Group			
Miranda	Roark			PMG Research of Bristol	Bristol, TN	Study team	2019nCoV-301 Pediatric Expansion Study Group			
Jennie	Eller			PMG Research of Bristol	Bristol, TN	Study team	2019nCoV-301 Pediatric Expansion Study Group			
Elizabeth	Barranco-Santana		MD	Ponce School of Medicine/NIAID (UM1AI148685)	Ponce, Puerto Rico	Principal investigator	2019nCoV-301 Pediatric Expansion Study Group			
Jessica	Rodriguez		MD	Ponce School of Medicine/NIAID (UM1AI148685)	Ponce, Puerto Rico	Study team	2019nCoV-301 Pediatric Expansion Study Group			
Rafael	Mendoza		MD	Ponce School of Medicine/NIAID (UM1AI148685)	Ponce, Puerto Rico	Study team	2019nCoV-301 Pediatric Expansion Study Group			
Karen	Ruperto		MD	Ponce School of Medicine/NIAID (UM1AI148685)	Ponce, Puerto Rico	Study team	2019nCoV-301 Pediatric Expansion Study Group			
Odette	Olivieri		MD	Ponce School of Medicine/NIAID (UM1AI148685)	Ponce, Puerto Rico	Study team	2019nCoV-301 Pediatric Expansion Study Group			
Enrique	Ocaña		MD	Ponce School of Medicine/NIAID (UM1AI148685)	Ponce, Puerto Rico	Study team	2019nCoV-301 Pediatric Expansion Study Group			
Bryce	Palchick		MD	Preferred Primary Care Physicians	Pittsburgh, PA	Principal investigator	2019nCoV-301 Pediatric Expansion Study Group			
Nathan	Bennett		MD	Preferred Primary Care Physicians	Pittsburgh, PA	Study team	2019nCoV-301 Pediatric Expansion Study Group			
Sarah	Sobrosky		MD	Preferred Primary Care Physicians	Pittsburgh, PA	Study team	2019nCoV-301 Pediatric Expansion Study Group			
Michael	Gates		MD	Preferred Primary Care Physicians	Pittsburgh, PA	Study team	2019nCoV-301 Pediatric Expansion Study Group			
Suzanne	Klutch		MD	Preferred Primary Care Physicians	Pittsburgh, PA	Study team	2019nCoV-301 Pediatric Expansion Study Group			
Jessica	Martier		Dr	Preferred Primary Care Physicians	Pittsburgh, PA	Study team	2019nCoV-301 Pediatric Expansion Study Group			
Marilou	Cruz		MD	Premier Health Research Center	Downey, CA	Principal investigator	2019nCoV-301 Pediatric Expansion Study Group			
Hoang-Chuing	Vu		MD	Premier Health Research Center	Downey, CA	Study team	2019nCoV-301 Pediatric Expansion Study Group			
Janet	Serrano			Premier Health Research Center	Downey, CA	Study team	2019nCoV-301 Pediatric Expansion Study Group			
Valerie	Martinez			Premier Health Research Center	Downey, CA	Study team	2019nCoV-301 Pediatric Expansion Study Group			
Amiel	Guevarra			Premier Health Research Center	Downey, CA	Study team	2019nCoV-301 Pediatric Expansion Study Group			
Marc	Cruz			Premier Health Research Center	Downey, CA	Study team	2019nCoV-301 Pediatric Expansion Study Group			
Teresa S.	Sligh		MD	Providence Clinical Research	North Hollywood, CA	Principal investigator	2019nCoV-301 Pediatric Expansion Study Group			
Parul	Desai		NP	Providence Clinical Research	North Hollywood, CA	Study team	2019nCoV-301 Pediatric Expansion Study Group			
Vincent	Huynh		BSc	Providence Clinical Research	North Hollywood, CA	Study team	2019nCoV-301 Pediatric Expansion Study Group			
Carlos	Lopez		MD	Providence Clinical Research	North Hollywood, CA	Study team	2019nCoV-301 Pediatric Expansion Study Group			
Erika	Mendoza		BA	Providence Clinical Research	North Hollywood, CA	Study team	2019nCoV-301 Pediatric Expansion Study Group			

*First name, last name, and suffix (if applicable) are required and will appear in PubMed.

***First Name and Middle Initial(s)**	***Last Name**	***Suffix (eg, Jr, III)**	Academic Degrees	Institution	Location (city, state/province, country)	Role or Contribution, eg, chair, principal investigator	Group (if more than 1 Group listed in the byline) and/or Subgroup (eg, Steering Committee)
Waseem	Chughtai		BS, MBBS	Research Your Health	Plano, TX	Study team	2019nCoV-301 Pediatric Expansion Study Group
Anuja	Sathe		MS	Research Your Health	Plano, TX	Study team	2019nCoV-301 Pediatric Expansion Study Group
Pamela	Fox			Research Your Health	Plano, TX	Study team	2019nCoV-301 Pediatric Expansion Study Group
Matthew G.	Davis		MD	Rochester Clinical Research	Rochester, NY	Principal investigator	2019nCoV-301 Pediatric Expansion Study Group
Jennifer	Foley			Rochester Clinical Research	Rochester, NY	Study team	2019nCoV-301 Pediatric Expansion Study Group
Shelly	Kane		MD	Rochester Clinical Research	Rochester, NY	Study team	2019nCoV-301 Pediatric Expansion Study Group
Cassidy	Glod			Rochester Clinical Research	Rochester, NY	Study team	2019nCoV-301 Pediatric Expansion Study Group
Marissa	Wuilliez			Rochester Clinical Research	Rochester, NY	Study team	2019nCoV-301 Pediatric Expansion Study Group
Abigail	Purcell			Rochester Clinical Research	Rochester, NY	Study team	2019nCoV-301 Pediatric Expansion Study Group
Shelly	Senders		MD	Senders Pediatrics	Cleveland, OH	Principal investigator	2019nCoV-301 Pediatric Expansion Study Group
Ashley	Jeffrey			Senders Pediatrics	Cleveland, OH	Study team	2019nCoV-301 Pediatric Expansion Study Group
Christopher	Fackelmann			Senders Pediatrics	Cleveland, OH	Study team	2019nCoV-301 Pediatric Expansion Study Group
Nicholas	Jezerinc			Senders Pediatrics	Cleveland, OH	Study team	2019nCoV-301 Pediatric Expansion Study Group
Caitlin	Fillioe			Senders Pediatrics	Cleveland, OH	Study team	2019nCoV-301 Pediatric Expansion Study Group
Timothy	Hudec			Senders Pediatrics	Cleveland, OH	Study team	2019nCoV-301 Pediatric Expansion Study Group
Charles	Fogarty		MD	Spartanburg Medical Research	Spartanburg, SC	Principal investigator	2019nCoV-301 Pediatric Expansion Study Group
Nicole	Crockford		MD	Spartanburg Medical Research	Spartanburg, SC	Study team	2019nCoV-301 Pediatric Expansion Study Group
Jami	Jones			Spartanburg Medical Research	Spartanburg, SC	Study team	2019nCoV-301 Pediatric Expansion Study Group
Connie	Mccauley			Spartanburg Medical Research	Spartanburg, SC	Study team	2019nCoV-301 Pediatric Expansion Study Group
Sherry	Yeisley			Spartanburg Medical Research	Spartanburg, SC	Study team	2019nCoV-301 Pediatric Expansion Study Group
Alison	Fogarty			Spartanburg Medical Research	Spartanburg, SC	Study team	2019nCoV-301 Pediatric Expansion Study Group
Douglas	Logan		MD	Sterling Research Group	Cincinnati, OH	Principal investigator	2019nCoV-301 Pediatric Expansion Study Group
Bruce C.	Gebhardt		MD	Sterling Research Group	Cincinnati, OH	Study team	2019nCoV-301 Pediatric Expansion Study Group
Padma N.	Mangu		MD	Sterling Research Group	Cincinnati, OH	Study team	2019nCoV-301 Pediatric Expansion Study Group
Debra	Beck Schroeck		MS, PA-C	Sterling Research Group	Cincinnati, OH	Study team	2019nCoV-301 Pediatric Expansion Study Group
Rajesh	Kumar Davit		MD	Sterling Research Group	Cincinnati, OH	Study team	2019nCoV-301 Pediatric Expansion Study Group
Gayle D.	Hennekes		PA-C, MPAS	Sterling Research Group	Cincinnati, OH	Study team	2019nCoV-301 Pediatric Expansion Study Group
Larkin Tyler	Wadsworth	III	MD	Sundance Clinical Research	St. Louis, MO	Principal investigator	2019nCoV-301 Pediatric Expansion Study Group
Horacio	Marafioti		MD	Sundance Clinical Research	St. Louis, MO	Study team	2019nCoV-301 Pediatric Expansion Study Group
Lyly	Dang		DNP-BC	Sundance Clinical Research	St. Louis, MO	Study team	2019nCoV-301 Pediatric Expansion Study Group
Lauren	Clement		NP-C	Sundance Clinical Research	St. Louis, MO	Study team	2019nCoV-301 Pediatric Expansion Study Group
Jennifer	Berry		FNP-BC	Sundance Clinical Research	St. Louis, MO	Study team	2019nCoV-301 Pediatric Expansion Study Group
Adebayo	Akinsola		MD	Tekton Research	Chamblee, GA	Principal investigator	2019nCoV-301 Pediatric Expansion Study Group
Gabriela	Baetista		MD	Tekton Research	Chamblee, GA	Study team	2019nCoV-301 Pediatric Expansion Study Group
Baba	Arimah			Tekton Research	Chamblee, GA	Study team	2019nCoV-301 Pediatric Expansion Study Group
Sheree	Dover			Tekton Research	Chamblee, GA	Study team	2019nCoV-301 Pediatric Expansion Study Group
Leslie	Hernandez			Tekton Research	Chamblee, GA	Study team	2019nCoV-301 Pediatric Expansion Study Group
Susan	Herrera			Tekton Research	Chamblee, GA	Study team	2019nCoV-301 Pediatric Expansion Study Group
Kenneth	Etokhana		MD	Tekton Research	San Antonio, TX	Principal investigator	2019nCoV-301 Pediatric Expansion Study Group
Nathaniel	De La Cruz			Tekton Research	San Antonio, TX	Study team	2019nCoV-301 Pediatric Expansion Study Group
Xavier	Fajardo			Tekton Research	San Antonio, TX	Study team	2019nCoV-301 Pediatric Expansion Study Group
Veronica	Galaviz			Tekton Research	San Antonio, TX	Study team	2019nCoV-301 Pediatric Expansion Study Group
Meranda	Ruiz			Tekton Research	San Antonio, TX	Study team	2019nCoV-301 Pediatric Expansion Study Group

Supplemental Online Content: Nonauthor Collaborators
*First name, last name, and suffix (if applicable) are required and will appear in PubMed.

***First Name and Middle Initial(s)**	***Last Name**	***Suffix (eg, Jr, III)**	Academic Degrees	Institution	Location (city, state/province, country)	Role or Contribution, eg, chair, principal investigator	Group (if more than 1 Group listed in the byline) and/or Subgroup (eg, Steering Committee)			
Tony	Diaz			Tekton Research	San Antonio, TX	Study team	2019nCoV-301 Pediatric Expansion Study Group			
Veronica	Garcia-Fragoso		MD	Texas Center for Drug Development	Houston, TX	Principal investigator	2019nCoV-301 Pediatric Expansion Study Group			
Maria Gabriela	Becerra		MD	Texas Center for Drug Development	Houston, TX	Study team	2019nCoV-301 Pediatric Expansion Study Group			
Cecilia	Mckeown			Texas Center for Drug Development	Houston, TX	Study team	2019nCoV-301 Pediatric Expansion Study Group			
Lisa	Holloway			Texas Center for Drug Development	Houston, TX	Study team	2019nCoV-301 Pediatric Expansion Study Group			
Stacey	Montero			Texas Center for Drug Development	Houston, TX	Study team	2019nCoV-301 Pediatric Expansion Study Group			
Tracy	Kowalsi			Texas Center for Drug Development	Houston, TX	Study team	2019nCoV-301 Pediatric Expansion Study Group			
Stuart H.	Cohen		MD	University of California Davis Health/NIAID (UM1AI068614)	Sacramento, CA	Principal investigator	2019nCoV-301 Pediatric Expansion Study Group			
Monica	Ruiz			University of California Davis Health/NIAID (UM1AI068614)	Sacramento, CA	Study team	2019nCoV-301 Pediatric Expansion Study Group			
Dean M.	Boswell		BS	University of California Davis Health/NIAID (UM1AI068614)	Sacramento, CA	Study team	2019nCoV-301 Pediatric Expansion Study Group			
Elizabeth E.	Robison		BS	University of California Davis Health/NIAID (UM1AI068614)	Sacramento, CA	Study team	2019nCoV-301 Pediatric Expansion Study Group			
Trina L.	Reynolds		BS	University of California Davis Health/NIAID (UM1AI068614)	Sacramento, CA	Study team	2019nCoV-301 Pediatric Expansion Study Group			
Sonja	Neumeister		MPH	University of California Davis Health/NIAID (UM1AI068614)	Sacramento, CA	Study team	2019nCoV-301 Pediatric Expansion Study Group			
Thomas B.	Campbell		MD	University of Colorado Hospital CRS/NIAID (UM1AI068636)/NCATS (UL1TR002535, UM1AI069432)	Aurora, CO	Principal investigator	2019nCoV-301 Pediatric Expansion Study Group			
Suzanne	Fiorillo		MSPH	University of Colorado Hospital CRS/NIAID (UM1AI068636)/NCATS (UL1TR002535, UM1AI069432)	Aurora, CO	Study team	2019nCoV-301 Pediatric Expansion Study Group			
Rebecca	Pitotti		RNP	University of Colorado Hospital CRS/NIAID (UM1AI068636)/NCATS (UL1TR002535, UM1AI069432)	Aurora, CO	Study team	2019nCoV-301 Pediatric Expansion Study Group			
Victoria	Riedel Anderson		MS	University of Colorado Hospital CRS/NIAID (UM1AI068636)/NCATS (UL1TR002535, UM1AI069432)	Aurora, CO	Study team	2019nCoV-301 Pediatric Expansion Study Group			
Jose	Castillo Mancilla		MD	University of Colorado Hospital CRS/NIAID (UM1AI068636)/NCATS (UL1TR002535, UM1AI069432)	Aurora, CO	Study team	2019nCoV-301 Pediatric Expansion Study Group			
Nga	Le		PharmD	University of Colorado Hospital CRS/NIAID (UM1AI068636)/NCATS (UL1TR002535, UM1AI069432)	Aurora, CO	Study team	2019nCoV-301 Pediatric Expansion Study Group			

*First name, last name, and suffix (if applicable) are required and will appear in PubMed.

***First Name and Middle Initial(s)**	***Last Name**	***Suffix (eg, Jr, III)**	Academic Degrees	Institution	Location (city, state/province, country)	Role or Contribution, eg, chair, principal investigator	Group (if more than 1 Group listed in the byline) and/or Subgroup (eg, Steering Committee)			
Milagritos	Tapia		MD	University of Maryland School of Medicine/NIAID (UM1AI148689)	Baltimore, MD	Study team	2019nCoV-301 Pediatric Expansion Study Group			
Kathleen	Neuzil		MD	University of Maryland School of Medicine/NIAID (UM1AI148689)	Baltimore, MD	Study team	2019nCoV-301 Pediatric Expansion Study Group			
Andrea	Berry		MD	University of Maryland School of Medicine/NIAID (UM1AI148689)	Baltimore, MD	Study team	2019nCoV-301 Pediatric Expansion Study Group			
E. Adrienne	Hammershaimb		MD, MS	University of Maryland School of Medicine/NIAID (UM1AI148689)	Baltimore, MD	Study team	2019nCoV-301 Pediatric Expansion Study Group			
Rosa	MacBryde		RN	University of Maryland School of Medicine/NIAID (UM1AI148689)	Baltimore, MD	Study team	2019nCoV-301 Pediatric Expansion Study Group			
Diana F	Florescu		MD	University of Nebraska Medical Center/NIAID (UM1AI068614)	Omaha, NE	Study team	2019nCoV-301 Pediatric Expansion Study Group			
Richard	Starlin		MD	University of Nebraska Medical Center/NIAID (UM1AI068614)	Omaha, NE	Study team	2019nCoV-301 Pediatric Expansion Study Group			
David	Kline		MD	University of Nebraska Medical Center/NIAID (UM1AI068614)	Omaha, NE	Study team	2019nCoV-301 Pediatric Expansion Study Group			
Andrea	Zimmer		MD	University of Nebraska Medical Center/NIAID (UM1AI068614)	Omaha, NE	Study team	2019nCoV-301 Pediatric Expansion Study Group			
Anum	Abbas		MD	University of Nebraska Medical Center/NIAID (UM1AI068614)	Omaha, NE	Study team	2019nCoV-301 Pediatric Expansion Study Group			
Natasha	Wilson		APRN	University of Nebraska Medical Center/NIAID (UM1AI068614)	Omaha, NE	Study team	2019nCoV-301 Pediatric Expansion Study Group			
Erin	Hoffman			University of North Carolina/NIAID (UM1AI068619)/University of North Carolina at Chapel Hill Center for AIDS Research (P30AI050410)/NC TraCS Institute (UL1TR002489)	Chapel Hill, NC	Study team	2019nCoV-301 Pediatric Expansion Study Group			
Carolina	Pastrana Medina			University of North Carolina/NIAID (UM1AI068619)/University of North Carolina at Chapel Hill Center for AIDS Research (P30AI050410)/NC TraCS Institute (UL1TR002489)	Chapel Hill, NC	Study team	2019nCoV-301 Pediatric Expansion Study Group			
Susan	Pedersen			University of North Carolina/NIAID (UM1AI068619)/University of North Carolina at Chapel Hill Center for AIDS Research (P30AI050410)/NC TraCS Institute (UL1TR002489)	Chapel Hill, NC	Study team	2019nCoV-301 Pediatric Expansion Study Group			

Supplemental Online Content: Nonauthor Collaborators
*First name, last name, and suffix (if applicable) are required and will appear in PubMed.

***First Name and Middle Initial(s)**	***Last Name**	***Suffix (eg, Jr, III)**	Academic Degrees	Institution	Location (city, state/province, country)	Role or Contribution, eg, chair, principal investigator	Group (if more than 1 Group listed in the byline) and/or Subgroup (eg, Steering Committee)			
Mandy	Tipton			University of North Carolina/NIAID (UM1AI068619)/University of North Carolina at Chapel Hill Center for AIDS Research (P30AI050410)/NC TraCS Institute (UL1TR002489)	Chapel Hill, NC	Study team	2019nCoV-301 Pediatric Expansion Study Group			
Alison	Burbank		MD	University of North Carolina/NIAID (UM1AI068619)/University of North Carolina at Chapel Hill Center for AIDS Research (P30AI050410)/NC TraCS Institute (UL1TR002489)	Chapel Hill, NC	Study team	2019nCoV-301 Pediatric Expansion Study Group			
Michelle	Hernandez		MD	University of North Carolina/NIAID (UM1AI068619)/University of North Carolina at Chapel Hill Center for AIDS Research (P30AI050410)/NC TraCS Institute (UL1TR002489)	Chapel Hill, NC	Study team	2019nCoV-301 Pediatric Expansion Study Group			
Peyton	Thompson		MD	University of North Carolina/NIAID (UM1AI068619)/University of North Carolina at Chapel Hill Center for AIDS Research (P30AI050410)/NC TraCS Institute (UL1TR002489)	Chapel Hill, NC	Study team	2019nCoV-301 Pediatric Expansion Study Group			
Zachary	Willis		MD	University of North Carolina/NIAID (UM1AI068619)/University of North Carolina at Chapel Hill Center for AIDS Research (P30AI050410)/NC TraCS Institute (UL1TR002489)	Chapel Hill, NC	Study team	2019nCoV-301 Pediatric Expansion Study Group			
Joseph	Eron		MD	University of North Carolina/NIAID (UM1AI068619)/University of North Carolina at Chapel Hill Center for AIDS Research (P30AI050410)/NC TraCS Institute (UL1TR002489)	Chapel Hill, NC	Study team	2019nCoV-301 Pediatric Expansion Study Group			
Robert W.	Coombs		MD, PhD	University of Washington	Seattle, WA	Study team	2019nCoV-301 Pediatric Expansion Study Group			
Erin A.	Goecker		MS	University of Washington	Seattle, WA	Study team	2019nCoV-301 Pediatric Expansion Study Group			
Yunda	Huang		PhD	University of Washington	Seattle, WA	Study team	2019nCoV-301 Pediatric Expansion Study Group			
Youyi	Fong		PhD	University of Washington	Seattle, WA	Study team	2019nCoV-301 Pediatric Expansion Study Group			
Robert J.	Buynak		MD	Velocity Clinical Research	Valparaiso, IN	Principal investigator	2019nCoV-301 Pediatric Expansion Study Group			
Angella	Webb		APRN	Velocity Clinical Research	Valparaiso, IN	Study team	2019nCoV-301 Pediatric Expansion Study Group			
Rena	Rivas			Velocity Clinical Research	Valparaiso, IN	Study team	2019nCoV-301 Pediatric Expansion Study Group			

*First name, last name, and suffix (if applicable) are required and will appear in PubMed.

***First Name and Middle Initial(s)**	***Last Name**	***Suffix (eg, Jr, III)**	Academic Degrees	Institution	Location (city, state/province, country)	Role or Contribution, eg, chair, principal investigator	Group (if more than 1 Group listed in the byline) and/or Subgroup (eg, Steering Committee)			
Stephanie	Andree		FNP	Velocity Clinical Research	Valparaiso, IN	Study team	2019nCoV-301 Pediatric Expansion Study Group			
Rachel	McNeal			Velocity Clinical Research	Valparaiso, IN	Study team	2019nCoV-301 Pediatric Expansion Study Group			
Megan	Smith		MD	Velocity Clinical Research	Valparaiso, IN	Study team	2019nCoV-301 Pediatric Expansion Study Group			
Julie	Kasarjian		MD	Velocity Clinical Research	Banning, CA	Principal investigator	2019nCoV-301 Pediatric Expansion Study Group			
Judith	Kirstein		MD	Velocity Clinical Research	Banning, CA	Study team	2019nCoV-301 Pediatric Expansion Study Group			
Krista	Foster			Velocity Clinical Research	Banning, CA	Study team	2019nCoV-301 Pediatric Expansion Study Group			
Nicole	Abels			Velocity Clinical Research	Banning, CA	Study team	2019nCoV-301 Pediatric Expansion Study Group			
Brandy	Lopez			Velocity Clinical Research	Banning, CA	Study team	2019nCoV-301 Pediatric Expansion Study Group			
Crystle	Rajania		Dr	Velocity Clinical Research	Banning, CA	Study team	2019nCoV-301 Pediatric Expansion Study Group			
Margaret	Rhee		MD	Velocity Clinical Research	Cleveland, OH	Principal investigator	2019nCoV-301 Pediatric Expansion Study Group			
Gabrielle	Jones			Velocity Clinical Research	Cleveland, OH	Study team	2019nCoV-301 Pediatric Expansion Study Group			
Alanna	Billups			Velocity Clinical Research	Cleveland, OH	Study team	2019nCoV-301 Pediatric Expansion Study Group			
Jane	Boggan			Velocity Clinical Research	Cleveland, OH	Study team	2019nCoV-301 Pediatric Expansion Study Group			
Denise	Roadman		PAC	Velocity Clinical Research	Cleveland, OH	Study team	2019nCoV-301 Pediatric Expansion Study Group			
Celeste	Blazy			Velocity Clinical Research	Cleveland, OH	Study team	2019nCoV-301 Pediatric Expansion Study Group			
Marian E.	Shaw		MD	Velocity Clinical Research	Meridian, ID	Principal investigator	2019nCoV-301 Pediatric Expansion Study Group			
Mark A.	Turner		MD	Velocity Clinical Research	Meridian, ID	Study team	2019nCoV-301 Pediatric Expansion Study Group			
Cory J.	Huffine		FNP-C	Velocity Clinical Research	Meridian, ID	Study team	2019nCoV-301 Pediatric Expansion Study Group			
Esther S.	Huffine		FNP-C	Velocity Clinical Research	Meridian, ID	Study team	2019nCoV-301 Pediatric Expansion Study Group			
Raymond	Coon			Velocity Clinical Research	Meridian, ID	Study team	2019nCoV-301 Pediatric Expansion Study Group			
Jacqueline	Hanson			Velocity Clinical Research	Meridian, ID	Study team	2019nCoV-301 Pediatric Expansion Study Group			
Michael	Waters		MD	Velocity Clinical Research	Chula Vista, CA	Principal investigator	2019nCoV-301 Pediatric Expansion Study Group			
Karla	Zepeda		NP	Velocity Clinical Research	Chula Vista, CA	Study team	2019nCoV-301 Pediatric Expansion Study Group			
Scott	Overcash		MD	Velocity Clinical Research	Chula Vista, CA	Study team	2019nCoV-301 Pediatric Expansion Study Group			
Jordan	Coslet		NP	Velocity Clinical Research	Chula Vista, CA	Study team	2019nCoV-301 Pediatric Expansion Study Group			
Dalia	Tovar		MA	Velocity Clinical Research	Chula Vista, CA	Study team	2019nCoV-301 Pediatric Expansion Study Group			
Kia	Lee			Velocity Clinical Research	Chula Vista, CA	Study team	2019nCoV-301 Pediatric Expansion Study Group			
Mark	Koch		MD	Ventavia Research Group	Fort Worth, TX	Principal investigator	2019nCoV-301 Pediatric Expansion Study Group			
Norma	Escamilla		MD	Ventavia Research Group	Fort Worth, TX	Study team	2019nCoV-301 Pediatric Expansion Study Group			
Lydia	Luna		MD	Ventavia Research Group	Fort Worth, TX	Study team	2019nCoV-301 Pediatric Expansion Study Group			
Erin	Mcleod			Ventavia Research Group	Fort Worth, TX	Study team	2019nCoV-301 Pediatric Expansion Study Group			
Kathryn	Dykes			Ventavia Research Group	Fort Worth, TX	Study team	2019nCoV-301 Pediatric Expansion Study Group			
Julie A.	Ake		MD, MSc	Walter Reed Army Institute of Research	Silver Spring, MD	Study team	2019nCoV-301 Pediatric Expansion Study Group			
Michael	Husseman		MD	Wee Care Pediatrics	Layton, UT	Principal investigator	2019nCoV-301 Pediatric Expansion Study Group			
Janes	Fennell		MD	Wee Care Pediatrics	Layton, UT	Study team	2019nCoV-301 Pediatric Expansion Study Group			
Jennifer	Gilsoul		MD	Wee Care Pediatrics	Layton, UT	Study team	2019nCoV-301 Pediatric Expansion Study Group			
Robert	Hoki		MD	Wee Care Pediatrics	Layton, UT	Study team	2019nCoV-301 Pediatric Expansion Study Group			
Ashley	MacDonald			Wee Care Pediatrics	Layton, UT	Study team	2019nCoV-301 Pediatric Expansion Study Group			
Mia	Lobato			Wee Care Pediatrics	Layton, UT	Study team	2019nCoV-301 Pediatric Expansion Study Group			
Peter	Silas		MD	Wee Care Pediatrics	Syracuse, UT	Principal investigator	2019nCoV-301 Pediatric Expansion Study Group			
Cody	Hawkes		MD	Wee Care Pediatrics	Syracuse, UT	Study team	2019nCoV-301 Pediatric Expansion Study Group			
Jennifer	Cooper			Wee Care Pediatrics	Syracuse, UT	Study team	2019nCoV-301 Pediatric Expansion Study Group			

Supplemental Online Content: Nonauthor Collaborators
*First name, last name, and suffix (if applicable) are required and will appear in PubMed.

***First Name and Middle Initial(s)**	***Last Name**	***Suffix (eg, Jr, III)**	Academic Degrees	Institution	Location (city, state/province, country)	Role or Contribution, eg, chair, principal investigator	Group (if more than 1 Group listed in the byline) and/or Subgroup (eg, Steering Committee)			
Jennifer	Nelson		MD	Wee Care Pediatrics	Syracuse, UT	Study team	2019nCoV-301 Pediatric Expansion Study Group			
Jerica	Twitchell		RN	Wee Care Pediatrics	Syracuse, UT	Study team	2019nCoV-301 Pediatric Expansion Study Group			
Kenneth	Steil		MD	Foothills Research Center	Phoenix, AZ	Principal investigator	2019nCoV-301 Pediatric Expansion Study Group			
Mildred	DeJesus		MD	Foothills Research Center	Phoenix, AZ	Study team	2019nCoV-301 Pediatric Expansion Study Group			
LaShonda	Gilbert		MD	Foothills Research Center	Phoenix, AZ	Study team	2019nCoV-301 Pediatric Expansion Study Group			
Carey	Goldsmith		MD	Foothills Research Center	Phoenix, AZ	Study team	2019nCoV-301 Pediatric Expansion Study Group			
Maria	Gustilo		MD	Foothills Research Center	Phoenix, AZ	Study team	2019nCoV-301 Pediatric Expansion Study Group			
Jaimi	Jones		Dr	Foothills Research Center	Phoenix, AZ	Study team	2019nCoV-301 Pediatric Expansion Study Group			
Mark	McKenzie		MD	WR Clinsearch	Chattanooga, TN	Principal investigator	2019nCoV-301 Pediatric Expansion Study Group			
Teresa	Deese			WR Clinsearch	Chattanooga, TN	Study team	2019nCoV-301 Pediatric Expansion Study Group			
Mitsi	Earwood			WR Clinsearch	Chattanooga, TN	Study team	2019nCoV-301 Pediatric Expansion Study Group			
Vickie	Leathers			WR Clinsearch	Chattanooga, TN	Study team	2019nCoV-301 Pediatric Expansion Study Group			
Diane	Sproles			WR Clinsearch	Chattanooga, TN	Study team	2019nCoV-301 Pediatric Expansion Study Group			
Obadias	Marques		Dr	WR Clinsearch	Chattanooga, TN	Study team	2019nCoV-301 Pediatric Expansion Study Group			
