# Supplementary material for: Safety, Immunogenicity, and Efficacy of the NVX-CoV2373 COVID-19 Vaccine in Adolescents: A Randomized Clinical Trial
Source: JAMA Netw Open. 2023 Apr 26;6(4):e239135. doi: 10.1001/jamanetworkopen.2023.9135 (PMC10536880; doi:10.1001/jamanetworkopen.2023.9135)
Supplement: Supplement 4. — Data Sharing Statement [file jamanetwopen-e239135-s004.pdf]

Data Sharing Statement

Áñez. Safety, Immunogenicity, and Efficacy of the NVX-CoV2373 COVID-19 Vaccine in Adolescents. *JAMA Netw Open*. Published April 26, 2023. doi:10.1001/jamanetworkopen.2023.9135

Data
Data available: No
