# Supplementary material for: Safety, Immunogenicity, and Efficacy of the NVX-CoV2373 COVID-19 Vaccine in Adolescents
Source: JAMA Netw Open. Author manuscript; Available in PMC 2023 Sep 28. (PMC10536880; doi:10.1001/jamanetworkopen.2023.9135)
Supplement: Supplementary file 1 [file NIHMS1927823-supplement-Supplement_1_Supplementary_Online_Content.pdf]

Supplementary Online Content

Áñez G, Dunkle LM, Gay CL, et al; 2019nCoV-301−Pediatric Expansion Study Group. Safety, immunogenicity, and efficacy of the NVX-CoV2373 COVID-19 vaccine in adolescents: a randomized clinical trial. *JAMA Netw Open*. 2023;6(4):e239135. doi:10.1001/jamanetworkopen.2023.9135

eAppendix. Data and Safety Monitoring Board (DSMB) Members List

eMethods. Data Accrual and Analysis

eFigure 1. Circulating Variant Strains of SARS-CoV-2 During Study Conduct

eTable 1. Primary and Secondary Objectives and End Points Addressed in This Manuscript, Protocol Version 8.0

eTable 2. End Point Definitions of COVID-19 Severity

eTable 3. Symptoms Suggestive of COVID-19

eTable 4. Baseline Demographic Characteristics of the Per-Protocol Efficacy Analysis Set

eTable 5. Baseline Demographic Characteristics of the Per-Protocol Immunogenicity Analysis Set

eTable 6. Duration (Days) of Safety Follow-up Post Dose 2 Precrossover (Safety Analysis Set)

eTable 7. Duration (Days) of Solicited Local Adverse Events Within 7 Days After Dose 1 and Dose 2 in All Participants (Safety Analysis Set)

eTable 8. Summary of Solicited Local Adverse Events Within 7 Days After Dose 1 and Dose 2 in All Participants (Safety Analysis Set)

eTable 9. Duration (Days) of Solicited Systemic Adverse Events Within 7 Days After Dose 1 and Dose 2 in All Participants (Safety Analysis Set)

eTable 10. Summary of Solicited Systemic Adverse Events Within 7 Days After Dose 1 and Dose 2 by Age Group (Safety Analysis Set)

eFigure 2. Solicited Local and Systemic Adverse Events by Age Subgroup

eTable 11. Overall Summary of Treatment-Emergent Adverse Events Reported Between Start of First Vaccination and Blinded Crossover or Early Withdrawal (Safety Analysis Set)

eTable 12. Overall Summary of Unsolicited Adverse Events by System Organ Class and Preferred Term Reported Within 49 Days After First Vaccination in at Least 0.5% of All Adolescent Participants in Any Study Vaccine Group by Age Strata (Safety Analysis Set)

eTable 13. Overall Summary of Unsolicited Serious Adverse Events From Start of First Vaccination to Blinded Crossover Dose in All Adolescent Participants in Any Study Vaccine Group by Age Strata (Safety Analysis Set)

eFigure 3. Box Plot of Neutralizing Antibody Titers for SARS-CoV-2 Wild-Type Virus at Specified Time Points in Baseline Serologically Negative/PCR-Negative Adolescent Participants

eFigure 4. Box Plot of Serum IgG Antibody Concentrations to SARS-CoV-2 S Protein in Baseline Serologically Negative/PCR-Negative Adolescent Participants 12 to <18 Years of Age (PP-IMM Analysis Set)

© 2023 Áñez G et al. *JAMA Network Open.*

eFigure 5. Box Plot of hACE2 Inhibition Antibodies to SARS-CoV-2 S Protein in Baseline Serologically Negative/PCR-Negative Adolescent Participants 12 to <18 Years of Age (PP-IMM Analysis Set)

eFigure 6. Serum IgG Antibody Concentrations to SARS-CoV-2 S Protein From Different Variants in Baseline Serologically Negative/PCR-Negative Adolescent Participants 12 to <18 Years of Age

eFigure 7. hACE2 Inhibition Antibodies to SARS-CoV-2 S Protein From Different Variants in Baseline Serologically Negative/PCR-Negative Adolescent Participants 12 to <18 Years of Age

eTable 14. Vaccine Efficacy Against RT-PCR–Confirmed Symptomatic Mild, Moderate, or Severe COVID-19 From First Injection Due to Any SARS-CoV-2 Variant in Adolescent Participants (Full Analysis Set)

eTable 15. Duration of Surveillance Time (Days) for Primary Efficacy End Point (Per-Protocol Efficacy Analysis Set)

eTable 16. Vaccine Efficacy Against RT-PCR–Confirmed Symptomatic Mild, Moderate, or Severe COVID-19 at Least 7 Days After Second Vaccination Due to Any SARS-CoV-2 Variant in Adolescent Participants Not Previously Exposed to SARS-CoV-2 (PP-EFF Analysis Set)

eTable 17. Vaccine Efficacy Against RT-PCR–Confirmed Symptomatic Mild, Moderate, or Severe COVID-19 at Least 7 Days After Second Vaccination Due to the SARS-CoV-2 Delta Variant in Adolescent Participants Not Previously Exposed to SARS-CoV-2 (PP-EFF Analysis Set)

eReferences.

This supplementary material has been provided by the authors to give readers additional information about their work.

© 2023 Áñez G et al. *JAMA Network Open*.

eAppendix. Data and Safety Monitoring Board (DSMB) Members List

**Member**	**Institution**
Richard J. Whitley, MD (Chair)	School of Medicine, University of Alabama at Birmingham
Abdel Babiker, PhD	MRC Clinical Trials Unit at University College, London
Lisa A. Cooper, MD, MPH	Johns Hopkins University School of Medicine and Bloomberg School of Public Health
Susan S. Ellenberg, PhD	University of Pennsylvania School of Medicine, Center for Clinical Epidemiology and Biostatistics, Division of Biostatistics
Alan Fix, MD, MS	Vaccine Development Global Program, Center for Vaccine Innovation and Access, PATH
Marie Griffin, MD, MPH	Vanderbilt University Medical Center
Steven Joffe, MD, MPH	University of Pennsylvania, Perelman School of Medicine
Jorge Kalil, MD, MPH	Heart Institute, Hospital das Clínicas da Faculdade de Medicina da Universidade de São Paulo, Brazil
Myron M. Levine, MD, DTPH	University of Maryland School of Medicine
Malegapuru W. Makgoba, MB, ChB, DPhil, FRCP	University of KwaZulu-Natal, South Africa
Reneé H. Moore, PhD	Drexel University
Anastasios A. Tsiatis, PhD	North Carolina State University
Sally Hunsberger, PhD (Executive Secretary)	NIAID, NIH

© 2023 Áñez G et al. *JAMA Network Open*.

eMethods. Data Accrual and Analysis

Safety Assessments

Following collection of sufficient safety data to support application for Emergency Use Authorization (EUA), ie, median 2 months' duration of safety follow-up, participants were offered administration of two injections of the alternate study material 21 days apart ("blinded crossover"). That is, initial recipients of placebo did receive SARS-CoV-2 rS with Matrix-M™ adjuvant and initial recipients of SARS-CoV-2 rS with Matrix-M™ adjuvant did receive placebo. The same procedure used for the initial set of vaccinations was followed at the time of the blinded crossover to ensure that the integrity of the blinded study was maintained.

Solicited AEs of reactogenicity after the initial series of vaccinations was collected via participants' parents/representative reporting in the eDiary utilizing a smartphone application. All participants' parents/representative were trained on the use of these applications, and smartphone devices were provided for those parents/representatives who needed them at the initiation of their participation in the study (Day 0).

Safety assessments included collection of participant-recorded solicited (local and systemic reactogenicity) events through 7 days following each injection in the initial set of vaccinations collected via eDiary. Unsolicited AEs and MAAEs were collected through 49 days, ie, 28 days after second injection of the initial and crossover sets of vaccinations. MAAEs attributed to vaccine, AESIs, SAEs, will be collected through EoS.

Safety Monitoring

Safety was monitored routinely by the Sponsor physicians. A centralized Data and Safety Monitoring Board (DSMB) was established in collaboration with NIH, NIAID, Biomedical Advanced Research and Development Authority (BARDA), and Novavax according to the charter dictated by the participating groups. This group reviewed interim unblinded data periodically throughout the period of blinded follow-up and made recommendations with respect to safety.

Prospective Surveillance of COVID-19

For prospective surveillance, participants' parents were requested to notify clinical sites as soon as fever or other specified symptoms were experienced by the participant or during the scheduled weekly remote contacts with the site. Sites then scheduled an in-person Acute Illness Visit for medical evaluation (to include oxygen [O_2] saturation and respiratory rate) and medically attended nasal swab in symptomatic participants. Active surveillance for COVID-19 continued after the blinded crossover through the first 12 months of study. Passive surveillance of safety and efficacy via remote contacts or the scheduled visits continues during Months 12 to 24.

Study participants whose medically attended nasal swabs from the Acute Illness Visit were confirmed at the central laboratory to be RT-PCR-positive for SARS-CoV-2 were contacted by the study site to arrange a Convalescent Visit. The Convalescent Visit was to occur approximately 1 month (or as soon thereafter, as feasible) after the onset of the RT-PCR–confirmed case of COVID-19 to assess status of AEs, record the clinical course of the disease on the End Point Form, and obtain a blood sample for convalescent serologic testing.

COVID-19 Endpoint Assessment

To ensure the quality and accuracy of investigator-recorded endpoint assessments collected on the Endpoint Assessment electronic case report form (eCRF) page, programmatic checking was performed prior to the data extraction for analysis. The algorithms and the data sources to be used for programming were determined and documented prior to unblinding. Using data elements relevant to the endpoint definition and captured in the eCRF, programmatic determination of potential endpoints and associated start date and severity were performed. Data elements used included RT-PCR results by the central laboratory from the swab and pulse oximeter readings collected at the Acute Illness Visit. Disease episodes were constructed programmatically, including date of initial symptoms, date of positive RT-PCR test result, and preliminary severity based on symptoms reported and pulse oximeter readings. The programmatically determined endpoints were compared with the data collected on the Endpoint Assessment eCRF. Discrepancies such as missing or difference in date of start of illness or difference in

© 2023 Áñez G et al. *JAMA Network Open*.

severity rating prompted Data Management to issue queries to the investigators. The Endpoint Assessment eCRF data, along with the official study RT-PCR results from the University of Washington, Seattle, WA, were used for analysis of the efficacy endpoints.

Potentially severe cases of symptomatic RT-PCR–positive COVID-19 were reviewed by an external Independent Endpoint Review Committee (ERC) established by the sponsor. The ERC consisted of physicians with clinical and research experience (eg, medical review and/or clinical trial experience) in infectious diseases. The committee's structure, responsibilities, and operation were specified within a charter. Potentially severe cases included COVID-19 reported as SAEs, programmatically identified endpoints consisting of at least 1 pulse oximeter reading ≤93%, and episodes identified as severe on the Endpoint Assessment eCRF. Participant profiles (as outlined in the charter) were provided to the committee members for review according to the process outlined in the charter. These participant profiles included demographics, medical history, AEs, concomitant medications, and the Endpoint Assessment eCRF. The external reviewers documented the criteria used for their clinical review of the cases.

The results of the review were to confirm the case as severe or rule that the case was not severe. The outcome of the review for each case was stored in an electronic medical review system. A file was exported from the system and provided to the Biostatistics group for use in analysis. Cases that were ruled as not severe by the committee despite an investigator-entered severe grading were further reviewed and documented by Novavax clinician(s) prior to unblinding to determine the severity to use in analysis. Cases that were ruled as severe by the ERC but not severe by the investigator were analyzed as severe in the analysis.

Nasal Swabs for Viral Detection

Swabs of the anterior nares were obtained at the study site on Day 0 (prior to study vaccination), at the Acute Illness Visit, and at the first crossover vaccination visit.

Quantitative RT-PCR was performed on RT-PCR–positive swabs using the Abbott RealTime RT-PCR to assess viral load and sequencing of viral genetic material detected in nasal swab RT-PCR testing to evaluate viral mutations.

SARS-CoV-2 RT-PCR Testing

The RT-PCR test being used is the Abbott RealTime SARS-CoV-2 Assay, which was granted EUA by the US Food and Drug Administration (FDA) on March 18, 2020 (https://www.fda.gov/media/136255/download). The testing was performed at the University of Washington. The validation and verification of the Abbott RealTime SARS-CoV-2 Assay analytical and clinical performance has been published.[1] Dry swabs were used and have been validated for this assay with storage at 2-8°C for up to 7 days and then frozen at −80°C. Once the sample is received at the University of Washington, the dry swabs are eluted into Roswell Park Memorial Institute (RPMI) +2% fetal bovine serum (FBS) or phosphate buffered saline (PBS) prior to testing. The University of Washington conducted an analysis validating and verifying the performance of the Abbott RealTime SARS-CoV-2 Assay with varying viral dilutions. The outcome of the analysis was published elsewhere.[2]

Whole-Genome Sequencing (WGS) and Clade/Lineage Assignment

Secondary endpoints of the trial included the first episode of RT-PCR–positive, symptomatic COVID-19 due to strain shown by gene sequencing to represent a variant not considered as a variant of concern (VOC) or variant of interest (VOI), as well as those due to VOC/VOI, according to the Centers for Disease Control and Prevention (CDC) SARS-CoV-2 Variant Classifications and Definitions,[3] starting at least 7 days postvaccination 2 in the initial vaccination period. However, due to the SARS-CoV-2 epidemiology at time of initiation of the Pediatric Expansion of PREVENT-19, there were no cases reported due to variants antigenically comparable to the prototype strain. In fact, all sequences from COVID-19 cases were identified as the Delta variant.

Baseline RT-PCR–positive samples as well as RT-PCR positive samples from the Acute Illness Visit with enough viral load were sent to the University of Washington Virology Laboratory for WGS, using methodology described elsewhere.[4,5] In case of multiple RT-PCR-positive samples for a given symptomatic episode, that with the highest viral load was chosen for sequencing.

Sequencing analysis included viral clade/lineage assignment using both the Nextstrain clade label system (https://nextstrain.org/blog/2021-01-06-updated-SARS-CoV-2-clade-naming) and the PANGO lineages designation system (https://cov-lineages.org/), and identification as a VOC/VOI/High

© 2023 Áñez G et al. *JAMA Network Open*.

Consequence, per the CDC (https://www.cdc.gov/coronavirus/2019-ncov/variants/variant-info.html). Evaluation of SARS-CoV-2 infection or disease was available by site and/or geographic region. The classification of variants was conducted by the University of Washington Virology Laboratory and provided for analysis.

Anti-S IgG Antibody Titer Determination (ELISA) [Fit-for-Purpose Assay] (eFigure 6)
The quantitation of anti-spike (S) protein immunoglobulin G (IgG) by enzyme-linked immunosorbent assay (ELISA) (EC_{50}) against recently emerging variants was conducted in the Novavax Vaccine Immunology Laboratory to determine cross-reactivity in post hoc testing. This procedure was fit-for-purpose and differs minimally from the fully validated version of the assay performed by Novavax Clinical Immunology Laboratory (GCLP) for the SARS-CoV-2 Wuhan prototype strain that was used for per protocol testing. The validated assay has been already described elsewhere.[6] The validated method uses a reference standard to measure the total IgG in the serum samples to ensure the consistent measurement and the IgG is reported in ELISA units (EU/mL). Both methods are technically similar, except for the final readout and differences in regulated environment.

For the fit-for-purpose assay, 96-well microtiter plates (Thermo Fisher Scientific, Rochester, NY, USA) were coated with 1.0 µg/mL of SARS-CoV-2 rS protein from Wuhan-Hu-1, Alpha, Beta, Delta, Delta Plus, Gamma, Mu, and Omicron BA.1. Plates were washed with PBS-T and nonspecific binding was blocked with TBS Startblock blocking buffer (Thermo Fisher Scientific). Serum samples were serially diluted 3-fold down starting with a 1:300 dilution (ie, 10^{-2} to 10^{-8}) and added to the coated plates followed by incubation at room temperature for 2 hours. Following incubation, plates were washed with PBS-T and HRP-conjugated goat anti-human IgG (Southern Biotech, Birmingham, AL, USA) was added for 1 hour. Plates were washed with PBS-T and 3,3',5,5'-tetramethylbenzidine (TMB) peroxidase substrate (Sigma, St Louis, MO, USA) was added. Reactions were stopped with TMB stop solution (ScyTek Laboratories, Inc. Logan, UT). Plates were read at optical density (OD) 450 nm with a SpectraMax Plus plate reader (Molecular Devices, Sunnyvale, CA, USA). EC_{50} titer values were calculated by 4-parameter fitting using SoftMax Pro 6.5.1 GxP software. Individual subject anti-S IgG titers, group geometric mean titers, and 95% confidence intervals (CIs) were plotted using GraphPad Prism 7.05 software. For serum titers below the assay lower limit of detection (LOD), a titer of <300 (starting dilution) was reported and a value of "150" assigned to the sample to calculate the group mean titer.

hACE2 Receptor Inhibiting Antibody Titer Determination (ELISA) [Fit-for-Purpose Assay] (eFigure 7)
Human ACE2 receptor blocking antibody ELISA against newer variants was performed in the Novavax Vaccine Immunology Laboratories as post hoc testing. This procedure was fit-for-purpose and differs minimally technically from the fully validated version of the SARS-CoV-2 Wuhan prototype strain assay performed by Novavax Clinical Immunology Laboratory. The validated assay has been already described elsewhere.[7]

For the fit-for-purpose assay, ninety-six-well plates were coated with 1.0 µg/mL SARS-CoV-2 rS protein from the Wuhan-Hu-1, Alpha, Beta, Delta, Delta Plus, Gamma, Mu, and Omicron BA.1 variants, overnight at 4°C. Plates were washed with PBS-T, and nonspecific binding was blocked with TBS Startblock blocking buffer (Thermo Fisher Scientific). Sera were serially diluted 2-fold starting with a 1:20 dilution and added to coated wells for 1 hour at room temperature. After washing, 30 ng/mL of histidine-tagged hACE2 (Sino Biologicals, Beijing, CN) was added to wells for 1 hour at room temperature. HRP-conjugated anti-histidine IgG was added and incubated for 1 hour followed by addition of TMB substrate. Plates were read at OD 450 nm with a SpectraMax Plus plate reader (Molecular Devices, Sunnyvale, CA, USA) and data analyzed with SoftMax Pro software. The % Inhibition for each dilution for each sample was calculated using the following equation in the SoftMax Pro program: 100-[(MeanResults/ControlValue@PositiveControl)*100].

Serum dilution vs %Inhibition plot was generated, and curve fitting was done by 4 parameter logistic (4PL) curve fitting to data. Serum antibody titer at 50% binding inhibition (IC_{50}) of hACE2 to SARS-CoV-2 rS protein was determined in the SoftMax Pro program. Individual subject hACE2 receptor inhibiting titers, group geometric mean titers, and 95% CI were plotted using GraphPad Prism 7.05 software. For a titer below the assay LOD, a titer of <20 (starting dilution) was reported and a value of "10" assigned to the sample to calculate the group mean titer.

© 2023 Áñez G et al. *JAMA Network Open*.

Analysis Populations

There were 6 main analysis sets used in this trial:

- The **Intent-to-Treat (ITT) Analysis Set** included all participants who were randomized, regardless of protocol violations or missing data. The ITT analysis set was used for participant disposition summaries and were analyzed according to the treatment arm to which the participant was randomized.
- The **Full Analysis Set (FAS)** included all participants who were randomized and received at least one dose of study vaccine/placebo, regardless of protocol violations or missing data. Participants who were unblinded with an intention to receive other COVID-19 vaccines were censored at the time of unblinding. The FAS population was analyzed according to the treatment group to which participants were randomized. The FAS were used for supportive analyses. When the efficacy endpoints were analyzed using FAS, baseline SARS-CoV-2 seropositivity or nasal swab RT-PCR-positivity was ignored.
- The **Safety Analysis Set** included all randomized participants who received at least one dose of study vaccine/placebo. Participants in the Safety Analysis Set were analyzed according to the treatment actually received. In cases where information is available that indicated that a participant received both active vaccine and placebo during the initial vaccination series, the participant was analyzed as part of the active group.
- The **Per-Protocol Efficacy (PP-EFF) Analysis Set** included all participants who received the full prescribed regimen of trial vaccine and had no major protocol deviations that occurred before the first COVID-19 positive episode (ie, participant was censored at the time of the protocol deviation) and were determined to affect the efficacy outcomes, including baseline SARS-CoV-2 seropositivity or nasal swab RT-PCR-positivity. Participants who were unblinded with an intention to receive other COVID-19 vaccines were censored at the time of unblinding. Although the study enrolled participants regardless of SARS-CoV-2 serologic status at the time of initial vaccination, any participants with confirmed infection or prior infection due to SARS-CoV-2 at baseline, by nasal swab RT-PCR or serology (assessed by anti-nucleocapsid [anti-NP]), were excluded from the PP-EFF population. PP-EFF was the primary set for all efficacy endpoints.
- A second **PP-EFF (PP-EFF-2) Analysis Set** was defined to allow evaluation of baseline serostatus analysis' impact on vaccine efficacy (VE). The PP-EFF-2 Analysis Set followed the same method described in the PP-EFF population, with the exception that it included all participants regardless of baseline serostatus (anti-NP serology) or baseline virological status (RT-PCR).
- The Day 35 **Per-Protocol Immunogenicity (PP-IMM) Analysis Set** included all participants who had at least a baseline and Day 35 sample result available after vaccination and had no major protocol deviations that occurred before the Day 35 visit (ie, participant was censored at the time of the protocol deviation) that were determined to affect the effectiveness outcomes, including baseline SARS-CoV-2 seropositivity or nasal swab RT-PCR positivity. Participants who were unblinded with an intention to receive other COVID-19 vaccines were censored at the time of unblinding. Although the study enrolled participants regardless of SARS-CoV-2 serologic status at the time of initial vaccination, any participants with confirmed infection or prior infection due to SARSCoV-2 at baseline, by nasal swab RT-PCR or serology (assessed by anti-NP), were excluded from the PP-IMM population. Participants must have received the second vaccination to be included in the PP-IMM analysis set. PP-IMM was the primary set for all effectiveness endpoints.

Statistical Method for Efficacy Endpoints

The VE is defined as VE (%) = (1 − RR) × 100, where RR = relative risk of incidence rates between the two trial vaccine groups (SARS-CoV-2 rS / placebo). The RR was estimated by exponentiating the treatment group coefficient from a Poisson regression analysis with robust error variance.[8] To assess incidence rates rather than absolute counts of cases, accounting for differences in follow-up times starting at 7 days after the second vaccination among participants, an offset was utilized in the Poisson regression. The generalized linear model with unstructured correlation matrix (robust error variances) was used. The explanatory variables in the model included the trial vaccine group. The dependent variable was the incidence rate of the endpoint of interest. The robust error variances were estimated using repeated statement and the participant identifier. To account for the censoring in the analysis, the offset

© 2023 Áñez G et al. *JAMA Network Open*.

was defined as the natural log of the time from the start of follow-up (7 days post second vaccination) to the outcome of interest or to the end of study in addition to censoring described in the analysis set definitions. Poisson distribution was used with a logarithmic link function. In the case where there were zero endpoints for one of the vaccine groups or the total number of endpoints in both treatment groups combined is less than 5, a Poisson model was substituted with an exact conditional binomial method using the Clopper-Pearson method. This method conditions on the total number of events across the treatment groups where the number of events in the active group are generated from a single binomial distribution. The point estimate from this single binomial distribution and the corresponding confidence intervals constructed using the Clopper-Pearson method were transformed back to relative risks. A Cox proportional hazards model was also developed as a supportive analysis to the Poisson regression. The model followed the same explanatory and dependent variables as the Poisson model and censored participants based on their follow-up time available.

Statistical Method for Effectiveness

For the effectiveness endpoint (noninferiority of the neutralizing antibody response compared with young adults from the PREVENT-19 trial, 18 to <26 years), successful demonstration of noninferiority requires meeting the following 3 prespecified criteria simultaneously:

1. Lower bound of 2-sided 95% CI for the ratio of geometric mean titers (GMTs) ($GMT_{12\text{-}<18yo}/GMT_{18\text{-}<26yo}$) >0.67
2. Point estimate of the ratio of GMTs ≥0.82 (estimated as square root of 2/3)
3. Lower bound of the 2-sided 95% CI for difference of serologic response ($SR_{12\text{-}<18yo}/SR_{18\text{-}<26yo}$) >−10%

With 400 evaluable participants (500, accounting for 20% non evaluability) in the active vaccine group randomly selected from each of the 18 to <26 years of age subset of participants in the Adult Main Study and the Pediatric Expansion of PREVENT-19, there is over 85% power (through simulations) to demonstrate the first two noninferiority criteria when assuming an underlying GMT for the 18 to <26 years of age group up to 1.1-fold higher than the 12 to <18 years of age group. In the absence of an established correlate of protection for SARS-CoV-2 vaccines, serologic response was defined as ≥4-fold increase in neutralization titers (MN50) at Day 35 relative to baseline titers. With this definition and assumed SCR of 95% in the 18 to <26 years of age group, there is over 80% power to demonstrate the third noninferiority criterion for a difference as large as 4% lower in the 12 to <18 years of age group.

© 2023 Áñez G et al. *JAMA Network Open*.

Supplemental Tables and Figures

eFigure 1. Circulating Variant Strains of SARS-CoV-2 During Study Conduct

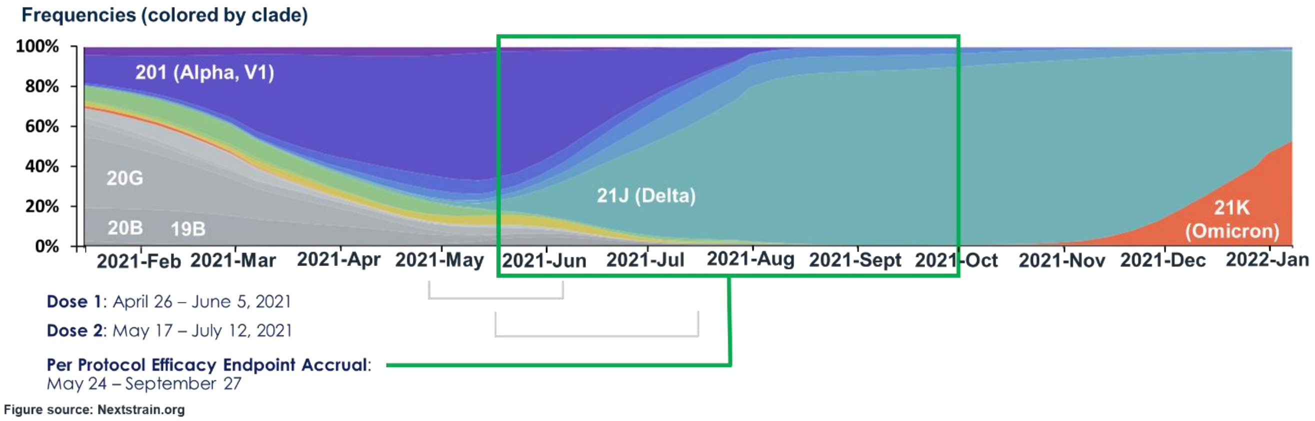

Circulating variant strains of SARS-CoV-2 reported during endpoint accrual (data source: Nextstrain.org).

© 2023 Áñez G et al. *JAMA Network Open*.

eTable 1. Primary and Secondary Objectives and End Points Addressed in This Manuscript, Protocol Version 8.0

<table>
<tr><th>Objectives</th><th>Endpoints</th></tr>
<tr><td>Primary:</td><td>Primary:</td></tr>
<tr><td>To evaluate the efficacy of a 2-dose regimen of SARS-CoV-2 rS adjuvanted with Matrix-M1 compared to placebo against PCR-confirmed symptomatic COVID-19 illness diagnosed ≥7 days after completion of the second injection in the initial set of vaccinations of adolescent participants 12 to <18 years of age.
To describe the safety experience for the vaccine vs placebo in adolescent participants (12 to <18 years of age) based on solicited short-term reactogenicity by toxicity grade for 7 days following each vaccination (Days 0 and 21) after the initial set of vaccinations.
To assess overall safety through 49 days (28 days after second injection of each set of vaccinations [initial and crossover]) by comparing vaccine vs placebo for all unsolicited AEs and MAAEs.
To assess the frequency and severity of MAAEs attributed to vaccine, AESIs, or SAEs through the EoS and to compare vaccine vs placebo after each set of vaccinations (initial and crossover).
To assess all-cause mortality in vaccine vs placebo recipients after each set of vaccinations (initial and crossover).
To assess noninferiority of the neutralizing antibody response for all adolescent participants seronegative to anti-SARS-CoV-2 NP antibodies at baseline, compared with that observed in seronegative adult participants 18 to 25 years of age from the Adult Main Study (Immunogenicity Population participants before crossover).</td><td>Primary Endpoints:
First episode of PCR-positive mild, moderate, or severe COVID-19, where severity is defined as:
Mild COVID-19 (≥1 of the following):
Fever (defined by subjective or objective measure, regardless of use of antipyretic medications)
New onset cough
≥2 additional COVID-19 symptoms:
New onset or worsening of shortness of breath or difficulty breathing compared to baseline.
New onset fatigue.
New onset generalized muscle or body aches.
New onset headache.
New loss of taste or smell.
Acute onset of sore throat, congestion or runny nose.
New onset nausea, vomiting or diarrhea.
OR Moderate COVID-19 (≥1 of the following):
High fever (≥38.4°C) for ≥3 days (regardless of use of antipyretic medications, need not be contiguous days).
Any evidence of significant LRTI:
Shortness of breath (or breathlessness or difficulty breathing) with or without exertion (greater than baseline).
Tachypnea: 24 to 29 breaths per minute at rest.
SpO_2: 94% to 95% on room air.
Abnormal chest X-ray or chest CT consistent with pneumonia or LRTI.
Adventitious sounds on lung auscultation (eg, crackles/rales, wheeze, rhonchi, pleural rub, stridor).
OR Severe COVID-19 (≥1 of the following):
Tachypnea: ≥30 breaths per minute at rest.
Resting heart rate ≥125 beats per minute.
SpO_2: ≤93% on room air or PaO_2/FiO_2 <300 mmHg.
High flow oxygen (O_2) therapy or NIV/NIPPV (eg, CPAP or BiPAP).
Mechanical ventilation or ECMO.
One or more major organ system dysfunction or failure to be defined by diagnostic testing/clinical syndrome/interventions, including any of the following:
Acute respiratory failure, including ARDS.
Acute renal failure.
Acute hepatic failure.
Acute right or left heart failure.
Septic or cardiogenic shock (with shock defined as SBP <90 mm Hg OR DBP <60 mm Hg).
Acute stroke (ischemic or hemorrhagic).</td></tr>
</table>

© 2023 Áñez G et al. *JAMA Network Open*.

Objectives	Endpoints
	○ Acute thrombotic event: AMI, DVT, PE.<br>○ Requirement for: vasopressors, systemic corticosteroids, or hemodialysis.<br>• MIS-C, as per the CDC definition:<br>○ An individual aged <21 years presenting with fever (>38.0°C for ≥24 hours, or report of subjective fever lasting ≥24 hours), laboratory evidence of inflammation (including, but not limited to, one or more of the following: an elevated C-reactive protein (CRP), erythrocyte sedimentation rate (ESR), fibrinogen, procalcitonin, d-dimer, ferritin, lactic acid dehydrogenase (LDH), or interleukin 6 (IL-6), elevated neutrophils, reduced lymphocytes and low albumin), and evidence of clinically severe illness requiring hospitalization, with multisystem (>2) organ involvement (cardiac, renal, respiratory, hematologic, gastrointestinal, dermatologic or neurological); AND<br>○ No alternative plausible diagnoses; AND<br>○ Positive for current or recent SARS-CoV-2 infection by RT-PCR, serology, or antigen test; or COVID-19 exposure within the 4 weeks prior to the onset of symptoms.<br>• Admission to an ICU.<br>• Death<br>Safety Endpoints:<br>• Reactogenicity incidence, duration and severity (mild, moderate or severe) recorded by parent(s)/caregiver(s) on their electronic patient-reported outcome diary application (eDiary) on days of vaccination and subsequent 6 days (total 7 days after each vaccine injection in the initial set of vaccinations).<br>○ Reactogenicity endpoints include injection site reactions:<br>▪ Pain.<br>▪ Tenderness.<br>▪ Erythema.<br>▪ Swelling/induration.<br>○ Systemic reactions:<br>▪ Fever.<br>▪ Malaise.<br>▪ Fatigue.<br>▪ Arthralgia.<br>▪ Myalgia.<br>▪ Headache.<br>▪ Nausea/vomiting.<br>• Incidence and severity of MAAEs through 49 days, ie, 28 days after second injection of each set of vaccinations (initial and crossover).<br>• Incidence and severity of unsolicited AEs through 49 days, ie, 28 days after second injection of each set of vaccinations (initial and crossover).

© 2023 Áñez G et al. *JAMA Network Open*.

Objectives	Endpoints
	• Incidence and severity of MAAEs attributed to study vaccine, SAEs and AESIs through Month 12. COVID-19 diagnoses will be included as "medically important" events that are reported as SAEs.<br>• Incidence and severity of SAEs (including COVID-19 diagnoses), MAAEs attributed to study vaccine and AESIs during Month 12 through Month 24 or the EoS.<br>• Death due to any cause.<br>Effectiveness Endpoint:<br>• Neutralizing antibody response at Day 35 for all adolescent participants seronegative to anti-SARS-CoV-2 NP antibodies at baseline, compared with that observed in seronegative adult participants 18 to 25 years of age from the Adult Main Study (Immunogenicity Population participants before crossover).
Secondary Objectives:	Secondary Endpoints:
• To evaluate the efficacy of a two-dose regimen of SARS-CoV-2 rS adjuvanted with Matrix-M™ compared to placebo against RT-PCR-confirmed moderate-to-severely symptomatic COVID-19 illness diagnosed ≥7 days after completion of the second vaccination in the initial set of vaccinations of adult participants ≥18 years of age.<br>• To assess VE against ANY symptomatic SARS-CoV-2 infection.<br>• To assess VE according to race and ethnicity.	• First episode of RT-PCR-positive moderate or severe COVID-19, as defined under the primary endpoint.<br>• ANY symptomatic SARS-CoV-2 infection, defined as: RT-PCR-positive nasal swab and ≥1 of any of the following symptoms:<br>○ Fever.<br>○ New onset cough.<br>○ New onset or worsening of shortness of breath or difficulty breathing compared to baseline.<br>○ New onset fatigue.<br>○ New onset generalized muscle or body aches.<br>○ New onset headache.<br>○ New loss of taste or smell.<br>○ Acute onset of sore throat, congestion, or runny nose.<br>○ New onset nausea, vomiting, or diarrhea.<br>• Description of course, treatment, and severity of COVID-19 reported after a RT-PCR-confirmed case via the Endpoint Form.

Abbreviations: AESI, adverse event of special interest; AMI, acute myocardial infarction; ARDS, acute respiratory distress syndrome; BiPAP, bilevel positive airway pressure; BMI, body mass index; CDC, Centers for Disease Control and Prevention; COVID-19, coronavirus disease 2019; CoVPN, COVID-19 Prevention Network; CPAP, continuous positive airway pressure; CT, computed tomography; DBP, diastolic blood pressure; DVT, deep vein thrombosis; ECMO, extracorporeal membrane oxygenation; eDiary, electronic patient-reported outcome diary; EoS, end of study; FiO_2, fraction of inspired oxygen; hACE2, human angiotensin-converting enzyme 2; ICU, intensive care unit; IgG, immunoglobulin G; IFN-γ, interferon gamma; IL, interleukin; LRTI, lower respiratory tract infection; MAAE, medically attended adverse event; MN, microneutralization; NIAID, National Institute of Allergy and Infectious Diseases; NIH, National Institutes of Health; NP, nucleocapsid; NIPPV, noninvasive positive pressure ventilation; NIV, noninvasive ventilation; O_2, oxygen; OWS, Operation Warp Speed; PaO_2, partial pressure of oxygen; PBMC, peripheral blood mononuclear cell; RT-PCR, reverse transcriptase-polymerase chain reaction; PE, pulmonary embolism; SAE, serious adverse event; SARS-CoV-2, severe acute respiratory syndrome coronavirus 2; SARS-CoV-2 rS, severe acute respiratory syndrome coronavirus 2 recombinant spike protein nanoparticle vaccine; SBP systolic blood pressure; SpO_2, oxygen saturation; Th1, type 1 T helper; Th2, type 2 T helper; TNF-α, tumor necrosis factor alpha; VE, vaccine efficacy.

© 2023 Áñez G et al. *JAMA Network Open*.

eTable 2. End Point Definitions of COVID-19 Severity

**COVID-19 Severity**	**Endpoint Definitions**
	**First episode of RT-PCR-positive mild, moderate, or severe COVID-19:**
Mild	≥1 of the following:<br>• Fever (defined by subjective or objective measure, regardless of use of antipyretic medications)<br>• New onset of cough<br>• ≥2 additional COVID-19 symptoms:<br>○ New onset or worsening of shortness of breath or difficulty breathing compared to baseline.<br>○ New onset of fatigue.<br>○ New onset of generalized muscle or body aches.<br>○ New onset of headache.<br>○ New loss of taste or smell.<br>○ Acute onset of sore throat, congestion, or runny nose.<br>○ New onset of nausea, vomiting, or diarrhea.
Moderate[a]	≥1 of the following:<br>• High fever (≥38.4°C) for ≥3 days (regardless of use of antipyretic medications, need not be contiguous days).<br>• Any evidence of significant LRTI:<br>○ Shortness of breath (or breathlessness or difficulty breathing) with or without exertion (greater than baseline).<br>○ Tachypnea: 24 to 29 breaths per minute at rest.<br>○ SpO_2: 94% to 95% on room air.<br>○ Abnormal chest X-ray or CT consistent with pneumonia or LRTI.<br>• Adventitious sounds on lung auscultation (eg, crackles/rales, wheeze, rhonchi, pleural rub, stridor).
Severe[a]	≥1 of the following:<br>• Tachypnea: ≥30 breaths per minute at rest.<br>• Resting heart rate ≥125 beats per minute.<br>• SpO_2: ≤93% on room air or PaO_2/FiO_2 <300 mmHg.<br>• High flow O_2 therapy or NIV/NIPPV (eg, CPAP or BiPAP).<br>• Mechanical ventilation or ECMO.<br>• One or more major organ system dysfunction or failure to be defined by diagnostic testing/clinical syndrome/interventions, including any of the following:<br>○ Acute respiratory failure, including ARDS.<br>○ Acute renal failure.<br>○ Acute hepatic failure.<br>○ Acute right or left heart failure.<br>○ Septic or cardiogenic shock (with shock defined as SBP <90 mm Hg OR DBP <60 mm Hg).<br>○ Acute stroke (ischemic or hemorrhagic).<br>○ Acute thrombotic event: AMI, DVT, PE.<br>○ Requirement for: vasopressors, systemic corticosteroids, or hemodialysis.<br>• MIS-C, as per the CDC definition:<br>○ An individual aged <21 years presenting with fever (>38.0°C for ≥24 hours, or report of subjective fever lasting ≥24 hours), laboratory evidence of inflammation (including, but not limited to, one or more of the following: an elevated CRP, ESR, fibrinogen, procalcitonin, d-dimer, ferritin, LDH, or IL-6, elevated neutrophils, reduced lymphocytes and low albumin), and evidence of clinically severe illness requiring hospitalization, with multisystem (>2) organ involvement (cardiac, renal, respiratory, hematologic, gastrointestinal, dermatologic or neurological); AND

© 2023 Áñez G et al. *JAMA Network Open*.

	○ No alternative plausible diagnoses; AND<br>○ Positive for current or recent SARS-CoV-2 infection by RT-PCR, serology, or antigen test; or COVID-19 exposure within the 4 weeks prior to the onset of symptoms.<br>• Admission to an ICU.<br>• Death.

Abbreviations: AMI, acute myocardial infarction; ARDS, acute respiratory distress syndrome; BiPAP, bi-level positive airway pressure; COVID-19, coronavirus disease 2019; CPAP, continuous positive air pressure; CRP, C-reactive protein; CT, computerized tomography; DBP, diastolic blood pressure; DVT, deep vein thrombosis; ECMO, extracorporeal membrane oxygenation; ESR, erythrocyte sedimentation rate; FiO_2, fraction of inspired oxygen; ICU, intensive care unit; IL-6, interleukin 6; LDH, lactic acid dehydrogenase; LRTI, lower respiratory tract infection; MIS-C, multisystem inflammatory syndrome in children; NIV, noninvasive ventilation; NIPPV, noninvasive positive pressure ventilation; PaO_2, partial pressure of oxygen in the alveolus; PE, pulmonary embolism; RT-PCR, reverse transcriptase-polymerase chain reaction; SBP, systolic blood pressure; SpO_2, oxygen saturation.

[a]Participants with a single vital sign abnormality placing them in the moderate or severe categories must also meet the criteria for mild COVID-19.

© 2023 Áñez G et al. *JAMA Network Open*.

eTable 3. Symptoms Suggestive of COVID-19

•	Fever (body temperature >38.0°C, in the absence of other symptoms) or chills
•	New onset or worsening of cough compared with baseline
•	New onset or worsening of shortness of breath or difficulty breathing over baseline
•	New onset of fatigue
•	New onset of generalized muscle or body aches
•	New onset of headache
•	New loss of taste or smell
•	Acute onset of sore throat
•	Acute onset of congestion or runny nose
•	New onset of nausea or vomiting
•	New onset of diarrhea

Abbreviations: COVID-19, coronavirus disease 2019.

© 2023 Áñez G et al. *JAMA Network Open*.

eTable 4. Baseline Demographic Characteristics of the Per-Protocol Efficacy Analysis Set

	**NVX-CoV2373 n = 1205**	**Placebo n = 594**	**Total n = 1799**
Age, y			
Mean (SD)	13.8 (1.4)	13.8 (1.4)	13.8 (1.4)
Median	14.0	14.0	14.0
Range	12–17	12–17	12–17
Age group, n (%)			
12 – < 15 y	822 (68.2)	407 (68.5)	1229 (68.3)
15 – < 18 y	383 (31.8)	187 (31.5)	570 (31.7)
Sex, n (%)			
Male	622 (51.6)	328 (55.2)	950 (52.8)
Female	583 (48.4)	266 (44.8)	849 (47.2)
Race or ethnic group, n (%)			
African American/Black	155 (12.9)	77 (13.0)	232 (12.9)
American Indian/Alaska Native	13 (1.1)	6 (1.0)	19 (1.1)
Asian	38 (3.2)	26 (4.4)	64 (3.6)
Multiple	67 (5.6)	33 (5.6)	100 (5.6)
Native Hawaiian or Other Pacific Islander	3 (0.2)	1 (0.2)	4 (0.2)
Not reported	7 (0.6)	4 (0.7)	11 (0.6)
White	922 (76.5)	447 (75.3)	1369 (76.1)
Ethnicity, n (%)			
Hispanic/Latino	185 (15.4)	100 (16.8)	285 (15.8)
Not Hispanic/Latino	1015 (84.2)	494 (83.2)	1509 (83.9)
Not reported	2 (0.2)	0 (0)	2 (0.1)
Unknown	3 (0.2)	0 (0)	3 (0.2)
BMI category, n (%)			
Underweight (<18.0 kg/m2)	35 (2.9)	26 (4.4)	61 (3.4)
Normal (18.0–24.9 kg/m2)	632 (52.4)	342 (57.6)	974 (54.1)
Overweight (25.0–29.9 kg/m2)	224 (18.6)	84 (14.1)	308 (17.1)
Obese (≥30.0 kg/m2)	314 (26.1)	142 (23.9)	456 (25.3)
Previous SARS-CoV-2 infection status, n (%)*			
Positive	0 (0)	0 (0)	0 (0)
Negative	1204 (99.9)	594 (100)	1798 (99.9)
Missing	1 (<0.1)	0	1 (<0.1)

Abbreviations: BMI, body mass index; max, maximum; min, minimum; NVX-CoV2373, 5 µg SARS-CoV-2 recombinant S with 50 µg Matrix-M™ adjuvant; RT-PCR, Reverse Transcriptase polymerase chain reaction; SARS-CoV-2 severe acute respiratory syndrome coronavirus 2; SD, standard deviation.
Percentages are based on per protocol analysis set within each treatment and overall.
* Participants with either anti-nucleoprotein or RT-PCR positive at baseline.
BMI was classified as follows (using gender and age specific percentiles): Underweight = subjects less than the 5th percentile; Healthy weight = subjects within the 5th percentile and up to the 85th percentile; Overweight = subjects within the 85th percentile to less than the 95th percentile; Obesity = subjects equal to or greater than the 95th percentile.

© 2023 Áñez G et al. *JAMA Network Open*.

eTable 5. Baseline Demographic Characteristics of the Per-Protocol Immunogenicity Analysis Set

	**NVX-CoV2373 n = 1120**	**Placebo n = 534**	**Total n = 1654**
Age, y			
Mean (SD)	13.8 (1.4)	13.7 (1.4)	13.8 (1.4)
Median	14.0	14.0	14.0
Range	12–17	12–17	12–17
Age group, n (%)			
12 – < 15 y	763 (68.1)	366 (68.5)	1129 (68.3)
15 – < 18 y	357 (31.9)	168 (31.5)	525 (31.7)
Sex, n (%)			
Male	579 (51.7)	295 (55.2)	874 (52.8)
Female	541 (48.3)	239 (44.8)	780 (47.2)
Race or ethnic group, n (%)			
African American/Black	139 (12.4)	67 (12.5)	206 (12.5)
American Indian/Alaska Native	13 (1.2)	6 (1.1)	19 (1.1)
Asian	34 (3.0)	23 (4.3)	57 (3.4)
Multiple	63 (5.6)	33 (6.2)	96 (5.8)
Native Hawaiian or Other Pacific Islander	3 (0.3)	1 (0.2)	4 (0.2)
Not reported	5 (0.4)	3 (0.6)	8 (0.5)
White	863 (77.1)	401 (75.1)	1264 (76.4)
Ethnicity, n (%)			
Hispanic/Latino	173 (15.4)	85 (15.9)	258 (15.6)
Not Hispanic/Latino	943 (84.2)	449 (84.1)	1392 (84.2)
Not reported	1 (<0.1)	0 (0)	1 (<0.1)
Unknown	3 (0.3)	0 (0)	3 (0.2)
BMI category, n (%)			
Underweight (<18.0 kg/m2)	31 (2.8)	23 (4.3)	54 (3.3)
Normal (18.0–24.9 kg/m2)	588 (52.5)	302 (56.6)	890 (53.8)
Overweight (25.0–29.9 kg/m2)	212 (18.9)	77 (14.4)	289 (17.5)
Obese (≥30.0 kg/m2)	289 (25.8)	132 (24.7)	421 (25.5)
Previous SARS-CoV-2 infection status, n (%)*			
Positive	0 (0)	0 (0)	0 (0)
Negative	1120 (100)	534 (100)	1654 (100)

Abbreviations: BMI, body mass index; max, maximum; min, minimum; NVX-CoV2373, 5 µg SARS-CoV-2 recombinant S with 50 µg Matrix-M™ adjuvant; RT-PCR, Reverse Transcriptase polymerase chain reaction; SARS-CoV-2 severe acute respiratory syndrome coronavirus 2; SD, standard deviation.
Percentages are based on per protocol immunogenicity analysis set within each treatment and overall.
* Participants with either anti-nucleoprotein or RT-PCR positive at baseline.
BMI was classified as follows (using gender and age specific percentiles): Underweight = subjects less than the 5th percentile; Healthy weight = subjects within the 5th percentile and up to the 85th percentile; Overweight = subjects within the 85th percentile to less than the 95th percentile; Obesity = subjects equal to or greater than the 95th percentile.

© 2023 Áñez G et al. *JAMA Network Open*.

eTable 6. Duration (Days) of Safety Follow-up Post Dose 2 Pre-Crossover (Safety Analysis Set)

	**NVX-CoV2373 N = 1468**	**Placebo N = 730**	**Total N = 2198**
Median	71	71	71
IQR	65–77	64–77	65–77
Min, Max	1–143	1–142	1–143

IQR, interquartile range; max, maximum; min, minimum.

© 2023 Áñez G et al. *JAMA Network Open*.

eTable 7. Duration (Days) of Solicited Local Adverse Events Within 7 Days After Dose 1 and Dose 2 in All Participants (Safety Analysis Set)

Solicited Local Adverse Events	NVX-CoV2373 N = 1487	Placebo N = 745
Pain (# of days ≥ grade 1), N1/N2	1448/1394	726/686
Dose 1, n	648	126
Median	2.0	1.0
IQR	1-3	1-1
Minimum-maximum	1-7	1-6
Dose 2, n	850	102
Median	2.0	1.0
IQR	1-3	1-1
Minimum-maximum	1-7	1-6
Tenderness (# of days ≥ grade 1), N1/N2	1448/1394	726/686
Dose 1, n	817	153
Median	2.0	1.0
IQR	1-3	1-2
Minimum-maximum	1-7	1-7
Dose 2, n	909	97
Median	2.0	1.0
IQR	1-3	1-2
Minimum-maximum	1-7	1-7
Erythema (# of days ≥ grade 1), N1/N2	1448/1394	726/686
Dose 1, n	15	5
Median	2.0	1.0
IQR	1-3	1-1
Minimum-maximum	1-4	1-1
Dose 2, n	104	0
Median	2.0	N/A
IQR	1-2.5	N/A
Minimum-maximum	1-6	N/A
Swelling (# of days ≥ grade 1), N1/N2	1448/1394	726/686
Dose 1, n	20	3
Median	1.0	1.0
IQR	1-2	1-2
Minimum-maximum	1-5	1-2
Dose 2, n	111	1
Median	2.0	1.0
IQR	1-2	1-1
Minimum-maximum	1-7	1-1

Abbreviations: IQR, interquartile range, n, number of participants who reported the solicited event; N, number of participants in the Safety Analysis Set following Dose 1/Dose 2; N1, number of participants in the Safety Analysis Set who received the first dose and completed at least 1 day of the reactogenicity diary; N2, number of participants in the Safety Analysis Set who received the second dose and completed at least 1 day of the reactogenicity diary; NVX-CoV2373, 5 *μg* SARS-CoV-2 rS with 50 *μg* Matrix-M™ adjuvant; SARS-CoV-2 rS, severe acute respiratory syndrome coronavirus 2 recombinant spike protein nanoparticle vaccine.

Note: Duration is calculated as the number of days the solicited event was greater than grade 0. n = number of subjects who reported the event.

© 2023 Áñez G et al. *JAMA Network Open*.

eTable 8. Summary of Solicited Local Adverse Events Within 7 Days After Dose 1 and Dose 2 in All Participants (Safety Analysis Set)

Solicited Local Adverse Events	All Participants	
	**NVX-CoV2373 N = 1487**	**Placebo N = 745**
Any local adverse event, N1/N2	1448/1394	726/686
Dose 1 (any grade)	948 (65.5%)	207 (28.5%)
Grade 3	22 (1.5%)	5 (0.7%)
Grade 4	0	0
Dose 2 (any grade)	1050 (75.3%)	141 (20.6%)
Grade 3	118 (8.5%)	4 (0.6%)
Grade 4	0	0
Any pain, N1/N2	1448/1394	726/686
Dose 1 (any grade)	648 (44.6%)	126 (17.4%)
Grade 3	10 (0.7%)	2 (0.3%)
Grade 4	0	0
Dose 2 (any grade)	850 (61.0%)	102 (14.9%)
Grade 3	38 (2.7%)	3 (0.4%)
Grade 4	0	0
Any tenderness, N1/N2	1448/1394	726/686
Dose 1 (any grade)	817 (56.4%)	153 (21.1%)
Grade 3	16 (1.1%)	2 (0.3%)
Grade 4	0	0
Dose 2 (any grade)	909 (65.2%)	97 (14.1%)
Grade 3	93 (6.7%)	1 (0.1%)
Grade 4	0	0
Any erythema, N1/N2	1448/1394	726/686
Dose 1 (any grade)	15 (1.0%)	5 (0.7%)
Grade 3	0	0
Grade 4	0	0
Dose 2 (any grade)	104 (7.5%)	0
Grade 3	10 (0.7%)	0
Grade 4	0	0
Any swelling, N1/N2	1448/1394	726/686
Dose 1 (any grade)	20 (1.4%)	3 (0.4%)
Grade 3	0	1 (0.1%)
Grade 4	0	0
Dose 2 (any grade)	111 (8.0%)	1 (0.1%)
Grade 3	8 (0.6%)	0
Grade 4	0	0

Abbreviations: FDA, US Food and Drug Administration; N, number of participants in the Safety Analysis Set following Dose 1/Dose 2; N1, number of participants in the Safety Analysis Set who received the first dose and completed at least 1 day of the reactogenicity diary; N2, number of participants in the Safety Analysis Set who received the second dose and completed at least 1 day of the reactogenicity diary; NVX-CoV2373, 5 *µg* SARS-CoV-2 rS with 50 *µg* Matrix-M™ adjuvant; SARS-CoV-2 rS, severe acute respiratory syndrome coronavirus 2 recombinant spike protein nanoparticle vaccine.
Note: Data are presented as number (%) of participants experiencing a solicited event. Percentages were based on n/N1 × 100 and n/N2 × 100. At each level of participant summarization, a participant was counted once if they indicated the event. Any grade pertains to reactions reported at grade ≥1. Grading of solicited adverse events was based on FDA Toxicity Grading Scale for Clinical Abnormalities.[10]

© 2023 Áñez G et al. *JAMA Network Open*.

eTable 9. Duration (Days) of Solicited Systemic Adverse Events Within 7 Days After hDose 1 and Dose 2 in All Participants (Safety Analysis Set)

**Solicited Systemic Adverse Events**	**NVX-CoV2373 N = 1487**	**Placebo N = 745**
Fatigue (# of days ≥ grade 1), N1/N2	1448/1394	726/686
Dose 1, n	350	113
Median	1.0	1.0
IQR	1-2	1-2
Minimum-maximum	1-7	1-6
Dose 2, n	695	100
Median	1.0	1.0
IQR	1-2	1-2
Minimum-maximum	1-7	1-6
Fever (# of days ≥ grade 1), N1/N2	1448/1394	726/686
Dose 1, n	11	5
Median	1.0	1.0
IQR	1-1	1-1
Minimum-maximum	1-3	1-2
Dose 2, n	235	1
Median	1.0	1.0
IQR	1-1	1-1
Minimum-maximum	1-2	1-1
Headache (# of days ≥ grade 1), N1/N2	1448/1394	726/686
Dose 1, n	440	181
Median	1.0	1.0
IQR	1-2	1-2
Minimum-maximum	1-7	1-7
Dose 2, n	793	119
Median	1.0	1.0
IQR	1-2	1-2
Minimum-maximum	1-7	1-6
Joint pain (# of days ≥ grade 1), N1/N2	1448/1394	726/686
Dose 1, n	102	35
Median	1.0	1.0
IQR	1-2	1-1
Minimum-maximum	1-6	1-5
Dose 2, n	225	21
Median	1.0	1.0
IQR	1-2	1-2
Minimum-maximum	1-6	1-5
Malaise (# of days ≥ grade 1), N1/N2	1448/1394	726/686
Dose 1, n	215	67
Median	1.0	1.0
IQR	1-2	1-2
Minimum-maximum	1-5	1-5
Dose 2, n	560	51
Median	1.0	1.0
IQR	1-2	1-2
Minimum-maximum	1-7	1-5

© 2023 Áñez G et al. *JAMA Network Open*.

Solicited Systemic Adverse Events	NVX-CoV2373<br>N = 1487	Placebo<br>N = 745
Muscle pain (# of days ≥ grade 1), N1/N2	1448/1394	726/686
Dose 1, n	492	114
Median	1.0	1.0
IQR	1-2	1-2
Minimum-maximum	1-7	1-7
Dose 2, n	683	82
Median	2.0	1.0
IQR	1-2	1-2
Minimum-maximum	1-6	1–-6
Nausea/Vomiting (# of days ≥ grade 1), N1/N2	1448/1394	726/686
Dose 1, n	113	56
Median	1.0	1.0
IQR	1-2	1-1
Minimum-maximum	1-5	1-4
Dose 2, n	277	33
Median	1.0	1.0
IQR	1-1	1-1
Minimum-maximum	1-7	1-6

Abbreviations: IQR, interquartile range, n, number of participants who reported the solicited event; N, number of participants in the Safety Analysis Set following Dose 1/Dose 2; N1, number of participants in the Safety Analysis Set who received the first dose and completed at least 1 day of the reactogenicity diary; N2, number of participants in the Safety Analysis Set who received the second dose and completed at least 1 day of the reactogenicity diary; NVX-CoV2373, 5 *μg* SARS-CoV-2 rS with 50 *μg* Matrix-M™ adjuvant; SARS-CoV-2 rS, severe acute respiratory syndrome coronavirus 2 recombinant spike protein nanoparticle vaccine.

Note: Duration is calculated as the number of days the solicited event was greater than grade 0. n = number of subjects who reported the event.

© 2023 Áñez G et al. *JAMA Network Open*.

eTable 10. Summary of Solicited Systemic Adverse Events Within 7 Days After Dose 1 and Dose 2 by Age Group (Safety Analysis Set)

Solicited Systemic Adverse Events	All Participants	
	**NVX-CoV2373 N= 1487**	**Placebo N = 745**
Any solicited systemic TEAE, N1/N2	1448/1394	726/686
Dose 1 (grade ≥1)	800 (55.2%)	296 (40.8%)
Grade 3	52 (3.6%)	25 (3.4%)
Grade 4	2 (0.1%)	0
Dose 2 (grade ≥1)	1038 (74.5%)	198 (28.9%)
Grade 3	304 (21.8%)	23 (3.4%)
Grade 4	2 (0.1%)	0
Headache, N1/N2	1448/1394	726/686
Dose 1 (grade ≥1)	440 (30.4%)	181 (24.9%)
Grade 3	13 (0.9%)	12 (1.7%)
Grade 4	0	0
Dose 2 (grade ≥1)	793 (56.9%)	119 (17.3%)
Grade 3	87 (6.2%)	14 (2.0%)
Grade 4	1(<0.1%)	0
Fatigue, N1/N2	1448/1394	726/686
Dose 1 (grade ≥1)	350 (24.2%)	113 (15.6%)
Grade 3	23 (1.6%)	9 (1.2%)
Grade 4	0	0
Dose 2 (grade ≥1)	695 (49.9%)	100 (14.6%)
Grade 3	185 (13.3%)	10 (1.5%)
Grade 4	0	0
Malaise, N1/N2	1448/1394	726/686
Dose 1 (grade ≥1)	215 (14.8%)	67 (9.2%)
Grade 3	16 (1.1%)	7 (1.0%)
Grade 4	0	0
Dose 2 (grade ≥1)	560 (40.2%)	51 (7.4%)
Grade 3	126 (9.0%)	4 (0.6%)
Grade 4	0	0
Muscle pain, N1/N2	1448/1394	726/686
Dose 1 (grade ≥1)	492 (34.0%)	114 (15.7%)
Grade 3	17 (1.2%)	4 (0.6%)
Grade 4	0	0
Dose 2 (grade ≥1)	683 (49.0%)	82 (12.0%)
Grade 3	104 (7.5%)	6 (0.9%)
Grade 4	0	0
Joint pain, N1/N2	1448/1394	726/686
Dose 1 (grade ≥1)	102 (7.0%)	35 (4.8%)
Grade 3	6 (0.4%)	1 (0.1%)
Grade 4	0	0
Dose 2 (grade ≥1)	226 (16.2%)	21 (3.1%)
Grade 3	40 (2.9%)	2 (0.3%)
Grade 4	0	0

© 2023 Áñez G et al. *JAMA Network Open*.

Solicited Systemic Adverse Events	All Participants	
	**NVX-CoV2373 N= 1487**	**Placebo N = 745**
Fever, N1/N2	1448/1394	726/686
Dose 1 (grade ≥1)	11 (0.8%)	5 (0.7%)
Grade 3	1 (<0.1%)	0
Grade 4	2 (0.1%)	0
Dose 2 (grade ≥1)	235 (16.9%)	1 (0.1%)
Grade 3	31 (2.2%)	0
Grade 4	0	0
Nausea/Vomiting, N1/N2	1448/1394	726/686
Dose 1 (grade ≥1)	113 (7.8%)	56 (7.7%)
Grade 3	2 (0.1%)	3 (0.4%)
Grade 4	0	0
Dose 2 (grade ≥1)	277 (19.9%)	33 (4.8%)
Grade 3	14 (1.0%)	3 (0.4%)
Grade 4	1 (<0.1%)	0

Abbreviations: FDA, US Food and Drug Administration; N number of participants in the Safety Analysis Set following Dose 1/Dose 2; N1 number of participants in the Safety Analysis Set who received the first dose and completed at least 1 day of the reactogenicity diary; N2 number of participants in the Safety Analysis Set who received the second dose and completed at least 1 day of the reactogenicity diary; NVX-CoV2373, 5 *μg* SARS-CoV-2 rS with 50 *μg* Matrix-M™ adjuvant; SARS-CoV-2 rS, severe acute respiratory syndrome coronavirus 2 recombinant spike protein nanoparticle vaccine; TEAE, treatment-emergent adverse event.

Note: Data are presented as number (%) of participants experiencing a solicited event. Percentages were based on n/N1 × 100 and n/N2 × 100. At each level of participant summarization, a participant was counted once if they indicated the event occurred and provided a severity during the reactogenicity period. The highest severity experienced during the reactogenicity period is summarized in this table.

Note: Grading of solicited adverse events was based on FDA Toxicity Grading Scale for Clinical Abnormalities.[10]

© 2023 Áñez G et al. *JAMA Network Open*.

eFigure 2. Solicited Local and Systemic Adverse Events by Age Subgroup

A

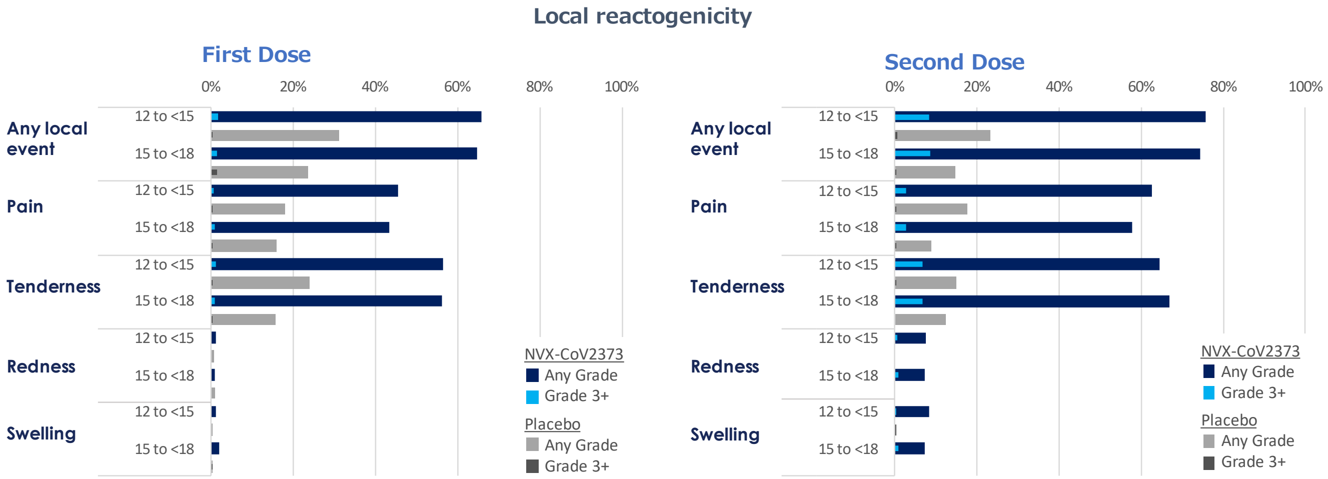

B

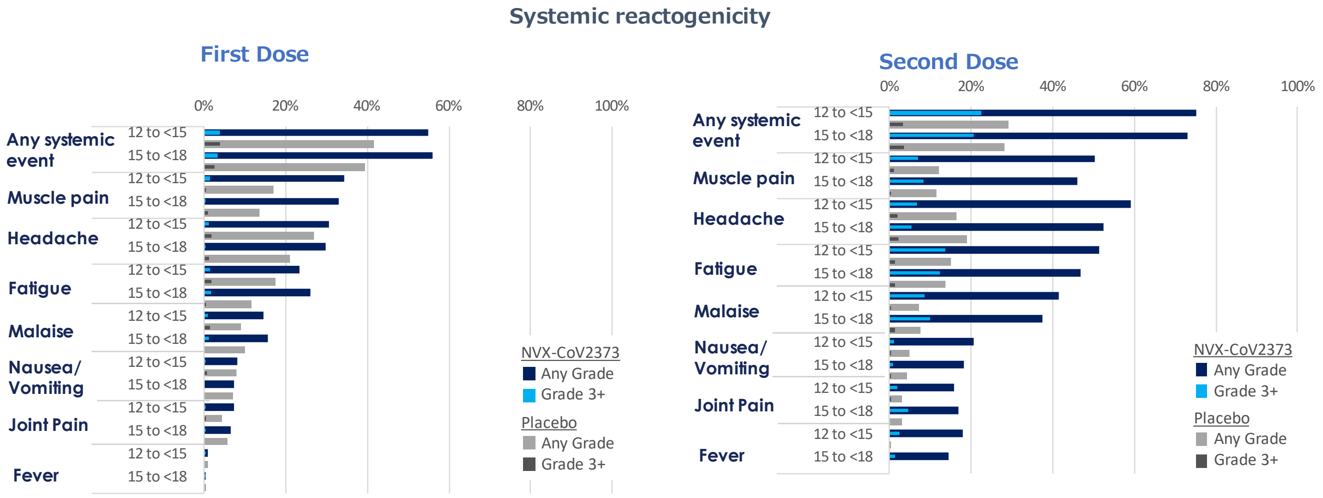

The percentage of participants vaccinated with NVX-CoV2373 in each age group with solicited local (A) and systemic (B) adverse events during the 7 days after each vaccination is plotted by FDA toxicity grade, either as any grade (mild, moderate, severe, or potentially life-threatening) or as Grade 3+ (severe or potentially life-threatening).[10]

© 2023 Áñez G et al. *JAMA Network Open*.

eTable 11. Overall Summary of Treatment-Emergent Adverse Events Reported Between Start of First Vaccination and Blinded Crossover or Early Withdrawal (Safety Analysis Set)

**TEAE Category**	**NVX-CoV2373 n = 1487**		**Placebo n = 745**	
	**n (%)**	**E**	**n (%)**	**E**
Any TEAE	236 (15.9)	386	116 (15.6)	178
Any severe TEAE[a]	6 (0.4)	7	2 (0.3)	2
Any treatment-related TEAE[a]	43 (2.9)	62	7 (0.9)	9
Any severe treatment-related TEAE[a]	0	0	0	0
Any MAAE	96 (6.5)	129	51 (6.8)	70
Any treatment-related MAAE[a]	5 (0.3)	10	3 (0.4)	4
Any serious treatment-related MAAE[a]	0	0	0	0
Any serious TEAE	7 (0.5)	9	2 (0.3)	2
Any TEAE leading to vaccination discontinuation	1 (<0.1)	1	1 (0.1)	1
Any treatment-related TEAE leading to vaccination discontinuation[a]	0	0	0	0
Any TEAE leading to study discontinuation	0	0	0	0
Any treatment-related TEAE leading to study discontinuation[a]	0	0	0	0
Any AESI: PIMMC	0	0	0	0
Any treatment-related AESI: PIMMC[a]	0	0	0	0
Any AESI: relevant to COVID-19	0	0	0	0
Any treatment-related AESI: relevant to COVID-19[a]	0	0	0	0

Abbreviations: AESI, adverse event of special interest; COVID-19, coronavirus disease 2019; E, number of events at each level of summarization; MAAE, medically attended adverse event; NVX-CoV2373, 5 *μg* SARS-CoV-2 rS with 50 *μg* Matrix-M™ adjuvant; PIMMC, potential immune-mediated medical conditions; SARS-CoV-2, severe acute respiratory syndrome coronavirus 2; SARS-CoV-2 rS, severe acute respiratory syndrome coronavirus 2 recombinant spike protein nanoparticle vaccine; TEAE, treatment-emergent adverse event.

[a] Relationship and severity were based on the data reported by site, ie, missing information was not imputed.

Note: Events mapped to solicited reactogenicity events were excluded from this presentation of unsolicited TEAEs.

Note: At each level of participant summarization, a participant was counted once if the participant reported ≥1 events.

© 2023 Áñez G et al. *JAMA Network Open*.

eTable 12. Overall Summary of Unsolicited Adverse Events by System Organ Class and Preferred Term Reported Within 49 Days After First Vaccination in at Least 0.5% of All Adolescent Participants in Any Study Vaccine Group by Age Strata (Safety Analysis Set)

**System Organ Class/ Preferred Term (MedDRA, Version 24.0)**	**Participants 12 to <18 Years**		**Participants 12 to <15 Years**		**Participants 15 to <18 Years**	
	**NVX-CoV2373 n = 1478**	**Placebo n = 745**	**NVX-CoV2373 n = 998**	**Placebo n = 500**	**NVX-CoV2373 n = 489**	**Placebo n = 245**
	**n (%)**	**n (%)**	**n (%)**	**n (%)**	**n (%)**	**n (%)**
Any system organ class	234 (15.7)	115 (15.4)	174 (17.4)	82 (16.4)	60 (12.3)	33 (13.5)
Infections and infestations	61 (4.1)	41 (5.5)	43 (4.3)	30 (6.0)	18 (3.7)	11 (4.5)
Upper respiratory tract infection	10 (0.7)	14 (1.9)	9 (0.9)	11 (2.2)	1 (0.2)	3 (1.2)
Viral infection	10 (0.7)	6 (0.8)	4 (0.4)	3 (0.6)	6 (1.2)	3 (1.2)
Nasopharyngitis	6 (0.4)	5 (0.7)	4 (0.4)	5 (1.0)	2 (0.4)	0
Respiratory, thoracic and mediastinal disorders	42 (2.8)	25 (3.4)	33 (3.3)	22 (4.4)	9 (1.8)	3 (1.2)
Nasal congestion	21 (1.4)	10 (1.3)	17 (1.7)	8 (1.6)	4 (0.8)	2 (0.8)
Cough	18 (1.2)	6 (0.8)	16 (1.6)	5 (1.0)	2 (0.4)	1 (0.4)
Oropharyngeal pain	16 (1.1)	13 (1.7)	12 (1.2)	10 (2.0)	4 (0.8)	3 (1.2)
Rhinorrhea	6 (0.4)	8 (1.1)	5 (0.5)	8 (1.6)	1 (0.2)	0
General disorders and administration site conditions	34 (2.3)	7 (0.9)	21 (2.1)	5 (1.0)	13 (2.7)	2 (0.8)
Fatigue	7 (0.5)	0	4 (0.4)	0	3 (0.6)	0
Injury, poisoning and procedural complications	38 (2.6)	15 (2.0)	27 (2.7)	8 (1.6)	11 (2.2)	7 (2.9)
Ligament sprain	3 (0.2)	3 (0.4)	3 (0.3)	1 (0.2)	0	2 (0.8)
Skin laceration	1 (<0.1)	5 (0.7)	1 (0.1)	4 (0.8)	0	1 (0.4)
Nervous system disorders	24 (1.6)	14 (1.9)	15 (1.5)	10 (2.0)	9 (1.8)	4 (1.6)
Headache	12 (0.8)	8 (1.1)	9 (0.9)	6 (1.2)	3 (0.6)	2 (0.8)
Gastrointestinal disorders	27 (1.8)	18 (2.4)	17 (1.7)	14 (2.8)	10 (2.0)	4 (1.6)
Nausea	8 (0.5)	5 (0.7)	4 (0.4)	5 (1.0)	4 (0.8)	0
Diarrhea	8 (0.5)	6 (0.8)	6 (0.6)	6 (1.2)	2 (0.4)	0
Vomiting	5 (0.3)	3 (0.4)	3 (0.3)	5 (1.0)	2 (0.4)	0

© 2023 Áñez G et al. *JAMA Network Open.*

System Organ Class/ Preferred Term (MedDRA, Version 24.0)	Participants 12 to <18 Years		Participants 12 to <15 Years		Participants 15 to <18 Years	
	NVX-CoV2373 n = 1478	Placebo n = 745	NVX-CoV2373 n = 998	Placebo n = 500	NVX-CoV2373 n = 489	Placebo n = 245
	n (%)	n (%)	n (%)	n (%)	n (%)	n (%)
Psychiatric disorders	22 (1.5)	9 (1.2)	15 (1.5)	6 (1.2)	7 (1.4)	3 (1.2)
Attention deficit hyperactivity disorder	8 (0.5)	2 (0.3)	5 (0.5)	2 (0.4)	3 (0.6)	0
Anxiety	2 (0.1)	2 (0.3)	1 (0.1)	0	1 (0.2)	2 (0.8)
Musculoskeletal and connective tissue disorders	18 (1.2)	2 (0.3)	14 (1.4)	0	4 (0.8)	2 (0.8)
Arthralgia	3 (0.2)	0	3 (0.3)	0	0	0
Skin and subcutaneous tissue disorders	21 (1.4)	6 (0.8)	17 (1.7)	5 (1.0)	4 (0.8)	1 (0.4)
Rash	7 (0.5)	2 (0.3)	6 (0.6)	2 (0.4)	1 (0.2)	0
Blood and lymphatic system disorders	11 (0.7)	0	9 (0.9)	0	2 (0.4)	0
Lymphadenopathy	10 (0.7)	0	8 (0.8)	0	2 (0.4)	0
Ear and labyrinth disorders	4 (0.3)	2 (0.3)	4 (0.4)	0	0	2 (0.8)
Eye disorders	7 (0.5)	1 (0.1)	6 (0.6)	0	1 (0.2)	1 (0.4)

Abbreviations: MedDRA, Medical Dictionary for Regulatory Activities; NVX-CoV2373, 5 *μg* SARS-CoV-2 rS with 50 *μg* Matrix-M™ adjuvant; SARS-CoV-2, severe acute respiratory syndrome coronavirus 2; SARS-CoV-2 rS, severe acute respiratory syndrome coronavirus 2 recombinant spike protein nanoparticle vaccine.

© 2023 Áñez G et al. *JAMA Network Open*.

eTable 13. Overall Summary of Unsolicited Serious Adverse Events From Start of First Vaccination to Blinded Crossover Dose in All Adolescent Participants in Any Study Vaccine Group by Age Strata (Safety Analysis Set)

System Organ Class/ Preferred Term (MedDRA, Version 24.0)	Participants 12 to <18 Years		Participants 12 to <15 Years		Participants 15 to <18 Years	
	NVX-CoV2373 n = 1487	Placebo n = 745	NVX-CoV2373 n = 998	Placebo n = 500	NVX-CoV2373 n = 489	Placebo n = 245
	n (%)	n (%)	n (%)	n (%)	n (%)	n (%)
Any system organ class	7 (0.5)	2 (0.3)	5 (0.5)	1 (0.2)	2 (0.4)	1 (0.4)
Injury, poisoning and procedural complications	3 (0.2)	0	2 (0.2)	0	1 (0.2)	0
Intentional overdose	2 (0.1)	0	1 (0.1)	0	1 (0.2)	0
Splenic rupture	1 (<0.1)	0	1 (0.1)	0	0	0
Infections and infestations	2 (0.1)	1 (0.1)	1 (0.1)	0	1 (0.2)	1 (0.4)
Gastroenteritis norovirus	1 (<0.1)	0	0	0	1 (0.2)	0
Localized infection	1 (<0.1)	0	1 (0.1)	0	0	0
Peritonsillar abscess	0	1 (0.1)	0	0	0	1 (0.4)
Nervous system disorders	2 (0.1)	0	1 (0.1)	0	1 (0.2)	0
Juvenile myoclonic epilepsy	1 (<0.1)	0	1 (0.1)	0	0	0
Seizure	1 (<0.1)	0	0	0	1 (0.2)	0
Psychiatric disorders	2 (0.1)	1 (0.1)	2 (0.2)	1 (0.2)	0	0
Aggression	1 (<0.1)	0	1 (0.1)	0	0	0
Suicide attempt	1 (<0.1)	0	1 (0.1)	0	0	0
Mental status changes	0	1 (0.1)	0	1 (0.2)	0	0

Abbreviations: MedDRA, Medical Dictionary for Regulatory Activities; NVX-CoV2373, 5 *μg* SARS-CoV-2 rS with 50 *μg* Matrix-M™ adjuvant; SARS-CoV-2, severe acute respiratory syndrome coronavirus 2; SARS-CoV-2 rS, severe acute respiratory syndrome coronavirus 2 recombinant spike protein nanoparticle vaccine.

© 2023 Áñez G et al. *JAMA Network Open*.

eFigure 3. Box Plot of Neutralizing Antibody Titers for SARS-CoV-2 Wild-Type Virus at Specified Time Points in Baseline Serologically Negative/PCR-Negative Adolescent Participants

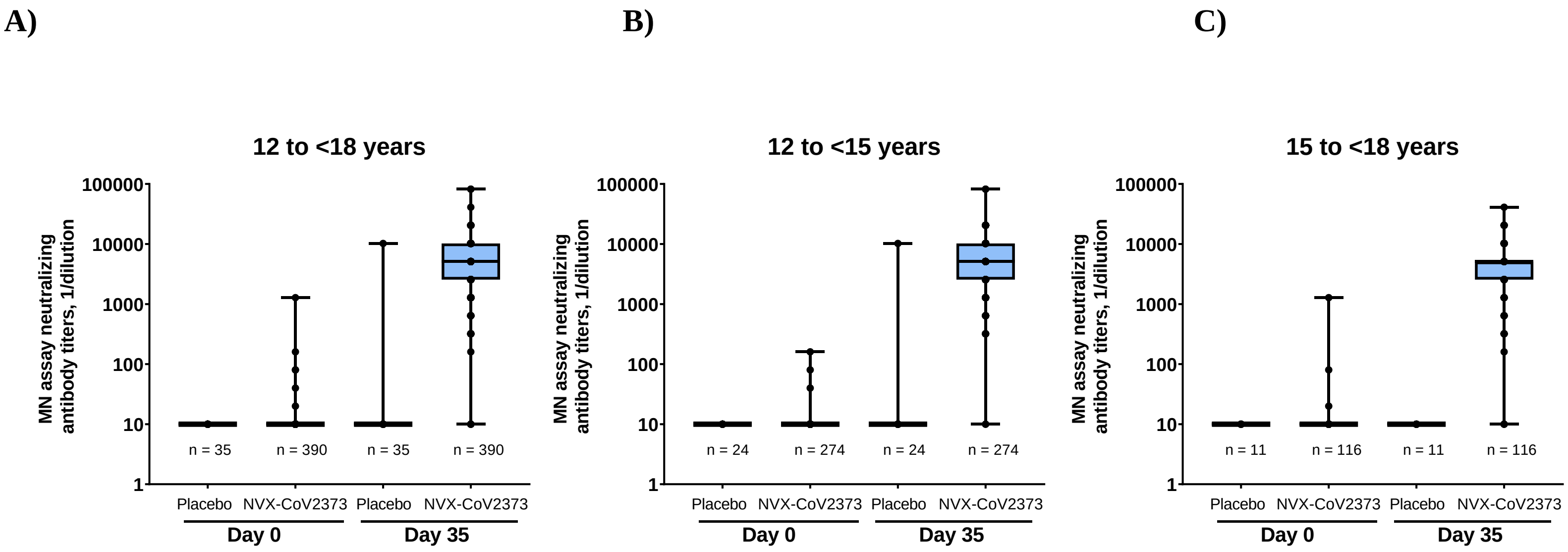

Note, titer values less than LLOQ (20) were replaced by 0.5 × LLOQ.
Data source: validated microneutralization assay conducted by 360biolabs.[9]

LLOQ, lower limit of quantification; MN, microneutralization; NVX-CoV2373, 5 µg SARS-CoV-2 rS + 50 µg Matrix-M adjuvant; PP-IMM, Per-Protocol Immunogenicity; SARS-CoV-2, severe acute respiratory syndrome coronavirus 2; SARS-CoV-2rS, NVX-CoV2373.

A) 12 to <18 Years of Age (PP-IMM Analysis Set), B) Participants 12 to <15 Years of Age (PP-IMM Analysis Set), and C) Participants 15 to <18 Years of Age (PP-IMM Analysis Set)

© 2023 Áñez G et al. *JAMA Network Open*.

eFigure 4. Box Plot of Serum IgG Antibody Concentrations to SARS-CoV-2 S Protein in Baseline Serologically Negative/PCR-Negative Adolescent Participants 12 to <18 Years of Age (PP-IMM Analysis Set)

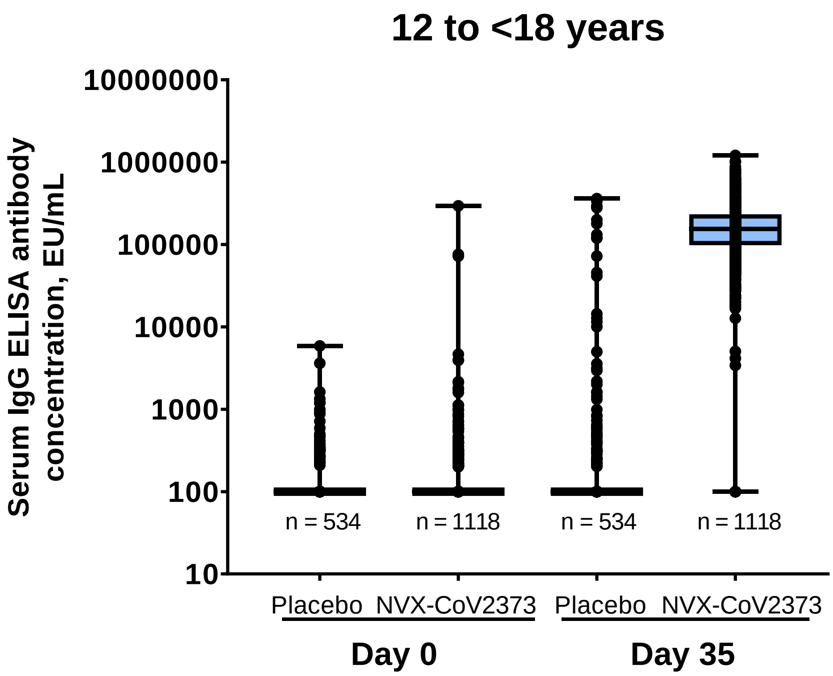

Note, titer values less than LLOQ (20) were replaced by 0.5 × LLOQ.
Data source: validated serum IgG ELISA assay conducted by Clinical Immunology Laboratory, Novavax.[6]
ELISA, enzyme-linked immunosorbent assay; IgG, immunoglobulin G; LLOQ, lower limit of quantification; NVX-CoV2373, 5 µg SARS-CoV-2 rS + 50 µg Matrix-M adjuvant; PP-IMM-2, Per-Protocol Immunogenicity 2; SARS-CoV-2, severe acute respiratory syndrome coronavirus 2; SARS-CoV-2rS, NVX-CoV2373.

© 2023 Áñez G et al. *JAMA Network Open.*

eFigure 5. Box Plot of hACE2 Inhibition Antibodies to SARS-CoV-2 S Protein in Baseline Serologically Negative/PCR-Negative Adolescent Participants 12 to <18 Years of Age (PP-IMM Analysis Set)

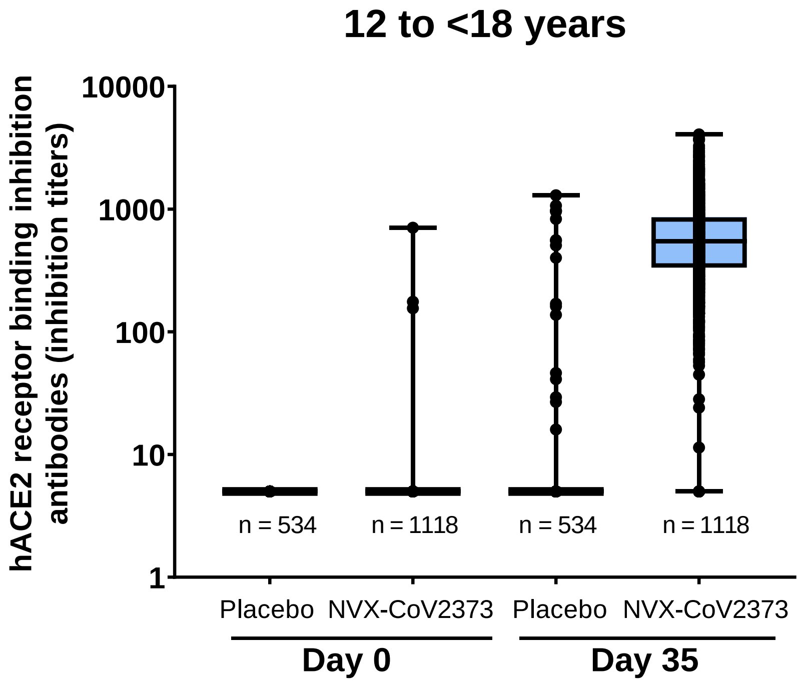

Note, titer values less than LLOQ (10 inhibition titers) were replaced by 0.5 × LLOQ.
Data source: validated hACE2 receptor binding inhibition assay conducted by Clinical Immunology laboratory, Novavax.[7]
hACE2, human angiotensin-converting enzyme 2; LLOQ, lower limit of quantification; NVX-CoV2373, 5 µg SARS-CoV-2 rS + 50 µg Matrix-M adjuvant; PP-IMM, Per-Protocol Immunogenicity; S, spike; SARS-CoV-2, severe acute respiratory syndrome coronavirus 2; SARS-CoV-2rS, NVX-CoV2373.

© 2023 Áñez G et al. *JAMA Network Open*.

eFigure 6. Serum IgG Antibody Concentrations to SARS-CoV-2 S Protein From Different Variants in Baseline Serologically Negative/PCR-Negative Adolescent Participants 12 to <18 Years of Age

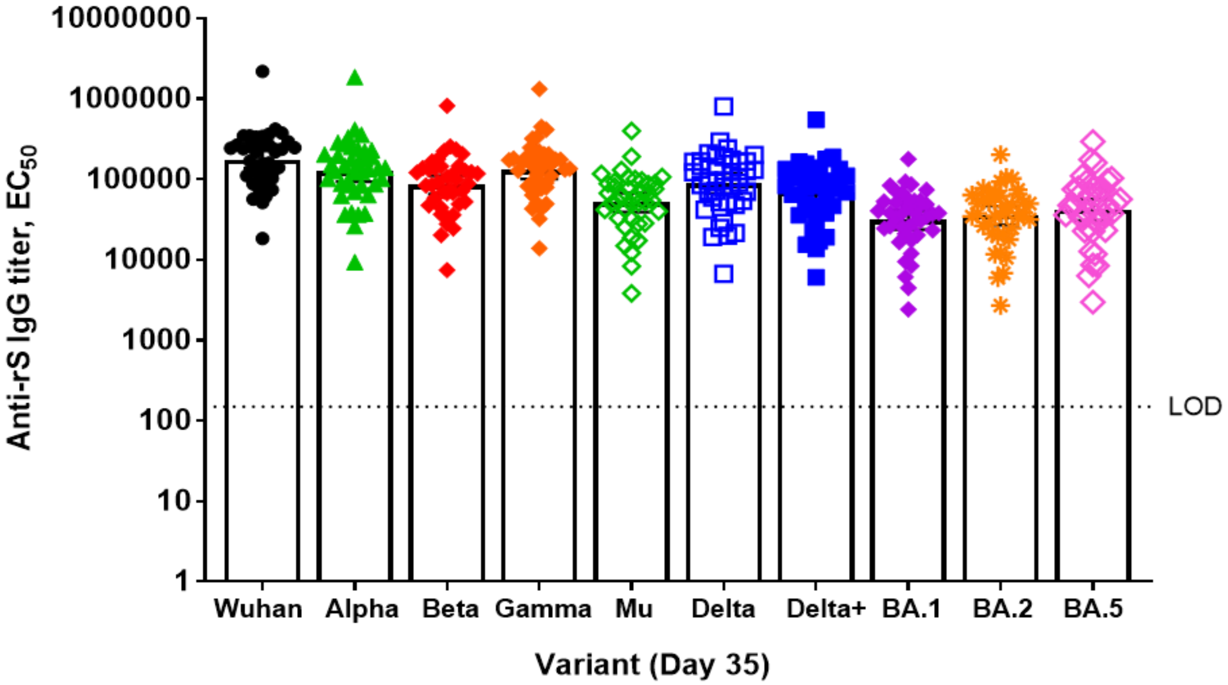

Data source: fit-for-purpose assay conducted by Vaccine Immunology Laboratory, Novavax.
IgG, immunoglobulin G; EC_{50}, half maximal effective concentration; LOD, limit of detection; rS, recombinant spike protein.
(n = 40, PP-IMM Analysis Set, Post Hoc Analysis, Nonvalidated, Fit-for-Purpose Assay)

© 2023 Áñez G et al. *JAMA Network Open*.

eFigure 7. hACE2 Inhibition Antibodies to SARS-CoV-2 S Protein From Different Variants in Baseline Serologically Negative/PCR-Negative Adolescent Participants 12 to <18 Years of Age

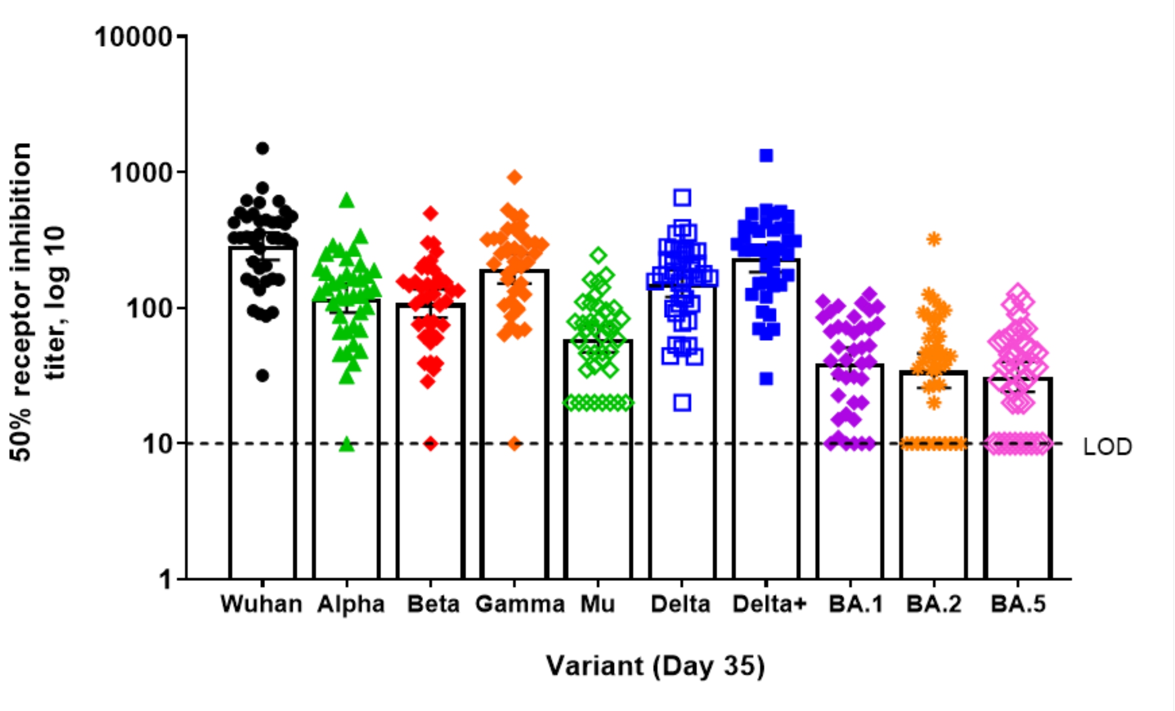

Data source: fit-for-purpose assay conducted by Vaccine Immunology Laboratory, Novavax.
LOD, limit of detection.
(n = 40, PP-IMM Analysis Set, Post Hoc Analysis, Nonvalidated Fit-for-Purpose Assay)

© 2023 Áñez G et al. *JAMA Network Open*.

eTable 14. Vaccine Efficacy Against RT-PCR-Confirmed Symptomatic Mild, Moderate, or Severe COVID-19 From First Injection Due to Any SARS-CoV-2 Variant in Adolescent Participants (Full Analysis Set)

Parameter	NVX-CoV2373 N=1484	Placebo N=748
Participants with occurrence of event,[a] n (%)	11 (0.7)	18 (2.4)
Median surveillance time[b] (days)	93.0	92.0
Log-linear model using modified Poisson regression[c]		
Mean disease incidence rate per year in 100 people	2.98	9.86
95% CI	1.65-5.39	6.22-15.61
Relative risk	0.30	
95% CI	0.14-0.64	
Vaccine efficacy (%)	69.74	
95% CI	36.00-85.69	

Abbreviations: CI, confidence interval; COVID-19, coronavirus disease 2019; NVX-CoV2373, 5 *μg* SARS-CoV-2 rS with 50 *μg* Matrix-M™ adjuvant; RT-PCR, reverse transcriptase-polymerase chain reaction; PP-EFF, Per-Protocol Efficacy; SARS-CoV-2 rS, severe acute respiratory syndrome coronavirus 2 recombinant spike protein nanoparticle vaccine; VE, vaccine efficacy.

[a]Event = first occurrence of RT-PCR-confirmed mild, moderate, or severe COVID-19 with onset of illness episode from the first injection within the surveillance period.

[b]Surveillance time was defined as the difference between the date at end of surveillance period (onset of first occurrence of event/ censoring) and date at start of surveillance period (first injection) + 1.

[c]Modified Poisson regression with logarithmic link function, treatment group as fixed effects and robust error variance.[8]

© 2023 Áñez G et al. *JAMA Network Open*.

eTable 15. Duration of Surveillance Time (Days) for Primary Efficacy End Point (Per-Protocol Efficacy Analysis Set)

	**NVX-CoV2373**	**Placebo**	**Total**
N	1205	594	1799
Mean (SD)	62.6 (13.57)	60.6 (15.85)	62.0 (14.39)
Median	64	63	64
IQR	58, 69	57, 69	57, 69
Min, Max	1, 135	1, 118	1, 135

IQR, interquartile range; max, maximum; min, minimum; SD, standard deviation

© 2023 Áñez G et al. *JAMA Network Open*.

eTable 16. Vaccine Efficacy Against RT-PCR-Confirmed Symptomatic Mild, Moderate, or Severe COVID-19 at Least 7 Days After Second Vaccination Due to Any SARS-CoV-2 Variant in Adolescent Participants Not Previously Exposed to SARS-CoV-2 (PP-EFF Analysis Set)

Parameter	NVX-CoV2373 N=1205	Placebo N=594
Participants with no occurrence of event,[a] n (%)	1199 (99.5)	580 (97.6)
Participants with occurrence of event,[b] n (%)	6 (0.5)	14 (2.4)
Severity of first occurrence, n (%)		
Mild	6 (0.5)	14 (2.4)
Moderate	0	0
Severe	0	0
Median surveillance time[c] (days)	64.0	63.0
Minimum-maximum	1-135	1-118
Log-linear model using modified Poisson regression[d]		
Mean disease incidence rate per year in 1000 people	2.90	14.20
95% CI	1.31-6.46	8.42-23.93
Relative risk	0.20	
95% CI	0.08-0.53	
Vaccine efficacy (%)	79.54	
95% CI	46.83-92.13	
Cox proportional hazard model (sensitivity analysis)[e]		
Vaccine efficacy (%)	79.39	
95% CI	46.34-92.08	

Abbreviations: CI, confidence interval; COVID-19, coronavirus disease 2019; NVX-CoV2373, 5 *μg* SARS-CoV-2 rS with 50 *μg* Matrix-M™ adjuvant; RT-PCR, reverse transcriptase-polymerase chain reaction; PP-EFF, Per-Protocol Efficacy; SARS-CoV-2 rS, severe acute respiratory syndrome coronavirus 2 recombinant spike protein nanoparticle vaccine; VE, vaccine efficacy.

[a]Includes participants with RT-PCR-confirmed infection who did not meet mild, moderate, or severe COVID-19 criteria.

[b]Event = first occurrence of RT-PCR-confirmed mild, moderate, or severe COVID-19 with onset of illness episode from at least 7 days after second vaccination within the surveillance period.

[c]Surveillance time was defined as the difference between the date at end of surveillance period (onset of first occurrence of event/ censoring) and date at start of surveillance period (7 days after the second injection) + 1.

[d]Modified Poisson regression with logarithmic link function, treatment group as fixed effects and robust error variance.[8]

[e]Cox-proportional hazard model with Efron's method for tie handling with vaccine group. Hazard ratio was used to estimate relative risk.

© 2023 Áñez G et al. *JAMA Network Open*.

eTable 17. Vaccine Efficacy Against RT-PCR-Confirmed Symptomatic Mild, Moderate or Severe COVID-19 at Least 7 Days After Second Vaccination Due to the SARS-CoV-2 Delta Variant in Adolescent Participants Not Previously Exposed to SARS-CoV-2 (PP-EFF Analysis Set)

Parameter	NVX-CoV2373 n= 1205	Placebo n = 594
Participants with no occurrence of event,[a] n (%)	1202 (99.8)	586 (98.7)
Participants with occurrence of event,[b] n (%)	3 (0.2)	8 (1.3)
Severity of first occurrence, n (%)		
Mild	3 (0.2)	8 (1.3)
Moderate	0	0
Severe	0	0
Median surveillance time[c] (days)	64.0	63.0
Minimum-maximum	1-135	1-118
Log-linear model using modified Poisson regression[d]		
Mean disease incidence rate per year in 1000 people	1.45	8.07
95% CI	0.47-4.49	4.04-16.11
Relative risk	0.18	
95% CI	0.05-0.68	
Vaccine efficacy (%)	82.04	
95% CI	32.42-95.23	

Abbreviations: CI, confidence interval; COVID-19, coronavirus disease 2019; NVX-CoV2373, 5 *μg* SARS-CoV-2 rS with 50 *μg* Matrix-M™ adjuvant; RT-PCR, reverse transcriptase-polymerase chain reaction; PP-EFF, Per-Protocol Efficacy; SARS-CoV-2 rS, severe acute respiratory syndrome coronavirus 2 recombinant spike protein nanoparticle vaccine; VE, vaccine efficacy.

[a]Includes participants with RT-PCR-confirmed infection who did not meet mild, moderate, or severe COVID-19 criteria due to the Delta variant.

[b]Event = first occurrence of RT-PCR-confirmed mild, moderate, or severe COVID-19 due to a VOC or VOI with onset of illness episode from at least 7 days after second vaccination within the surveillance period.

[c]Surveillance time was defined as the difference between the date at end of surveillance period (onset of first occurrence of event/censoring) and date at start of surveillance period (7 days after the second injection) + 1.

[d]Modified Poisson regression with logarithmic link function, treatment group as fixed effects and robust error variance.[8]

© 2023 Áñez G et al. *JAMA Network Open*.

eReferences.

1. Degli-Angeli E, Dragavon J, Huang M-L, et al. Validation and verification of the Abbott RealTime SARS-CoV-2 assay analytical and clinical performance. *J Clin Virol*. 2020;129:104474
2. Skalina KA, Goldstein DY, Sulail J, et al. Extended storage of SARS-CoV-2 nasopharyngeal swabs does not negatively impact results of molecular-based testing across three clinical platforms. *J Clin Pathol*. 2020;75(1):61-64
3. US Centers for Disease Control and Prevention. SARS-CoV-2 variant classifications and definitions. Accessed August 2022 https://www.cdc.gov/coronavirus/2019-ncov/variants/variant-info.html
4. Addetia A, Lin MJ, Peddu V, Roychoudhury P, Jerome KR, Greninger AL. Sensitive recovery of complete SARS-CoV-2 genomes from clinical samples by use of Swift Biosciences' SARS-CoV-2 Multiplex Amplicon Sequencing Panel. *J Clin Microbiol*. 2020;59(1):e02226-20
5. Shrestha L, Lin MJ, Xie H, et al. Clinical performance characteristics of the Swift Normalase Amplicon Panel for sensitive recovery of severe acute respiratory syndrome Coronavirus 2 genomes. *J Mol Diagn*. 2022;24(9):963-976
6. Keech C, Albert G, Cho I, et al. Phase 1–2 trial of a SARS-CoV-2 recombinant spike protein nanoparticle vaccine. *N Engl J Med*. 2020;383(24):2320-2332
7. Tian J-H, Patel N, Haupt R, et al. SARS-CoV-2 spike glycoprotein vaccine candidate NVX-CoV2373 immunogenicity in baboons and protection in mice. Nat Commun 2021;12(1):372
8. Zou G. A modified poisson regression approach to prospective studies with binary data. *Am J Epidemiol*. 2004;159(7):702-706
9. Formica N, Mallory R, Albert G, et al. Different dose regimens of a SARS-CoV-2 recombinant spike protein vaccine (NVX-CoV2373) in younger and older adults: a phase 2 randomized placebo-controlled trial. *PLoS Med*. 2021;18(10):e1003769
10. US Food and Drug Administration, Center for Biologics Evaluation and Research (US). Guidance for industry: toxicity grading scale for healthy adult and adolescent volunteers enrolled in preventive vaccine clinical trials, September 2007 https://www.fda.gov/media/73679/download

© 2023 Áñez G et al. *JAMA Network Open*.
