# Supplementary material for: Safety, Immunogenicity, and Efficacy of the NVX-CoV2373 COVID-19 Vaccine in Adolescents
Source: JAMA Netw Open. Author manuscript; Available in PMC 2023 Sep 28. (PMC10536880; doi:10.1001/jamanetworkopen.2023.9135)
Supplement: Supplementary file 2 [file NIHMS1927823-supplement-Supplement_2_Trial_Protocol_and_Statistical_Analysis_Plan.pdf]

TITLE PAGE

A Phase 3, Randomized, Observer-Blinded, Placebo-Controlled Study to Evaluate the Efficacy, Safety, and Immunogenicity of a SARS-CoV-2 Recombinant Spike Protein Nanoparticle Vaccine (SARS-CoV-2 rS) with Matrix-M1™ Adjuvant in Adult Participants ≥ 18 Years with a Pediatric Expansion in Adolescents (12 to < 18 Years)

Protocol Number:	2019nCoV-301
Amendment Number:	Version 9.0
Test Product:	SARS-CoV-2 rS with Matrix-M1™ Adjuvant
Indication:	Prevention of COVID-19 caused by SARS-CoV-2
Development Phase:	Phase 3
Sponsor:	Novavax, Inc.<br>21 Firstfield Road<br>Gaithersburg, MD 20878<br>United States
IND:	022430
EudraCT:	2020-004042-11
Approval Date:	14 May 2021

The confidential information in this document is provided to you as an investigator, potential investigator or consultant for review by you, your staff and applicable Independent Ethics Committee and/or Institutional Review Board. It is understood that the information will not be disclosed to others without written authorization from Novavax, Inc. except to the extent necessary to obtain informed consent from those persons, or their legally authorized representative, to whom the vaccine may be administered.

SPONSOR SIGNATURE PAGE

PROTOCOL TITLE: A Phase 3, Randomized, Observer-Blinded, Placebo-Controlled Study to Evaluate the Efficacy, Safety, and Immunogenicity of a SARS-CoV-2 Recombinant Spike Protein Nanoparticle Vaccine (SARS-CoV-2 rS) with Matrix-M1™ Adjuvant in Adult Participants ≥ 18 Years with a Pediatric Expansion in Adolescents (12 to < 18 Years)

PROTOCOL NUMBER: 2019nCoV-301

Novavax, Inc.

DocuSigned by:

[redacted]

14-May-21 | 12:56 EDT

Date (day/month/year)

DocuSigned by:

[redacted]

14-May-21 | 12:56 EDT

Date (day/month/year)

1 GENERAL INFORMATION

A Phase 3, Randomized, Observer-Blinded, Placebo-Controlled Study to Evaluate the Efficacy, Safety, and Immunogenicity of a SARS-CoV-2 Recombinant Spike Protein Nanoparticle Vaccine (SARS-CoV-2 rS) with Matrix-M1™ Adjuvant in Adult Participants ≥ 18 Years with a Pediatric Expansion in Adolescents (12 to < 18 Years)

Protocol Number:	2019nCoV-301
Amendment:	Version 9.0
Approval Date:	14 May 2021
Sponsor:	Novavax, Inc.<br>21 Firstfield Road<br>Gaithersburg, MD 20878<br>United States
Clinical Research Organization:	ICON Clinical Research, Ltd.<br>[redacted]
Sponsor Signatories:	[redacted] Clinical Operations<br>Novavax, Inc.<br>21 Firstfield Road<br>Gaithersburg, MD 20878<br>United States
Sponsor Medical Expert:	[redacted] Clinical Development, [redacted]<br>Novavax, Inc.<br>21 Firstfield Road<br>Gaithersburg, MD 20878<br>United States
National Coordinating Investigator for Mexico:	[redacted]

2 STUDY SYNOPSIS

**Name of Sponsor/Company:**<br>Novavax, Inc.	
**Name of Product:**<br>SARS-CoV-2 rS	
**Name of Active Ingredient:**<br>SARS-CoV-2 rS + Matrix-M1 adjuvant	
**Title of Study:**<br>A Phase 3, Randomized, Observer-Blinded, Placebo-Controlled Study to Evaluate the Efficacy, Safety and Immunogenicity of a SARS-CoV-2 Recombinant Spike Protein Nanoparticle Vaccine (SARS-CoV-2 rS) with Matrix-M1™ Adjuvant in Adult Participants ≥ 18 Years with a Pediatric Expansion in Adolescents (12 to < 18 Years)	
**Principal Investigator:**<br>This is a multicenter study.	
**Study Centers:**<br>Approximately 160 study sites across North America and globally, if needed	
**Planned Study Period:**<br>Dec 2020 to 2023	**Development Phase:**<br>Phase 3
**Main Study**	
**Objectives:**	**Endpoints:**
**Primary Objective:**<br>• To evaluate the efficacy of a 2-dose regimen of SARS-CoV-2 rS adjuvanted with Matrix-M1 compared to placebo against polymerase chain reaction (PCR)-confirmed symptomatic coronavirus disease 2019 (COVID-19) illness diagnosed ≥ 7 days after completion of the second injection in the initial set of vaccinations of adult participants ≥ 18 years of age.	**Primary Endpoint:**<br>• First episode of PCR-positive mild, moderate, or severe COVID-19, where severity is defined as:<br>**Mild COVID-19 (≥ 1 of the following):**<br>• Fever (defined by subjective or objective measure, regardless of use of anti-pyretic medications)<br>• New onset cough<br>• ≥ 2 additional COVID-19 symptoms:<br>o New onset or worsening of shortness of breath or difficulty breathing compared to baseline.<br>o New onset fatigue.<br>o New onset generalized muscle or body aches.<br>o New onset headache.<br>o New loss of taste or smell.<br>o Acute onset of sore throat, congestion or runny nose.<br>o New onset nausea, vomiting or diarrhea.<br>**OR Moderate COVID-19 (≥ 1 of the following):**<br>• High fever (≥ 38.4°C) for ≥ 3 days (regardless of use of anti-pyretic medications, need not be contiguous days).<br>• Any evidence of significant lower respiratory tract infection (LRTI):<br>o Shortness of breath (or breathlessness or difficulty breathing) with or without exertion (greater than baseline).

	○ Tachypnea: 24 to 29 breaths per minute at rest.<br>○ SpO_2: 94% to 95% on room air.<br>○ Abnormal chest X-ray or chest computerized tomography (CT) consistent with pneumonia or LRTI.<br>• Adventitious sounds on lung auscultation (eg, crackles/rales, wheeze, rhonchi, pleural rub, stridor).<br>**OR Severe COVID-19 (≥ 1 of the following):**<br>• Tachypnea: ≥ 30 breaths per minute at rest.<br>• Resting heart rate ≥ 125 beats per minute.<br>• SpO_2: ≤ 93% on room air or PaO_2/FiO_2 < 300 mmHg.<br>• High flow oxygen (O_2) therapy or non-invasive ventilation (NIV)/non-invasive positive pressure ventilation (NIPPV) (eg, continuous positive airway pressure [CPAP] or bilevel positive airway pressure [BiPAP]).<br>• Mechanical ventilation or extracorporeal membrane oxygenation (ECMO).<br>• One or more major organ system dysfunction or failure to be defined by diagnostic testing/clinical syndrome/interventions, including any of the following:<br>○ Acute respiratory failure, including acute respiratory distress syndrome (ARDS).<br>○ Acute renal failure.<br>○ Acute hepatic failure.<br>○ Acute right or left heart failure.<br>○ Septic or cardiogenic shock (with shock defined as systolic blood pressure [SBP] < 90 mm Hg OR diastolic blood pressure [DBP] < 60 mm Hg).<br>○ Acute stroke (ischemic or hemorrhagic).<br>○ Acute thrombotic event: acute myocardial infarction (AMI), deep vein thrombosis (DVT), pulmonary embolism (PE).<br>○ Requirement for: vasopressors, systemic corticosteroids, or hemodialysis.<br>• Admission to an intensive care unit (ICU).<br>• Death.
**Key Secondary Objective:**<br>• To evaluate the efficacy of a 2-dose regimen of SARS-CoV-2 rS adjuvanted with Matrix-M1 compared to placebo against PCR-confirmed symptomatic COVID-19 illness due to a SARS-CoV-2 variant not considered as a "variant of concern / interest" according to the CDC Variants Classification, diagnosed ≥ 7 days after completion of the second injection in the initial set of vaccinations of adult participants ≥ 18 years of age.	**Key Secondary Endpoint:**<br>• First episode of PCR-positive COVID-19, as defined under the primary endpoint, shown by gene sequencing to represent a variant not considered as a "variant of concern / interest" according to the CDC Variants Classification.

**Other Secondary Objectives:**	**Other Secondary Endpoints:**
• To evaluate the efficacy of a 2-dose regimen of SARS-CoV-2 rS adjuvanted with Matrix-M1 compared to placebo against PCR-confirmed moderate-to-severely symptomatic COVID-19 illness diagnosed ≥ 7 days after completion of the second vaccination in the initial set of vaccinations of adult participants ≥ 18 years of age.<br>• To assess vaccine efficacy (VE) against ANY symptomatic SARS-CoV-2 infection.<br>• To assess VE according to race and ethnicity.<br>• To assess VE in high-risk adults versus non-high-risk adults (high-risk is defined by age ≥ 65 years with or without co-morbidities or age < 65 years **with** co-morbidities [eg, obesity (body mass index [BMI] > 30 kg/m^2), chronic kidney or lung disease, cardiovascular disease and diabetes mellitus type 2] and/or by life circumstance [living or working conditions involving known frequent exposure to SARS-CoV-2 or to densely populated circumstances (eg, factory or meat packing plants, essential retail workers, etc)]).<br>• To assess the durability of vaccine efficacy (measured by all defined efficacy endpoints) in initial active vaccine recipients versus crossover (delayed) active vaccine recipients.<br>• To describe the humoral immune response to vaccine in terms of neutralizing antibody to SARS-CoV-2 for all Immunogenicity Population Participants, and for subsets with and without prior SARS-CoV-2 exposure determined by detectable anti-nucleoprotein (NP) antibodies at baseline.<br>• To assess the immune response to vaccine by immunoglobulin G (IgG) antibody to SARS-CoV-2 S protein and human angiotensin-converting enzyme 2 (hACE2) inhibiting antibodies at Day 35 and later for all Immunogenicity Population participants, and for subsets with and without prior SARS-CoV-2 exposure determined by detectable anti-NP antibodies at baseline.<br>• To assess the durability of immune response (IgG antibody to SARS-CoV-2 S protein, hACE2 inhibition, and microneutralization [MN]) at 12, 18 and 24 months of study in all Immunogenicity Population participants, and for subsets with and without detectable anti-NP antibodies at baseline or prior to crossover set of vaccinations.	• First episode of PCR-positive moderate or severe COVID-19, as defined under the primary endpoint.<br>• ANY symptomatic SARS-CoV-2 infection, defined as: PCR-positive nasal swab **and** ≥ 1 of any of the following symptoms:<br>o Fever.<br>o New onset cough.<br>o New onset or worsening of shortness of breath or difficulty breathing compared to baseline.<br>o New onset fatigue.<br>o New onset generalized muscle or body aches.<br>o New onset headache.<br>o New loss of taste or smell.<br>o Acute onset of sore throat, congestion or runny nose.<br>o New onset nausea, vomiting or diarrhea.<br>• Neutralizing antibody titers from Immunogenicity Population at Days 0, 35 and immediately prior to administration of the crossover set of vaccinations.<br>• Serum IgG levels to SARS-CoV-2 S protein, hACE2 inhibition titers from Immunogenicity Population at Days 0, 35 and immediately prior to administration of the crossover set of vaccinations.<br>• Serum IgG levels to SARS-CoV-2 S protein, MN and hACE2 inhibition titers from Immunogenicity Population at Months 12, 18 and 24.<br>• Description of course, treatment and severity of COVID-19 reported after a PCR-confirmed case via the Endpoint Form.<br>• Reactogenicity incidence and severity (mild, moderate or severe) recorded by all participants on their electronic patient-reported outcome diary application (eDiary) on days of vaccination and subsequent 6 days (total 7 days after each vaccine injection in the initial set of vaccinations).<br>o Reactogenicity endpoints include injection site reactions:<br>▪ Pain.<br>▪ Tenderness.<br>▪ Erythema.<br>▪ Swelling/induration.<br>o Systemic reactions:<br>▪ Fever.<br>▪ Malaise.<br>▪ Fatigue.<br>▪ Arthralgia.<br>▪ Myalgia.<br>▪ Headache.

• To describe and compare the safety experience for the vaccine versus placebo in adult participants ≥ 18 years of age based on solicited short-term reactogenicity by toxicity grade for 7 days following each vaccination (Days 0 and 21) after the initial set of vaccinations.<br>• To assess overall safety through 49 days (28 days after second injection of each set of vaccinations [initial and crossover]) and to compare vaccine versus placebo for all unsolicited AEs and medically attended adverse events (MAAEs).<br>• To assess the frequency and severity of MAAEs attributed to vaccine, adverse events of special interest (AESIs), or serious adverse events (SAEs) through the end of study (EoS) and to compare vaccine versus placebo after each set of vaccinations (initial and crossover).<br>• To assess all-cause mortality in vaccine versus placebo recipients after each set of vaccinations (initial and crossover).<br>• To describe the severity and course of COVID-19 in vaccine versus placebo recipients in terms of healthcare requirements, utilization and medical assessments after each set of vaccinations (initial and crossover).<br>• To assess the proportion of participants (vaccine versus placebo recipients) with SARS-CoV-2 infection determined by anti-SARS-CoV-2 NP antibodies, including specifically asymptomatic infection, across the 2 years of study follow-up.<br>• To assess the VE against SARS-CoV-2 infection determined by anti-SARS-CoV-2 NP antibodies, regardless of whether the infection was symptomatic.<br>• To assess in a subset of participants the immunogenicity of a new lot of SARS-CoV-2 rS with Matrix-M1 adjuvant in comparison to the lot utilized in the initial set of vaccinations (ie, immunobridging).<br>• To assess non-inferiority of the neutralizing antibody response for adolescent participants seronegative to anti-SARS-CoV-2 NP antibodies at baseline, compared with that observed in seronegative adult participants 18 to < 26 years of age from the **Adult Main Study** (Immunogenicity Population participants before crossover).	▪ Nausea/vomiting.<br>• Incidence and severity of MAAEs through 49 days, ie, 28 days after second injection of each set of vaccinations (initial and crossover).<br>• Incidence and severity of unsolicited AEs through 49 days, ie, 28 days after second injection of each set of vaccinations (initial and crossover).<br>• Incidence and severity of MAAEs attributed to study vaccine, SAEs and AESIs through Month 12.<br>• Incidence and severity of SAEs, MAAEs attributed to study vaccine and AESIs during Month 12 through Month 24 or the EoS.<br>• Death due to any cause.<br>• Data points to be collected for healthcare requirements, utilization and medical assessments from participants who become ill on study will be defined in a separate substudy protocol.<br>• Antibodies to SARS-CoV-2 NP at Days 0 and 35, immediately prior to administration of the crossover set of vaccinations, and at Months 12, 18 and 24 will be used to determine natural infection and to determine the incidence of asymptomatic infection acquired during study follow-up.<br>• Antibodies to SARS-CoV-2 NP, regardless of whether the infection was symptomatic.<br>• IgG antibodies to SARS-CoV-2 rS at approximately 35 days after the first crossover vaccination in approximately 300 active vaccine recipients 18 to ≤ 64 years of age enrolled at selected study sites.<br>• Neutralizing antibody response at Day 35 for all adolescent participants seronegative to anti-SARS-CoV-2 NP antibodies at baseline, compared with that observed in seronegative adult participants 18 to < 26 years of age from the **Adult Main Study** (Immunogenicity Population participants before crossover).
**Exploratory Objectives:**<br>• To evaluate the efficacy of study vaccine compared to placebo against PCR-confirmed symptomatic COVID-19 illness due to a	**Exploratory Endpoints:**<br>• First episode of PCR-positive COVID-19, as defined under the primary endpoint, shown by gene sequencing

SARS- CoV-2 variant considered as a "variant of concern / interest" according to the CDC Variants Classification, diagnosed ≥ 7 days after completion of the second vaccination in the initial set of vaccinations of adult participants ≥ 18 years of age.<br>• To assess cell-mediated response:<br>o Type 1 T helper (Th1) or Type 2 T helper (Th2) predominance after initial set of vaccinations.<br>• To contribute to a larger cross-study National Institutes of Health (NIH) effort to define correlates of risk and protection against SARS-CoV-2 infection and disease.<br>• To assess impact of vaccination on nasal viral load in nasal swabs of participants who develop symptoms of possible COVID-19.<br>• To assess impact of vaccination on asymptomatic SARS-CoV-2 PCR positivity and viral load at the time of the crossover set of vaccinations.<br>• To describe sequences of the genetic material from SARS-CoV-2 viruses detected in COVID-19 cases to evaluate possible viral mutations that may be associated with breakthrough infections.	to represent a "variant of concern / interest" according to the CDC Variants Classification.<br>• Th1 or Th2 responses, eg, interleukin [IL]-2, IL-4, IL-5, IL-13, tumor necrosis factor alpha (TNF-α), interferon gamma (IFN-γ) in whole blood and/or harvested peripheral blood mononuclear cell (PBMCs) prior to and on Day 35 after the initial set of vaccinations.<br>• Serum samples from a designated subset of up to approximately 4,500 Immunogenicity Population participants to be transferred to National Institute of Allergy and Infectious Diseases (NIAID) for testing and analysis to determine correlates of risk and protection. Endpoints will be described in a separate statistical analysis plan developed by external statistics groups (eg, COVID-19 Prevention Network [CoVPN], Operation Warp Speed [OWS]).<br>• Quantitative PCR tests may be performed on nasal swabs collected from this trial to assess whether vaccination impacts viral shedding.<br>• Quantitative PCR tests performed on nasal swabs collected immediately prior to administration of blinded crossover vaccination to assess impact of initial vaccination on frequency of asymptomatic SARS-CoV-2 infection and level of viral shedding.<br>• Next-generation sequencing of viral genomes detected in nasal swabs tested by PCR to describe the genetic evolution of circulating SARS-CoV-2 strains during the conduct of the study.
**Pediatric Expansion**	
**Objectives:**	**Endpoints:**
**Primary Objectives:**<br>• To evaluate the efficacy of a 2-dose regimen of SARS-CoV-2 rS adjuvanted with Matrix-M1 compared to placebo against PCR-confirmed symptomatic COVID-19 illness diagnosed ≥ 7 days after completion of the second injection in the initial set of vaccinations of adolescent participants 12 to < 18 years of age.<br>• To describe the safety experience for the vaccine versus placebo in adolescent participants (12 to < 18 years of age) based on solicited short-term reactogenicity by toxicity grade for 7 days following each vaccination (Days 0 and 21) after the initial set of vaccinations.	**Primary Endpoints:**<br>• First episode of PCR-positive mild, moderate, or severe COVID-19, where severity is defined as:<br>**Mild COVID-19 (≥ 1 of the following):**<br>• Fever (defined by subjective or objective measure, regardless of use of anti-pyretic medications)<br>• New onset cough<br>• ≥ 2 additional COVID-19 symptoms:<br>o New onset or worsening of shortness of breath or difficulty breathing compared to baseline.<br>o New onset fatigue.<br>o New onset generalized muscle or body aches.<br>o New onset headache.<br>o New loss of taste or smell.<br>o Acute onset of sore throat, congestion or runny nose.<br>o New onset nausea, vomiting or diarrhea.<br>**OR Moderate COVID-19 (≥ 1 of the following):**<br>• High fever (≥ 38.4°C) for ≥ 3 days (regardless of use of anti-pyretic medications, need not be contiguous days).

• To assess overall safety through 49 days (28 days after second injection of each set of vaccinations [initial and crossover]) by comparing vaccine versus placebo for all unsolicited AEs and MAAEs.<br>• To assess the frequency and severity of MAAEs attributed to vaccine, AESIs, or SAEs through the EoS and to compare vaccine versus placebo after each set of vaccinations (initial and crossover).<br>• To assess all-cause mortality in vaccine versus placebo recipients after each set of vaccinations (initial and crossover).<br>• To assess non-inferiority of the neutralizing antibody response for all adolescent participants seronegative to anti-SARS-CoV-2 NP antibodies at baseline, compared with that observed in seronegative adult participants 18 to < 26 years of age from the **Adult Main Study** (Immunogenicity Population participants before crossover).	• Any evidence of significant LRTI:<br>o Shortness of breath (or breathlessness or difficulty breathing) with or without exertion (greater than baseline).<br>o Tachypnea: 24 to 29 breaths per minute at rest.<br>o SpO_2: 94% to 95% on room air.<br>o Abnormal chest X-ray or chest CT consistent with pneumonia or LRTI.<br>• Adventitious sounds on lung auscultation (eg, crackles/rales, wheeze, rhonchi, pleural rub, stridor).<br>**OR Severe COVID-19 (≥ 1 of the following):**<br>• Tachypnea: ≥ 30 breaths per minute at rest.<br>• Resting heart rate ≥ 125 beats per minute.<br>• SpO_2: ≤ 93% on room air or PaO_2/FiO_2 < 300 mmHg.<br>• High flow oxygen (O_2) therapy or NIV/NIPPV (eg, CPAP or BiPAP).<br>• Mechanical ventilation or ECMO.<br>• One or more major organ system dysfunction or failure to be defined by diagnostic testing/clinical syndrome/interventions, including any of the following:<br>o Acute respiratory failure, including ARDS.<br>o Acute renal failure.<br>o Acute hepatic failure.<br>o Acute right or left heart failure.<br>o Septic or cardiogenic shock (with shock defined as SBP < 90 mm Hg OR DBP < 60 mm Hg).<br>o Acute stroke (ischemic or hemorrhagic).<br>o Acute thrombotic event: AMI, DVT, PE.<br>o Requirement for: vasopressors, systemic corticosteroids, or hemodialysis.<br>• MIS-C, as per the CDC definition:<br>o An individual aged < 21 years presenting with fever (>38.0°C for ≥24 hours, or report of subjective fever lasting ≥24 hours), laboratory evidence of inflammation (including, but not limited to, one or more of the following: an elevated C-reactive protein (CRP), erythrocyte sedimentation rate (ESR), fibrinogen, procalcitonin, d-dimer, ferritin, lactic acid dehydrogenase (LDH), or interleukin 6 (IL-6), elevated neutrophils, reduced lymphocytes and low albumin), and evidence of clinically severe illness requiring hospitalization, with multisystem (>2) organ involvement (cardiac, renal, respiratory, hematologic, gastrointestinal, dermatologic or neurological); AND<br>o No alternative plausible diagnoses; AND<br>o Positive for current or recent SARS-CoV-2 infection by RT-PCR, serology, or antigen test; or COVID-19

	exposure within the 4 weeks prior to the onset of symptoms.<br>• Admission to an ICU.<br>• Death<br>**Safety Endpoints:**<br>• Reactogenicity incidence, duration and severity (mild, moderate or severe) recorded by parent(s)/caregiver(s) on their electronic patient-reported outcome diary application (eDiary) on days of vaccination and subsequent 6 days (total 7 days after each vaccine injection in the initial set of vaccinations).<br>o Reactogenicity endpoints include injection site reactions:<br>▪ Pain.<br>▪ Tenderness.<br>▪ Erythema.<br>▪ Swelling/induration.<br>o Systemic reactions:<br>▪ Fever.<br>▪ Malaise.<br>▪ Fatigue.<br>▪ Arthralgia.<br>▪ Myalgia.<br>▪ Headache.<br>▪ Nausea/vomiting.<br>• Incidence and severity of MAAEs through 49 days, ie, 28 days after second injection of each set of vaccinations (initial and crossover).<br>• Incidence and severity of unsolicited AEs through 49 days, ie, 28 days after second injection of each set of vaccinations (initial and crossover).<br>• Incidence and severity of MAAEs attributed to study vaccine, SAEs and AESIs through Month 12.<br>• Incidence and severity of SAEs (including COVID-19 diagnoses), MAAEs attributed to study vaccine and AESIs during Month 12 through Month 24 or the EoS.<br>• Death due to any cause.<br>**Effectiveness Endpoint:**<br>• Neutralizing antibody response at Day 35 for all adolescent participants seronegative to anti-SARS-CoV-2 NP antibodies at baseline, compared with that observed in seronegative adult participants 18 to < 26 years of age from the **Adult Main Study** (Immunogenicity Population participants before crossover).

Secondary Objectives:	Secondary Endpoints:
• To evaluate the efficacy of a 2-dose regimen of SARS-CoV-2 rS adjuvanted with Matrix-M1 compared to placebo against PCR-confirmed symptomatic COVID-19 illness due to SARS-CoV-2 variant not considered as a "variant of concern / interest" according to the CDC Variants Classification, diagnosed ≥ 7 days after completion of the second injection in the initial set of vaccinations of adolescent participants 12 to < 18 years of age.<br>• To evaluate the efficacy of a 2-dose regimen of SARS-CoV-2 rS adjuvanted with Matrix-M1 compared to placebo against PCR-confirmed moderate-to-severely symptomatic COVID-19 illness diagnosed ≥ 7 days after completion of the second vaccination in the initial set of vaccinations of adolescent participants 12 to < 18 years of age.<br>• To assess VE against ANY symptomatic SARS-CoV-2 infection.<br>• To assess VE according to race and ethnicity.<br>• To assess the durability of VE (measured by all defined efficacy endpoints) in adolescents after initial active vaccine recipients versus crossover (delayed) active vaccine recipients.<br>• To monitor occurrence and severity of COVID-19 cases by following participant-reported symptoms.<br>• To assess the neutralizing antibody response to SARS-CoV-2 for adolescent participants by subsets with and without anti-NP antibodies at baseline, compared with that observed in adult participants 18 to < 26 years of age from the **Adult Main Study** (Immunogenicity Population participants before crossover).<br>• To assess the anti-spike immunoglobulin G (IgG) antibody response and human angiotensin converting enzyme 2 (hACE2) inhibiting antibody response at Day 35 for adolescent participants by subsets with and without detectable anti-NP antibodies at baseline, compared with that observed in adult participants 18 to < 26 years of age from the **Adult Main Study** (Immunogenicity Population participants before crossover).<br>• To assess the durability of immune response (anti-rS IgG antibody, hACE2 inhibition, and microneutralization [MN] titers) at 12, 18 and 24 months of study in all adolescent participants, and for subsets with and without	• First episode of PCR-positive COVID-19, as defined under the primary endpoint, shown by gene sequencing to represent a variant not considered as a "variant of concern / interest" according to the CDC Variants Classification.<br>• First episode of PCR-positive moderate or severe COVID-19, as defined under the primary endpoint.<br>• ANY symptomatic SARS-CoV-2 infection, defined as: PCR-positive nasal swab **and** ≥ 1 of any of the following symptoms:<br>o Fever.<br>o New onset cough.<br>o New onset or worsening of shortness of breath or difficulty breathing compared to baseline.<br>o New onset fatigue.<br>o New onset generalized muscle or body aches.<br>o New onset headache.<br>o New loss of taste or smell.<br>o Acute onset of sore throat, congestion or runny nose.<br>o New onset nausea, vomiting or diarrhea.<br>• Proportion of adolescent participants reporting SARS-CoV-2 infection (COVID-19) from Day 28 through end of Year 1, with severity classification as defined in the **Adult Main Study** (mild, moderate, or severe).<br>• Neutralizing antibody response at Day 35 for adolescent participants by age strata and with and without anti-SARS-CoV-2 NP antibodies at baseline, compared with that observed in adult participants 18 to < 26 years of age from the **Adult Main Study** (Immunogenicity Population participants before crossover).<br>• Antibodies to SARS-CoV-2 NP at Days 0 and 35, and at Months 12, 18 and 24 will be used to determine natural infection and to determine the incidence of asymptomatic infection acquired during study follow-up.<br>• Serum IgG levels to SARS-CoV-2 S protein, hACE2 inhibition titers 14 days after second injection of the initial vaccination series (Day 35) in adolescent participants and subsets with and without anti-NP antibodies at baseline.<br>• Serum IgG levels to SARS-CoV-2 S protein, MN and hACE2 inhibition titers at Months 12, 18 and 24.<br>• Description of course, treatment and severity of COVID-19 reported after a PCR-confirmed case via the Endpoint Form.<br>• Antibodies to SARS-CoV-2 NP, regardless of whether the infection was symptomatic.

detectable anti-NP antibodies at baseline or prior to crossover set of vaccinations, with that observed in adult participants 18 to < 26 years of age from the **Adult Main Study** (Immunogenicity Population participants before crossover).<br>• To assess the proportion of adolescent participants (vaccine versus placebo recipients) with SARS-CoV-2 infection determined by anti-SARS-CoV-2 NP antibodies, including specifically asymptomatic infection, across the 2 years of study follow-up.<br>• To assess the VE against SARS-CoV-2 infection determined by anti-SARS-CoV-2 NP antibodies in adolescent participants, regardless of whether the infection was symptomatic.	
**Exploratory Objectives:**<br>• To evaluate the efficacy of study vaccine compared to placebo against PCR-confirmed symptomatic COVID-19 illness due to a SARS-CoV-2 variant considered as a "variant of concern / interest" according to the CDC Variants Classification, diagnosed ≥ 7 days after completion of the second vaccination in the initial set of vaccinations of adolescent participants 12 to < 18 years of age.<br>• To assess cell-mediated response in a cohort of 50 adolescent participants at baseline and at Days 7 and 28:<br>o Type 1 T helper (Th1) or Type 2 T helper (Th2) predominance.<br>• To assess impact of vaccination on nasal viral load in nasal swabs of adolescent participants who develop symptoms of possible COVID-19.<br>• To assess impact of vaccination on asymptomatic SARS-CoV-2 PCR positivity and viral load in adolescent participants at the time of the crossover set of vaccinations.<br>• To describe sequences of the genetic material from SARS-CoV-2 viruses detected in COVID-19 cases in adolescent participants to evaluate possible viral mutations that may be associated with breakthrough infections.	**Exploratory Endpoints:**<br>• First episode of PCR-positive COVID-19, as defined under the primary endpoint, shown by gene sequencing to represent a "variant of concern / interest" according to the CDC Variants Classification.<br>• Th1 or Th2 responses, eg, interleukin [IL]-2, IL-4, IL-5, IL-13, tumor necrosis factor alpha (TNF-α), interferon gamma (IFN-γ) in whole blood and/or harvested peripheral blood mononuclear cell (PBMCs).<br>• Quantitative PCR tests may be performed on nasal swabs collected from this trial to assess whether vaccination impacts viral shedding.<br>• Quantitative PCR tests performed on nasal swabs collected immediately prior to administration of blinded crossover vaccination to assess impact of initial vaccination on frequency of asymptomatic SARS-CoV-2 infection and level of viral shedding.<br>• Next-generation sequencing of viral genomes detected in nasal swabs tested by PCR to describe the genetic evolution of circulating SARS-CoV-2 strains during the conduct of the study.

Study Design:

This is a Phase 3, randomized, observer-blinded, placebo-controlled study to evaluate the efficacy, safety and immunogenicity of SARS-CoV-2 rS with Matrix-M1 adjuvant in adult participants ≥ 18 years of age (**Adult Main Study**) with a **Pediatric Expansion**. In the **Adult Main Study**, participants will be stratified by age group, and enrollment will occur concurrently within the 2 age strata, 18 to ≤ 64 years and ≥ 65 years. In the **Pediatric Expansion**, adolescent participants 12 to < 18 years of age will be enrolled without further stratification.

The study will be a multicenter, global study with countries selected based on the expected COVID-19 epidemiology and healthcare system characteristics.

Enrollment

In the **Adult Main Study**, at least 25% of the study population is intended to be in the ≥ 65 years age group. Most study participants are expected to be enrolled in the United States (US). **Prioritization will be given to enrollment of individuals at high risk for COVID-19 by virtue of Black/African American or Native American race, Latinx ethnicity, co-morbid conditions (eg, obesity [BMI > 30 kg/m^2], chronic kidney or lung disease, cardiovascular disease and diabetes mellitus type 2) and life circumstances (living or working conditions involving known frequent exposure to SARS-CoV-2 or to densely populated circumstances [eg, factory or meat packing plants, essential retail workers, etc]).** (See Section 8.6 Recruitment and Retention for guidelines with respect to high-risk characteristics.) Consideration will be paid to the enrollment of participants for whom vaccines authorized for Emergency Use are not, or not anticipated to be, recommended or available during the early months of this trial.

In the **Pediatric Expansion**, participants will only be enrolled in the US. All efforts will be made to enroll similar numbers of participants in the subgroups 12 to < 15 years of age, and 15 to < 18 years of age. As with the **Adult Main Study**, an effort will be made to recruit adolescents of a diverse population including underserved minorities. Enrollment of the full adolescent cohort of participants (12 to < 18 years) will be contingent upon the review of early safety data (ie, 7 days of reactogenicity and overall safety post-dose 1) to be reviewed in the first ~60 enrolled adolescents (randomized in a 2:1 ratio to receive 5 µg SARS-CoV-2 rS adjuvanted with 50 µg Matrix-M1 or placebo) before enrollment of the remainder of the adolescent participants (N=~2,940). Likewise, administration of the second vaccine dose to the full participant population will be contingent upon the review of early safety data (ie, 7 days of reactogenicity and overall safety post-dose 2) in the first ~60 enrolled adolescents before dosing the remainder of the adolescent participants. Safety data will be provided to and reviewed by the Data and Safety Monitoring Board (DSMB) after each early safety data review period (ie 7 days after each vaccination in the sentinel group). Simultaneously, safety data will be reviewed internally by the Sponsor.

Trial Vaccinations

In both the **Adult Main Study** and **Pediatric Expansion**, study vaccination regimens will comprise 2 intramuscular (IM) injections (Day 0 and Day 21 + 7 days), with the trial vaccine assigned in a full dose injection volume of 0.5 mL. The dose level selected for evaluation is 5 µg SARS-CoV-2 rS adjuvanted with 50 µg Matrix-M1 based on optimal safety and immunogenicity observed in nonclinical and early clinical data. All vaccinations will be administered on an outpatient basis by qualified vaccine administrators in a way to maintain the blind as described in the Pharmacy Manual. Unblinded product will be managed by unblinded study site personnel who may administer study vaccine, if qualified to do so, but will not otherwise be involved in the study procedures or observations of participants.

Safety Assessments

In the **Adult Main Study**, following collection of sufficient safety data to support application for Emergency Use Authorization (EUA), participants will be scheduled for administration of 2 injections of the alternate study material 21 days apart ("blinded crossover"). That is, initial recipients of placebo will receive SARS-CoV-2 rS with Matrix-M1 adjuvant and initial recipients of SARS-CoV-2 rS with Matrix-M1 adjuvant will receive placebo. The same procedure for vaccine administration followed for the initial set of vaccinations will be followed at the time of the blinded crossover to ensure that the integrity of the blinded study is maintained.

In the **Pediatric Expansion**, following ~6 months of follow-up after the completion of the initial set of vaccinations (or earlier depending upon feasibility, EUA vaccine availability and the dynamics of the pandemic), pediatric participants will be scheduled for administration of 2 injections of the alternate study material 21 days apart ("blinded crossover"). That is, initial recipients of placebo will receive SARS-CoV-2 rS with Matrix-M1 adjuvant and initial recipients of active vaccine will receive placebo. The same procedure for vaccine administration followed for the initial set of vaccinations will be followed at the time of the blinded crossover to ensure that the integrity of the blinded study is maintained.

In both the **Adult Main Study** and **Pediatric Expansion**, solicited AEs of reactogenicity after the initial set of vaccinations will be collected via participant (**Adult Main Study**) or parent(s)/caregiver(s) (**Pediatric Expansion**) reporting in the eDiary utilizing a smartphone application. Participants or parent(s)/caregiver(s) who do not possess an appropriate device will be provided a device compatible with study requirements. All participants will be trained on the use of these applications at the initiation of their participation in the study (Day 0), and a Help Desk will be available 24 hours 7 days a week (24/7) for technical issues. For data entry issues, participants and parent(s)/caregiver(s) should contact the study site.

In the **Adult Main Study**, overall safety assessments, to be collected via the eDiary, from Day 0 through the first 12 months of follow-up post final after the initial set of vaccinations will include participant-recorded solicited (local and systemic reactogenicity) events through 7 days following each injection in the initial set of vaccinations. Unsolicited AEs and MAAEs will be collected through 49 days, ie, 28 days after second injection of the initial and crossover sets of vaccinations. MAAEs attributed to vaccine, AESIs, SAEs and investigator-assessed targeted physical examination findings, including vital sign measurements will be collected through Month 12. Safety follow-up phone calls will be conducted at 3 and 6 months (±30 days) post-crossover to collect MAAEs attributed to vaccine, AESIs, and SAEs in all participants that received crossover vaccinations. During the second 12 months of follow-up after the initial set of vaccinations, participants will be queried every 3 months via remote contacts (ie, Months 15 and 21) or during the scheduled visits (ie, Months 12, 18, and 24) for MAAEs attributed to study vaccine, AESIs and SAEs.

In the **Pediatric Expansion**, overall safety assessments from Day 0 through the first 12 months of follow-up after the initial set of vaccinations will include parent(s)/caregiver(s)-recorded solicited (local and systemic reactogenicity) events through 7 days following each injection in the initial set of vaccinations collected via eDiary. Unsolicited AEs and MAAEs will be collected through 49 days, ie, 28 days after second injection of the initial and crossover sets of vaccinations and MAAEs attributed to vaccine, AESIs, and SAEs will be recorded by the parent/caregiver on the memory aid and reported to the sites by weekly remote contact with the parent(s)/caregiver(s) through Month 12, and every 3 months via remote contacts (ie, Months 15 and 21) or during the scheduled visits (ie, Months 12, 18, and 24) during the second year (total of 24 months).

Immunogenicity Assessments

In the **Adult Main Study**, blood samples for serologic assessments (anti-NP antibodies, IgG antibody to SARS-CoV-2 S protein, MN, and hACE2 inhibition) will be collected from all participants before the first vaccination and at selected subsequent time points, including prior to the crossover set of vaccinations. Immune responses immediately following vaccinations will be obtained from all participants after the initial set of vaccinations but not after the crossover vaccinations. Testing will be performed on a subset of collected sera from the Immunogenicity Population of up to approximately 1,200 adult participants from the active and placebo treatment groups split approximately evenly across the 2 age categories designated at random by biostatisticians who are blinded to treatment assignment. Blood samples will be obtained approximately 14 days after the second crossover vaccination dose from approximately 900 participants 18 to ≤ 64 years of age at selected study sites to compare immunogenicity of the vaccine lot utilized for the crossover vaccination period to that of the earlier lot used for the initial vaccination period.

In the **Pediatric Expansion**, blood samples for serologic assessments (anti-NP antibodies, IgG antibody to SARS-CoV-2 S protein, MN, and hACE2 inhibition) will be collected from all pediatric participants before the initial set of vaccinations and at selected subsequent time points thereafter, including immediately prior to the crossover set of vaccinations. Immune responses immediately following vaccinations will be obtained from all participants after the initial set of vaccinations but not immediately after the crossover vaccinations (Visit C2). Persistence of the immune responses will be measured at 12, 18, and 24 months after the initial set of vaccinations. Testing will be performed on all adolescent participants. In summary, there are 7 scheduled blood draws in pediatric study participants for immunogenicity assessments at the Day 0, 21, 35, C1 (Day 180), and Months 12, 18, and 24 visits, and during unscheduled visits (ie Acute illness, Convalescent, and General). For participants in the CMI cohort only (N=50), additional blood will be collected to obtain PBMCs at the Day 0, 7 and 28 visits.

Both adult and pediatric participants who test positive for COVID-19 anti-NP antibodies and/or PCR-positive nasal swab at baseline indicating previous or current asymptomatic SARS-CoV-2 infection, will have subsequent SARS-CoV-2 S protein immune responses analyzed, but will not contribute to the primary efficacy/effectiveness

analyses. Those who test positive immediately prior to the crossover vaccination series may contribute to the immunogenicity analyses at Months 12, 18 and 24. Immediate immunogenicity analyses will not be performed subsequent to the crossover vaccination period. Results from the anti-NP positive and/or PCR-positive nasal swab participants prior to either set of vaccinations will be assessed and reported separately. In the **Adult Main Study**, whole blood samples for PBMC will be collected at Days 0, 21 and 35 from a small subset of participants (< 100) representing both age strata and reasonably reflecting the demographic subgroups enrolled at study site(s) with the capacity to isolate PBMCs. In the **Pediatric Expansion**, whole blood samples for PBMC will be collected at Days 0, 7 and 28 from a small subset comprised of 50 adolescent participants enrolled at selected study site(s) with the capacity to isolate PBMCs. These cells will be evaluated for cell-mediated immune responses to the initial set of vaccinations.

Prospective Surveillance of COVID-19

Prospective surveillance of COVID-19 will be conducted in the **Adult Main Study** and the **Pediatric Expansion**.

For prospective surveillance in the **Adult Main Study**, participants will be provided with an oral thermometer on Day 0 and instructed to monitor their body temperature daily throughout the first 12 months of the study and to record temperature and any other relevant symptoms daily in their eDiary (see Section 10.4.3 for details). Participants who are noted during regular monitoring of the eDiary entries to not have reported temperature and symptoms for ≥ 7 days will be contacted by phone to assess clinical status and maintain engagement in the study.

Starting on Day 4, throughout the first 12 months of the study, when fever or other specified symptoms (see Table 5 for symptoms suggestive of COVID-19) are reported in the eDiary for at least 2 consecutive days for the same symptom, participants will be directed via the eDiary to begin nasal self-swabbing for PCR testing within 3 days of symptom onset at home for a total of 3 days and to initiate daily completion of the InFLUenza Patient-Reported Outcome (FLU-PRO) symptom reporting instrument for 10 days after COVID-19 symptom onset or until the participant experiences 2 consecutive asymptomatic days. Participants will be instructed at their enrollment visit on the methods of nasal self-swabbing for COVID-19 and completion of the FLU-PRO symptom reporting instrument. In addition, the eDiary will alert the study site to contact the participant to schedule the in-person Unscheduled Acute Illness Visit. During the first 4 days after the second vaccination when solicited systemic reactogenicity symptoms may be similar to those of COVID-19, investigators should use their clinical judgement to decide if an Unscheduled Acute Illness visit is warranted. Active surveillance for COVID-19 will continue after the blinded crossover through the first 12 months of study. Passive surveillance of safety and efficacy via remote contacts or the scheduled visits will continue during Months 12 to 24.

After the first day of home nasal swabbing, repeat nasal self-swabs should be obtained daily for a total of 3 days to ensure capture of intermittent shedding. The self-swabs obtained by the participant should be maintained according to directions provided in the 3-swab kit, and the designated courier should be contacted to pick up the kit for shipping to the central lab, as directed.

At the in-person Unscheduled Acute Illness Visit, participants will be queried regarding AE symptoms, concomitant medications taken for these symptoms, undergo a targeted physical examination (to include oxygen [O_2] saturation and respiratory rate), as indicated by the participant's signs and symptoms, and have obtained by the study personnel a medically attended nasal swab, a blood sample for serologic testing and be trained on the use of the portable pulse oximeter that they will take home with them. Medically attended swabs collected at the Unscheduled Acute Illness Visit will be processed at the study site for shipment to the central laboratory according to established procedures as described in the Laboratory Manual. Other clinical or laboratory evaluations that may be performed at the discretion of study personnel to inform need for isolation or additional medical care will be outside the scope of the study protocol.

Completion of the FLU-PRO reporting instrument and O_2 saturation (at rest and following mild exercise, defined as walking around the room for 1 minute) will be captured daily in the eDiary for 10 days after COVID-19 symptom onset or until the participant experiences 2 consecutive asymptomatic days.

Study participants whose home nasal self-swab and/or medically attended nasal swabs are confirmed at the central laboratory to be PCR-positive for SARS-CoV-2 at the Unscheduled Acute Illness Visit will be contacted by the study site to arrange an Unscheduled Convalescent Visit. The Unscheduled Convalescent Visit will occur approximately 1 month (or as soon thereafter, as feasible) after the onset of the PCR-confirmed case of COVID-

19 at the Unscheduled Acute Illness Visit to assess status of AEs, record the clinical course of the disease on the Endpoint Form and obtain a blood sample for convalescent serologic testing. Pulse oximeters should be returned to the study site at this visit.

For the **Pediatric Expansion**, starting on Day 4, throughout the first 12 months of the study, parent(s)/caregiver(s) of participants will be asked to report symptoms of COVID-19 to the site as soon as possible after symptoms onset, or during the weekly remote contact. Fever and other symptoms of COVID-19 (including date of onset, duration, etc.) will be collected in a paper memory aid and will be reported to the sites by the parent(s)/caregiver(s) either as a spontaneous phone call or during the weekly remote contact during the first 12 months. If the parent(s)/caregiver(s) report symptoms compatible with COVID-19 by spontaneous contact or during the weekly contact, the study site will schedule an in-person Unscheduled Acute Illness Visit. During the first 4 days after vaccination when solicited systemic reactogenicity symptoms may be similar to those of COVID-19, investigators should use their clinical judgement to decide if an Unscheduled Acute Illness visit is warranted. Active surveillance for COVID-19 will continue after the blinded crossover through the first 12 months of study. Passive surveillance of safety and efficacy via remote contacts or the scheduled visits will continue during Months 12 to 24. An eDiary will be used for the collection of 7-day reactogenicity data after each initial vaccination, however, it will not include a prompt to complete the FLU-PRO instrument or prompts for self-swabbing as these are not collected for the adolescent participants. An eDiary collection of reactogenicity will not be performed after crossover vaccinations. Prospective surveillance will continue via weekly phone calls after blinded crossover until Month 12, and will be conducted at 3 and 6 months (±30 days) post-crossover from Month 12 to Month 24.

At the in-person Unscheduled Acute Illness Visit, participants will be queried regarding AE symptoms, concomitant medications taken for these symptoms, undergo a targeted physical examination (to include oxygen [O_2] saturation and respiratory rate), as indicated by the participant's signs and symptoms, and have obtained by the study personnel a medically-attended nasal swab, and a blood sample for serologic testing. No portable pulse oximeter will be distributed for home use for adolescent participants and there will be no self-swab collection in the adolescent participants. Medically attended swabs collected at the Unscheduled Acute Illness Visit will be processed at the study site for shipment to the central laboratory according to established procedures as described in the Laboratory Manual. Other clinical or laboratory evaluations that may be performed at the discretion of study personnel to inform need for isolation or additional medical care will be outside the scope of the study protocol.

Study participants whose medically-attended nasal swabs are confirmed at the central laboratory to be PCR-positive for SARS-CoV-2 at the Unscheduled Acute Illness Visit will be contacted by the study site to arrange an Unscheduled Convalescent Visit. The Unscheduled Convalescent Visit will occur approximately 1 month (or as soon thereafter, as feasible) after the onset of the PCR-confirmed case of COVID-19 at the Unscheduled Acute Illness Visit to assess status of AEs, record the clinical course of the disease on the Endpoint Form and obtain a blood sample for convalescent serologic testing.

Timing of Trial Visits

The **Adult Main Study** will consist of a screening period (up to 30 days prior to Day 0); initial vaccination days (Days 0 and 21 + 7 days); and outpatient study visits on Days 0, 21 (+ 7 days), and 35 (+ 7 days) in the initial set of vaccinations. Additional subsequent study visits for blood draws and the crossover set of vaccinations (approximately 21 days apart) will occur after acquisition of safety data on SARS-CoV-2 rS with Matrix-M1 adjuvant sufficient to support application for Emergency Use Authorization (EUA) in adults.

Adult participants who experience a PCR+ episode of COVID-19, regardless of whether their illness represents an endpoint, will be eligible to receive both the initial and crossover sets of vaccinations if they remain in blinded follow-up. Study visits subsequent to the crossover scheduled at Months 12 (± 15 days), 18 (± 30 days) and 24 (± 30 days) after the initial set of vaccinations will be performed to assess the durability of immune response, taking into account when participants received active vaccine (initial or crossover).

In addition to the aforementioned Unscheduled Acute Illness and Unscheduled Convalescent Visits in the **Adult Main Study**, an Unscheduled General Visit may be conducted by study personnel in the event of a general medical issue other than COVID-19 symptomatology, eg, reconsent participants to amended Consent Form, if needed, and remote contact for safety follow-up may be conducted approximately every 3 months after crossover if no other visits have been required. An EoS visit will be recorded for all study participants at approximately 24 months (± 30 days) after their initial set of vaccinations or at their last visit on study. Should participants decide to terminate

early, an EoS telephone or in-person visit will occur to collect the maximum safety data and blood sample, if possible. All study participants who choose to be unblinded or to withdraw from active surveillance will be encouraged to continue in follow-up for safety and reported COVID-19 cases via remote contacts with investigative site personnel.

Participants that requested to be unblinded and/or are continuing in the study for Safety Follow-up will have visits replaced by remote contact (phone, email, text), at a minimum, every 90 days (± 30 days) from last dose received during the first year of participation. During the second year (ie, Months 12 to 24), contact will be continued every 180 days (± 30 days). **No eDiary, nasal swabs or blood samples will be collected during safety follow-up.** MAAEs, SAEs, AESI, and COVID-19 diagnoses will be collected during the remote contacts at the time points specified in Table 1. Receipt of another COVID-19 vaccine used under EUA should be recorded as a concomitant medication. The End of Study page is not to be completed for these participants until the end of their participation in the study.

The **Pediatric Expansion** will consist of a screening period (up to 7 days prior to Day 0); initial vaccination days (Days 0 and 21 + 7 days); and outpatient study visits on Days 0, 21 (+ 7 days), and 35 (+ 7 days) in the initial set of vaccinations. A sentinel group of ~60 adolescents (15 to < 18 years of age, N=~30, and 12 to < 15 years of age, N=~30 [randomized in a 2:1 ratio to receive 5 µg SARS-CoV-2 rS adjuvanted with 50 µg Matrix-M1 or placebo]) will be enrolled first and will be followed for 7 days after the first vaccine dose for assessment of reactogenicity and overall safety, which will be reviewed prior to enrollment of the remaining ~2,940 adolescents. Similarly, 7 days of reactogenicity and overall safety data following the receipt of the second vaccine dose in the sentinel group will be reviewed before the second vaccine dose is administered to the rest of the adolescent participants. In-person review of safety and/or blood collection for immunogenicity will be supplemented by remote contact (phone, text, email) on Days 7 (+3 days), 14 (+ 3 days), 28 (+3 days), 42 (+3 days) and 49 (+ 7 days) in the initial set of vaccinations. The blinded crossover visits for pediatric participants will begin ~6 months after the last vaccination of the initial set of vaccinations (or earlier depending upon feasibility, EUA vaccine availability for this age group, and the dynamics of the pandemic). All participants who remain in blinded follow-up at the time of the blinded crossover will be eligible for crossover vaccination, regardless of whether they have experienced a COVID-19 diagnosis. Subsequent visits at Months 12 (± 15 days), 18 (± 30 days) and 24 (± 30 days) after the initial set of vaccinations will be performed to assess the durability of immune response, taking into account when participants received active vaccine (initial or crossover).

In addition to the aforementioned Unscheduled Acute Illness and Unscheduled Convalescent Visits in the **Pediatric Expansion**, an Unscheduled General Visit may be conducted by study personnel in the event of a general medical issue other than COVID-19 symptomatology, eg, reconsent to an amended Consent Form, if needed. An EoS visit will be recorded for all study participants at approximately 24 months (± 30 days) after their initial set of vaccinations or at their last visit on study. Should participants decide to terminate early, an EoS telephone or in-person visit will occur to collect the maximum safety data and blood sample, if possible. All study participants who choose to be unblinded or to withdraw from active surveillance will be encouraged to continue in follow-up for safety and reported COVID-19 cases via remote contacts with investigative site personnel.

Participants that requested to be unblinded and/or are continuing in the study for Safety Follow-up will have visits replaced by remote contact (phone, email, text), at a minimum, every 90 days (± 30 days) from last dose received during the first year of participation. During the second year (ie, Months 12 to 24), contact will be continued every 180 days (± 30 days). **No eDiary, nasal swabs or blood samples will be collected during safety follow-up.** MAAEs, SAEs, AESI, and COVID-19 diagnoses will be collected during the remote contacts at the same time points described in Table 1 for scheduled visits in the participants that did not request unblinding. Receipt of another COVID-19 vaccine used under EUA should be recorded as a concomitant medication. The End of Study page is not to be completed for these participants until the end of their participation in the study.

Safety Monitoring

This protocol has extensive safety monitoring in place. Safety is monitored routinely by the ICON Medical Monitor, Novavax Pharmacovigilance and Safety Surveillance Physicians, Novavax Clinical Development Leads and routinely by the 2019nCoV-301 Protocol Safety Review Team (PSRT) (for **Adult Main Study** only). In the **Adult Main Study** and **Pediatric Expansion**, a centralized DSMB will be established in collaboration with NIH, NIAID, Biomedical Advanced Research and Development Authority (BARDA) and Novavax according to the

charter dictated by the participating groups. This group will review interim unblinded data periodically in the **Adult Main Study and Pediatric Expansion** and make recommendations with respect to safety and emerging efficacy.

The short-term safety of the adolescent cohort (ie, analysis of 7-day reactogenicity and safety in the first ~60 participants enrolled in an approximately even distribution across age groups 15 to < 18 years and 12 to < 15 years before enrollment of the remainder of the adolescent cohort) will be reviewed by the DSMB. Likewise, analysis of 7-day reactogenicity and safety after receipt of the second vaccine dose in the first ~60 participants enrolled will be reviewed by the DSMB before the second vaccine dose is administered to the remainder of the adolescent participants. The safety data will be simultaneously shared and reviewed internally by the Sponsor.

Finally, the DSMB may recommend that the trial be terminated or that specific groups be withdrawn from the study, if any subgroup manifests serious or widespread adverse side effects. The DSMB will be informed immediately by the ICON unblinded statistician if the pre-specified stopping boundary is met in the **Adult Main Study**, indicating that the vaccine causes harm by increasing the rate of mild, moderate or severe COVID-19. In addition, the DSMB will monitor the study for high vaccine efficacy or for futility to detect vaccine activity.

Study Vaccination Pause Rules (Pediatric Expansion)

AEs meeting any one of the following criteria will result in a pause being placed on subsequent vaccinations pending further review by the Novavax internal Safety Review Team (SRT). AEs meeting the criteria below will be forwarded to the DSMB Chair for review:

- Any SAE attributed to vaccine based on Sponsor assessment within the first 7 days following the first or second initial vaccination in the first 60 adolescent participants.
- Any toxicity grade 3 or higher (severe or life-threatening) single solicited (local or systemic) AE term occurring in ≥10% of the first 60 participants (≥6 participants) within the first 7 days following the first or second initial vaccination.
- Any grade 3 (severe) unsolicited single AE preferred term for which the investigator assesses as related occurring in ≥5% of the first 60 participants (≥3 participants) within the first 7 days following the first or second initial vaccination.

In addition, any SAE assessed as related to vaccine (final assessment by the Sponsor) will be reported by the Sponsor to the DSMB Chair as soon as possible, and within 24 hours of the sponsor’s assessment of the relatedness of the event. Based on the initial report of the event to the DSMB Chair, the Chair may convene an ad hoc meeting of the Board or make alternative recommendations to the Sponsor with respect to the conduct of the study. The DSMB Charter defines processes for how this review will occur and how the Chair’s recommendations will be documented.

The Sponsor, along with medical monitor, may request a DSMB review for any safety concerns that may arise in the Pediatric Expansion of the study, even if they are not associated with any specific pause rule.

Participant Retention

Because the blinded crossover will provide all study participants with active vaccine, either initially or at the time of blinded crossover, it is anticipated that study participants and parent(s)/caregiver(s) will be incentivized to continue participation in the blinded study. Participants unblinded between the first (Day 0) and second (Day 21) dose of trial vaccine will not be eligible to receive further investigational product on this protocol. Similarly, participants who choose to withdraw from the blinded trial after having received both doses of blinded study vaccine will not be eligible for blinded crossover. Safety follow-up of these participants will include remote contacts (eg, phone, email or text) to collect SAE, MAAE, AESI and COVID-19 diagnoses at the time points indicated on the Schedule of Events [see Table 1 for the **Adult Main Study** and Table 2 for the **Pediatric Expansion**] or at regular intervals if visits are not mandated. Receipt of another COVID-19 vaccine under EUA and date of administration should be recorded as a concomitant medication. Participants whom, upon unblinding for the purpose of receiving EUA-authorized vaccine, learn they had received active vaccine will be censored at the time of unblinding. Adult participants who are unblinded after 26 January 2021 are not eligible to receive further investigational product on this protocol. Similarly, participants who choose to withdraw from the blinded trial after having received both doses of blinded study vaccine will not be eligible for blinded crossover. Safety

follow-up of these participants will include remote contacts (eg, phone, email or text) to collect SAE, MAAE, AESI and COVID-19 diagnoses at the time points indicated for scheduled visits in the participants that did not request unblinding (see Table 1) or at regular intervals if visits are not mandated. No eDiary, nasal swabs or blood samples for immunogenicity will be obtained.

Participants in the **Pediatric Expansion** who are unblinded between the first (Day 0) and second (Day 21) dose of trial vaccine will not be eligible to receive further investigational product on this protocol. Similarly, participants who choose to withdraw from the blinded trial after having received both doses of blinded study vaccine will not be eligible for blinded crossover. Safety follow-up of these participants will include remote contacts (eg, phone, email or text) to collect SAE, MAAE, AESI and COVID-19 diagnoses at the time points indicated [see Table 2 for the **Pediatric Expansion**] or at regular intervals if visits are not mandated. Receipt of another COVID-19 vaccine under EUA and date of administration should be recorded as a concomitant medication.

Number of Participants:

The **Adult Main Study** is planned to enroll up to approximately 30,000 participants who will be randomized initially 2:1 to receive active vaccine or placebo. The **Pediatric Expansion** is planned to enroll up to approximately 3,000 adolescent participants 12 to < 18 years of age who will be randomized initially 2:1 to receive active vaccine or placebo.

Diagnosis and Main Criteria for Inclusion/Exclusion:

Inclusion Criteria

Each participant in the **Adult Main Study** must meet all of the following criteria to be enrolled in this study:

1. Adults ≥ 18 years of age at screening who, by virtue of age, race, ethnicity or life circumstances, are considered at substantial risk of exposure to and infection with SARS-CoV-2. (See Section 8.6, Recruitment and Retention for guidelines with respect to high-risk characteristics.)
2. Willing and able to give informed consent prior to study enrollment and to comply with study procedures.
3. Participants of childbearing potential (defined as any participant who has experienced menarche and who is NOT surgically sterile [ie, hysterectomy, bilateral tubal ligation, or bilateral oophorectomy] or postmenopausal [defined as amenorrhea at least 12 consecutive months]) must agree to be heterosexually inactive from at least 28 days prior to enrollment and through 3 months after the last vaccination OR agree to consistently use a medically acceptable method of contraception from at least 28 days prior to enrollment and through 3 months after the last vaccination.
4. Is medically stable, as determined by the investigator (based on review of health status, vital signs [to include body temperature], medical history, and targeted physical examination [to include body weight]). Vital signs must be within medically acceptable ranges prior to the first vaccination.
5. Agree to not participate in another SARS-CoV-2 prevention trial during the study follow-up.

Each participant in the **Pediatric Expansion** must meet all of the following criteria to be enrolled in this study:

1. Pediatric participants 12 to < 18 years of age at screening, determined to be healthy or medically stable by the investigator (based on review of health status, vital signs [to include body temperature], medical history, and targeted physical examination [to include body weight]). Vital signs must be within medically acceptable ranges prior to the first vaccination.
2. Participant and parent(s)/caregiver(s) or legally acceptable representative willing and able to give informed consent and assent, as required, prior to study enrollment and to comply with study procedures.
3. Participants of childbearing potential (defined as any participant who has experienced menarche and who is NOT surgically sterile [ie, hysterectomy, bilateral tubal ligation, or bilateral oophorectomy] must agree to be heterosexually inactive from at least 28 days prior to enrollment and through 3 months after the last vaccination OR agree to consistently use a medically acceptable method of contraception from at least 28 days prior to enrollment and through 3 months after the last vaccination.
4. Agree to not participate in another SARS-CoV-2 prevention trial during the study follow-up.

Exclusion Criteria

Adult and pediatric participants meeting any of the following criteria will be excluded from the study:

1. Unstable acute or chronic illness. Criteria for unstable medical conditions include:

a. Substantive changes in chronic prescribed medication (change in class or significant change in dose) in the past 2 months.
b. Currently undergoing workup of undiagnosed illness that could lead to diagnosis of a new condition.

Note: Well-controlled human immunodeficiency virus [HIV] with undetectable HIV ribonucleic acid [RNA < 50 copies/mL] and CD4 count > 200 cells/μL for at least 1 year, documented within the last 6 months, is NOT considered an unstable chronic illness. Participant's or parent's/caregiver's verbal report will suffice as documentation.

2. Participation in research involving an investigational product (drug/biologic/device) administered within 45 days prior to first study vaccination.
3. History of a previous laboratory-confirmed diagnosis of SARS-CoV-2 infection or COVID-19.
4. Received any vaccine within 4 days prior to first study vaccination or planned receipt of any vaccine before Day 49 (ie, 28 days after second vaccination), except for influenza vaccination, which may be received ≥ 4 days prior to or ≥ 7 days after either study vaccination.
5. Autoimmune or immunodeficiency disease/condition (iatrogenic or congenital) or ongoing therapy that causes clinically significant immunosuppression.
   NOTE: Stable endocrine disorders (eg, thyroiditis, pancreatitis), including stable diabetes mellitus with no history of diabetic ketoacidosis) are NOT excluded.
6. Chronic administration (defined as > 14 continuous days) of immunosuppressant or systemic glucocorticoids, within 90 days prior to first study vaccination.
   NOTE: An immunosuppressant dose of glucocorticoid is defined as a systemic dose ≥ 20 mg of prednisone per day or equivalent. The use of topical, inhaled, and nasal glucocorticoids is permitted. Topical tacrolimus and ocular cyclosporin are permitted.
7. Received immunoglobulin or blood-derived products within 90 days prior to first study vaccination.
8. Active cancer (malignancy) on chemotherapy that is judged to cause significant immunocompromise within 1 year prior to first study vaccination (with the exception of malignancy cured via excision, at the discretion of the investigator).
9. Any known allergies to products contained in the investigational product.
10. Participants who are breastfeeding, pregnant or who plan to become pregnant within 3 months following last study vaccination.
11. Any other condition that, in the opinion of the investigator, would pose a health risk to the participant if enrolled or could interfere with evaluation of the trial vaccine or interpretation of study results.
12. Study team member or first-degree relative of any study team member (inclusive of Sponsor, and study site personnel involved in the study).
13. Current participation in any other COVID-19 prevention clinical trial.

Test Product, Dose and Mode of Administration:

The following supplies will be used for vaccination in the study:

- SARS-CoV-2 rS (5 µg) + Matrix-M1 adjuvant (50 µg)

Study vaccination regimens will comprise 2 IM injections (Day 0 and Day 21 + 7 days), with the trial vaccine assigned in a full dose injection volume of 0.5 mL.

- In the event of an error in administration of the wrong study material for the first vaccine injection, the participant will be administered the same study material for the second vaccine and will be analyzed in the treatment group received rather than the group to which they were randomized. Participants should then receive the originally randomized material at the time of blinded crossover. A participant who receives the "wrong" study material at the second injection **inadvertently** must be unblinded to offer a third dose ensuring that the participant receives at least 2 doses of the NVX-CoV2373 vaccine or to receive an EUA vaccine. In either situation, a protocol deviation must be reported.
- At the time of the blinded crossover, participants will receive the alternate study material in 2 IM injections 21 + 7 days apart.

Reference Therapy, Dose and Duration of Administration:

Placebo (normal saline, 0.5 mL) will be provided by the Sponsor.

Duration of Treatment:

The duration of the study, excluding screening, is approximately 24 months after the initial set of vaccinations for each participant.

Statistical Methods and Sample Size Calculation:

The sample size for the original design in the **Adult Main Study** was driven by the total number of cases expected to achieve statistical significance for the primary efficacy endpoint; a total of up to approximately 30,000 participants ≥ 18 years of age would be enrolled to provide a target of 144 symptomatic COVID-19 illness PCR-confirmed SARS-CoV-2 infections. With the current change of the study design to perform a single efficacy analysis for the initial vaccination phase when the blinded crossover is implemented, the estimated (through simulations) powers to reject the null hypothesis of VE lower bound of 95% CI ≤ 30% and achieving the point estimate of VE ≥ 50% simultaneously for the primary endpoint is summarized in **Error! Reference source not found.** in Section 12.1.
The analyses for the **Adult Main Study** will be performed separately from analyses for the **Pediatric Expansion**. Only participants ≥ 18 years of age at the time of randomization will be included for analyses of data collected in the **Adult Main Study**.

The sample size in the **Pediatric Expansion** is chosen to provide an adequate safety database of ≥ 2,000 pediatric recipients of investigational product to support licensure of SARS-CoV-2 rS with Matrix-M1 adjuvant in pediatric participants 12 to < 18 years of age. Recruitment of study participants will attempt to enroll a similar number of participants in the 12 to < 15 and 15 to < 18 year old age groups. The enrollment of approximately 3,000 adolescent participants with a 2:1 randomization to active vaccine or placebo will provide a total of approximately 2,000 pediatric participants exposed to active vaccine.

The analysis of efficacy in the **Pediatric Expansion** will be descriptive in nature using the same methods as the **Adult Main Study** but with no formal statistical hypothesis tested. The estimated (through simulations) powers to demonstrate VE ≥ 0% are presented in Table 10 in Section 12.1.

For the effectiveness endpoint, successful demonstration of non-inferiority will require meeting the following 3 pre-specified criteria simultaneously,

1. upper bound of 2-sided 95% CI for the ratio of GMTs ($GMT_{18\text{-}<26yo}/GMT_{12\text{-}<18yo}$) < 1.5,
2. point estimate of the ratio of GMTs ≤ 1.22 (estimated as square root of 1.5)
3. upper bound of the 2-sided 95% CI for difference of seroresponse rates ($SRR_{18\text{-}<26yo} - SRR_{12\text{-}<18yo}$) < 10%

With 400 evaluable participants (500 accounting for 20% non-evaluability) in the active vaccine group randomly selected from each of the 18 to < 26 years of age subset of participants in the **Adult Main Study** and the **Pediatric Expansion**, there is over 85% power (through simulations) to demonstrate the first 2 non-inferiority criteria when assuming an underlying GMT for the 18 to < 26 years of age group up to 1.1-fold higher than the 12 to < 18 years of age group. In the absence of an established correlate of protection for SARS-CoV-2 vaccines, seroresponse will be defined as ≥ 4-fold increase in neutralization titers (MN_{50}) at Day 35 relative to baseline titers. With this definition and assumed seroresponse rates (SRR) of 95% in the 18 to < 26 years of age group, there is over 80% power to demonstrate the third non-inferiority criterion for a difference as large as 4% lower in the 12 to < 18 years of age group. A descriptive assessment of immunogenicity will evaluate the same criteria in the 12 to < 15 and 15 to < 18 years of age groups separately.

With approximately 2,000 participants in the active vaccine group, there is a >90% probability of observing at least 1 participant with an AE if the true incidence of the AE is 0.12% and a 99% probability if the true incidence of the AE is 0.23%. With 2,000 participants receiving at least 1 vaccine dose, there is a >99% probability of observing at least 1 participant with an AE if the true incidence of the AE is at least 0.12%.

The blinded crossover at ~6 months after completion of the initial set of vaccination and 12 months follow-up after the crossover set of vaccinations will aim to collect ≥ 12 months safety data after receipt of the active adjuvanted vaccine. The sample size will provide more than adequate numbers to support the analyses of immune response that are anticipated to support effectiveness.

The following analysis sets are identified for analysis:

Intent-to-Treat Efficacy (ITT) Analysis Set

In the **Adult Main Study** and **Pediatric Expansion**, the ITT analysis sets will include all participants who are randomized, regardless of protocol violations or missing data. The ITT analysis set will be used for participant disposition summaries and will be analyzed according to the treatment arm to which the participant was randomized.

Full Analysis Set (FAS)

In the **Adult Main Study** and **Pediatric Expansion**, the FAS will include all participants who are randomized and received at least 1 dose of study vaccine/placebo, regardless of protocol violations or missing data. Participants who are unblinded with an intention to receive other COVID-19 vaccines will be censored at the time of unblinding. The FAS population will be analyzed according to the treatment group to which they were randomized. The FAS analysis sets will be used for supportive analyses.

Safety Analysis Set

In the **Adult Main Study** and **Pediatric Expansion**, the safety analysis set will include all participants who receive at least 1 dose of trial vaccine. Participants in the safety analysis set will be analyzed according to the vaccine actually received. In cases where information is available that indicates that a participant received both active and placebo, the participant will be analyzed as part of the active group. Participants who unblind with intention to receive another COVID-19 vaccine (under EUA or otherwise licensed) or after unblinding due to dosing error will be censored at the time of unblinding.

Per-Protocol Efficacy (PP-EFF) Analysis Set

In the **Adult Main Study** and **Pediatric Expansion**, the PP-EFF analysis sets (for each age stratum) will include all participants who receive the full prescribed regimen of trial vaccine and have no major protocol deviations that occur before the first COVID-19 PCR-positive episode (ie, participant will be censored at the time the protocol deviation) and are determined to affect the efficacy outcomes, including baseline SARS-CoV-2 seropositivity or nasal swab PCR-positivity. Participants who are unblinded with an intention to receive other COVID-19 vaccines will be censored at the time of unblinding. Although the study will enroll participants regardless of SARS-CoV-2 serologic status at the time of initial vaccination, any participants with confirmed infection or prior infection due to SARS-CoV-2 at baseline, by nasal swab PCR or serology, will be excluded from the PP-EFF population. PP-EFF will be the primary set for all efficacy endpoints. Participants determined to have positive nasal swab PCR or serology immediately prior to the first crossover vaccination will be excluded from the post-crossover PP-EFF population. A second per-protocol efficacy (PP-EFF-2) analysis set is defined to allow for evaluation of baseline serostatus analysis's impact on vaccine efficacy. The PP-EFF-2 analysis set will follow the same method described in the PP-EFF population with the exception that it will include all participants regardless of baseline serostatus.

The review and determination for exclusion from the PP-EFF and PP-EFF-2 analysis sets will be carried out in a blinded fashion by a study clinician based on all available information prior to unblinding.

Per-Protocol Immunogenicity (PP-IMM) Analysis Set

In the **Adult Main Study** and **Pediatric Expansion**, the PP-IMM analysis set will be determined for each study visit. Immediate immune reaction to study vaccine will be evaluated after the initial set of vaccinations. The PP-IMM analysis set will include participants that have at least a baseline and 1 serum sample result available after vaccination and have no major protocol violations that are considered clinically relevant to impact immune response at the corresponding study visit, including nasal PCR-positive swabs or seropositivity for SARS-CoV-2 prior to the visit in question. All participants in the PP-IMM analysis populations will be designated at time of vaccination within the immunogenicity subset. For participant visits on or after Day 21, participants must receive the second vaccination to be included in the PP-IMM analysis set. Prior exposed participants will be determined using baseline SARS-CoV-2 nasal swab or seropositivity at screening to assess if immune responses differ between previously exposed and unexposed individuals. Durability of immune responses will be evaluated in participants who provide serologic data at Months 12, 18 and 24, taking into account when they received active vaccine and if/when they were infected with SARS-CoV-2, based on PCR or serology.

Adult participants in the PP-IMM population for the immunobridging analysis will be those at selected study sites who receive the 2 crossover vaccination doses and have blood drawn for anti-S IgG antibodies immediately prior to the first crossover vaccination dose and approximately 14 days after the second crossover vaccination dose.

The review and determination for exclusion from the PP-IMM analysis set will be carried out in a blinded fashion by a study clinician based on all available information prior to unblinding.

Primary Endpoint

Adult Main Study

The primary endpoint will be analyzed on the PP-EFF analysis set and supported by analysis of the FAS analysis set.

The VE is defined as VE (%) = (1 – RR) x 100, where RR = relative risk of incidence rates between the 2 trial vaccine groups (SARS-CoV-2 rS / Placebo). The RR will be estimated by exponentiating the treatment group coefficient from a Poisson regression analysis with robust error variance [Zou 2004]. The age strata will be included in the model as a covariate. To assess incidence rates rather than absolute counts of cases, accounting for differences in follow-up times starting with 7 days after the second vaccination among participants, an offset will be utilized in the Poisson regression. A two-sided, 95% confidence interval (CI) will be constructed around the estimate.

A super superiority of the VE at each analysis will be used to determine success of the primary endpoint. A hypothesis test with a one-sided Type I error of 2.5% will be constructed with the following hypotheses:

H_0: VE ≤ 0.30 (RR ≥ 0.70)

H_1: VE > 0.30 (RR < 0.70)

Rejection of the null hypothesis demonstrates a statistically significant VE with a lower bound of CI > 30%. In order to be considered for EUA or licensure by the United States Food and Drug Administration (FDA), a vaccine must show super superiority where there is a minimum VE of 50% and a lower bound of two-sided 95% confidence bound of at least 30%. Based upon the number of primary efficacy endpoints planned for analysis, a lower bound of more than 30% corresponds with a VE point estimate of at least 50%.

Pediatric Expansion

Efficacy

The primary endpoint will be analyzed on the PP-EFF analysis set and supported by analysis of the FAS analysis set. The analysis will be descriptive in nature with no formal statistical hypothesis tested.

The VE is defined as VE (%) = (1 – RR) x 100, where RR = relative risk of incidence rates between the 2 trial vaccine groups (SARS-CoV-2 rS / Placebo). The RR will be estimated by exponentiating the treatment group coefficient from a Poisson regression analysis with robust error variance [Zou 2004]. To assess incidence rates rather than absolute counts of cases, accounting for differences in follow-up times starting with 7 days after the second vaccination among participants, an offset will be utilized in the Poisson regression. A two-sided, 95% confidence interval (CI) will be constructed around the estimate.

Safety

Accumulating safety data, blinded to treatment group, will be reviewed routinely by the ICON Medical Monitor, Novavax Pharmacovigilance and Safety Surveillance Physicians, and Novavax Clinical Development Leads to detect possible signals of a concerning frequency or severity of solicited (following the initial set of vaccinations only) or unsolicited AEs that may require escalation to the DSMB for unblinded review.

In formal analyses, numbers and percentages (with 95% CIs based on the Clopper-Pearson method) of participants with solicited local and systemic AEs through 7 days after each vaccination of the initial set of vaccinations will be summarized by trial vaccine group and the maximum toxicity grade over 7 days after each vaccination. The duration of solicited local and systemic AEs after each vaccination will also be summarized by trial vaccine group.

Reactogenicity will not be collected following the crossover set of vaccinations, except for immediate reactogenicity (ie, 30 minutes after each of the crossover vaccinations).

Unsolicited AEs will be coded by preferred term and system organ class using the latest version of the Medical Dictionary for Regulatory Activities (MedDRA) and summarized by age cohort and trial vaccine group as well as by severity and relationship to trial vaccine. All AEs through 28 days after second vaccination of each set of vaccinations (initial and crossover); all MAAEs related to vaccine, SAE, or AESI through EoS will be listed separately and summarized by age cohort and trial vaccine group. Participants who are unblinded to treatment assignment will be requested to report SAE, MAAE, AESIs and COVID-19 diagnoses by remote contact at the remaining prespecified time points following unblinding (see Table 2).

Effectiveness

Formal non-randomized non-inferiority (NI) analysis of the primary effectiveness endpoint, neutralizing antibody to SARS-CoV-2 at Day 35, will be carried out using the PP-IMM analysis set. The analysis of the 2 pre-specified NI success criteria for ratio of GMTs will be performed using the point estimate and upper bound of the two-sided 95% confidence interval (CI) on the ratio of GMTs between 2 age cohorts (adults 18 to < 26 year old cohort in the **Adult Main Study**/adolescent cohort in the **Pediatric Expansion**). The ratio of GMTs between 2 age cohorts and the corresponding two-sided 95% CI will be calculated on log-transformed titers using an analysis of covariance (ANCOVA) with treatment group and baseline (Day 0) measurement as the covariate. The evaluation of the pre-specified NI success criterion for difference of seroresponse rates between 2 age cohorts (adolescents minus adults 18 to < 26 year old will be based on a 95% CI constructed with the method of Miettinen and Nurminen.

Secondary Endpoints

Adult Main Study and Pediatric Expansion

The key secondary efficacy endpoint (**Adult Main Study** only) and other secondary efficacy endpoints will be analyzed using the same manner as the primary efficacy analysis. The analysis of the key secondary endpoint will be carried out using a one-sided alpha of 0.025 only after the successful demonstration the primary endpoint to preserve the Type I error rate. All remaining secondary efficacy endpoints will also be performed using an unadjusted two-sided 0.05 alpha level.

For the moderate-to-severe efficacy endpoint, severe cases will be counted from potentially severe cases reviewed and confirmed by an external endpoint committee in a blinded manner. Details of this endpoint committee will be in the Statistical Analysis Plan.

In the **Pediatric Expansion**, analyses of secondary efficacy endpoints are descriptive in nature with no formal statistical hypothesis testing.

The secondary immunogenicity analyses will be performed using the PP-IMM analysis set and the FAS.

For the serum antibody levels specific for the SARS-CoV-2 S protein antigen(s) (IgG antibody to SARS-CoV-2 S protein and hACE2 inhibition) and MN, the geometric mean at each study visit, the geometric mean fold rise (GMFR) comparing to the baseline (Day 0) at each post-vaccination study visit, and the GMFR comparing pre- and post-second dose, along with 95% CI will be summarized by trial vaccine group. The 95% CI will be calculated based on the t distribution of the log-transformed values for geometric means or GMFR, then back transformed to the original scale for presentation. Seroresponse rate (SRR) will also be calculated with exact 95% CI. Seroresponse is defined as achieving a 4-fold rise in antibody response from baseline.

For the immunobridging analysis in the Adult Main Study comparing the initial and crossover lots of vaccine, the ratio of geometric mean IgG antibody concentrations will be computed at Day 35 for the initial lot versus the crossover lot using the data from the participants selected for analysis.

Pediatric Expansion

Descriptive analysis without a pre-specified NI margin will be carried out for the serum antibody levels specific for the SARS-CoV-2 S protein antigen(s) (IgG antibody to SARS-CoV-2 S protein and hACE2 inhibition) at Day 35 comparing adolescents and young adults 18 to < 26 years of age using the same statistical method as the

primary endpoint. Evaluation of immune responses will also assess the 12 to < 15 and 15 to < 18 years age groups of the **Pediatric Expansion**.

The secondary immunogenicity analyses will be performed using the PP-IMM analysis set and the FAS.

Safety Analyses

Adult Main Study and Pediatric Expansion

In formal analyses, numbers and percentages (with 95% CIs based on the Clopper-Pearson method) of participants with solicited local and systemic AEs through 7 days after each vaccination of the initial set of vaccinations will be summarized by trial vaccine group and the maximum toxicity grade over 7 days after each vaccination. The duration of solicited local and systemic AEs after each vaccination will also be summarized by trial vaccine group. Reactogenicity will not be collected following the crossover set of vaccinations.

Unsolicited AEs will be coded by preferred term and system organ class using the latest version of the Medical Dictionary for Regulatory Activities (MedDRA) and summarized by trial vaccine group as well as by severity and relationship to trial vaccine. All AEs through 28 days after second injection of each set of vaccinations (initial and crossover); all MAAEs related to vaccine, SAE, or AESI through EoS will be listed separately and summarized by trial vaccine group. Participants who choose to be unblinded prior to the blinded crossover and receive EUA-authorized vaccine will be followed for safety (SAE, MAAE, AESI and COVID-19 diagnosis) by remote contact on the remaining schedule as specified in Table 1 and Table 2. No further eDiary entries, nasal swabs or blood draws for immunogenicity will be required.

Vital sign measurements will be summarized by trial vaccine group at each time point using descriptive statistics.

Concomitant medications will be summarized by trial vaccine group and preferred drug name as coded using the World Health Organization (WHO) drug dictionary.

Statistical Analyses

At analyses of primary and secondary endpoints prior to end of study, the study will remain blinded at the participant level for study site personnel and study participants until the end of the study (24 months after the first vaccination) while the Sponsor will be unblinded at the participant level to prepare for regulatory submissions. There will be an unblinded statistician and programmer isolated (by firewall) from study personnel that prepare these analyses.

Interim Analyses

Adult Main Study and Pediatric Expansion

There are no formal interim analyses planned that require adjustment to Type I error.

Analyses planned before end of study include a single analysis of primary and secondary efficacy endpoints in the **Adult Main Study** and a subsequent analysis of data from the **Pediatric Expansion** at a cutoff point after initiation of the blinded crossover when participant data up to the crossover or cutoff date (whichever is earlier) has been accumulated and cleaned for all subjects. An analysis of safety and immunogenicity at Day 35 for adolescents (or at a later time point) may be conducted for the **Pediatric Expansion,** also prior to the end of the study**.** For the **Adult Main Study** and **Pediatric Expansion**, after Month 12, an analysis will be performed to examine durability effect of the vaccine on efficacy endpoints as described in the next section. The analysis at Month 12 will also report on safety follow-up through 1 year.

Monitoring Potential Vaccine Harm

Adult Main Study

The DSMB will monitor the study for potential vaccine harm in the **Adult Main Study** based on imbalance in the primary efficacy endpoint, ie, all mild, moderate or severe COVID-19 cases, and severe COVID-19 cases between SARS-CoV-2 rS with Matrix-M1 adjuvant versus placebo. For harm monitoring, cases will be counted starting on Day 0 after the first dose of study vaccination. If the prespecified stopping boundary is reached for either mild, moderate and severe COVID-19 or severe COVID-19, then the ICON unblinded statistician will

immediately inform the DSMB that the harm rules have been met. This monitoring guideline is chosen to allow stopping for prudence as early as possible, maximizing participant safety.

Monitoring of possible harm and any other potential safety issues in the **Pediatric Expansion** will be overseen by the Sponsor, in close collaboration with the DSMB.

Post Blinded Crossover

Adult Main Study

Following blinded crossover, follow-up as specified for the first 12 months of study will continue to collect by remote contact safety and efficacy endpoints after crossover. Follow-up during Months 12-24 will continue via remote contacts every 3-6 months through study completion (except when a visit is scheduled and replaces the remote contact) with the intent to capture 2 years follow-up on each participant. Once all participants have had 1 year of follow-up from enrollment, an analysis will be executed to examine durability effect of the vaccine on efficacy endpoints, taking into account the timing of the blinded crossover as described in Follmann et al, 2020.

Pediatric Expansion

Following blinded crossover, follow-up with weekly remote contacts will continue to collect safety and COVID-19 diagnoses after crossover through Month 12. Follow-up during Months 12-24 will continue via remote contacts every 3 months through study completion (except when a visit is scheduled and replaces the remote contact) with the intent to capture ≥ 12 months follow-up on each participant following receipt of active vaccine. An analysis of immune responses and "effectiveness" data may be prepared for regulatory submission after all Day 35 data have been collected. Once all participants have had 1 year of follow-up from enrollment, planned analysis will be executed to examine durability effect of the vaccine on efficacy endpoints, taking into account the timing of the blinded crossover as described in Follmann et al, 2020. Antibody persistence will be determined by collecting samples for effectiveness assessment at 12, 18 and 24 months post initial set of vaccinations, taking into account the timing of the blinded crossover.

Data and Safety Monitoring Board

In the **Adult Main Study and Pediatric Expansion**, a centralized DSMB will be established in collaboration with NIH, NIAID, Biomedical Advanced Research and Development Authority (BARDA) and Novavax according to the charter dictated by the participating groups. This group will then review interim unblinded data regularly in the **Adult Main Study and Pediatric Expansion** and make recommendations with respect to safety and emerging efficacy. Furthermore, the DSMB may recommend that the trial be terminated or that specific groups be withdrawn from the study, if any subgroup manifests serious or widespread side effects. The DSMB will be informed immediately by the ICON unblinded statistician if the pre-specified stopping boundary is met in the **Adult Main Study**, indicating that the vaccine causes harm by increasing the rate of mild, moderate or severe COVID-19.

Date of the Protocol: 13 May 2021

3 SCHEDULE OF ASSESSMENTS

The Schedule of Assessments (SoA) is presented in Table 1 for the **Adult Main Study** and in Table 2 for the **Pediatric Expansion**.

Table 1 Schedule of Assessments in the Adult Main Study

Study Period:	Screening Period	Initial Vaccination Period			Crossover Vaccination period		Safety Follow-up call[26]	Unscheduled Visits			Months Following Initial Vaccination		
								Acute Illness[3]	Convalescent[4]	General[5]			
**Study Day:**	–30 to 0	0[1]	21	35	C1	C2	3- and 6-Months Post-Crossover	–	–	–	12[24]	18[24]	24[24]
**Window (days)[2]:**	–	0	+ 7	+ 7	0	+ 7	±30	–	–	–	± 15	± 30	± 30
**Minimum Days Following Most Recent Vaccination[2]:**	–	0	21	14	-	21	–	–	–	–	360	540	720
**Days Following Most Recent Vaccination[2]**	–	–	–	–	-	-	–	–	–	–	360	540	720
**Study Visit:**	Screening	1	2[25]	3[25]	4[25]	5[25]	Phone call	Acute Illness[25]	Convalescent[25]	General[25]	6[25]	7[25]	EoS[6, 25]
Informed consent	X												
Medical and social history[7]	X												
Inclusion/exclusion criteria	X	X[8,9]	X[8,9]										
Demographics[10]	X												
Prior/concomitant medications		X[8,9]	X[8,9]	X	X[8,9]	X[8,9]		X	X	X	X[11]	X[11]	X[11]
Vital sign measurements (including body temperature)	X	X[12]	X[12]		X[12]	X[12]		X					
Urine pregnancy test[13]	X	X[9]	X[9]		X[9]	X[9]							
Targeted physical examination[14]	X	X[9]	X[9]	X	X[9]	X		X[15]	X	X	X		
Blood sampling for SARS-CoV-2 (anti-NP) antibodies		X[9]		X	X			X	X		X	X	X
Vaccination		X	X		X	X							
Reactogenicity[16,17]		X	X										
Blood sampling for SARS-CoV-2 vaccine immunogenicity (IgG antibody to SARS-CoV-2 S protein, MN, hACE2 inhibition)[18]		X[9]	X[9]	X	X			X	X		X	X	X
Blood sampling for whole blood, CMI[19]		X	X	X									

Table 1 Schedule of Assessments in the Adult Main Study

Study Period:	Screening Period	Initial Vaccination Period			Crossover Vaccination period		Safety Follow-up call[26]	Unscheduled Visits: Acute Illness[3]	Unscheduled Visits: Convalescent[4]	Unscheduled Visits: General[5]	Months Following Initial Vaccination		
**Study Day:**	**–30 to 0**	**0[1]**	**21**	**35**	**C1**	**C2**	**3- and 6-Months Post-Crossover**	–	–	–	**12[24]**	**18[24]**	**24[24]**
**Window (days)[2]:**	–	**0**	**+ 7**	**+ 7**	**0**	**+ 7**	**±30**	–	–	–	**± 15**	**± 30**	**± 30**
**Minimum Days Following Most Recent Vaccination[2]:**	–	**0**	**21**	**14**	**-**	**21**	–	–	–	–	**360**	**540**	**720**
**Days Following Most Recent Vaccination[2]**	–	–	–	–	**-**	**-**	–	–	–	–	**360**	**540**	**720**
**Study Visit:**	**Screening**	**1**	**2[25]**	**3[25]**	**4[25]**	**5[25]**	**Phone call**	**Acute Illness[25]**	**Convalescent[25]**	**General[25]**	**6[25]**	**7[25]**	**EoS[6, 25]**
Monitoring for COVID-19 illness[20]			From 4 days after initial vaccination using eDiary										
Nasal swab(s) at clinic – anterior nares[3]		X[9]			X[9]			X					
Nasal self-swab(s) by participant – anterior nares		Starting on Day 4, when fever or other specified symptoms are reported in the eDiary for at least 2 consecutive days for the same symptom, participants will begin daily nasal self-swabbing within 3 days of symptom onset at home for a total of 3 days to ensure capture of intermittent shedding											
Daily oxygen saturation via pulse oximeter (supplied by study site at Unscheduled Acute Illness Visit)[3]			At Unscheduled Acute Illness Visit, study site records O_2 saturation measured while training the participant on the use of the device and provides the device for use at home until symptoms resolve										
All unsolicited AEs since prior visit[21]		X	X	X	X[20]	X[20]		X[20]	X[20]				
All MAAEs[21]		X	X	X	X[20]	X[20]		X[20]	X[20]				
MAAEs since last visit/contact[21]								X[20]	X[20]				
Any MAAE attributed to vaccine		X	X	X	X	X	X	X	X	X	X	X	X
SAEs	X	X	X	X	X	X	X	X	X	X	X	X	X
AESI[22]	X	X	X	X	X	X	X	X	X	X	X	X	X
Endpoint Review[4]									X				
EoS form[23]													X

Abbreviations: AE = adverse event; AESI = adverse event(s) of special interest; BMI = body mass index; BP = blood pressure; C1 = first vaccination visit of the crossover vaccination period; C2 = second vaccination visit of the crossover vaccination period; CMI = cell-mediated immunity; COVID-19 = coronavirus disease 2019; eDiary = electronic patient-reported outcome diary application; ELISA = enzyme-linked immunosorbent assay; EoS = end of study; FDA = United States Food and Drug Administration; FLU-PRO = InFLUenza Patient-Reported Outcome; hACE2 = human angiotensin-converting enzyme 2; HIV = human immunodeficiency virus; MAAE = medically attended adverse event; MN = microneutralization; NP = nucleoprotein; O_2 = oxygen; PBMC = peripheral blood mononuclear cells; PIMMC = potential immune-mediated medical conditions; PCR = polymerase chain reaction; RNA = ribonucleic acid; SAE = serious adverse event; SARS-CoV-2 = severe acute respiratory syndrome coronavirus 2; Th1 = type 1 T helper; Th2 = type 2 T helper.

Table 1 Schedule of Assessments in the Adult Main Study

Study Period:	Screening Period	Initial Vaccination Period			Crossover Vaccination period		Safety Follow-up call[26]	Unscheduled Visits			Months Following Initial Vaccination		
								Acute Illness[3]	Convalescent[4]	General[5]			
**Study Day:**	–30 to 0	0[1]	21	35	C1	C2	3- and 6-Months Post-Crossover	–	–	–	12[24]	18[24]	24[24]
**Window (days)[2]:**	–	0	+ 7	+ 7	0	+ 7	±30	–	–	–	± 15	± 30	± 30
**Minimum Days Following Most Recent Vaccination[2]:**	–	0	21	14	-	21	–	–	–	–	360	540	720
**Days Following Most Recent Vaccination[2]**	–	–	–	–	-	-	–	–	–	–	360	540	720
**Study Visit:**	Screening	1	2[25]	3[25]	4[25]	5[25]	Phone call	Acute Illness[25]	Convalescent[25]	General[25]	6[25]	7[25]	EoS[6, 25]

1. If screening and randomization occur on the same day (ie, Day 0), study visit procedures should not be duplicated.
2. Days relative to vaccination are only estimates because the window allowance is not inclusive. Should a study pause occur, visits/windows will be adjusted to allow participants to continue without protocol deviation. Visit schedules following the vaccinations are calculated relative to the day the vaccinations were received. The timing of the 2 blinded crossover visits (C1 and C2) is dependent on public policy issues, the rate of primary endpoint accrual and the timing of regulatory authorization for Emergency Use. For Months 12, 18 and 24 visits, "Minimum Days Following Most Recent Vaccination" refers to initial vaccination 2 (Day 21).
3. At the in-person Unscheduled Acute Illness Visit, participants will be queried regarding AE symptoms, concomitant medications taken for these symptoms, undergo a targeted physical examination (to include O_2 saturation and respiratory rate), if indicated by signs and symptoms, and have obtained by the study personnel a medically attended nasal swab, a blood sample for serologic testing and be trained on the use of the portable pulse oximeter that they will take home with them.
4. Study participants whose home nasal self-swab and/or medically attended nasal swabs are confirmed to be PCR-positive for SARS-CoV-2 at the Unscheduled Acute Illness Visit will be contacted by the study site to arrange an Unscheduled Convalescent Visit. The Unscheduled Convalescent Visit will occur approximately 1 month (or as soon thereafter, as feasible) after the onset of the PCR-confirmed case of COVID-19 at the Unscheduled Acute Illness Visit to assess status of AEs, record the clinical course of the disease on the Endpoint Form and obtain a blood sample for convalescent serologic testing. Pulse oximeters should be returned to the study site at this visit.
5. An Unscheduled General Visit will be conducted by study personnel in the event of a general medical issue other than COVID-19 symptomatology.
6. EoS visit. Should participants decide to terminate early, an EoS telephone visit will occur to collect the maximum safety data and blood sample, if possible.
7. Including prior and concomitant medical conditions, recent vaccinations (≤ 90 days), and significant surgical procedures.
8. Should participants start specific medications or have specific diagnoses that would have been exclusionary at baseline, consultation with the ICON Medical Monitor or Sponsor is required.
9. Performed prior to each vaccination.
10. Screening only. Including date of birth (day, month, and year), sex, race, ethnicity, weight, height, and BMI (derived).
11. Only those medications associated with any MAAE attributed to vaccine, potential AESI, or SAE will be recorded. For day of second dose, recording is prior to each vaccination.
12. On vaccination days, vital sign measurements will be collected once before vaccination to ensure participant has controlled BP and heart rate and no evidence of fever prior to vaccination.

Table 1 Schedule of Assessments in the Adult Main Study

Study Period:	Screening Period	Initial Vaccination Period			Crossover Vaccination period		Safety Follow-up call[26]	Unscheduled Visits			Months Following Initial Vaccination		
								Acute Illness[3]	Convalescent[4]	General[5]			
Study Day:	–30 to 0	0[1]	21	35	C1	C2	3- and 6-Months Post-Crossover	–	–	–	12[24]	18[24]	24[24]
Window (days)[2]:	–	0	+ 7	+ 7	0	+ 7	±30	–	–	–	± 15	± 30	± 30
Minimum Days Following Most Recent Vaccination[2]:	–	0	21	14	-	21	–	–	–	–	360	540	720
Days Following Most Recent Vaccination[2]	–	–	–	–	-	-	–	–	–	–	360	540	720
Study Visit:	Screening	1	2[25]	3[25]	4[25]	5[25]	Phone call	Acute Illness[25]	Convalescent[25]	General[25]	6[25]	7[25]	EoS[6, 25]

13. For participants of childbearing potential (excluding participants who are, or whose male monogamous partners are, surgically sterile or not heterosexually active, or postmenopausal), a urine pregnancy test will be performed at screening and prior to each vaccination. A positive urine pregnancy test at any of the vaccination visits will result in the participant not receiving any further vaccination.
14. Examination at screening to include height and weight. Targeted physical examination after Day 21 is optional, as needed for AE evaluation, except for vaccination visits where it is mandatory.
15. Targeted physical examination at Unscheduled Acute Illness Visit should include recording of respiratory rate and O_2 saturation measured during the process of training the study participant in the use of the device.
16. On vaccination days (either at initial set of vaccinations or at the crossover set of vaccinations), participants will remain in clinic for at least 30 minutes to be monitored for any immediate reaction. Any immediate reaction will be noted as AEs on day of vaccination. Following receipt of the initial set of vaccinations, reactogenicity events that occur after completion of 4 days of reactogenicity reporting (via eDiary) and that meet the criteria for nasal swab for COVID-19 (eg, fever, generalized myalgia, etc) should prompt notification of the study site, collection of a nasal swab and follow-up according to the directions for COVID-19 surveillance. Following the crossover set of vaccinations, surveillance for COVID-19 symptoms will continue as described after Day 28 of the initial set of vaccinations.
17. Participants will utilize an eDiary to record reactogenicity following the initial set of vaccinations. All participants will record reactogenicity starting on the same day of the vaccinations and for an additional 6 days (not counting vaccination day). Study site personnel will regularly review the eDiary for completeness. Should any reactogenicity event extend beyond 7 days after vaccination (toxicity grade ≥ 1), then it will be recorded as an AE with the same start date as the reactogenicity event and followed to resolution per FDA guidelines for dataset capture. 7-day reactogenicity will not be captured after the crossover set of vaccinations.
18. A subset of approximately 900 participants 18 to ≤ 64 years of age enrolled at selected sites will be asked to return for a visit approximately 14 days after the second crossover vaccination dose to provide a blood sample for immunogenicity.
19. Subset of participants (< 100) enrolled at pre-identified study site(s) with the capability to process blood samples for PBMC.
20. Adult participants will be provided with a thermometer and instructed to monitor their body temperature daily throughout the study and to record temperature and relevant symptoms daily in their eDiary. Participants who are noted during regular monitoring of the daily eDiary entries to not have reported temperature and symptoms for ≥ 7 days will be contacted by phone to assess clinical situation and maintain engagement in the study. Starting on Day 4, throughout the first 12 months of the study, when fever or other specified symptoms (see Table 5 for symptoms suggestive of COVID-19) are reported in the eDiary for at least 2 consecutive days for the same symptom, participants will be directed via the eDiary to begin daily nasal self-swabbing for PCR testing within 3 days of symptom onset at home for a total of 3 consecutive days and

Table 1 Schedule of Assessments in the Adult Main Study

Study Period:	Screening Period	Initial Vaccination Period			Crossover Vaccination period		Safety Follow-up call[26]	Unscheduled Visits			Months Following Initial Vaccination		
								Acute Illness[3]	Convalescent[4]	General[5]			
Study Day:	–30 to 0	0[1]	21	35	C1	C2	3- and 6-Months Post-Crossover	–	–	–	12[24]	18[24]	24[24]
Window (days)[2]:	–	0	+ 7	+ 7	0	+ 7	±30	–	–	–	± 15	± 30	± 30
Minimum Days Following Most Recent Vaccination[2]:	–	0	21	14	-	21	–	–	–	–	360	540	720
Days Following Most Recent Vaccination[2]	–	–	–	–	-	-	–	–	–	–	360	540	720
Study Visit:	Screening	1	2[25]	3[25]	4[25]	5[25]	Phone call	Acute Illness[25]	Convalescent[25]	General[25]	6[25]	7[25]	EoS[6, 25]

to initiate daily completion of the FLU-PRO symptom reporting instrument for 10 days after COVID-19 symptom onset or until the participant experiences 2 consecutive asymptomatic days. In addition, the eDiary will alert the study site to contact the participant to schedule the in-person Unscheduled Acute Illness Visit.

21. Through 49 days, ie, 28 days after second injection of the initial and crossover sets of vaccinations.
22. AESI: To include PIMMC (listed in Appendix 2, Table 11), or any newly identified complications of COVID-19 (listed in Appendix 2, Table 12) or other potential AESI followed through 24 months after participants' initial set of vaccinations.
23. EoS form will be completed for all participants, including those who are terminated early.
24. From Months 12 to 24, study sites will initiate remote contacts (phone, email, text) with participants at Months 15 and 21 to collect SAEs, MAAEs attributed to vaccine, AESIs or COVID-19 illness, in addition to conducting in-person visits for blood draws at Month 12, 18 and 24 (ie, no remote contact needed when in-person visits are scheduled at these time points).
25. Participants that requested to be unblinded and/or are continuing in the study for Safety Follow-up will have visits replaced by remote contact (phone, email, text), at a minimum, every 90 days (± 30 days) from last dose received during the first year of participation. During the second year (ie, Months 12-24), contact will be continued every 180 days (± 30 days). **No eDiary, nasal swabs or blood samples will be collected during safety follow-up**. MAAEs, SAEs, AESI, and COVID-19 diagnoses will be collected during the remote contacts at the same time points described in Table 1 for scheduled visits in the participants that did not request unblinding. Receipt of another COVID-19 vaccine used under EUA should be recorded as a concomitant medication. The End of Study page is not to be completed for these participants until the end of their participation in the study.
26. Participants who do not report symptoms of possible COVID-19 in their eDiary during the period after blinded crossover until Month 12 should be contacted remotely by sites to collect any SAEs, MAAEs attributed to study vaccine or AESIs.

Table 2 Schedule of Assessments in the Pediatric Expansion

**Study Period:**	**Screening Period**	**Initial Vaccination Period**								**Crossover Vaccination period**		**Regular remote contact**	**Unscheduled visit[4]**			**Months Following Initial Vaccination**		
													**Acute Illness[3]**	**Convalescent[4]**	**General[5]**			
**Study Day:**	**–7 to 0**	**0[1]**	**7**	**14**	**21**	**28**	**35**	**42**	**49**	**C1**	**C2**	**56-M24[21]**	–	–	–	**12[21]**	**18[21]**	**24[21]**
**Window (days)[2]:**	–	**0**	**+ 3**	**+ 3**	**+ 7**	**+3**	**+ 7**	**+ 3**	**+ 7**	**+ 7**	**+ 7**	**+3**	–	–	–	**± 15**	**± 30**	**± 30**
**Minimum Days Following Most Recent Vaccination[2]:**	–	**0**	–	–	**21**	–	**14**	–	**28**	**180**	**21**	–	–	–	–	**360**	**540**	**720**
**Study Visit:**	**Screening**	**1**	**2**	**PC**	**3**	**4**	**5**	**PC**	**6**	**7**	**8**	**Phone call**	**Acute Illness[22]**	**Convalescent[22]**	**General[22]**	**9**	**10**	**EoS[6,22]**
Informed consent	X																	
Medical and social history[7]	X																	
Inclusion/exclusion criteria	X	X[8,9]			X[8,9]													
Demographics[10]	X																	
Prior/concomitant medications		X[8,9]	X	X	X[8,9]	X	X	X	X	X[8,9]	X[9]	X[11]	X	X	X	X[11]	X[11]	X[11]
Vital sign measurements (including body temperature)	X	X[12]			X[12]					X[12]	X[12]		X					
Urine pregnancy test[13]	X	X[9]			X[9]					X[9]	X[9]							
Targeted physical examination[14]	X	X[9]			X[9]		X			X[9]	X		X	X	X	X		
Randomization		X																
Blood sampling for SARS-CoV-2 (anti-NP) antibodies		X[9]					X			X[9]			X	X	X	X	X	X
Vaccination		X			X					X	X							
Reactogenicity[15,16]		X			X					X	X							
Blood sampling for SARS-CoV-2 vaccine immunogenicity (IgG antibody to SARS-CoV-2 S protein, MN, hACE2 inhibition)		X[9]			X[9]		X			X[9]			X	X		X	X	X
Blood sampling for whole blood, CMI[17]		X[9]	X			X[9]												
Safety follow-up phone call			X	X		X		X	X			X						
Monitoring for COVID-19 illness[18]		From 4 days after initial vaccination, collected in paper memory aid and reported to the sites by the parent(s)/caregiver(s) via remote contact																
Nasal swab(s) at clinic – anterior nares[3]		X[9]								X[9]			X					
All unsolicited AEs since prior visit[23]		X	X	X	X	X	X	X	X	X	X	X	X	X	X			
All MAAEs[23]		X	X	X	X	X	X	X	X	X	X	X	X	X	X			
Any MAAE attributed to vaccine		X	X	X	X	X	X	X	X	X	X	X	X	X	X	X	X	X

Table 2 Schedule of Assessments in the Pediatric Expansion

Study Period:	Screening Period	Initial Vaccination Period								Crossover Vaccination period		Regular remote contact	Unscheduled visit[4]			Months Following Initial Vaccination		
													Acute Illness[3]	Convalescent[4]	General[5]			
Study Day:	–7 to 0	0[1]	7	14	21	28	35	42	49	C1	C2	56-M24[21]	–	–	–	12[21]	18[21]	24[21]
Window (days)[2]:	–	0	+ 3	+ 3	+ 7	+3	+ 7	+ 3	+ 7	+ 7	+ 7	+3	–	–	–	± 15	± 30	± 30
Minimum Days Following Most Recent Vaccination[2]:	–	0	–	–	21	–	14	–	28	180	21	–	–	–	–	360	540	720
Study Visit:	Screening	1	2	PC	3	4	5	PC	6	7	8	Phone call	Acute Illness[22]	Convalescent[22]	General[22]	9	10	EoS[6, 22]
SAEs	X	X	X	X	X	X	X	X	X	X	X	X	X	X	X	X	X	X
AESI[19]	X	X	X	X	X	X	X	X	X	X	X	X	X	X	X	X	X	X
Endpoint Review form[4]														X				
EoS form[20]																		X

Abbreviations: AE = adverse event; AESI = adverse event(s) of special interest; BMI = body mass index; BP = blood pressure; C1 = first vaccination visit of the crossover vaccination period; C2 = second vaccination visit of the crossover vaccination period; CMI = cell-mediated immunity; COVID-19 = coronavirus disease 2019; eDiary = electronic patient-reported outcome diary application; EoS = end of study; EUA = Emergency Use Authorization; FDA = United States Food and Drug Administration; hACE2 = human angiotensin-converting enzyme 2; IgG = immunoglobulin G; M24 = Month 24; MAAE = medically attended adverse event; MN = microneutralization; NP = nucleoprotein; O_2 = oxygen; PBMC = peripheral blood mononuclear cells; PC = phone call; PCR = polymerase chain reaction; PIMMC = potential immune-mediated medical conditions; S = spike; SAE = serious adverse event; SARS-CoV-2 = severe acute respiratory syndrome coronavirus 2.

1. If screening and randomization occur on the same day (ie, Day 0), study visit procedures should not be duplicated.
2. Days relative to vaccination are only estimates because the window allowance is not inclusive. Should a study pause occur, visits/windows will be adjusted to allow participants to continue without protocol deviation. Visit schedules following the vaccinations are calculated relative to the day the vaccinations were received. The first blinded crossover visits (C1) will be conducted after approximately 6 months (or earlier depending upon feasibility, EUA vaccine availability for this age group, and the dynamics of the pandemic) of follow-up after completion of the initial set of vaccinations has occurred. For Months 12, 18 and 24 visits, “Minimum Days Following Most Recent Vaccination” refers to initial vaccination 2 (Day 21).
3. At the in-person Unscheduled Acute Illness Visit, participants will be queried regarding AE symptoms, concomitant medications taken for these symptoms, undergo a targeted physical examination (to include O_2 saturation and respiratory rate), if indicated by signs and symptoms, and have obtained by the study personnel a medically attended nasal swab, and a blood sample for serologic testing.
4. Study participants whose medically attended nasal swabs are confirmed to be PCR-positive for SARS-CoV-2 at the Unscheduled Acute Illness Visit will be contacted by the study site to arrange an Unscheduled Convalescent Visit. The Unscheduled Convalescent Visit will occur approximately 1 month (or as soon thereafter, as feasible) after the onset of the PCR-confirmed case of COVID-19 at the Unscheduled Acute Illness Visit to assess status of AEs, record the clinical course of the disease on the Endpoint Form and obtain a blood sample for convalescent serologic testing.
5. An Unscheduled General Visit will be conducted by study personnel in the event of a general medical issue other than COVID-19 symptomatology.
6. EoS visit. Should participants decide to terminate early, an EoS telephone visit will occur to collect the maximum safety data and blood sample, if possible.
7. Including prior and concomitant medical conditions, recent vaccinations (≤ 90 days), and significant surgical procedures.

8. Should participants start specific medications or have specific diagnoses that would have been exclusionary at baseline, consultation with the ICON Medical Monitor or Sponsor is required.
9. Performed prior to each vaccination. There are 7 scheduled blood draws for immunogenicity assessments at the Day 0, 21, 35, C1 (Day 180), and Months 12, 18, and 24 visits, and during unscheduled visits (ie Acute illness, Convalescent, and General). For participants in the CMI cohort only (N=50), additional blood will be collected to obtain PBMCs at the Day 0, 7 and 28 visits.
10. Screening only. Including date of birth (day, month, and year), sex, race, ethnicity, weight, height, and BMI (derived).
11. Only those medications associated with any MAAE attributed to vaccine, potential AESI, or SAE will be recorded. For day of second dose, recording is prior to each vaccination.
12. On vaccination days, vital sign measurements will be collected once before vaccination to ensure participant has controlled BP and heart rate and no evidence of fever prior to vaccination.
13. For participants of childbearing potential (excluding participants who are premenarche or surgically sterile), a urine pregnancy test will be performed at screening and prior to each vaccination. A positive urine pregnancy test at any of the vaccination visits will result in the participant not receiving any further vaccination.
14. Examination at screening to include height and weight. Targeted physical examination after Day 21 is optional, as needed for AE evaluation, except for vaccination visits where it is mandatory.
15. On vaccination days (either at initial set of vaccinations or at the crossover set of vaccinations), participants will remain in clinic for at least 30 minutes to be monitored for any immediate reaction. Any immediate reaction will be noted as AEs on day of vaccination.
16. Parent(s)/caregiver(s) of the participants will utilize an eDiary to record reactogenicity following the initial set of vaccinations. Parent(s)/caregiver(s) of the participants will record reactogenicity starting on the same day of the vaccinations and for an additional 6 days (not counting vaccination day). Parent(s)/caregiver(s) of the participants will be provided with a thermometer and instructed to monitor the participant body temperature daily throughout the reactogenicity period and to record temperature and relevant symptoms daily in their eDiary. Study site personnel will regularly review the eDiary for completeness. Should any reactogenicity event extend beyond 7 days after vaccination (toxicity grade ≥ 1), then it will be recorded as an AE with the same start date as the reactogenicity event and followed to resolution per FDA guidelines for dataset capture. 7-day reactogenicity will not be captured after the crossover set of vaccinations.
17. Subset of 50 adolescent participants enrolled at pre-identified study site(s) with the capability to process blood samples for PBMC.
18. Parent(s)/caregiver(s) of the participants will be instructed to report COVID-19 symptoms / diagnosis to the sites as soon as they are aware or at the weekly remote contact. Starting on Day 4, throughout the first 12 months of the study, when fever or other specified symptoms (see Table 5 for symptoms suggestive of COVID-19) are reported the study site to contact the participant to schedule the in-person Unscheduled Acute Illness Visit and medically-attended nasal swab.
19. AESI: To include PIMMC (listed in Appendix 2, Table 11), or any newly identified complications of COVID-19 (listed in Appendix 2, Table 12) or other potential AESI followed through 24 months after participants' initial set of vaccinations.
20. EoS form will be completed for all participants, including those who are terminated early.
21. From Day 0 to Month 12, study sites will perform weekly remote contacts (phone, email, text) with the participants' parent(s)/caregiver(s) to collect SAEs, MAAEs attributed to vaccine, AESIs or COVID-19 illness except during weeks in which an in-clinic visits is to be performed (eg, Day 21 or Day 35 visits), and every 3 months from Months 12-24 (ie, at Months 15 and 21), in addition to conducting in-person visits for blood draws at Month 12, 18 and 24 (ie, no remote contact needed when in-person visits are scheduled).
22. Participants that requested to be unblinded and/or are continuing in the study for Safety Follow-up will have visits replaced by remote contact (phone, email, text), at a minimum, every 90 days (± 30 days) from last dose received during the first year of participation. During the second year (ie, Months 12-24), contact will be continued every 180 days (± 30 days). **No eDiary, nasal swabs or blood samples will be collected during safety follow-up**. MAAEs, SAEs, AESI, and COVID- 19 diagnoses will be collected during the remote contacts. Receipt of another COVID-19 vaccine used under EUA should be recorded as a concomitant medication. The End of Study page is not to be completed for these participants until the end of their participation in the study.
23. Through 49 days, ie, 28 days after second injection of the initial and crossover sets of vaccinations.

4 TABLE OF CONTENTS

1 GENERAL INFORMATION....3
2 STUDY SYNOPSIS....4
3 SCHEDULE OF ASSESSMENTS....27
4 TABLE OF CONTENTS....35
4.1 List of Tables....40
4.2 List of Figures....40
4.3 List of Appendices....40
5 LIST OF ABBREVIATIONS AND DEFINITION OF TERMS....41
6 INTRODUCTION....45
6.1 Background....45
6.2 Nonclinical Summary....46
6.3 Clinical Summary....48
6.4 Study Rationale....49
6.5 Rationale for Dose Selection....50
6.6 Benefit – Risk Assessment....51
6.6.1 Risk Assessment....51
6.6.2 Overall Benefit – Risk Conclusion....51
7 STUDY OBJECTIVES, ENDPOINTS AND ESTIMANDS....53
7.1 Main Study....53
7.1.1 Primary Objective:....53
7.1.2 Secondary Objectives:....53
7.1.2.1 Key Secondary Objective:....53
7.1.2.2 Other Secondary Objectives:....53
7.1.3 Exploratory Objectives:....55
7.1.4 Primary Endpoint:....56
7.1.5 Secondary Endpoints:....57
7.1.5.1 Key Secondary Endpoint:....57
7.1.5.2 Other Secondary Endpoints:....57
7.1.6 Exploratory Endpoints:....59
7.2 Pediatric Expansion....60
7.2.1 Primary Objective:....60
7.2.2 Secondary Objectives:....61
7.2.3 Exploratory Objectives:....62
7.2.4 Primary Endpoints:....62

7.2.5 Safety Endpoints 64
7.2.6 Effectiveness Endpoint: 65
7.2.7 Secondary Endpoints: 65
7.2.8 Exploratory Endpoints: 66
8 OVERALL STUDY DESIGN AND PLAN 67
8.1 Study Design Description 67
8.1.1 Enrollment 67
8.1.2 Vaccine Administration 69
8.1.3 Safety Monitoring 70
8.1.4 Immunogenicity Testing 71
8.1.5 Prospective Surveillance of COVID-19 72
8.1.6 Periods of Adult Main Study 75
8.1.7 Periods of Pediatric Expansion 76
8.1.8 Safety Follow-up after Unblinding 77
8.1.9 Safety Monitoring 77
8.1.10 Study Vaccination Pause Rules (Pediatric Expansion) 78
8.1.11 Participant Retention 79
8.2 Discussion of Study Design 79
8.2.1 End of Study Definition 81
8.2.2 Trial Vaccine After the End of Study 81
8.3 Study Population 82
8.3.1 Inclusion Criteria 82
8.3.2 Exclusion Criteria 83
8.3.3 Other Considerations: 84
8.4 Prohibited Medications 84
8.5 Lifestyle Considerations 84
8.6 Strategies for Recruitment and Retention 85
8.7 Screen Failures 85
8.8 Trial Vaccine Discontinuation and Participant Discontinuation/Withdrawal 86
8.8.1 Trial Vaccine Discontinuation 86
8.8.2 Study Temporary Discontinuation/Vaccine Pause 86
8.8.3 Withdrawal of Participants 87
8.8.3.1 Discontinuation/Withdrawal by Participant 87
8.8.3.2 Discontinuation/Withdrawal by Investigator 87
8.8.3.3 Discontinuation/Withdrawal by Sponsor (Study Halting Rules) 87

8.8.4 Study Vaccination Pause Rules (Pediatric Expansion) 87
8.9 Lost to Follow-up 88
8.10 Discontinuation of Study Sites 88
9 STUDY TREATMENT 89
9.1 Administration of Study Treatment(s) 89
9.2 Study Treatment Packaging and Labelling 90
9.2.1 Packaging and Labelling 90
9.2.2 Storage 90
9.3 Vaccine Compliance 90
9.4 Prior Vaccinations and Concomitant Therapy 90
9.5 Blinding and Randomization of Study Treatment(s) 91
9.6 Procedure for Breaking the Randomization Code 91
9.7 Study Treatment Accountability 92
10 STUDY ASSESSMENTS AND PROCEDURES 93
10.1 Efficacy Assessments 93
10.1.1 Active Surveillance for COVID-19 93
10.1.2 Unscheduled Acute Illness and Convalescent Visits 94
10.1.3 Nasal Swabs for Virus Detection 95
10.1.4 FLU-PRO (Adult Main Study only) 96
10.1.5 Oxygen Saturation Monitoring 96
10.2 Immunogenicity Assessments 97
10.3 Safety Assessments 98
10.3.1 Definitions 98
10.3.1.1 Adverse Events 98
10.3.1.2 Events Meeting the AE Definition 98
10.3.1.3 Serious Adverse Event 99
10.3.1.4 Treatment-Emergent Adverse Event 100
10.3.1.5 Adverse Event of Special Interest 100
10.3.1.6 Medically Attended Adverse Events 100
10.3.1.7 Reactogenicity Symptoms 100
10.3.1.8 Pregnancy 101
10.3.2 Time Period and Frequency for Collecting AE and SAE Information 101
10.3.3 Method of Detecting AEs, MAAEs and SAEs 102
10.3.4 Recording of AEs and SAEs 102
10.3.4.1 Assessment of Intensity (Severity) 103

10.3.4.2 Assessment of Causality 103
10.3.4.3 Action Taken with Trial Vaccine Due to AE 104
10.3.4.4 Other Action Taken 104
10.3.4.5 AE Outcome 105
10.3.5 Follow-up of AEs/SAEs 105
10.3.6 Reporting of SAEs 105
10.3.6.1 Safety Reporting to Sponsor 106
10.3.6.2 Safety Reporting to Health Authorities, Independent Ethics Committees/Institutional Review Boards and Investigators 106
10.3.6.3 24/7 Medical Emergency Coverage for Urgent Protocol-related Medical Questions 106
10.3.7 Laboratory Assessments 107
10.3.8 Physical Examination 107
10.3.9 Vital Signs 107
10.3.10 Overdose 108
10.4 Other Assessments 108
10.4.1 Medical and Social History 108
10.4.2 Demographics 108
10.4.3 eDiary 108
10.5 Post Blinded Crossover 109
11 MEDICAL RESOURCE UTILIZATION 110
12 STATISTICAL CONSIDERATIONS 111
12.1 Sample Size and Power 111
12.2 Analysis Sets 116
12.2.1 Intent-to-Treat (ITT) Analysis Set 116
12.2.2 Full Analysis Set (FAS) 116
12.2.3 Safety Analysis Set 116
12.2.4 Per-Protocol Efficacy (PP-EFF) Analysis Set 117
12.2.5 Per-Protocol Immunogenicity (PP-IMM) Analysis Set 117
12.2.6 Participant Disposition 118
12.3 Statistical Analyses 118
12.3.1 Primary Endpoint 119
12.3.1.1 Efficacy 119
12.3.2 Secondary Endpoints 120
12.3.2.1 Adult Main Study and Pediatric Expansion 120

12.3.3 Statistical Models ... 121
12.3.4 Handling of Missing Data ... 122
12.3.5 Safety Analyses ... 122
12.4 Interim Analysis ... 123
12.4.1 Adult Main Study and Pediatric Expansion ... 123
12.5 Monitoring Potential Vaccine Harm ... 123
12.6 Safety Monitoring ... 125
12.7 Data Safety and Monitoring Board ... 126
13 ETHICS ... 128
13.1 Independent Ethics Committee/Institutional Review Board ... 128
13.2 Documentation of Informed Consent ... 128
14 QUALITY CONTROL AND QUALITY ASSURANCE ... 129
14.1 Conduct of the Study ... 129
14.2 Site Monitoring ... 129
15 DATA HANDLING AND RECORD KEEPING ... 131
15.1 Case Report Forms/Source Data Handling ... 131
15.2 Data Protection ... 131
15.3 Dissemination of Clinical Study Data ... 131
15.4 Retention of Essential Documents ... 132
16 FINANCING AND INSURANCE ... 133
17 PUBLICATION POLICY ... 134
18 CONFLICT OF INTEREST POLICY ... 135
19 SIGNATURE OF INVESTIGATOR ... 136
20 REFERENCE LIST ... 137
21 APPENDICES ... 139

4.1 List of Tables

Table 1 Schedule of Assessments in the Adult Main Study 27
Table 2 Schedule of Assessments in the Pediatric Expansion 32
Table 3 Adult Main Study - Trial Vaccine Groups 68
Table 4 Pediatric Expansion - Trial Vaccine Groups 69
Table 5 Symptoms Suggestive of COVID-19 75
Table 6 Clinical Laboratory Tests 107
Table 7 Power Under Various Number of Primary Efficacy Endpoints and Vaccine Efficacy Assumptions 111
Table 8 Example Minimum Vaccine Efficacy Needed to Demonstrate Lower Bound of the Confidence Interval > 30%* 112
Table 9 Power to Conclude Non-Inferiority of the Crossover Lot...............................113
Table 10 Power Under Various Number of Primary Efficacy Endpoints and True Vaccine Efficacy Assumptions to Conclude VE > 0% 114
Table 11 Potential Immune-Mediated Medical Conditions 175
Table 12 Adverse Events Representing Complications Specific to of COVID-19[1] 176

4.2 List of Figures

Figure 1 Example of Stopping Boundaries for Potential Vaccine Harm.......................125

4.3 List of Appendices

Appendix 1 Protocol Change History

Appendix 2 Listings of Adverse Events of Special Interest

Appendix 3 Example Stopping Bounds for Vaccine Harm Monitoring

Appendix 4: FDA Toxicity Grading Scale for Local and General Systemic Reactogenicity

5 LIST OF ABBREVIATIONS AND DEFINITION OF TERMS

24/7	24 hours 7 days a week
AE	Adverse event
AESI	Adverse event of special interest
ALCOAC	Attributable, legible, contemporaneous, original, accurate, and complete
AMI	Acute myocardial infarction
ANCA	Anti-neutrophil cytoplasmic antibody
ARDS	Acute respiratory distress syndrome
BARDA	Biomedical Advanced Research and Development Authority
BiPAP	Bilevel positive airway pressure
BMI	Body mass index
BP	Blood pressure
CI	Confidence interval
CMI	Cell-mediated immunity
CONSORT	Consolidated Standards of Reporting Trials
COVID-19	Coronavirus disease 2019
COVPN	COVID-19 Prevention Network
CPAP	Continuous positive airway pressure
CT	Computerized tomography
DAIDS	Division of AIDS, NIAID, NIH
DBP	Diastolic blood pressure
DSMB	Data and Safety Monitoring Board
DVT	Deep vein thrombosis
EBOV	Zaire ebolavirus
ECMO	Extracorporeal membrane oxygenation
eCRF	Electronic case report form
EDC	Electronic data capture
eDiary	Electronic patient-reported outcome diary application
ELISA	Enzyme-linked immunosorbent assay
EMA	European Medicines Agency
EnvD	Envelope dimers
EoS	End of study
ER	Emergency room
EU	European Union

EUA	Emergency Use Authorization
EudraCT	European Union Drug Regulating Authorities Clinical Trials
FAS-EFF	Full Analysis Set - Efficacy
FAS-IMM	Full Analysis Set - Immunogenicity
FDA	United States Food and Drug Administration
FLU-PRO	InFLUenza Patient-Reported Outcome (questionnaire)
GCP	Good Clinical Practice
GLP	Good Laboratory Practice
GMFR	Geometric mean fold rise
GMT	Geometric mean titer
GP	Glycoprotein
HA	Hemagglutinin
hACE2	Human angiotensin-converting enzyme 2
HIV	Human immunodeficiency virus
IB	Investigator's brochure
ICF	Informed consent form
ICH	International Council for Harmonisation
ICU	Intensive care unit
IEC	Independent Ethics Committee
IFN-γ	Interferon gamma
IgA	Immunoglobulin A
IgG	Immunoglobulin G
IL	Interleukin
IM	Intramuscular
IND	Investigational New Drug
IRB	Institutional Review Board
ITT-EFF	Intent-to-Treat Efficacy
ITT-IMM	Intent-to-Treat Immunogenicity
IV	Intravenous
IWRS	Interactive web response system
LRTI	Lower respiratory tract infection
MAAE	Medically attended adverse event
MedDRA	Medical Dictionary for Regulatory Activities
MERS	Middle East Respiratory Syndrome
MN	Microneutralization

NHP	Nonhuman primate
NI	Non-inferiority
NIAID	National Institute of Allergy and Infectious Diseases
NIH	National Institutes of Health
NIPPV	Non-invasive positive pressure ventilation
NIV	Non-invasive ventilation
NP	Nucleocapsid
NZW	New Zealand White
O_2	Oxygen
OWS	Operation Warp Speed
PBMC	Peripheral blood mononuclear cell
PCR	Polymerase chain reaction
PE	Pulmonary embolism
PHEIC	Public health emergency of international concern
PIMMC	Potential immune-mediated medical conditions
PP-EFF	Per-protocol efficacy
PP-IMM	Per-protocol immunogenicity
PSRT	Protocol Safety Review Team
PVSS	Pharmacovigilance and Safety Services
r	Recombinant
RNA	Ribonucleic acid
RR	Relative risk
RSV	Respiratory syncytial virus
S	Spike
SAE	Serious adverse event
SAP	Statistical analysis plan
SARS-CoV	Severe acute respiratory syndrome coronavirus
SBP	Systolic blood pressure
SoA	Schedule of assessments
SOP	Standard operating procedure
SUSAR	Suspected unexpected serious adverse reaction
TEAE	Treatment-emergent adverse event
Th1	Type 1 T helper
Th2	Type 2 T helper
TNF-α	Tumor necrosis factor alpha

US	United States
VE	Vaccine efficacy
VLP	Virus-like particle
WHO	World Health Organization
ZIKV	Zika virus

6 INTRODUCTION

6.1 Background

Coronaviruses are medium sized, enveloped, positive-stranded ribonucleic acid (RNA) viruses, with a characteristic crown-like appearance in electron micrographs due to circumferential studding of the viral envelope with projections comprising the S protein. There are 4 different strains (229E, OC43, NL63, and HKU1), which are ubiquitous in humans and generally result in mild upper respiratory illnesses and other common cold symptoms including malaise, headache, nasal discharge, sore throat, fever, and cough [Su 2016]. In addition, other coronavirus strains are widespread in animals, where they typically cause enteric disease. These zoonotic coronaviruses have been known to evolve into strains that can infect humans with serious consequences including severe acute respiratory syndrome coronavirus (SARS-CoV) from 2002 to 2003, Middle East Respiratory Syndrome (MERS)-CoV since 2012, and most recently, the novel SARS-CoV-2 since 2019 [Habibzadeh 2020].

In late December of 2019, an outbreak of respiratory disease caused by novel coronavirus (2019-nCoV) was detected in Wuhan, Hubei province, China. The virus' rapidly discerned genetic relationship with the 2002-2003 SARS-CoV has resulted in adoption of the name "SARS-CoV-2," with the disease being referred to as coronavirus disease 2019 (COVID-19). Despite containment efforts since the start of the outbreak, the SARS-CoV-2 has spread rapidly with over 214 countries/territories/areas outside of China reporting laboratory confirmed COVID-19 cases as of 15 May 2020 [WHO 2020] On 30 January 2020, the International Health Regulations Emergency Committee of the World Health Organization (WHO) designated the outbreak as a public health emergency of international concern (PHEIC) and subsequently declared a pandemic on 11 March 2020.

Novavax, Inc. is developing a recombinant vaccine adjuvanted with the saponin-based Matrix-M1™ adjuvant for the prevention of disease caused by SARS-CoV-2. SARS-CoV-2 recombinant (r) spike (S) protein nanoparticle vaccine (SARS-CoV-2 rS) is constructed from the full-length, wild-type SARS-CoV-2 S glycoprotein (GP) based on the GenBank gene sequence MN908947, nucleotides 21563-25384 from the 2019 SARS-CoV-2 genome. The S protein is a type 1 trimeric glycoprotein of 1,273 amino acids that is produced as an inactive S0 precursor. The S-gene was codon-optimized for expression in *Spodoptera frugiperda* insect cells. The SARS-CoV-2 rS nanoparticle vaccine is intended for administration with Matrix-M1 adjuvant, which is a saponin-based adjuvant that has previously been shown to enhance the immunogenicity of other nanoparticle vaccines in nonclinical and clinical studies.

The purpose of this study is to evaluate the efficacy, safety, and immunogenicity of SARS-CoV-2 rS with Matrix-M1 adjuvant in adult participants ≥ 18 years of age (**Adult Main Study**). Clinical endpoints will be assessed overall, and also within age subgroups, with the main age strata 18 to ≤ 64 years and ≥ 65 years. All study participants will receive 2 doses of trial

vaccine, on Days 0 and 21 + 7 days. The dose/immunization schedule implemented in this study is based on the optimal safety and immunogenicity data observed in the nonclinical and early clinical studies. A **Pediatric Expansion** is being added to evaluate the safety and effectiveness of SARS-CoV-2 rS with Matrix-M1 adjuvant in adolescents (12 to < 18 years).

6.2 Nonclinical Summary

In support of the development of SARS-CoV-2 rS, Novavax has obtained nonclinical pharmacology data concerning several SARS-CoV-2 S protein variants, toxicity data concerning SARS-CoV-2 rS with Matrix-M1 adjuvant, and prior toxicity data concerning other viral glycoproteins manufactured in the baculovirus-Sf9 system and formulated with Matrix-M1 adjuvant.

Nonclinical Data from SARS-CoV-2 Spike Protein Constructs that Support SARS-CoV-2 rS Development

Mouse immunogenicity studies were conducted to evaluate several SARS-CoV-2 S protein variants and select the vaccine candidate [Tian 2020]. The selected vaccine candidate, BV2373 (3Q-2P), was demonstrated to be immunogenic and elicited functional antibodies. For the tested constructs, shallow dose responses with Matrix-M1 adjuvant were observed, suggesting that the adjuvant may be significantly antigen-sparing in large animals and humans.

The candidate SARS-CoV-2 rS vaccine, based on the BV2373 construct, has been evaluated in dose titration studies in hamsters, cynomolgus macaques and baboons. In hamsters, 2-dose regimens with 1 or 10 µg SARS-CoV-2 rS/15 µg Matrix-M1 adjuvant were highly immunogenic, resulting in high anti-S immunoglobulin G (IgG) titers and high human angiotensin-converting enzyme 2 (hACE2) binding inhibition titers in a shallow dose-dependent manner, suggestive of dose-sparing. Of note, anti-S IgG levels and hACE2 binding inhibition titers were highly correlated. In cynomolgus macaques, 2-dose regimens of 5 or 25 µg SARS-CoV-2 rS/25 or 50 µg Matrix-Ml adjuvant were also highly immunogenic, resulting in high anti-S IgG levels, high hACE2 binding inhibition titers, and high neutralizing antibody responses. The 5 and 25 µg antigen doses gave generally similar responses when administered twice with 50 µg of Matrix-Ml adjuvant. In baboons, which may be more predictive of responses in humans, 5 and 25 µg SARS-CoV-2 rS/50 µg Matrix-Ml adjuvant induced high levels of anti-S IgG, hACE2-binding inhibiting antibodies, and neutralizing antibodies. Matrix-Ml adjuvant provided antigen-sparing, and supported induction of functional antibodies. Importantly, Matrix-Ml adjuvanted SARS-CoV-2 rS also appeared to induce strong Thl type CD4+ T cell responses to SARS-CoV-2 spike protein that included polyfunctional effector phenotypes. Current data in this small baboon study confirms that doses of 5 µg and 25 µg with 50 µg Matrix-M1 were the correct doses to test clinically, with Matrix-M1 adjuvant appearing critical for maximum responses. This finding was confirmed in a Phase 1 trial in humans [Keech 2020].

Virus challenge studies were performed in mice, hamsters, and cynomolgus macaques. In 2 mouse challenge models, immunization with 1 or 2 doses of SARS-CoV-2 rS/Matrix-M1 adjuvant suppressed viral replication, reduced lung inflammation, and reduced systemic morbidity (weight loss) after SARS-CoV-2 live virus challenge and were not associated with any obvious exacerbation of the inflammatory response to the virus or worsening of clinical outcomes. The best responses were seen in animals receiving 2 doses of adjuvanted vaccine. Similar protection (weight loss and activity level) against live virus challenge was seen in hamsters, a SARS-CoV-2 permissive species due to the presence of the ACE2 receptor. Cynomolgus macaques, administered with human doses of 5 or 25 µg SARS-CoV-2 rS adjuvanted with 50 µg Matrix-M1 had high and comparable levels of anti-S IgG titers and hACE2 receptor binding inhibition titers detected 21 days after the first immunization. All of the macaques immunized with any dose or regimen of SARS-CoV-2 rS/Matrix-M1 adjuvant were protected against live virus challenge as evidenced by the reduction of total viral RNA and subgenomic RNA to below the limit of quantitation in bronchoalveolar lavages and nasal swabs.

A Good Laboratory Practice (GLP)-compliant toxicity study in New Zealand White (NZW) rabbits was performed to evaluate 50 µg of SARS-CoV-2 rS (BV2373 construct) with and without 50 µg Matrix-M1 adjuvant. Immunization of rabbits up to 4 times with full human doses of SARS-CoV-2 rS, with or without 50 µg Matrix-M1 adjuvant, was well tolerated, had no effects on mortality, cageside observations, body weight, food consumption, or physical examination findings. There were no test article-related gross or histopathologic findings aside from subacute inflammation at the injection sites, which was deemed a typical response to immunization.

Nonclinical Data from Other Baculovirus-Sf9-Produced Nanoparticle Vaccines that Support SARS-CoV-2 rS Development

The immunogenicity and protective efficacy of 2002-2003 SARS-CoV S protein and chimeric influenza/SARS-CoV virus-like particle (VLP) vaccines produced in the baculovirus-Sf9 system and administered with and without aluminum hydroxide adjuvants was demonstrated in a mouse challenge study [Liu 2011]. Robust neutralizing antibody titers were observed following vaccination, although both antigens required adsorption to aluminum hydroxide for optimal responses. The immunogenicity and protective efficacy of a MERS-CoV S nanoparticle vaccine with and without Matrix-M1 adjuvant was demonstrated in a mouse challenge study [Coleman 2017]. Following vaccination, the MERS-CoV S nanoparticle was immunogenic across all active treatment groups; however, the presence of Matrix-M adjuvant induced a 3 to > 10-fold enhancement of the binding and neutralizing antibody responses. In addition, Matrix-M1 adjuvant essentially eliminated the antigen dose-response, suggesting the potential for major antigen-sparing and consequent improved manufacturing efficiency and timeliness [Coleman 2017]. The Matrix-M1 adjuvant was also shown to enhance antibody, cellular, and protective immune responses in Balb/c mice administered Zaire ebolavirus (EBOV) GP vaccine with or without Matrix-M1 or aluminum phosphate adjuvants [Bengtsson 2016].

In addition, 3 GLP-compliant toxicology studies in NZW rabbits have been performed with 4 different antigens (influenza hemagglutinin [HA] ± respiratory syncytial virus [RSV] F, Zika virus envelope dimers [ZIKV EnvD], and EBOV GP), in which up to 100 µg Matrix-M1 adjuvant alone or with antigen was evaluated. These toxicological investigations indicated that baculovirus-Sf9-produced antigens (up to 240 µg total nanoparticle dose) with Matrix-M1 adjuvant (up to 100 µg) were well tolerated in the animals tested with no evidence of toxicity suggestive of any unusual risk or target organ for toxicity. Non-adverse findings, including local injection site inflammation, enlargement of the lymph nodes draining the injection sites, and elevated serum markers of inflammation (including C-reactive protein), were transient and were considered consistent with immune system stimulation consequent to immunization.

Further details are provided in the SARS-CoV-2 rS Investigator's Brochure (IB).

6.3 Clinical Summary

The first clinical study with SARS-CoV-2 rS nanoparticle vaccine is 2019nCoV-101, which is a 2-part, randomized, observer-blinded, placebo-controlled, Phase 1/2 trial. Part 1 (Phase 1) is designed to evaluate the immunogenicity and safety of SARS-CoV-2 rS nanoparticle vaccine with or without Matrix-M1 adjuvant in 131 healthy participants ≥ 18 to ≤ 59 years of age. Results of an interim analysis for the Phase 1 portion of the trial at Day 35 showed that SARS-CoV-2 rS with Matrix-M1 adjuvant was well tolerated and elicited robust immune responses. There were no serious adverse events (SAEs) or adverse events of special interest (AESIs) reported. Reactogenicity was mainly mild in severity and of short duration (mean ≤ 2 days), with second vaccinations inducing greater local and systemic reactogenicity. The adjuvant significantly enhanced immune responses (anti-S IgG, hACE2 receptor binding inhibition antibody, and neutralizing antibody) and was antigen dose-sparing; the 2-dose 5 µg SARS-CoV-2 rS/Matrix-M1 adjuvant induced mean anti-S IgG and neutralizing antibody responses that exceeded the mean responses in convalescent sera from COVID-19 patients with clinically significant illnesses. The vaccine also induced antigen-specific T cells with a largely Type 1 T helper (Th1) phenotype.

Part 2 (Phase 2) is designed to evaluate the immunogenicity, safety and preliminary efficacy of SARS-CoV-2 rS and Matrix-M1 adjuvant in up to 1,500 healthy adults ≥ 18 to ≤ 84 years of age with more co-morbidities than the participant population in Part 1 of the study. An interim 5-day reactogenicity analysis was conducted on 846 participants following the first dose of study vaccine to support initiation of the Phase 3 study. This analysis comprised 607 participants aged 18 to 59 years (the same age range of Part 1 of the study) and 239 participants aged 60 to 84 years, with data presented in masked groups to maintain the integrity of the study. Overall, local and systemic reactogenicity data from this analysis were consistent with the reactogenicity data in Part 1 of the study, with no safety concerns between the younger and older age cohorts. Both local and systemic reactogenicity events occurred less frequently in older adults.

Novavax has, in its internally sponsored clinical trials, tested baculovirus-Sf9-produced nanoparticle vaccines in 14,848 participants comprising older adults, young adults, and a limited number of children 2 to 5 years of age; and also including 3,075 pregnant women, with acceptable safety. Matrix-M1 adjuvant has been given to 4,311 humans (of which, approximately 2,657 humans received nanoparticle vaccine) with acceptable short-term reactogenicity and an unremarkable long-term safety profile.

Further details are provided in the SARS-CoV-2 rS IB.

6.4 Study Rationale

Both nonclinical and early clinical data to date support continued clinical development of SARS-CoV-2 rS and Matrix-M1 adjuvant as a potential vaccine against SARS-CoV-2. In rodent and nonhuman primate (NHP) challenge models, Matrix-M1 adjuvanted SARS-CoV-2 rS induced high titers of antibodies in a dose-dependent fashion, as measured against anti-spike protein and hACE2 receptor binding, achieved neutralization of wild-type virus that exceeded the magnitude of responses measured in human convalescent sera and provided protection against SARS-CoV-2 challenge [Tian 2020; Mandolesi 2020; Guebre-Xabier 2020]. Notably, in NHP studies, clinical doses of vaccine (5 and 25 µg SARS-CoV-2 rS/50-µg Matrix-M1) resulted in sterile immunity in the lungs and nasal passage following wild-type virus challenge, suggesting that the vaccine may both protect against upper and lower respiratory tract disease and interrupt transmission [Guebre-Xabier 2020].

Results from a Day 35 interim analysis of Part 1 (Phase 1) of Study 2019nCoV-101 indicate that in 131 healthy adult participants 18 to 59 years of age, 2-dose regimens of 5 and 25 µg SARS-CoV-2 rS/50 µg Matrix-M1 (on Days 0 and 21) were well tolerated and induced robust immune responses with high levels of neutralizing antibodies that closely correlated with anti-spike IgG [Keech 2020]. IgG and neutralizing antibody responses following the second dose of vaccine were very high, generally exceeded the titers seen in convalescent serum from hospitalized COVID-19 patients and exceeded overall convalescent sera geometric mean titers (GMTs) by 4-fold. The benefit of Matrix-M1 adjuvant was clear in the greater magnitude of humoral and T-cell response, induction of functional antibodies, and dose-sparing.

A Phase 2 clinical program is underway and will provide additional safety and immunogenicity results in older participants (> 60 years of age) and participants with co-morbidities. Reactogenicity data following the first dose indicate that the reactogenicity profile between adults 18 to 59 years and older adults ≥ 60 years are comparable, with older adults generally reporting solicited events less frequently. Combining the current nonclinical and clinical data with positive Phase 1/2 data provide the impetus for early initiation of the Phase 3 clinical development program in the context of the current public health pandemic crisis.

The purpose of this Phase 3 study is to evaluate the efficacy, safety and immunogenicity of SARS-CoV-2 rS with Matrix-M1 adjuvant in adult participants ≥ 18 years of age (**Adult Main Study**).

Clinical endpoints (see Sections 7.1.4 to 7.1.6) will be assessed overall, and within age subgroups, with the main age strata 18 to ≤ 64 years and ≥ 65 years. All participants will receive 2 doses of trial vaccine: 1 dose on each of Days 0 and 21 + 7 days. This schedule is based on clinical data from the Phase 1/2 clinical program. If another COVID-19 vaccine is demonstrated to be safe and efficacious and authorized by regulatory authorities, participants for whom the new vaccine is recommended and available will be counseled with respect to their options. Prior to the second dose of trial vaccine (Day 21), these participants may be offered the opportunity to be unblinded so that those who received placebo may receive the Emergency Use Authorization (EUA)-authorized vaccine, if they choose. Participants who are unblinded prior to the second dose of trial vaccine (Day 21) and receive EUA-authorized vaccine in this manner will be censored in the final efficacy analysis at the time of unblinding but will be strongly encouraged to remain in safety follow-up as defined in the protocol. Participants who, upon unblinding prior to 26 January 2021 for the purpose of receiving EUA-authorized vaccine, learn they had received active vaccine are censored at the time of unblinding but may receive the second dose of Novavax trial vaccine. Participants unblinded between the first (Day 0) and second (Day 21) dose of trial vaccine after 26 January 2021 will not be eligible to receive further investigational product on this protocol. Similarly, participants who choose to withdraw from the blinded trial after having received both doses of blinded study vaccine will not be eligible for blinded crossover.

A **Pediatric Expansion** is being added to evaluate the safety and effectiveness of SARS-CoV-2 rS with Matrix-M1 adjuvant in adolescents (12 to < 18 years).

6.5 Rationale for Dose Selection

Clinical doses of vaccine and adjuvant (5 and 25 µg SARS-CoV-2 rS adjuvanted with 50 µg Matrix-M1) administered in 2 doses resulted in sterilizing immunity in the lungs and nasal passage following wild-type virus challenge in NHP, suggesting that the vaccine may protect against both upper and lower respiratory tract disease and interrupt transmission [Guebre-Xabier 2020]. These dose levels are being evaluated in Part 1 of Study 2019nCoV-101 in 131 healthy adult participants 18 to 59 years of age and in Part 2 of Study 2019nCoV-101 in 750 to 1,500 participants 18 to 84 years of age, including participants with co-morbidities. Results from the Part 1 Day 35 interim analysis support either dose of SARS-CoV-2 rS/Matrix-M1 adjuvant in terms of safety and immunology, with the lower dose (5 µg) offering the advantage of dose-sparing [Keech 2020]. Based on the available nonclinical and Phase 1 data, the dose selected for the Phase 3 study is 5 µg SARS-CoV-2 rS/50 µg Matrix-M1 adjuvant administered as an intramuscular (IM) injection on Days 0 and 21. All vaccinations will be administered on an outpatient basis by designated study site personnel in a way to maintain the blind. Pharmacy management of unblinded product will be performed by unblinded study site personnel who may administer the study vaccine, if qualified to do so, but will not otherwise be involved in the study procedures or observation of participants.

6.6 Benefit – Risk Assessment

The SARS-CoV-2 rS nanoparticle vaccine contains purified protein antigens. It cannot replicate, the protein is not produced using infectious SARS-CoV-2, nor can the vaccine cause COVID-19. However, in common with all vaccines produced in cell culture or other systems, the SARS-CoV-2 rS nanoparticle vaccine contains residual non-vaccine proteins derived from the production system, and sensitization to these, or the SARS-CoV-2 S protein itself, may theoretically occur. While the occurrence of immediate hypersensitivity is possible with the administration of any vaccine, whether licensed or in development, no such reactions have been observed in any of the Sponsor's clinical trials to date. As clinical data become available with increased exposure, it is possible that this profile may change. The risk of AEs related to hypersensitivity will be mitigated by observation of participants for at least 30 minutes after each study vaccination.

The risk for enhanced COVID-19 in immunized participants is a theoretical risk. Enhanced disease in coronavirus vaccine-immunized animals after infectious virus challenge has been demonstrated in nonclinical studies of several, but not all, coronavirus vaccine candidates. There is currently no evidence for immunoenhancement in nonclinical testing of SARS-CoV-2 rS or other Novavax baculovirus-Sf9-based vaccines taken into nonclinical evaluation or clinical trials.

No risks have been identified in nonclinical or early clinical testing of SARS-CoV-2 or other coronavirus vaccines (SARS-CoV and MERS-CoV) developed using the baculovirus-Sf9 system to date. In supportive toxicology studies with other viral GP nanoparticle vaccines developed using the baculovirus-Sf9 system with different antigens, findings were generally consistent with an immune response to the vaccine formulations. These toxicological investigations indicated that baculovirus-Sf9-produced antigens (up to 240 µg total nanoparticle dose) with Matrix-M1 adjuvant (up to 100 µg) were well tolerated in the animal and antigen system tested with no evidence of toxicity suggestive of any unusual risk or target organ for toxicity. Non-adverse findings, including local injection site inflammation and serum chemical markers of inflammation (such as C-reactive protein), were transient and considered consistent with immune system stimulation consequent to immunization.

6.6.1 Risk Assessment

Details of risk-based monitoring are provided in the Monitoring Plan.

6.6.2 Overall Benefit – Risk Conclusion

Findings to date suggest that SARS-CoV-2 rS when administered with Matrix-M1 adjuvant demonstrated an acceptable safety profile in adult participants aged 18 to 84 years and a robust immunogenicity profile in healthy adult participants aged 18 to < 59 years. Results of an interim 5-day reactogenicity analysis on 846 participants aged 18 to 84 years in Part 2 of Study 2019nCoV-101 showed a similar reactogenicity profile between younger and older participants, with both local and systemic reactogenicity events occurring less frequently in older adults.

Novavax baculovirus-Sf9-produced nanoparticle vaccines comprising viral glycoproteins, with and without Matrix-M1 or aluminum adjuvants, have been shown to induce robust and protective immune responses in relevant animal models to influenza HAs, RSV F protein, SARS-CoV and MERS-CoV S proteins, rabies GP, and EBOV GP. In addition, the Novavax RSV F protein candidate adsorbed to aluminum phosphate has induced antibodies in pregnant women which, when transferred transplacentally, were associated with reduced rates of RSV lower respiratory tract infections in their infants during the first 90 to 180 days of life. The goal of this program is to investigate the efficacy, safety and immunogenicity of the SARS-CoV-2 rS vaccine with Matrix-M1 adjuvant in prevention of COVID-19.

Further details are provided in the SARS-CoV-2 rS IB.

7 STUDY OBJECTIVES, ENDPOINTS AND ESTIMANDS

7.1 Main Study

7.1.1 Primary Objective:

- To evaluate the efficacy of a 2-dose regimen of SARS-CoV-2 rS adjuvanted with Matrix-M1 compared to placebo against polymerase chain reaction (PCR)-confirmed symptomatic COVID-19 illness diagnosed ≥ 7 days after completion of the second injection in the initial set of vaccinations of adult participants ≥ 18 years of age.

7.1.2 Secondary Objectives:

7.1.2.1 Key Secondary Objective:

- To evaluate the efficacy of a 2-dose regimen of SARS-CoV-2 rS adjuvanted with Matrix-M1 compared to placebo against PCR-confirmed symptomatic COVID-19 illness due to a SARS-CoV-2 variant not considered as a "variant of concern / interest" according to the CDC Variants Classification, diagnosed ≥ 7 days after completion of the second injection in the initial set of vaccinations of adult participants ≥ 18 years of age.

7.1.2.2 Other Secondary Objectives:

- To evaluate the efficacy of a 2-dose regimen of SARS-CoV-2 rS adjuvanted with Matrix-M1 compared to placebo against PCR-confirmed moderate-to-severely symptomatic COVID-19 illness diagnosed ≥ 7 days after completion of the second vaccination in the initial set of vaccinations of adult participants ≥ 18 years of age.
- To assess vaccine efficacy (VE) against ANY symptomatic SARS-CoV-2 infection.
- To assess VE according to race and ethnicity.
- To assess VE in high-risk adults versus non-high-risk adults (high-risk is defined by age ≥ 65 years with or without co-morbidities or age < 65 years **with** co-morbidities [eg, obesity (body mass index [BMI] > 30 kg/m^2), chronic kidney or lung disease, cardiovascular disease and diabetes mellitus type 2) and/or by life circumstance [living or working conditions involving known frequent exposure to SARS-CoV-2 or to densely populated circumstances (eg, factory or meat packing plants, essential retail workers, etc)]).
- To assess the durability of vaccine efficacy (measured by all defined efficacy endpoints) in initial active vaccine recipients versus crossover (delayed) active vaccine recipients.
- To describe the humoral immune response to vaccine in terms of neutralizing antibody to SARS-CoV-2 for all Immunogenicity Population Participants, and for subsets with and without prior SARS-CoV-2 exposure determined by detectable anti-nucleoprotein (NP) antibodies at baseline.

- To assess the immune response to vaccine by IgG antibody to SARS-CoV-2 S protein and hACE2 inhibiting antibodies at Day 35 and later for all Immunogenicity Population participants, and for subsets with and without prior SARS-CoV-2 exposure determined by detectable anti- NP antibodies at baseline.
- To assess the durability of immune response (IgG antibody to SARS-CoV-2 S protein, hACE2 inhibition, and microneutralization [MN]) at 12, 18 and 24 months of study in all Immunogenicity Population participants, and for subsets with and without detectable anti-NP antibodies at baseline or prior to the crossover set of vaccinations.
- To describe and compare the safety experience for the vaccine versus placebo in adult participants ≥ 18 years of age based on solicited short-term reactogenicity by toxicity grade for 7 days following each vaccination (Days 0 and 21) after the initial set of vaccinations.
- To assess overall safety through 49 days (28 days after second injection of each set of vaccinations [initial and crossover]) and to compare vaccine versus placebo for all unsolicited AEs and medically attended adverse events (MAAEs).
- To assess the frequency and severity of MAAEs attributed to vaccine, AESIs, or SAEs through the end of study (EoS) and to compare vaccine versus placebo after each set of vaccinations (initial and crossover).
- To assess all-cause mortality in vaccine versus placebo recipients after each set of vaccinations (initial and crossover).
- To describe the severity and course of COVID-19 in vaccine versus placebo recipients in terms of healthcare requirements, utilization and medical assessments after each set of vaccinations (initial and crossover).
- To assess the proportion of participants (vaccine versus placebo recipients) with SARS-CoV-2 infection determined by anti-SARS-CoV-2 NP antibodies, including specifically asymptomatic infection, across the 2 years of study follow-up.
- To assess the VE against SARS-CoV-2 infection determined by anti-SARS-CoV-2 NP antibodies, regardless of whether the infection was symptomatic.
- To assess in a subset of participants the immunogenicity of a new lot of SARS-CoV-2 rS with Matrix-M1 adjuvant in comparison to the lot utilized in the initial set of vaccinations (ie, immunobridging).
- To assess non-inferiority of the neutralizing antibody response for adolescent participants seronegative to anti-SARS-CoV-2 NP antibodies at baseline, compared with that observed in seronegative adult participants 18 to < 26 years of age from the **Adult Main Study** (Immunogenicity Population participants before crossover).

7.1.3 Exploratory Objectives:

- To evaluate the efficacy of study vaccine compared to placebo against PCR-confirmed symptomatic COVID-19 illness due to a SARS-CoV-2 variant considered as a "variant of concern / interest" according to the CDC Variants Classification, diagnosed ≥ 7 days after completion of the second vaccination in the initial set of vaccinations of adult participants ≥ 18 years of age.
- To assess cell-mediated response:
  - Th1 or Type 2 T helper (Th2) predominance after initial set of vaccinations.
- To contribute to a larger cross-study National Institutes of Health (NIH) effort to define correlates of risk and protection against SARS-CoV-2 infection and disease.
- To assess impact of vaccination on nasal viral load in nasal swabs of participants who develop symptoms of possible COVID-19.
- To assess impact of vaccination on asymptomatic SARS-CoV-2 PCR positivity and viral load at the time of the crossover set of vaccinations.
- To describe sequences of the genetic material from SARS-CoV-2 detected in COVID-19 cases to evaluate possible viral mutations that may be associated with breakthrough infections.

7.1.4 Primary Endpoint:

First episode of PCR-positive mild, moderate, or severe COVID-19, where severity is defined as:

Mild COVID-19 (≥ 1 of the following):

- Fever (defined by subjective or objective measure, regardless of use of anti-pyretic medications)
- New onset cough
- ≥ 2 additional COVID-19 symptoms:
  - New onset or worsening of shortness of breath or difficulty breathing compared to baseline.
  - New onset fatigue.
  - New onset generalized muscle or body aches.
  - New onset headache.
  - New loss of taste or smell.
  - Acute onset of sore throat, congestion or runny nose.
  - New onset nausea, vomiting or diarrhea.

OR Moderate COVID-19 (≥ 1 of the following):

- High fever (≥ 38.4°C) for ≥ 3 days (regardless of use of anti-pyretic medications, need not be contiguous days).
- Any evidence of significant lower respiratory tract infection (LRTI):
  - Shortness of breath (or breathlessness or difficulty breathing) with or without exertion (greater than baseline).
  - Tachypnea: 24 to 29 breaths per minute at rest.
  - SpO_2: 94% to 95% on room air.
  - Abnormal chest X-ray or chest computerized tomography (CT) consistent with pneumonia or LRTI.
- Adventitious sounds on lung auscultation (eg, crackles/rales, wheeze, rhonchi, pleural rub, stridor).

OR Severe COVID-19 (≥ 1 of the following):

- Tachypnea: ≥ 30 breaths per minute at rest.
- Resting heart rate ≥ 125 beats per minute.
- SpO_2: ≤ 93% on room air or PaO_2/FiO_2 < 300 mmHg.
- High flow oxygen (O_2) therapy or non-invasive ventilation (NIV)/non-invasive positive pressure ventilation (NIPPV) (eg, continuous positive airway pressure [CPAP] or bilevel positive airway pressure [BiPAP]).
- Mechanical ventilation or extracorporeal membrane oxygenation (ECMO).
- One or more major organ system dysfunction or failure to be defined by diagnostic testing/clinical syndrome/interventions, including any of the following:
  - Acute respiratory failure, including acute respiratory distress syndrome (ARDS).
  - Acute renal failure.
  - Acute hepatic failure.
  - Acute right or left heart failure.
  - Septic or cardiogenic shock (with shock defined as systolic blood pressure [SBP] < 90 mm Hg OR diastolic blood pressure [DBP] < 60 mm Hg).
  - Acute stroke (ischemic or hemorrhagic).
  - Acute thrombotic event: acute myocardial infarction (AMI), deep vein thrombosis (DVT), pulmonary embolism (PE).
  - Requirement for: vasopressors, systemic corticosteroids, or hemodialysis.
- Admission to an intensive care unit (ICU).
- Death.

7.1.5 Secondary Endpoints:

7.1.5.1 Key Secondary Endpoint:

First episode of PCR-positive COVID-19, as defined under the primary endpoint, shown by gene sequencing to represent a variant not considered as a “variant of concern / interest” according to the CDC Variants Classification.

7.1.5.2 Other Secondary Endpoints:

- First episode of PCR-positive moderate or severe COVID-19, as defined under the primary endpoint.
- ANY symptomatic SARS-CoV-2 infection, defined as: PCR-positive nasal swab **and** ≥ 1 of any of the following symptoms:
  - Fever.

  - New onset cough.
  - New onset or worsening of shortness of breath or difficulty breathing compared to baseline.
  - New onset fatigue.
  - New onset generalized muscle or body aches.
  - New onset headache lasting.
  - New loss of taste or smell.
  - Acute onset of sore throat, congestion or runny nose.
  - New onset nausea, vomiting or diarrhea.
- Neutralizing antibody titers from Immunogenicity Population at Days 0, 35 and immediately prior to administration of the crossover set of vaccinations.
- Serum IgG levels to SARS-CoV-2 S protein, hACE2 inhibition titers from Immunogenicity Population at Days 0, 35 and immediately prior to administration of the crossover set of vaccinations.
- Serum IgG levels to SARS-CoV-2 S protein, MN and hACE2 inhibition titers from Immunogenicity Population at Months 12, 18 and 24.
- Description of course, treatment and severity of COVID-19 reported after a PCR-confirmed case via the Endpoint Form.
- Reactogenicity incidence and severity (mild, moderate or severe) recorded by all participants on their electronic patient-reported outcome diary application (eDiary) on days of vaccination and subsequent 6 days (total 7 days after each vaccine injection in the initial set of vaccinations).
  - Reactogenicity endpoints include injection site reactions:
    - Pain.
    - Tenderness.
    - Erythema.
    - Swelling/induration.
  - Systemic reactions:
    - Fever.
    - Malaise.
    - Fatigue.
    - Arthralgia.
    - Myalgia.

    - Headache.
    - Nausea/vomiting.

- Incidence and severity of MAAEs through 49 days, ie, 28 days after second injection of each set of vaccinations (initial and crossover).
- Incidence and severity of unsolicited AEs through 49 days, ie, 28 days after second injection of each set of vaccinations (initial and crossover).
- Incidence and severity of MAAEs attributed to study vaccine, SAEs and AESIs through Month 12.
- Incidence and severity of SAEs, MAAEs attributed to study vaccine and AESIs during Month 12 through Month 24 or the EoS.
- Death due to any cause.
- Data points to be collected for healthcare requirements, utilization and medical assessments from participants who become ill on study will be defined in a separate substudy protocol.
- Antibodies to SARS-CoV-2 NP at Days 0 and 35, immediately prior to administration of the crossover set of vaccinations, and at Months 12, 18 and 24 will be used to determine natural infection and to determine the incidence of asymptomatic infection acquired during study follow-up.
- Antibodies to SARS-CoV-2 NP, regardless of whether the infection was symptomatic.
- IgG antibodies to SARS-CoV-2 rS at approximately 35 days after the first crossover vaccination in approximately 300 active vaccine recipients 18 to ≤ 64 years of age enrolled at selected study sites.
- Neutralizing antibody response at Day 35 for all adolescent participants seronegative to anti-SARS-CoV-2 NP antibodies at baseline, compared with that observed in seronegative adult participants 18 to < 26 years of age from the **Adult Main Study** (Immunogenicity Population participants before crossover).

7.1.6 Exploratory Endpoints:

- First episode of PCR-positive COVID-19, as defined under the primary endpoint, shown by gene sequencing to represent a “variant of concern / interest” according to the CDC Variants Classification.
- Th1 or Th2 responses, eg, interleukin [IL]-2, IL-4, IL-5, IL-13, tumor necrosis factor alpha (TNF-α), interferon gamma (IFN-γ) in whole blood and/or harvested peripheral blood mononuclear cell (PBMCs) prior to and on Day 35 after the initial set of vaccinations.

- Serum samples from a designated subset of up to approximately 4,500 Immunogenicity Population participants to be transferred to National Institute of Allergy and Infectious Diseases (NIAID) for testing and analysis to determine correlates of risk and protection. Endpoints will be described in a separate statistical analysis plan (SAP) developed by external statistics groups (eg, COVID-19 Prevention Network [CoVPN], Operation Warp Speed [OWS]).
- Quantitative PCR tests may be performed on nasal swabs collected from this trial to assess whether vaccination impacts viral shedding.
- Quantitative PCR tests performed on nasal swabs collected immediately prior to administration of blinded crossover vaccination to assess impact of initial vaccination on frequency of asymptomatic SARS-CoV-2 infection and level of viral shedding.
- Next-generation sequencing of viral genomes detected in nasal swabs tested by PCR to describe the genetic evolution of circulating SARS-CoV-2 strains during the conduct of the study.

7.2 Pediatric Expansion

7.2.1 Primary Objective:

- To evaluate the efficacy of a 2-dose regimen of SARS-CoV-2 rS adjuvanted with Matrix-M1 compared to placebo against PCR-confirmed symptomatic COVID-19 illness diagnosed ≥ 7 days after completion of the second injection in the initial set of vaccinations of adolescent participants 12 to < 18 years of age.
- To describe the safety experience for the vaccine versus placebo in adolescent participants (12 to < 18 years of age) based on solicited short-term reactogenicity by toxicity grade for 7 days following each vaccination (Days 0 and 21) after the initial set of vaccinations.
- To assess overall safety through 49 days (28 days after second injection of each set of vaccinations [initial and crossover]) by comparing vaccine versus placebo for all unsolicited AEs and MAAEs.
- To assess the frequency and severity of MAAEs attributed to vaccine, AESIs, or SAEs through the EoS and to compare vaccine versus placebo after each set of vaccinations (initial and crossover).
- To assess all-cause mortality in vaccine versus placebo recipients after each set of vaccinations (initial and crossover).
- To assess non-inferiority of the neutralizing antibody response for all adolescent participants seronegative to anti-SARS-CoV-2 NP antibodies at baseline, compared with that observed in seronegative adult participants 18 to < 26 years of age from the **Adult Main Study** (Immunogenicity Population participants before crossover).

7.2.2 Secondary Objectives:

- To evaluate the efficacy of a 2-dose regimen of SARS-CoV-2 rS adjuvanted with Matrix-M1 compared to placebo against PCR-confirmed symptomatic COVID-19 illness due to SARS- CoV-2 variant not considered as a “variant of concern / interest” according to the CDC Variants Classification, diagnosed ≥ 7 days after completion of the second injection in the initial set of vaccinations of adolescent participants 12 to < 18 years of age.
- To evaluate the efficacy of a 2-dose regimen of SARS-CoV-2 rS adjuvanted with Matrix-M1 compared to placebo against PCR-confirmed moderate-to-severely symptomatic COVID-19 illness diagnosed ≥ 7 days after completion of the second vaccination in the initial set of vaccinations of adolescent participants 12 to < 18 years of age.
- To assess VE against ANY symptomatic SARS-CoV-2 infection.
- To assess VE according to race and ethnicity.
- To assess the durability of VE (measured by all defined efficacy endpoints) in adolescents after initial active vaccine recipients versus crossover (delayed) active vaccine recipients.
- To monitor occurrence and severity of COVID-19 cases by following participant-reported symptoms.
- To assess the neutralizing antibody response to SARS-CoV-2 for adolescent participants by subsets with and without anti-NP antibodies at baseline, compared with that observed in adult participants 18 to < 26 years of age from the **Adult Main Study** (Immunogenicity Population participants before crossover).
- To assess the anti-spike immunoglobulin G (IgG) antibody response and human angiotensin converting enzyme 2 (hACE2) inhibiting antibody response at Day 35 for adolescent participants by subsets with and without detectable anti-NP antibodies at baseline, compared with that observed in adult participants 18 to < 26 years of age from the **Adult Main Study** (Immunogenicity Population participants before crossover).
- To assess the durability of immune response (anti-rS IgG antibody, hACE2 inhibition, and microneutralization [MN] titers) at 12, 18 and 24 months of study in all adolescent participants, and for subsets with and without detectable anti-NP antibodies at baseline or prior to crossover set of vaccinations, with that observed in adult participants 18 to < 26 years of age from the **Adult Main Study** (Immunogenicity Population participants before crossover).
- To assess the proportion of adolescent participants (vaccine versus placebo recipients) with SARS-CoV-2 infection determined by anti-SARS-CoV-2 NP antibodies, including specifically asymptomatic infection, across the 2 years of study follow-up.

- To assess the VE against SARS-CoV-2 infection determined by anti-SARS-CoV-2 NP antibodies in adolescent participants, regardless of whether the infection was symptomatic.

7.2.3 Exploratory Objectives:

- To evaluate the efficacy of study vaccine compared to placebo against PCR-confirmed symptomatic COVID-19 illness due to a SARS- CoV-2 variant considered as a "variant of concern / interest" according to the CDC Variants Classification, diagnosed ≥ 7 days after completion of the second vaccination in the initial set of vaccinations of adolescent participants 12 to < 18 years of age.
- To assess cell-mediated response in a cohort of 50 adolescent participants at baseline and at Days **7 and 28:**
  - Type 1 T helper (Th1) or Type 2 T helper (Th2) predominance.
- To assess impact of vaccination on nasal viral load in nasal swabs of adolescent participants who develop symptoms of possible COVID-19.
- To assess impact of vaccination on asymptomatic SARS-CoV-2 PCR positivity and viral load in adolescent participants at the time of the crossover set of vaccinations.
- To describe sequences of the genetic material from SARS-CoV-2 viruses detected in COVID-19 cases in adolescent participants to evaluate possible viral mutations that may be associated with breakthrough infections.

7.2.4 Primary Endpoints:

First episode of PCR-positive mild, moderate, or severe COVID-19, where severity is defined as:

Mild COVID-19 (≥ 1 of the following):

- Fever (defined by subjective or objective measure, regardless of use of anti-pyretic medications)
- New onset cough
- ≥ 2 additional COVID-19 symptoms:
  - New onset or worsening of shortness of breath or difficulty breathing compared to baseline.
  - New onset fatigue.
  - New onset generalized muscle or body aches.
  - New onset headache.
  - New loss of taste or smell.
  - Acute onset of sore throat, congestion or runny nose.
  - New onset nausea, vomiting or diarrhea.

OR Moderate COVID-19 (≥ 1 of the following):

- High fever (≥ 38.4°C) for ≥ 3 days (regardless of use of anti-pyretic medications, need not be contiguous days).
- Any evidence of significant LRTI:
  - Shortness of breath (or breathlessness or difficulty breathing) with or without exertion (greater than baseline).
  - Tachypnea: 24 to 29 breaths per minute at rest.
  - SpO_2: 94% to 95% on room air.
  - Abnormal chest X-ray or chest CT consistent with pneumonia or LRTI.
- Adventitious sounds on lung auscultation (eg, crackles/rales, wheeze, rhonchi, pleural rub, stridor).

OR Severe COVID-19 (≥ 1 of the following):

- Tachypnea: ≥ 30 breaths per minute at rest.
- Resting heart rate ≥ 125 beats per minute.
- SpO_2: ≤ 93% on room air or PaO_2/FiO_2 < 300 mmHg.
- High flow oxygen (O_2) therapy or NIV/NIPPV (eg, CPAP or BiPAP).
- Mechanical ventilation or ECMO.
- One or more major organ system dysfunction or failure to be defined by diagnostic testing/clinical syndrome/interventions, including any of the following:
  - Acute respiratory failure, including ARDS.
  - Acute renal failure.
  - Acute hepatic failure.
  - Acute right or left heart failure.
  - Septic or cardiogenic shock (with shock defined as SBP < 90 mm Hg OR DBP < 60 mm Hg).
  - Acute stroke (ischemic or hemorrhagic).
  - Acute thrombotic event: AMI, DVT, PE.
  - Requirement for: vasopressors, systemic corticosteroids, or hemodialysis.
- Multisystem Inflammatory Syndrome in Children (MIS-C), as per the CDC definition:
  - An individual aged <21 years presenting with fever (>38.0°C for ≥24 hours, or report of subjective fever lasting ≥24 hours), laboratory evidence of inflammation (including, but not limited to, one or more of the following: an elevated C-reactive protein (CRP), erythrocyte sedimentation rate (ESR), fibrinogen, procalcitonin, d-dimer, ferritin, lactic acid dehydrogenase (LDH), or interleukin 6 (IL-6), elevated neutrophils, reduced

lymphocytes and low albumin), and evidence of clinically severe illness requiring hospitalization, with multisystem (>2) organ involvement (cardiac, renal, respiratory, hematologic, gastrointestinal, dermatologic or neurological); AND

  - No alternative plausible diagnoses; AND
  - Positive for current or recent SARS-CoV-2 infection by RT-PCR, serology, or antigen test; or COVID-19 exposure within the 4 weeks prior to the onset of symptoms.
- Admission to an ICU.
- Death

7.2.5 Safety Endpoints

- Reactogenicity incidence, duration and severity (mild, moderate or severe) recorded by parent(s)/caregiver(s) on their electronic patient-reported outcome diary application (eDiary) on days of vaccination and subsequent 6 days (total 7 days after each vaccine injection in the initial set of vaccinations).
  - Reactogenicity endpoints include injection site reactions:
    - Pain.
    - Tenderness.
    - Erythema.
    - Swelling/induration.
  - Systemic reactions:
    - Fever.
    - Malaise.
    - Fatigue.
    - Arthralgia.
    - Myalgia.
    - Headache.
    - Nausea/vomiting.
- Incidence and severity of MAAEs through 49 days, ie, 28 days after second injection of each set of vaccinations (initial and crossover).
- Incidence and severity of unsolicited AEs through 49 days, ie, 28 days after second injection of each set of vaccinations (initial and crossover).
- Incidence and severity of MAAEs attributed to study vaccine, SAEs and AESIs through Month 12.
- Incidence and severity of SAEs (including COVID-19 diagnoses), MAAEs attributed to study vaccine and AESIs during Month 12 through Month 24 or the EoS.

- Death due to any cause.

7.2.6 Effectiveness Endpoint:

- Neutralizing antibody response at Day 35 for all adolescent participants seronegative to anti-SARS-CoV-2 NP antibodies at baseline, compared with that observed in seronegative adult participants 18 to < 26 years of age from the **Adult Main Study** (Immunogenicity Population participants before crossover).

7.2.7 Secondary Endpoints:

- First episode of PCR-positive COVID-19, as defined under the primary endpoint, shown by gene sequencing to represent a variant not considered as a "variant of concern/ interest" according to the CDC Variants Classification.
- First episode of PCR-positive moderate or severe COVID-19, as defined under the primary endpoint.
- ANY symptomatic SARS-CoV-2 infection, defined as: PCR-positive nasal swab **and** $\geq$ 1 of any of the following symptoms:
  - Fever
  - New Onset cough
  - New onset or worsening of shortness of breath or difficulty breathing compared to baseline
  - New onset fatigue
  - New onset generalized muscle or body aches.
  - New onset headache.
  - New loss of taste or smell.
  - Acute onset of sore throat, congestion or runny nose.
  - New onset nausea, vomiting or diarrhea.
- Proportion of adolescent participants reporting SARS-CoV-2 infection (COVID-19) from Day 28 through end of Year 1, with severity classification as defined in the **Adult Main Study** (mild, moderate, or severe).
- Neutralizing antibody response at Day 35 for adolescent participants by age strata with and without anti-SARS-CoV-2 NP antibodies at baseline, compared with that observed in adult participants 18 to < 26 years of age from the **Adult Main Study** (Immunogenicity Population participants before crossover).
- Antibodies to SARS-CoV-2 NP at Days 0 and 35, and at Months 12, 18 and 24 will be used to determine natural infection and to determine the incidence of asymptomatic infection acquired during study follow-up.

- Serum IgG levels to SARS-CoV-2 S protein, hACE2 inhibition titers 14 days after second injection of the initial vaccination series (Day 35) in adolescent participants and subsets with and without anti-NP antibodies at baseline.
- Serum IgG levels to SARS-CoV-2 S protein, MN and hACE2 inhibition titers at Months 12, 18 and 24.
- Description of course, treatment and severity of COVID-19 reported after a PCR-confirmed case via the Endpoint Form.
- Antibodies to SARS-CoV-2 NP, regardless of whether the infection was symptomatic.

7.2.8 Exploratory Endpoints:

- First episode of PCR-positive COVID-19, as defined under the primary endpoint, shown by gene sequencing to represent a “variant of concern / interest” according to the CDC Variants Classification.
- Th1 or Th2 responses, eg, interleukin [IL]-2, IL-4, IL 5, IL-13, tumor necrosis factor alpha (TNF-α), interferon gamma (IFN-γ) in whole blood and/or harvested peripheral blood mononuclear cell (PBMCs).
- Quantitative PCR tests may be performed on nasal swabs collected from this trial to assess whether vaccination impacts viral shedding.
- Quantitative PCR tests performed on nasal swabs collected immediately prior to administration of blinded crossover vaccination to assess impact of initial vaccination on frequency of asymptomatic SARS-CoV-2 infection and level of viral shedding.
- Next-generation sequencing of viral genomes detected in nasal swabs tested by PCR to describe the genetic evolution of circulating SARS-CoV-2 strains during the conduct of the study.

8 OVERALL STUDY DESIGN AND PLAN

8.1 Study Design Description

This is a Phase 3, randomized, observer-blinded, placebo-controlled study to evaluate the efficacy, safety and immunogenicity of SARS-CoV-2 rS with Matrix-M1 adjuvant in adult participants ≥ 18 years of age (**Adult Main Study**) with a **Pediatric Expansion**. In the **Adult Main Study**, participants will be stratified by age group, and enrollment will occur concurrently within the 2 age strata, 18 to ≤ 64 years and ≥ 65 years. In the **Pediatric Expansion**, adolescent participants 12 to < 18 years of age will be enrolled without further stratification.

The study will be a multicenter, global study with countries selected based on the expected COVID-19 epidemiology and healthcare system characteristics.

8.1.1 Enrollment

In the **Adult Main Study**, most study participants are expected to be enrolled in the United States (US). **Prioritization will be given to enrollment of individuals at high risk for COVID-19 by virtue of Black/African American or Native American race, Latinx ethnicity, co-morbid conditions (eg, obesity [BMI > 30 kg/m^2], chronic kidney or lung disease, cardiovascular disease and diabetes mellitus type 2) and life circumstances [living or working conditions involving known frequent exposure to SARS-CoV-2 or to densely populated circumstances [eg, factory or meat packing plants, essential retail workers, etc]).** (See Section 8.6 Recruitment and Retention for guidelines with respect to high-risk characteristics.) Consideration will be paid to the enrollment of participants for whom vaccines authorized for Emergency Use are not, or not anticipated to be, recommended or available during the early months of this trial.

In the **Pediatric Expansion**, participants will only be enrolled in the US. All efforts will be made to enroll similar numbers of participants in the subgroups 12 to < 15 years of age, and 15 to < 18 years of age. As with the Adult Main Study, an effort will be made to recruit adolescents of a diverse population including underserved minorities. As with the Adult Main Study, an effort will be made to recruit adolescents of a diverse population including underserved minorities. Enrollment of the full adolescent cohort of participants (12 to < 18 years) will be contingent upon the review of early safety data (ie, 7 days of reactogenicity and overall safety post-dose 1) to be reviewed in the first ~60 enrolled adolescents (randomized in a 2:1 ratio to receive 5 µg SARS-CoV-2 rS adjuvanted with 50 µg Matrix-M1 or placebo) before enrollment of the remainder of the adolescent participants (N=~2,940). Likewise, administration receipt of the second vaccine dose to the full participant population will be contingent upon the review of early safety data (ie, 7 days of reactogenicity and overall safety post-dose 2) in the first ~60 enrolled adolescents before dosing the remainder of the adolescent participants. Safety data will be provided to and reviewed by the Data and Safety Monitoring Board (DSMB) after each early safety data review period (ie 7 days after each vaccination in the sentinel group). Simultaneously, safety data will be reviewed internally by the Sponsor.

After signing the informed consent form (ICF), including consent for the use of samples for future testing, potential participants may be screened within a window of up to 30 days (**Adult Main Study**) or 7 days (**Pediatric Expansion**), although it is expected that the majority of participants will be consented, randomized and vaccinated on Day 0.

In the **Adult Main Study**, a total of up to approximately 30,000 participants ≥ 18 years of age will be assigned to their respective age stratum with a goal of no more than 3:1 representation in the 18 to ≤ 64 years : ≥ 65 years groups. Participants will then be randomized in a 2:1 ratio via block randomization to receive 2 IM injections of SARS-CoV-2 rS + Matrix-M1 adjuvant or normal saline placebo as described in Table 3.

Study sites will be selected in the US and globally to ensure a diverse study population with respect to Black/African American or Native American race, Latinx ethnicity and age. Significant effort will be made to work with community engagement resources to ensure enrollment of underserved minorities. Consideration will be paid to the enrollment of participants for whom vaccines authorized for Emergency Use are not, or not anticipated to be, recommended or available during the early months of this trial.

While enrollment will be structured to strive for at least 25% of participants are ≥ 65 years of age, every effort will be made, due to the epidemiology of complications of COVID-19, to prioritize the enrollment of participants ≥ 65 years of age. Similarly, efforts will be made to prioritize the enrollment of participants < 65 years of age with co-morbidities (eg, obesity [BMI > 30 kg/m^2], chronic kidney or lung disease, cardiovascular disease and diabetes mellitus type 2), who are at higher risk of complications due to COVID-19. Participants will also be considered at high risk if their living or working conditions involve known frequent exposure to SARS-CoV-2 or to densely populated circumstances (eg, factory or meat packing plants, essential retail workers, etc). Consideration will be paid to the enrollment of participants for whom vaccines authorized for Emergency Use are not, or not anticipated to be, recommended or available during the early months of this trial.

Table 3 Adult Main Study - Trial Vaccine Groups

Trial Vaccine Group	Estimated Number of Randomized Participants[1]	Vaccination Periods			
		Initial		Crossover[2]	
		2 Vaccinations		2 Vaccinations	
		Day 0	Day 21 (+ 7 days)	Day 0	Day 21 (+ 7 days)
SARS-CoV-2 rS (5 µg) + Matrix-M1 adjuvant (50 µg)	N up to 20,000	Active vaccine	Active vaccine	Placebo	Placebo
	18 to ≤ 64 years: ≤ 15,000<br>≥ 65 years: ≥ 5,000				
Placebo (normal saline)	N up to 10,000	Placebo	Placebo	Active vaccine	Active vaccine
	18 to ≤ 64 years: ≤ 7,500<br>≥ 65 years: ≥ 2,500				

Abbreviations: COVID-19 = coronavirus disease 2019; EUA = Emergency Use Authorization.

1. Availability of other COVID-19 vaccines under EUA may impact the possibility of enrolling certain age categories.
2. Following accrual of sufficient efficacy and safety data to support application for EUA, participants will be scheduled for administration of 2 injections of the alternate study material 21 days apart ("blinded crossover").

In the **Pediatric Expansion**, a total of up to approximately 3,000 adolescent participants (12 to < 18 years of age) will be enrolled. Participants will then be randomized in a 2:1 ratio via block randomization to receive 2 IM injections of SARS-CoV-2 rS + Matrix-M1 adjuvant or normal saline placebo as described in Table 4.

Table 4 Pediatric Expansion - Trial Vaccine Groups

Trial Vaccine Group	Estimated Number of Randomized Participants	Vaccination Periods			
		Initial		Crossover[1]	
		2 Vaccinations		2 Vaccinations	
		Day 0	Day 21 (+ 7 days)	Day 0	Day 21 (+ 7 days)
SARS-CoV-2 rS (5 µg) + Matrix-M1 adjuvant (50 µg)	12 to < 18 years: 2,000	Active vaccine	Active vaccine	Placebo	Placebo
Placebo (normal saline)	12 to < 18 years: 1,000	Placebo	Placebo	Active vaccine	Active vaccine

1. Blinded crossover will occur ~6 months after the completion of the initial set of vaccinations.

8.1.2 Vaccine Administration

In both the **Adult Main Study** and **Pediatric Expansion**, study vaccination regimens will comprise 2 IM injections (Day 0 and Day 21 + 7 days), with the trial vaccine assigned in a full dose injection volume of 0.5 mL. The dose level selected for evaluation is 5 µg SARS-CoV-2 rS adjuvanted with 50 µg Matrix-M1 based on optimal safety and immunogenicity observed in nonclinical and early clinical data. All vaccinations will be administered on an outpatient basis by qualified vaccine administrators in a way to maintain the blind as described in the Pharmacy Manual. Unblinded product will be managed by unblinded study site personnel who may administer study vaccine, if qualified to do so, but will not otherwise be involved in the study procedures or observations of participants.

In the **Adult Main Study**, following collection of sufficient safety data to support application for EUA, participants will be scheduled for administration of 2 injections of the alternate study material 21 days apart ("blinded crossover"). That is, initial recipients of placebo will receive SARS-CoV-2 rS with Matrix-M1 adjuvant and initial recipients of SARS-CoV-2 rS with Matrix-M1 adjuvant will receive placebo. The same procedure for vaccine administration followed for the initial set of vaccinations will be followed at the time of the blinded crossover to ensure that the integrity of the blinded study is maintained.

In the **Pediatric Expansion**, following ~6 months of follow-up after the completion of the initial set of vaccinations (or earlier depending upon feasibility, EUA vaccine availability and the

dynamics of the pandemic), pediatric participants will be scheduled for administration of 2 injections of the alternate study material 21 days apart ("blinded crossover"). That is, initial recipients of placebo will receive SARS-CoV-2 rS with Matrix-M1 adjuvant and initial recipients of active vaccine will receive placebo. The same procedure for vaccine administration followed for the initial set of vaccinations will be followed at the time of the blinded crossover to ensure that the integrity of the blinded study is maintained.

Participants in the **Pediatric Expansion** who are unblinded between the first (Day 0) and second (Day 21) dose of trial vaccine will not be eligible to receive further investigational product on this protocol. Similarly, participants who choose to withdraw from the blinded trial after having received both doses of blinded study vaccine will not be eligible for blinded crossover. Safety follow-up of these participants will include remote contacts (eg, phone, email or text) to collect SAE, MAAE, AESI and COVID-19 diagnoses at the time points indicated [see Table 2 for the **Pediatric Expansion**] or at regular intervals if visits are not mandated. Receipt of another COVID-19 vaccine under EUA and date of administration should be recorded as a concomitant medication.

8.1.3 Safety Monitoring

In both the **Adult Main Study** and **Pediatric Expansion**, solicited AEs of reactogenicity after the initial set of vaccinations will be collected via participant (**Adult Main Study**) or parent(s)/caregiver(s) (**Pediatric Expansion**) reporting in the eDiary utilizing a smartphone application (see Section 10.4.3 for details). Participants or parent(s)/caregiver(s) who do not possess an appropriate device will be provided a device compatible with study requirements. All participants will be trained on the use of these applications at the initiation of their participation in the study (Day 0), and a Help Desk will be available 24 hours 7 days a week (24/7) for technical issues. For data entry issues, participants and parent(s)/caregiver(s) should contact the study site.

In the **Adult Main Study**, overall safety assessments, to be collected via the eDiary, from Day 0 through the first 12 months of follow-up after the initial set of vaccinations will include participant-recorded solicited (local and systemic reactogenicity) events through 7 days following each injection in the initial set of vaccinations; unsolicited AEs and MAAEs will be collected through 49 days, ie, 28 days after second injection of the initial and crossover sets of vaccinations. MAAEs attributed to vaccine, AESIs, SAEs and investigator-assessed targeted physical examination findings, including vital sign measurements will be collected as specified through Month 12. Safety follow-up phone calls will be conducted at 3 and 6 months (±30 days) post-crossover to collect MAAEs attributed to vaccine, AESIs, and SAEs in all participants that received crossover vaccinations. During the second 12 months of follow-up after the initial set of vaccinations, participants will be queried every 3 months via remote contacts (ie, Months 15 and 21) or during the scheduled visits (ie, Months 12, 18, and 24) regarding MAAEs attributed to study vaccine, AESIs and SAEs.

In the **Pediatric Expansion**, overall safety assessments from Day 0 through the first 12 months of follow-up after the initial set of vaccinations will include parent(s)/caregiver(s)-recorded solicited (local and systemic reactogenicity) events through 7 days following each injection in the initial set of vaccinations collected via eDiary. Unsolicited AEs and MAAEs will be collected through 49 days, ie, 28 days after second injection of the initial and crossover sets of vaccinations and MAAEs attributed to vaccine, AESIs, and SAEs will be recorded by the parent/caregiver on the memory aid and reported to the sites by weekly remote contact with the parent(s)/caregiver(s) through Month 12, and every 3 months via remote contacts (ie, Months 15 and 21) or during the scheduled visits (ie, Months 12, 18, and 24) during the second year (total of 24 months).

8.1.4 Immunogenicity Testing

In the **Adult Main Study**, blood samples for serologic assessments (anti-NP antibodies, IgG antibody to SARS-CoV-2 S protein, MN, and hACE2 inhibition) will be collected from all participants before the first vaccination and at selected subsequent time points, including prior to the crossover set of vaccinations. Immune responses immediately following vaccinations will be obtained from all participants after the initial set of vaccinations but not after the crossover vaccinations. Testing will be performed on a subset of collected sera from the Immunogenicity Population of up to approximately 1,200 adult participants from the active and placebo treatment groups split approximately evenly across the two age categories designated at random by biostatisticians who are blinded to treatment assignment. Blood samples will be obtained approximately 14 days after the second crossover vaccination dose from approximately 900 participants 18 to ≤ 64 years of age at selected study sites to compare immunogenicity of the vaccine lot utilized for the crossover vaccination period to that of the earlier lot used for the initial vaccination period.

In the **Pediatric Expansion**, blood samples for serologic assessments (anti-NP antibodies, IgG antibody to SARS-CoV-2 S protein, MN, and hACE2 inhibition) will be collected from all pediatric participants before the initial set of vaccinations and at selected subsequent time points thereafter, including immediately prior to the crossover set of vaccinations. Immune responses immediately following vaccinations will be obtained from all participants after the initial set of vaccinations but not immediately after the crossover vaccinations (Visit C2). Persistence of the immune responses will be measured at 12, 18, and 24 months after the initial set of vaccinations. Testing will be performed on all adolescent participants. In summary, there are 7 scheduled blood draws in pediatric study participants for immunogenicity assessments at the Day 0, 21, 35, C1 (Day 180), and Months 12, 18, and 24 visits, and during unscheduled visits (ie, Acute illness, Convalescent, and General). For participants in the CMI cohort only (N=50), additional blood will be collected to obtain PBMCs at the Day 0, 7 and 28 visits. Both adult and pediatric participants who test positive for COVID-19 anti-NP antibodies and/or PCR-positive nasal swab at baseline, indicating previous SARS-CoV-2 infection, will have subsequent SARS-CoV-2 S protein immune responses analyzed but will not contribute to the primary efficacy/effectiveness analyses. Those

who test positive immediately prior to the crossover vaccination series may contribute to the immunogenicity analyses at Months 12, 18 and 24. Immediate immunogenicity analyses will not be performed subsequent to the crossover vaccination period. Results from the anti-NP positive and/or PCR-positive nasal swab participants prior to either sets of vaccinations will be assessed and reported separately. In the **Adult Main Study**, whole blood samples for PBMC will be collected at Days 0, 21 and 35 from a small subset of participants (< 100) representing both age strata and reasonably reflecting the demographic subgroups enrolled at selected study site(s) with the capacity to isolate PBMCs. In the **Pediatric Expansion**, whole blood samples for PBMC will be collected at Days 0, 7 and 28 from a small subset comprised of 50 adolescent participants enrolled at selected study site(s) with the capacity to isolate PBMCs. These cells will be evaluated for cell-mediated immune responses to the initial set of vaccinations.

8.1.5 Prospective Surveillance of COVID-19

Prospective surveillance of COVID-19 will be conducted in the **Adult Main Study** and the **Pediatric Expansion**. For prospective surveillance in the **Adult Main Study**, participants will be provided with an oral thermometer on Day 0 and instructed to monitor their body temperature daily throughout the first 12 months of the study and to record temperature and any other relevant symptoms daily in their eDiary (see Section 10.4.3 for details). Participants who are noted during regular monitoring of the daily eDiary entries to not have reported temperature and symptoms for ≥ 7 days will be contacted by phone to assess clinical status and maintain engagement in the study. Electronic data capture (EDC) will be accomplished with an application installed on the participants' smartphone. Study participants who do not own smartphones compatible with these systems will be provided a device compatible with the applications. All participants will be trained on the use of these applications at the initiation of the study, and a Help Desk will be available 24/7 for technical issues. For data entry issues, participants should contact the study site.

Starting on Day 4, throughout the first 12 months of the study, when fever or other specified symptoms (see Table 5 for symptoms suggestive of COVID-19) are reported in the eDiary for at least 2 consecutive days for the same symptom, participants will be directed via the eDiary to begin daily nasal self-swabbing for PCR testing within 3 days of symptom onset at home for a total of 3 days and to initiate daily completion of the InFLUenza Patient-Reported Outcome (FLU-PRO) symptom reporting instrument for 10 days after COVID-19 symptom onset or until the participant experiences 2 consecutive asymptomatic days. Participants will be instructed at their enrollment visit on the methods of nasal self-swabbing for COVID-19 and completion of the FLU-PRO symptom reporting instrument. In addition, the eDiary will alert the study site to contact the participant to schedule the in-person Unscheduled Acute Illness Visit. During the first 4 days after the second vaccination when solicited systemic reactogenicity symptoms may be similar to those of COVID-19, investigators should use their clinical judgement to decide if an Unscheduled Acute Illness visit is warranted. Active surveillance for COVID-19 will continue after the blinded

crossover through the first 12 months of study. Passive surveillance of safety and efficacy via remote contacts or the scheduled visits will continue during Months 12 to 24.

After the first day of home nasal swabbing, repeat nasal self-swabs should be obtained daily for a total of 3 days to ensure capture of intermittent shedding. The self-swabs obtained by the participant should be maintained according to directions provided in the 3-swab kit, and the designated courier should be contacted to pick up the kit for shipping to the central lab, as directed.

At the in-person Unscheduled Acute Illness Visit, participants will be queried regarding AE symptoms, concomitant medications taken for these symptoms, undergo a targeted physical examination (to include O_2 saturation and respiratory rate), as indicated by the participant's signs and symptoms, and have obtained by the study personnel a medically attended nasal swab, a blood sample for serologic testing and be trained on the use of the portable pulse oximeter that they will take home with them. Medically attended swabs collected at the Unscheduled Acute Illness Visit will be processed at the study site for shipment to the central laboratory according to established procedures as described in the Laboratory Manual. Other clinical or laboratory evaluations that may be performed at the discretion of study personnel to inform need for isolation or additional medical care will be outside the scope of the study protocol.

Completion of the FLU-PRO reporting instrument and O_2 saturation (at rest and following mild exercise, defined as walking around the room for 1 minute) will be captured daily in the eDiary for 10 days after COVID-19 symptom onset or until the participant experiences 2 consecutive asymptomatic days.

Study participants whose home nasal self-swab and/or medically attended nasal swabs are confirmed at the central laboratory to be PCR-positive for SARS-CoV-2 at the Unscheduled Acute Illness Visit will be contacted by the study site to arrange an Unscheduled Convalescent Visit. The Unscheduled Convalescent Visit will occur approximately 1 month (or as soon thereafter, as feasible) after the onset of the PCR-confirmed case of COVID-19 at the Unscheduled Acute Illness Visit to assess status of AEs, record the clinical course of the disease on the Endpoint Form and obtain a blood sample for convalescent serologic testing. Pulse oximeters should be returned to the study site at this visit.

For the **Pediatric Expansion**, starting on Day 4, throughout the first 12 months of the study, parent(s)/caregiver(s) of participants will be asked to report symptoms of COVID-19 to the site as soon as possible after symptoms onset, or during the weekly remote contact. Fever and other symptoms of COVID-19 (including date of onset, duration, etc.) will be collected in a paper memory aid and will be reported to the sites by the parent(s)/caregiver(s) either as a spontaneous phone call or during the weekly remote contact during the first 12 months. If the parent(s)/caregiver(s) report symptoms compatible with COVID-19 by spontaneous contact or during the weekly contact, the study site will schedule an in-person Unscheduled Acute Illness Visit. During the first 4 days after vaccination when solicited systemic reactogenicity symptoms

may be similar to those of COVID-19, investigators should use their clinical judgement to decide if an Unscheduled Acute Illness visit is warranted. Active surveillance for COVID-19 will continue after the blinded crossover through the first 12 months of study. Passive surveillance of safety and efficacy via remote contacts or the scheduled visits will continue during Months 12 to 24. An eDiary will be used for the collection of 7-day reactogenicity data after each initial vaccination, however, it will not include a prompt to complete the FLU-PRO instrument or prompts for self-swabbing as these are not collected for the adolescent participants. An eDiary collection of reactogenicity will not be performed after crossover vaccinations. Prospective surveillance will continue via weekly phone calls after blinded crossover until Month 12, and will be conducted at 3 and 6 months (±30 days) post-crossover from Month 12 to Month 24.

At the in-person Unscheduled Acute Illness Visit, participants will be queried regarding AE symptoms, concomitant medications taken for these symptoms, undergo a targeted physical examination (to include oxygen [O_2] saturation and respiratory rate), as indicated by the participant's signs and symptoms, and have obtained by the study personnel a medically attended nasal swab, and a blood sample for serologic testing. No portable pulse oximeter will be distributed for home use for adolescent participants and there will be no self-swab collection in the adolescent participants. Medically attended swabs collected at the Unscheduled Acute Illness Visit will be processed at the study site for shipment to the central laboratory according to established procedures as described in the Laboratory Manual. Other clinical or laboratory evaluations that may be performed at the discretion of study personnel to inform need for isolation or additional medical care will be outside the scope of the study protocol.

Study participants whose medically attended nasal swabs are confirmed at the central laboratory to be PCR-positive for SARS-CoV-2 at the Unscheduled Acute Illness Visit will be contacted by the study site to arrange an Unscheduled Convalescent Visit. The Unscheduled Convalescent Visit will occur approximately 1 month (or as soon thereafter, as feasible) after the onset of the PCR-confirmed case of COVID-19 at the Unscheduled Acute Illness Visit to assess status of AEs, record the clinical course of the disease on the Endpoint Form and obtain a blood sample for convalescent serologic testing. An acute illness clinically consistent with COVID-19 should be suspected based on the presence of any of the symptoms enumerated below (Table 5) that are reported for at least 2 consecutive days and, as more specifically described in the primary endpoint (Section 7.1.4). In the case of rapid decompensation to a severe COVID-19 case, hospital or post-mortem data can be used for virologic confirmation of positive cases, but every effort should be made to send a duplicate swab to the central lab for the study to be included in the primary analysis. Hospitalization records and/or data should be obtained as feasible to ensure adequate characterization of the severity of COVID-19.

Table 5 Symptoms Suggestive of COVID-19

Symptoms Suggestive of COVID-19
Fever (body temperature > 38.0° C, in the absence of other symptoms) or chills
New onset or worsening of cough compared with baseline
New onset or worsening of shortness of breath or difficulty breathing over baseline
New onset fatigue
New onset generalized muscle or body aches
New onset headache
New loss of taste or smell
Acute onset sore throat
Acute onset congestion or runny nose
New onset nausea or vomiting
New onset of diarrhea

An in-person, unscheduled visit to the study site or at the participant's home may replace remote reporting and testing, if medically indicated and acceptable based on the ongoing pandemic and participant containment requirements and participant consent. Participants who have failed to complete their daily temperature and/or symptom reports in their eDiary for ≥ 7 days will receive a call from the study site to remind them to collect illness symptoms.

Endpoint collection will be obtained using hospital-derived information as reported on SAE forms or, if possible, electronic medical records. Consent for access to hospital records and data will be obtained at the time of entry into the study.

Modifications to follow-up procedures to comply with evolving regulations and recommendations due to the ongoing pandemic will be incorporated as needed to ensure appropriate data collection while maintaining health and safety of participants, communities and study personnel.

8.1.6 Periods of Adult Main Study

The **Adult Main Study** will consist of a screening period up to 30 days prior to Day 0); initial vaccination days (Days 0 and 21 + 7 days); outpatient study visits on Days 0, 21 (+ 7 days), and 35 (+ 7 days) in the initial set of vaccinations. Additional subsequent study visits for blood draws and the crossover set of vaccinations (approximately 21 days apart) will occur after acquisition of safety data on SARS-CoV-2 rS with Matrix-M1 adjuvant sufficient to support application for EUA in adults.

Adult participants who experience a PCR+ episode of COVID-19, regardless of whether their illness represents an endpoint, will be eligible to receive both the initial and crossover sets of vaccinations if they remain in blinded follow-up. Study visits subsequent to the crossover scheduled at Months 12 (± 15 days), 18 (± 30 days) and 24 (± 30 days) after the initial set of

vaccinations will be performed to assess the durability of immune response, taking into account when participants received active vaccine (initial or crossover).

In addition to the aforementioned Unscheduled Acute Illness and Unscheduled Convalescent Visits in the **Adult Main Study**, an Unscheduled General Visit may be conducted by study personnel in the event of a general medical issue other than COVID-19 symptomatology, eg, reconsent participants to amended Consent Form, if needed and remote contact for safety follow-up may be conducted approximately every 3 months after crossover if no other visits have been required. An EoS visit will be recorded for all study participants at approximately 24 months (± 30 days) after their initial set of vaccinations or at their last visit on study. Should participants decide to terminate early, an EoS telephone or in-person visit will occur to collect the maximum safety data and blood sample, if possible. All study participants who choose to be unblinded or to withdraw from active surveillance will be encouraged to continue in follow-up for safety and reported COVID-19 cases via remote contacts with investigative site personnel.

Participants that requested to be unblinded and/or are continuing in the study for Safety Follow-up will have visits replaced by remote contact (phone, email, text), at a minimum, every 90 days (± 30 days) from last dose received during the first year of participation. During the second year (ie, Months 12 to 24), contact will be continued every 180 days (± 30 days). **No eDiary, nasal swabs or blood samples will be collected during safety follow-up.** MAAEs, SAEs, AESI, and COVID-19 diagnoses will be collected during the remote contacts at the time points specified in Table 1. Receipt of another COVID-19 vaccine used under EUA should be recorded as a concomitant medication. The End of Study page is not to be completed for these participants until the end of their participation in the study.

8.1.7 Periods of Pediatric Expansion

The **Pediatric Expansion** will consist of a screening period (up to 7 days prior to Day 0); initial vaccination days (Days 0 and 21 + 7 days); and outpatient study visits on Days 0, 21 (+ 7 days), and 35 (+ 7 days) in the initial set of vaccinations. A sentinel group of 60 adolescents (15 to < 18 years of age, N=~30, and 12 to < 15 years of age, N=~30 [randomized in a 2:1 ratio to receive 5 µg SARS-CoV-2 rS adjuvanted with 50 µg Matrix-M1 or placebo]) will be enrolled first and will be followed for 7 days after the first vaccine dose for assessment of reactogenicity and overall safety data, which will be reviewed prior to enrollment of the remaining ~2,940 adolescents. Similarly, 7 days of reactogenicity and overall safety data following the receipt of the second vaccine dose in the sentinel group will be reviewed before the second vaccine dose is administered to the rest of the adolescent participants. In-person review of safety and/or blood collection for immunogenicity will be supplemented by remote contact (phone, text, email) on Days 7 (+ 3 days), 14 (+ 3 days), 28 (+3 days), 42 (+3 days) and 49 (+ 7 days) in the initial set of vaccinations. The blinded crossover visits for pediatric participants will begin ~6 months after the last vaccination of the initial set of vaccinations (or earlier depending upon feasibility, EUA vaccine availability

for this age group, and the dynamics of the pandemic). All participants who remain in blinded follow-up at the time of the blinded crossover will be eligible for crossover vaccination, regardless of whether they have experienced a COVID-19 diagnosis. Subsequent visits at Months 12 (± 15 days), 18 (± 30 days) and 24 (± 30 days) after the initial set of vaccinations will be performed to assess the durability of immune response, taking into account when participants received active vaccine (initial or crossover).

In addition to the aforementioned Unscheduled Acute Illness and Unscheduled Convalescent Visits in the **Pediatric Expansion**, an Unscheduled General Visit may be conducted by study personnel in the event of a general medical issue other than COVID-19 symptomatology, eg, reconsent to an amended Consent Form, if needed. An EoS visit will be recorded for all study participants at approximately 24 months (± 30 days) after their initial set of vaccinations or at their last visit on study. Should participants decide to terminate early, an EoS telephone or in-person visit will occur to collect the maximum safety data and blood sample, if possible. All study participants who choose to be unblinded or to withdraw from active surveillance will be encouraged to continue in follow-up for safety and reported COVID-19 cases via remote contacts with investigative site personnel.

8.1.8 Safety Follow-up after Unblinding

Participants that requested to be unblinded and/or are continuing in the study for Safety Follow-up will have visits replaced by remote contact (phone, email, text), at a minimum, every 90 days (± 30 days) from last dose received during the first year of participation. During the second year (ie, Months 12 to 24), contact will be continued every 180 days (± 30 days). **No eDiary, nasal swabs or blood samples will be collected during safety follow-up.** MAAEs, SAEs, AESI, and COVID-19 diagnoses will be collected during the remote contacts at the same time points described in Table 1 for scheduled visits in the participants that did not request unblinding. Receipt of another COVID-19 vaccine used under EUA should be recorded as a concomitant medication. The End of Study page is not to be completed for these participants until the end of their participation in the study.

The duration of the study, excluding screening, is approximately 24 months for each participant. Participants who choose to be unblinded to receive EUA-authorized vaccine will be followed for safety (SAE, MAAE, AESI and COVID-19 diagnosis) by remote contact on the remaining schedule as specified in Table 1 and Table 2. No further eDiary entries, nasal swabs or blood draws for immunogenicity will be required.

8.1.9 Safety Monitoring

This protocol has extensive safety monitoring in place. Safety is monitored routinely by the ICON Medical Monitor, Novavax Pharmacovigilance and Safety Surveillance Physicians, Novavax Clinical Development Leads and routinely by the 2019nCoV-301 Protocol Safety Review Team (PSRT) (for **Adult Main Study** only). In the **Adult Main Study** and **Pediatric Expansion**, a

centralized DSMB will be established in collaboration with NIH, NIAID, Biomedical Advanced Research and Development Authority (BARDA) and Novavax according to the charter dictated by the participating groups. This group will review interim unblinded data periodically in the **Adult Main Study** and **Pediatric Expansion** and make recommendations with respect to safety and emerging efficacy.

The short-term safety of the adolescent cohort (ie, analysis of 7-day reactogenicity and safety in the first ~60 participants enrolled in an approximately even distribution across age groups; 15 to < 18 years and 12 to < 15 years before enrollment of the remainder of the adolescent cohort) will be reviewed by the DSMB. Likewise, analysis of 7-day reactogenicity and safety after receipt of the second vaccine dose in the first ~60 participants enrolled will be reviewed by the DSMB before the second vaccine dose is administered to the remainder of the adolescent participants. The safety data will be simultaneously shared and reviewed internally by the Sponsor.

, Finally, the DSMB may recommend that the trial be terminated or that specific groups be withdrawn from the study, if any subgroup manifests serious or widespread side effects. The DSMB will be informed immediately by the ICON unblinded statistician if the pre-specified stopping boundary is met in the **Adult Main Study**, indicating that the vaccine causes harm by increasing the rate of mild, moderate or severe COVID-19. In addition, the DSMB will monitor the study for high vaccine efficacy or for futility to detect vaccine activity.

8.1.10 Study Vaccination Pause Rules (Pediatric Expansion)

AEs meeting any one of the following criteria will result in a pause being placed on subsequent vaccinations pending further review by the Novavax internal Safety Review Team (SRT). AEs meeting the criteria below will be forwarded to the DSMB Chair for review:

- Any SAE attributed to vaccine based on Sponsor assessment within the first 7 days following the first or second initial vaccination in the first 60 adolescent participants.
- Any toxicity grade 3 or higher (severe or life-threatening) single solicited (local or systemic) AE term occurring in ≥10% of the first 60 participants (≥6 participants) within the first 7 days following the first or second initial vaccination.
- Any grade 3 (severe) unsolicited single AE preferred term for which the investigator assesses as related occurring in ≥5% of the first 60 participants (≥3 participants) within the first 7 days following the first or second initial vaccination.

In addition, any SAE assessed as related to vaccine (final assessment by the Sponsor) will be reported by the Sponsor to the DSMB Chair as soon as possible, and within 24 hours of the sponsor’s assessment of the relatedness of the event. Based on the initial report of the event to the DSMB Chair, the Chair may convene an ad hoc meeting of the Board or make alternative recommendations to the Sponsor with respect to the conduct of the study. The DSMB Charter

defines processes for how this review will occur and how the Chair's recommendations will be documented.

The Sponsor, along with medical monitor, may request a DSMB review for any safety concerns that may arise in the Pediatric Expansion of the study, even if they are not associated with any specific pause rule.

8.1.11 Participant Retention

Because the blinded crossover will provide all study participants with active vaccine, either initially or at the time of blinded crossover, it is anticipated that study participants and parents/caregivers will be incentivized to continue participation in the blinded study. Participants unblinded between the first (Day 0) and second (Day 21) dose of trial vaccine will not be eligible to receive further investigational product on this protocol. Similarly, participants who choose to withdraw from the blinded trial after having received both doses of blinded study vaccine will not be eligible for blinded crossover. Safety follow-up of these participants will include remote contacts (eg, phone, email or text) to collect SAE, MAAE, AESI and COVID-19 diagnoses at the time points indicated on the Schedule of Events [see Table 1 for the **Adult Main Study** and Table 2 for the **Pediatric Expansion**] or at regular intervals if visits are not mandated. Receipt of another COVID-19 vaccine under EUA and date of administration should be recorded as a concomitant medication. Participants whom, upon unblinding for the purpose of receiving EUA-authorized vaccine, learn they had received active vaccine will be censored at the time of unblinding. Adult participants who are unblinded after 26 January 2021 are not eligible to receive further investigational product on this protocol. Similarly, participants who choose to withdraw from the blinded trial after having received both doses of blinded study vaccine will not be eligible for blinded crossover. Safety follow-up of these participants will include remote contacts (eg, phone, email or text) to collect SAE, MAAE, AESI and COVID-19 diagnoses at the time points indicated for scheduled visits in the participants that did not request unblinding (see Table 1) or at regular intervals if visits are not mandated. No eDiary, nasal swabs or blood samples for immunogenicity will be obtained.

8.2 Discussion of Study Design

This is a Phase 3, multinational, multicenter, randomized, observer-blinded, placebo-controlled study to evaluate the efficacy, safety and immunogenicity of 5 µg SARS-CoV-2 rS with 50 µg Matrix-M1 adjuvant in up to approximately 30,000 adult participants ≥ 18 years of age at substantial risk of exposure to and infection with SARS-CoV-2 infection (**Adult Main Study**). In the **Pediatric Expansion**, approximately 3,000 adolescent participants 12 to < 18 years of age will

be enrolled. A placebo control is considered ethical, as there are no vaccines or other preventive agents approved for SARS-CoV-2. In this study, participants will be stratified by age group (**Adult Main Study**: 18 to ≤ 64 years and ≥ 65 years, **Pediatric Expansion**: 12 to < 18 years) and randomized in a 2:1 ratio via block randomization to initially receive 2 IM injections of 5 µg SARS-CoV-2 rS adjuvanted with 50 µg Matrix-M1 or normal saline placebo on Days 0 and 21 + 7 days. An unequal randomization schema (2:1) was selected to expose more participants to active vaccine, and statistical modeling showed satisfactory statistical power for this randomization ratio. Such an approach should not negatively impact the power of the study, given the large sample size and prevalence of active disease.

Following collection of sufficient safety data to support application for EUA, participants will be scheduled for administration of 2 injections of the alternate study material 21 days apart ("blinded crossover"). That is, initial recipients of placebo will receive SARS-CoV-2 rS with Matrix-M1 adjuvant and initial recipients of SARS-CoV-2 rS with Matrix-M1 adjuvant will receive placebo. This blinded crossover will allow all participants to receive active vaccine in the study.

This study will be multinational and multicenter, with countries selected based on the expected COVID-19 epidemiology and healthcare system characteristics. Study sites will be selected globally, including in the US, to ensure a diverse study population is represented with respect to Black/African American and Native American race and Latinx ethnicity. At least 25% of the adult study population is intended to be in the ≥ 65 years age group. Most study participants are expected to be enrolled in the US. Consideration will be paid to the enrollment of participants for whom vaccines authorized for Emergency Use are not, or not anticipated to be, recommended or available during the early months of this trial.

For the Adult Main Study, prioritization will be given to enrollment of individuals at high risk for COVID-19 by virtue of Black/African American or Native American race, Latinx ethnicity, co-morbid conditions (BMI > 30 kg/m^2, chronic kidney or lung disease, cardiovascular disease, diabetes mellitus type 2) and by life circumstance (living or working conditions involving known frequent exposure to SARS-CoV-2 or to densely populated circumstances [eg, factory or meat packing plants, essential retail workers, etc). (See Section 8.6 Recruitment and Retention for guidelines with respect to high-risk characteristics.)

In the **Pediatric Expansion**, participants will only be enrolled in the US. All efforts will be made to enroll similar numbers of participants in the subgroups 12 to < 15 years of age, and 15 to < 18 years of age. As with the Adult Main Study, an effort will be made to recruit adolescents of a diverse population including underserved minorities. Enrollment of the full adolescent cohort of participants (12 to < 18 years) will be contingent upon the review of early safety data (ie, 7 days of reactogenicity and overall safety post-dose 1) to be reviewed in the first ~60 enrolled adolescents (randomized in a 2:1 ratio to receive 5 µg SARS-CoV-2 rS adjuvanted with 50 µg Matrix-M1 or placebo) before enrollment of the remainder of the adolescent participants

(N=~2,940). Likewise, administration receipt of the second vaccine dose to the full participant population will be contingent upon the review of early safety data (ie, 7 days of reactogenicity and overall safety post-dose 2) in the first ~60 enrolled adolescents before dosing the remainder of the adolescent participants. Safety data will be provided to and reviewed by the DSMB after each early safety data review period (ie, 7 days after each vaccination in the sentinel group). Simultaneously, safety data will be reviewed internally by the Sponsor.

Much of the clinical data will be collected using EDC systems installed on participants' or parent(s)/caregiver(s) smartphones. Participants or parent(s)/caregiver(s) who do not own a device that can accommodate this form of patient-reported outcomes will be provided with a compatible device to use for the study. Care will be taken to thoroughly train all participants in the use of the application on their electronic devices, and a 24/7 Help Desk will be available for technical issues, as needed. For data entry issues, participants or parent(s)/caregiver(s) should contact the study site. Lastly, this study will follow participants for 24 months after the initial set of vaccinations to explore long-term efficacy, safety, and immunogenicity of Matrix-M1 adjuvanted SARS-CoV-2 rS.

8.2.1 End of Study Definition

A participant is considered to have completed the study if they have completed all phases of the study, including the EoS visit.

The end of the study is defined as the date of the last EoS visit for the last participant in the study globally.

8.2.2 Trial Vaccine After the End of Study

In the **Adult Main Study**, following collection of sufficient safety data to support application for EUA, participants will be scheduled for administration of 2 injections of the alternate study material 21 days apart ("blinded crossover"). That is, initial recipients of placebo will receive SARS-CoV-2 rS with Matrix-M1 adjuvant and initial recipients of SARS-CoV-2 rS with Matrix-M1 adjuvant will receive placebo. Because the blinded crossover will provide all study participants with SARS-CoV-2 rS with Matrix-M1 adjuvant, either initially or at the time of blinded crossover, it is anticipated that participants will be incentivized to remain in the blinded study. Participants who choose, however, to be unblinded prior to the blinded crossover and receive EUA-authorized vaccine outside this protocol will be censored in the efficacy analysis at the time of unblinding but will be encouraged to remain in safety follow-up as defined in this protocol (See Section 8.1.3).

8.3 Study Population

Prospective approval of protocol deviations to recruitment and enrollment criteria, also known as protocol waivers or exemptions, is not permitted.

8.3.1 Inclusion Criteria

Each participant in the **Adult Main Study** must meet all of the following criteria to be enrolled in this study:

1. Adults ≥ 18 years of age at screening who, by virtue of age, race, ethnicity or life circumstances, are considered at substantial risk of exposure to and infection with SARS-CoV-2. (See Section 8.6, Recruitment and Retention for guidelines with respect to high-risk characteristics.)
2. Willing and able to give informed consent prior to study enrollment and to comply with study procedures.
3. Participants of childbearing potential (defined as any participant who has experienced menarche and who is NOT surgically sterile [ie, hysterectomy, bilateral tubal ligation, or bilateral oophorectomy] or postmenopausal [defined as amenorrhea at least 12 consecutive months]) must agree to be heterosexually inactive from at least 28 days prior to enrollment and through 3 months after the last vaccination OR agree to consistently use a medically acceptable method of contraception from at least 28 days prior to enrollment and through 3 months after the last vaccination.
4. Is medically stable, as determined by the investigator (based on review of health status, vital signs [to include body temperature], medical history, and targeted physical examination [to include body weight]). Vital signs must be within medically acceptable ranges prior to the first vaccination.
5. Agree to not participate in any other SARS-CoV-2 prevention trial during the study follow-up.

Each participant in the **Pediatric Expansion** must meet all of the following criteria to be enrolled in this study:

1. Pediatric participants 12 to < 18 years of age at screening, determined to be healthy or medically stable by the investigator (based on review of health status, vital signs [to include body temperature], medical history, and targeted physical examination [to include body weight]). Vital signs must be within medically acceptable ranges prior to the first vaccination.
2. Participant and parent(s)/caregiver(s) or legally acceptable representative willing and able to give informed consent and assent, as required, prior to study enrollment and to comply with study procedures.

3. Participants of childbearing potential (defined as any participant who has experienced menarche and who is NOT surgically sterile [ie, hysterectomy, bilateral tubal ligation, or bilateral oophorectomy] must agree to be heterosexually inactive from at least 28 days prior to enrollment and through 3 months after the last vaccination OR agree to consistently use a medically acceptable method of contraception from at least 28 days prior to enrollment and through 3 months after the last vaccination.
4. Agree to not participate in another SARS-CoV-2 prevention trial during the study follow-up.

8.3.2 Exclusion Criteria

Adult and pediatric participants meeting any of the following criteria will be excluded from the study:

1. Unstable acute or chronic illness. Criteria for unstable medical conditions include:
   a. Substantive changes in chronic prescribed medication (change in class or significant change in dose) in the past 2 months.
   b. Currently undergoing workup of undiagnosed illness that could lead to diagnosis of a new condition.

   Note: Well-controlled human immunodeficiency virus [HIV] with undetectable HIV RNA [< 50 copies/mL] and CD4 count > 200 cells/µL for at least 1 year, documented within the last 6 months, is NOT considered an unstable chronic illness. Participant's or parent's/caregiver's verbal report will suffice as documentation.
2. Participation in research involving an investigational product (drug/biologic/device) administered within 45 days prior to first study vaccination.
3. History of a previous laboratory-confirmed diagnosis of SARS-CoV-2 infection or COVID-19.
4. Received any vaccine within 4 days prior to first study vaccination or planned receipt of any vaccine before Day 49 (ie, 28 days after second vaccination), except for influenza vaccination, which may be received ≥ 4 days prior to or ≥ 7 days after either study vaccination.
5. Autoimmune or immunodeficiency disease/condition (iatrogenic or congenital) or therapy that causes clinically significant immunosuppression.

   NOTE: Stable endocrine disorders (eg, thyroiditis, pancreatitis), including stable diabetes mellitus with no history of diabetic ketoacidosis) are NOT excluded.
6. Chronic administration (defined as > 14 continuous days) of immunosuppressant or systemic glucocorticoids, within 90 days prior to first study vaccination.

NOTE: An immunosuppressant dose of glucocorticoid is defined as a systemic dose ≥ 20 mg of prednisone per day or equivalent. The use of topical, inhaled, and nasal glucocorticoids is permitted. Topical tacrolimus and ocular cyclosporin are permitted.

7. Received immunoglobulin or blood-derived products, within 90 days prior to first study vaccination.
8. Active cancer (malignancy) on chemotherapy that is judged to cause significant immunocompromise within 1 year prior to first study vaccination (with the exception of malignancy cured via excision, at the discretion of the investigator).
9. Any known allergies to products contained in the investigational product.
10. Participants who are breastfeeding, pregnant or who plan to become pregnant within 3 months following last study vaccination.
11. Any other condition that, in the opinion of the investigator, would pose a health risk to the participant if enrolled or could interfere with evaluation of the trial vaccine or interpretation of study results.
12. Study team member or first-degree relative of any study team member (inclusive of Sponsor, and study site personnel involved in the study).
13. Current participation in any other COVID-19 prevention clinical trial.

8.3.3 Other Considerations:

Participants meeting the following criterion may be delayed for subsequent vaccination:

- Respiratory symptoms in the past 3 days (ie, body temperature of > 38.0ºC, cough, sore throat, difficulty breathing). Participant may be vaccinated when all symptoms have been resolved for > 3 days. Out of window vaccination is allowed for this reason.

8.4 Prohibited Medications

Prescription medications for the prevention of COVID-19 are prohibited during this study. EUA-authorized vaccines received by participants outside this protocol must be recorded as concomitant medications. Any vaccine received within 4 days prior to the first study vaccination or planned receipt of any vaccine before Day 49 (ie, 28 days after second vaccination). Seasonal influenza vaccination, may be received ≥ 4 days prior to or ≥ 7 days after either study vaccination to avoid confounding reactogenicity observations.

8.5 Lifestyle Considerations

There are no lifestyle restrictions.

8.6 Strategies for Recruitment and Retention

All recruitment material will be approved by an Independent Ethics Committee (IEC) or Institutional Review Board (IRB) prior to implementation.

In addition to at least 25% adult participants ≥ 65 years of age, every effort must be made to recruit and enroll racially and ethnically diverse populations that appropriately reflect the populations most impacted by the COVID-19 pandemic. Additionally, potential participants of any eligible age who reflect the baseline co-morbidities that are most likely to suffer severe COVID-19 (eg, obesity [BMI > 30 kg/m^2], chronic kidney or lung disease, cardiovascular disease and diabetes mellitus type 2) should be prioritized for enrollment in the study with a goal of ≥ 25% of participants with high-risk co-morbidities. Participants will also be considered at high risk if their living or working conditions involve known frequent exposure to SARS-CoV-2 or to densely populated circumstances (eg, factory or meat packing plants, essential retail workers, etc). Consideration will be paid to the enrollment of participants for whom vaccines authorized for Emergency Use are not, or not anticipated to be, recommended or available during the early months of this trial.

Specifically, in the **Adult Main Study**, study sites should also target **overall enrollment of at least 15% Black/African Americans, 10% to 20% Latinx Americans and, where feasible, 1% to 2% Native Americans for the Adult Main Study.** Demographic characteristics and baseline co-morbidities will be monitored during the enrollment period to ensure the desired population is being recruited. As with the **Adult Main Study**, in the **Pediatric Expansion**, an effort will be made to recruit adolescents of a diverse population including underserved minorities.

Retention of participants and capture of endpoint COVID-19 will be facilitated by regular monitoring of the daily eDiary so that participants who are noted to not have reported temperature and symptoms for ≥ 7 days will be contacted by phone to assess clinical status and maintain engagement in the study. Constant centralized monitoring of the electronic database will enable identification of any potential issues related to participant retention, including study participants who have missed regular reporting. Retention of study participants will also be facilitated by the inclusion of the “blinded crossover” that ensures all participants receive active vaccine, either initially or after accrual of sufficient efficacy and safety data to support application for EUA.

8.7 Screen Failures

Screen failures are defined as participants who consent to participate in the clinical study but are not subsequently randomly assigned to trial vaccine/entered in the study. A minimal set of screen failure information is required to ensure transparent reporting of screen failure participants to meet the Consolidated Standards of Reporting Trials (CONSORT) publishing requirements and to respond to queries from regulatory authorities. Minimal information includes demography, screen failure details, eligibility criteria, and any SAEs.

Participants who withdraw, are withdrawn or terminated from this study, or are lost to follow-up after signing the ICF but prior to first study vaccination may be replaced. Participants who receive trial vaccine and subsequently withdraw, are discontinued from further vaccination, are terminated from the study, or are lost to follow-up will not be replaced.

8.8 Trial Vaccine Discontinuation and Participant Discontinuation/Withdrawal

8.8.1 Trial Vaccine Discontinuation

The investigator may withhold the second vaccination from a participant in the study if the participant:

1. Is noncompliant with the protocol.
2. Experiences an SAE or intolerable AE(s) for which vaccination is not advised by the investigator.
3. Pregnancy (discontinuation of further vaccination is required).

The investigator must determine the primary reason for the participant's premature discontinuation of trial vaccine and record this information on the treatment disposition electronic case report form (eCRF) page. The investigator and study staff must discuss with the participant, the need for the participant's continued participation for safety follow-up according to the study visit schedule. Participants who discontinue due to an AE should be followed to resolution of the AE or determination that it is a chronic condition.

The appropriate personnel from the study site in consultation with ICON Clinical Research Ltd. will assess whether trial vaccine should be discontinued for any participant whose treatment code has been broken inadvertently for any reason.

The investigator must also contact the interactive web response system (IWRS) to register the participant's discontinuation from trial vaccine.

8.8.2 Study Temporary Discontinuation/Vaccine Pause

Safety issues, such as the following, will be reviewed by the independent DSMB during their periodic data reviews, and a determination made regarding the continuation of vaccination in the study:

- An unacceptable imbalance of more SAEs and/or suspected unexpected serious adverse reactions (SUSARs) in the active vaccine treatment group.
- Moderate-to-severe COVID-19 occurring in statistically more vaccine recipients than placebo recipients, as might be suggestive of vaccine-enhanced disease, see Section 12.5.

Continued follow-up of all enrolled participants will be maintained to fully characterize the safety profile.

8.8.3 Withdrawal of Participants

8.8.3.1 Discontinuation/Withdrawal by Participant

Participants may voluntarily withdraw consent to participate in the study for any reason at any time or may be withdrawn at the discretion of the investigator for safety, behavioral, compliance, or administrative reasons.

If a participant withdraws consent, the investigator must make every effort to determine the primary reason for this decision and record this information on the treatment disposition eCRF page. If the participant decides to completely withdraw from the study (refuses any further study participation or contact), all study participation for that participant will cease and data to be collected at subsequent visits will be considered missing. Further attempts to contact the participant are not allowed unless safety findings require communication or follow-up.

Participants may refuse further procedures (including vaccination) but are encouraged to remain in the study for safety follow-up. In such cases where only safety is being conducted, participant contact could be managed via telemedicine contact (eg, telephone, web chat, video, FaceTime, etc), as described in Section 8.2.2.

8.8.3.2 Discontinuation/Withdrawal by Investigator

The investigator can also withdraw a participant upon the request of the Sponsor or if the Sponsor terminates the study. Upon the occurrence of an SAE or intolerable AE, the investigator may confer with the Sponsor before the second vaccination. Participants who become pregnant on-study will be followed for the duration of the pregnancy to document the pregnancy outcome.

8.8.3.3 Discontinuation/Withdrawal by Sponsor (Study Halting Rules)

Although Novavax has every intention of completing the study, they reserve the right to discontinue the study at any time for clinical or administrative reasons. The EoS is defined as the date on which the last participant completes the last study visit (including the EoS visit). Any additional long-term follow-up that is required for monitoring of the resolution of an AE or finding may be appended to the clinical study report.

In addition, the study will be discontinued if Novavax, Inc. or designee, including through DSMB recommendation, judges it necessary for medical, safety, or business reasons consistent with applicable laws, regulations and GCP.

8.8.4 Study Vaccination Pause Rules (Pediatric Expansion)

AEs meeting any one of the following criteria will result in a pause being placed on subsequent vaccinations pending further review by the Novavax internal Safety Review Team (SRT). AEs meeting the criteria below will be forwarded to the DSMB Chair for review:

- Any SAE attributed to vaccine based on Sponsor assessment within the first 7 days following the first or second initial vaccination in the first 60 adolescent participants.
- Any toxicity grade 3 or higher (severe or life-threatening) single solicited (local or systemic) AE term occurring in ≥10% of the first 60 participants (≥6 participants) within the first 7 days following the first or second initial vaccination.
- Any grade 3 (severe) unsolicited single AE preferred term for which the investigator assesses as related occurring in ≥5% of the first 60 participants (≥3 participants) within the first 7 days following the first or second initial vaccination.

In addition, any SAE assessed as related to vaccine (final assessment by the Sponsor) will be reported by the Sponsor to the DSMB Chair as soon as possible, and within 24 hours of the sponsor's assessment of the relatedness of the event. Based on the initial report of the event to the DSMB Chair, the Chair may convene an ad hoc meeting of the Board or make alternative recommendations to the Sponsor with respect to the conduct of the study. The DSMB Charter defines processes for how this review will occur and how the Chair's recommendations will be documented.

The sponsor, along with medical monitor, may request a DSMB review for any safety concerns that may arise in the Pediatric Expansion of the study, even if they are not associated with any specific pause rule.

8.9 Lost to Follow-up

Participants will be asked to provide an emergency contact at the time of the Informed Consent process. All reasonable efforts, including contact of emergency contact, must be made to locate participants to determine and report their ongoing status. Lost to follow-up is defined by the inability to reach the participant after a minimum of 3 documented phone calls, text messages, faxes or emails (not performed on the same day), as well as a lack of response by the participant to one registered mail letter. All attempts should be documented in the participant's source documents and/or medical records. If it is determined that the participant has died, the study site will use permissible local methods to obtain the date and cause of death and as much other information as can be obtained, including post-mortem reports.

Data that would have been collected at subsequent visits will be considered missing.

8.10 Discontinuation of Study Sites

Study site participation may be discontinued if Novavax, Inc. or designee, the investigator or IRB/IEC of the study site judges it necessary for medical or safety reasons consistent with applicable laws, regulations and Good Clinical Practice (GCP) guidelines.

9 STUDY TREATMENT

The following supplies will be used for vaccination in the study:

- SARS-CoV-2 rS (5 µg) adjuvanted with Matrix-M1 (50 µg)
- Placebo (normal saline)

Formulation: SARS-CoV-2 rS is a liquid solution formulated in 25 mM phosphate buffer (pH 7.2), 300 mM sodium chloride, and 0.01% (w/v) polysorbate 80, and diluted with the same to specified concentrations co-formulated with Matrix-M1 adjuvant that also contains phosphate buffered saline.

Further details on the trial vaccine can be found in the SARS-CoV-2 rS IB and description of the presentation are defined in the Pharmacy Manual.

9.1 Administration of Study Treatment(s)

The vaccine should be drawn into a syringe on the day of administration by a qualified and unblinded member of study site personnel, and the vaccine should be administered according to standard practice by qualified study site personnel in a way to maintain the blind, as directed in the Pharmacy Manual.

The study vaccination regimen will comprise 2 IM injections (Day 0 and Day 21 + 7 days) of injection volume of 0.5 mL in the deltoid. The dose level is 5 µg SARS-CoV-2 rS adjuvanted with 50 µg Matrix-M1 based on data from earlier nonclinical and early clinical trials. Participants who are discovered to have received the "wrong" study vaccine at the first injection should be administered the same material at the second injection and will be analyzed in the treatment received rather than that to which they were randomized. Participants should then receive the originally randomized material at the time of blinded crossover. A participant who receives the "wrong" study material at the second injection **inadvertently** must be unblinded to offer a third dose ensuring that the participant receives at least 2 doses of the NVX-CoV2373 vaccine or to receive an EUA vaccine. In either situation, a protocol deviation must be reported.

At the time of the blinded crossover, study participants will receive the alternate study material in 2 IM injections 21 + 7 days apart; that is, initial recipients of placebo will receive SARS-CoV-2 rS with Matrix-M1 adjuvant and initial recipients of SARS-CoV-2 rS with Matrix-M1 adjuvant will receive placebo. The blind to treatment assignment will not be broken.

All initial and crossover sets of vaccinations will be administered on an outpatient basis by qualified vaccine administrators in a way to maintain the blind. Unblinded product will require unblinded study site personnel who may administer the study vaccine if qualified to do so, but will not otherwise be involved in the study procedures or observations of participants.

9.2 Study Treatment Packaging and Labelling

9.2.1 Packaging and Labelling

Novavax, Inc. will provide adequate quantities and appropriate labelling of SARS-CoV-2 rS with Matrix-M1 adjuvant and PCI Pharma Services will ensure distribution to the study sites from a designated depot. Sodium chloride, 0.9% for injection (US Pharmacopeia, sterile or equivalent) is commercially available and will be supplied by PCI Pharma Services. The clinical unit pharmacy or equivalent will prepare the clinical trial materials (vaccine or placebo) for each participant. Detailed instructions for the handling of trial vaccine vials will be provided in a separate Pharmacy Manual.

9.2.2 Storage

All trial vaccines must be stored according to the labelled instructions in a secure cabinet or room with access restricted to necessary clinic personnel. The study site will be required to keep a temperature log to establish a record of compliance with storage conditions.

The SARS-CoV-2 rS vaccine with Matrix-M1 adjuvant should be stored at 2°C to 8°C in a secured location. DO NOT FREEZE.

Placebo (0.9% sodium chloride for injection) should be stored according to label directions.

9.3 Vaccine Compliance

All doses of the trial vaccine should be administered in the clinical unit under direct observation of clinic personnel and recorded in the eCRF but may need to occur outside of the study site depending on the pandemic situation (eg, home vaccinations). Home vaccination visits must have adequate oversight for issues associated with any immediate reaction. Clinic personnel will confirm that the participant has received the entire dose. The location (right or left arm), date, and timing of all doses of trial vaccine will be recorded in the participants' eCRF. If a participant is not administered trial vaccine, the reason for the missed dose will be recorded.

9.4 Prior Vaccinations and Concomitant Therapy

Administration of non-study medications, therapies, or vaccines (including EUA-authorized vaccines) will be recorded in the eCRF. Concomitant medications will include all medications (including vaccines) taken by the participant from the time of signing the ICF through 49 days, ie, 28 days after second injection of the initial set of vaccinations, and all medications taken by the participant for treatment of a reportable SAEs, MAAEs attributed to vaccine or AESIs from Day 50 through EoS (or through the early termination visit, if prior to EoS). Prescription and over-the-counter drugs, as well as herbals, vitamins, and supplements, will be included when used for the above indications.

Prohibited medications are detailed in Section 8.4.

9.5 Blinding and Randomization of Study Treatment(s)

This is an observer-blinded study. To maintain the blind, placebo vaccination via IM route will be included and unblinded study site personnel will manage vaccine logistics, preparation, and administration according to the Pharmacy Manual so as to maintain the blind from the remainder of the study site personnel and participants. The unblinded study site personnel may administer study vaccine if qualified to do so, but will not be involved in study-related assessments or have participant contact for data collection after administration of trial vaccine. At the time of implementation of the blinded crossover period, a similar procedure will be employed to ensure that all study participants and personnel remain blinded as to initial and subsequent treatment assignment.

Within each study site, participants will be assigned to the appropriate age stratum (18 to ≤ 64 years or ≥ 65 years in the **Adult Main Study**, and 12 to < 18 years in the **Pediatric Expansion**) and randomized to study treatment according to a list produced by ICON. Prior to production, the randomization specification will be reviewed and agreed by the study team (Sponsor and ICON). As block size is considered potentially unblinding information, it will be known to the Study Biostatistician only.

An IWRS will be responsible for the allocation of randomization numbers to individual participants. Randomization will take place at baseline after confirmation that the participant meets the inclusion/exclusion criteria. At the time of blinded crossover, the IWRS system will assign participants to the alternative treatment from that which they received initially. Participants will be randomized in a 2:1 ratio to receive either SARS-CoV-2 rS with Matrix-M1 adjuvant or placebo, administered via IM route. A copy of the randomization code with true treatment allocations will be held by ICON during the study. Another randomization list (containing treatment) will be provided to clinical supplies.

9.6 Procedure for Breaking the Randomization Code

A participant's vaccine assignment will not be revealed to the study site study team until the end of the study unless medical treatment of the participant depends on knowing the trial vaccine the participant received. Should a situation arise where unblinding is required, the investigator at that study site has the sole authority to obtain immediate unblinding via the IWRS without communication with the Sponsor, although communication with ICON or the Sponsor must occur as soon thereafter as possible.

Prior to unblinding, or as soon thereafter as possible, the investigator should contact the ICON Medical Monitor to discuss the medical emergency and the reason for revealing the actual vaccine received by that participant. Emergency code breaks are performed using the IWRS. Reasons for vaccine unblinding must be clearly explained and justified in the eCRF. The date on which the code was broken must also be documented. Participants in the **Adult Main Study** who, upon unblinding for the purpose of receiving EUA-authorized vaccine, learn they had received active

vaccine will be censored at the time of unblinding. Adult participants who are unblinded after 26 January 2021 are not eligible to receive further investigational product on this protocol.

Participants in the **Pediatric Expansion** who are unblinded between the first (Day 0) and second (Day 21) dose of trial vaccine will not be eligible to receive further investigational product on this protocol. Similarly, participants who choose to withdraw from the blinded trial after having received both doses of blinded study vaccine will not be eligible for blinded crossover.

When the investigator contacts the IWRS system to break a treatment code for a participant, they must provide the requested participant identifying information and confirm the necessity to break the treatment code for the participant. The investigator will then receive details of the study treatment for the specified participant and a fax or email confirming this information. The system will automatically inform the PSRT, ICON Site Monitor, the ICON Medical Monitor, and the ICON Project Manager that the code has been broken, but no treatment assignment will be communicated.

It is the investigator's responsibility to ensure that there is a procedure in place to allow access to the IWRS in case of emergency. The investigator will inform the participant how to contact their backup in cases of emergency when they are unavailable. The investigator will provide the protocol number, trial vaccine name if available, participant number, and instructions for contacting the local entity which has responsibility for emergency code breaks to the participant in case an emergency treatment code break is required at a time when the investigator and backup are unavailable.

In addition to the aforementioned emergency situations where the blind may be broken, the data will also be unblinded to the ICON unblinded statistician at specified time points for planned interim reviews by the DSMB.

9.7 Study Treatment Accountability

The investigator (or delegate) will maintain accurate records of receipt of all trial vaccine, including dates of receipt. Accurate records will be kept regarding when and how much trial vaccine is dispensed and used by each participant in the study. Reasons for departure from the expected dispensing regimen must also be recorded. At the completion of the study, and to satisfy regulatory requirements regarding trial vaccine accountability, all used and unused trial vaccines will be reconciled and retained or destroyed according to applicable regulations. No trial vaccine will be destroyed until authorized in writing by the Sponsor.

10 STUDY ASSESSMENTS AND PROCEDURES

Before performing any study procedures, all potential participants and parent(s)/caregiver(s) will sign an ICF and Assent form, as required, and provide an emergency contact as outlined in Section 13.2. Participants will undergo study procedures at the time points specified in the SoA (Table 1).

Due to the ongoing pandemic, recent national regulatory and local IEC/IRB and public health guidance may be applied at the study site locations regarding alterations in the ability of study participants to attend a study site for protocol-specified visits, with the study site's investigator being allowed to conduct safety assessments (eg, telephone, email or text message contact, alternative location for assessment, including local laboratories or home visits) when necessary and feasible, as long as protective procedures ensure such visits are conducted according to appropriate guidelines sufficient to assure the safety of study participants. Serum samples may be drawn using local phlebotomy services, home health, or other modalities if study site visits cannot occur. Vaccination visits must have adequate oversight for issues associated with any immediate reaction but may need to occur outside of the study site depending on the pandemic situation (eg, home vaccinations).

10.1 Efficacy Assessments

10.1.1 Active Surveillance for COVID-19

Participants in the **Adult Main Study** will be provided with a thermometer and instructed to monitor their body temperature daily throughout the study and to record body temperature and any other relevant symptoms daily in their eDiary (see Section 10.4.3 for details). Participants who are noted during regular monitoring of the daily eDiary entries to not have reported temperature and symptoms for ≥ 7 days will be contacted by phone to assess clinical status and maintain engagement in the study.

Symptoms of severe COVID-19 should be reported as an SAE (important medical event) beginning on Day 0 following the first study vaccine administration (Section 7.1.4) and appropriate medical care should be sought. Starting on Day 4, throughout the first 12 months of the study, when fever or other specified symptoms (see Table 5 for symptoms suggestive of COVID-19) are reported in the eDiary for at least 2 consecutive days for the same symptom, participants will be directed via the eDiary to begin daily nasal self-swabbing for PCR testing within 3 days of symptom onset at home for a total of 3 days and to initiate daily completion of the FLU-PRO symptom reporting instrument for 10 days after COVID-19 symptom onset or until the participant experiences 2 consecutive asymptomatic days. Participants will be instructed at their enrollment visit on the methods of nasal self-swabbing for COVID-19 and completion of the FLU-PRO symptom reporting instrument. In addition, the eDiary will alert the study site to contact the participant to schedule the in-person Unscheduled Acute Illness Visit. During the first 4 days after the second vaccination when solicited systemic reactogenicity symptoms may be similar to those of COVID-19, investigators should use their clinical judgement to decide if an Unscheduled Acute

Illness visit is warranted. Active surveillance for COVID-19 will continue after the blinded crossover through the first 12 months of study. Passive surveillance of safety and efficacy via remote contacts or the scheduled visits will continue during Months 12 to 24.

After the first day of home nasal swabbing, repeat nasal self-swabs should be obtained daily for a total of 3 days to ensure capture of intermittent shedding. The self-swabs obtained by the participant should be maintained according to directions provided in the 3-swab kit, and the designated courier should be contacted to pick up the kit for shipping to the central lab, as directed.

Participants may also be evaluated, and appropriate swab and blood samples obtained, by other methods to ensure the appropriate level of medical care (eg, telemedicine, hospital/COVID-19 ward records, home visits) based on ongoing pandemic and participant containment requirements.

For the **Pediatric Expansion**, starting on Day 4, throughout the first 12 months of the study, parent(s)/caregiver(s) of participants will be asked to report symptoms of COVID-19 to the site as soon as possible after symptoms onset, or during the weekly remote contact. Fever and other symptoms of COVID-19 (including date of onset, duration, etc.) will be collected in a paper memory aid and will be reported to the sites by the parent(s)/caregiver(s) either as a spontaneous phone call or during the weekly remote contact during the first 12 months. If the parent(s)/caregiver(s) report symptoms compatible with COVID-19 by spontaneous contact or during the weekly contact, the study site will schedule an in-person Unscheduled Acute Illness Visit. During the first 4 days after vaccination when solicited systemic reactogenicity symptoms may be similar to those of COVID-19, investigators should use their clinical judgement to decide if an Unscheduled Acute Illness visit is warranted. Active surveillance for COVID-19 will continue after the blinded crossover through the first 12 months of study. Passive surveillance of safety and efficacy via remote contacts or the scheduled visits will continue during Months 12 to 24. An eDiary will be used for the collection of 7-day reactogenicity data after each initial vaccination, however, it will not include a prompt to complete the FLU-PRO instrument or prompts for self-swabbing as these are not collected for the adolescent participants. An eDiary collection of reactogenicity will not be performed after crossover vaccinations. Prospective surveillance will continue via weekly phone calls after blinded crossover until Month 12, and will be conducted at 3 and 6 months (±30 days) post-crossover from Month 12 to Month 24.

10.1.2 Unscheduled Acute Illness and Convalescent Visits

At the in-person Unscheduled Acute Illness Visit, participants will be queried regarding AE symptoms, concomitant medications taken for these symptoms, undergo a targeted physical examination (to include O_2 saturation and respiratory rate), as indicated by participant's signs and symptoms, and have obtained by the study personnel a medically attended nasal swab, a blood sample for serologic testing and be trained on the use of the portable pulse oximeter that they will take home with them. No portable pulse oximeter will be distributed for home use for adolescent participants and there will be no self-swab collection in the adolescent participants. Medically

attended swabs collected at the Unscheduled Acute Illness Visit will be processed at the study site for shipment to the central laboratory according to established procedures as described in the Laboratory Manual.

All Unscheduled Acute Illness Visits and assessments performed during the visits will be recorded in the participant's eCRF. Laboratory samples collected for the purpose of routine medical care may not be reimbursed by the clinical trial.

Study participants whose home nasal self-swab and/or medically attended nasal swabs are confirmed at the central laboratory to be PCR-positive for SARS-CoV-2 at the Unscheduled Acute Illness Visit will be contacted by the study site to arrange an Unscheduled Convalescent Visit. The Unscheduled Convalescent Visit will occur approximately 1 month (or as soon thereafter, as feasible) after the onset of the PCR-confirmed case of COVID-19 at the Unscheduled Acute Illness Visit to assess status of AEs, record the clinical course of the disease on the Endpoint Form and obtain a blood sample for convalescent serologic testing. Pulse oximeters should be returned to the study site at this visit.

10.1.3 Nasal Swabs for Virus Detection

Nasal swabs of the anterior nares will be obtained at the study site on Day 0 (prior to study vaccination), at the Unscheduled Acute Illness Visit and at the first crossover vaccination visit. There will be no self-swab collection in the adolescent participants.

Participants who experience an SAE of severe COVID-19 any time after Day 0 should, if at all possible, have a nasal swab obtained (by site personnel or other healthcare personnel) to be sent by the study site to the study central laboratory. Such a swab, if obtained, will constitute the medically attended nasal swab recorded on the Acute Illness Visit form.

Starting on Day 4, throughout the first 12 months of the study, when fever or other specified symptoms (see Table 5 for symptoms suggestive of COVID-19) are reported in the eDiary for at least 2 consecutive days for the same symptom, participants in the **Adult Main Study** will be directed via the eDiary to begin daily nasal self-swabbing for PCR testing within 3 days of symptom onset at home for a total of 3 days to ensure capture of intermittent shedding. The self-swabs obtained by the participant should be maintained according to directions provided in the 3-swab kit, and the designated courier should be contacted to pick up the kit for shipping to the central lab, as directed. Medically attended swabs collected at the Unscheduled Acute Illness Visit and at the first crossover vaccination visit will be processed at the study site for shipment to the central laboratory according to established procedures.

Participants in the **Adult Main Study** will be instructed at their enrollment visit on the method of self-swabbing for COVID-19 and procedure for arranging transport of swabs to the central lab. Quantitative PCR may be performed on PCR-positive swabs to assess viral load and sequencing

of viral genetic material detected in nasal swab PCR testing to evaluate viral mutations. No participant genetic material will be sequenced or otherwise tested.

10.1.4 FLU-PRO (Adult Main Study only)

Participants will be instructed at their enrollment visit on the completion of the FLU-PRO symptom questionnaire. A Help Desk will be available 24/7 for technical issues. For data entry issues, participants should contact the study site.

Starting on Day 4, throughout the first 12 months of the study, when fever or other specified symptoms (see Table 5 for symptoms suggestive of COVID-19) are reported in the eDiary for at least 2 consecutive days for the same symptom, participants will be directed via the eDiary to initiate daily completion of the FLU-PRO symptom reporting instrument for 10 days after COVID-19 symptom onset or until the participant experiences 2 consecutive asymptomatic days.

The FLU-PRO will be completed by participants electronically via an application installed on the participants' smartphone or a similar device provided by the study for individuals who do not own a smartphone. The FLU-PRO questionnaire was designed to standardize and comprehensively assess symptoms associated with various viruses across multiple body systems over the course of influenza disease, and has been adapted for COVID-19, within and across subgroups. It was developed using qualitative and quantitative methods consistent with scientific measurement standards and US Food and Drug Administration (FDA) and European Medicines Agency (EMA) guidelines for clinical outcome assessments.

FLU-PRO has been tested and used in studies of influenza, influenza-like illness, rhinovirus, enterovirus, and more recently COVID-19. To date, the FLU-PRO has been completed by over 4,000 participants between 12 to 81 years of age with adherence rates over 90%.

FLU-PRO is a 32-item instrument that assesses severity of symptoms of influenza and influenza-like illness across 6 body systems (nose, throat, eyes, chest/respiratory, gastrointestinal, and body/systemic), with at least 2 additional symptoms (ie, acute loss of sense of smell and/or taste) added that have been associated with COVID-19. In the current study, participants will complete the FLU-PRO daily for 10 days after onset or until the participant experiences 2 asymptomatic days. Each question is brief (sign or symptom only with severity rating) and the entire questionnaire takes under 4 minutes to complete. The instrument also provides data on the presence/absence and severity of symptoms, symptom profiles, and change over time; these are the data points that will be used in this trial. It is noted that for purposes of this study the FLU-PRO diary is being used only for collection of symptoms and severity; there is no intention of using the FLU-PRO scores in any analyses.

10.1.5 Oxygen Saturation Monitoring

At the Unscheduled Acute Illness Visit, O_2 saturation (at rest and following mild exercise, defined as walking around the room for 1 minute) will be measured using a pulse oximeter. In the **Adult**

Main Study, the study sites will provide a portable pulse oximeter for recording O_2 saturation (at rest and following mild exercise) daily in the eDiary and train the participant on the use of the pulse oximeter under both conditions (rest and mild exercise). The device will be accompanied by careful instructions for use and recording of O_2 saturation in the eDiary and who to contact for assistance if the device malfunctions. The purpose of the monitoring of O_2 saturation is to enhance the assessment of severity and progression of COVID-19, as well as to ensure that participants are adequately informed with respect to their medical condition and are warned to seek medical care when needed. No portable pulse oximeter will be distributed for home use for adolescent participants.

10.2 Immunogenicity Assessments

In the **Adult Main Study,** blood samples for serologic assessments (anti-NP antibodies, IgG antibody to SARS-CoV-2 S protein, MN, and hACE2 inhibition) will be collected from all enrolled participants before vaccination and at the appointed time points following vaccination (see Table 1). Blood samples will be obtained during the immediate period after the first set of vaccinations only (Day 0, 21 and 35), and in the long-term (Months 12, 18 and 24) follow-up of all participants.

Immune measurements (IgG antibody to SARS-CoV-2 S protein, MN, and hACE2 inhibition) will be performed on a subset of collected sera from the Immunogenicity Population of up to approximately 1,200 adult participants from the active and placebo treatment groups split approximately evenly across the 2 age categories, designated at random by biostatisticians who are blinded to treatment assignment. Testing for anti-NP antibodies will be performed on serum from up to all enrolled participants to evaluate prior infection at baseline and new infection (including asymptomatic infection) across the duration of the study. Additional testing relevant to coronavirus infection and epidemiology may be identified during or after the trial that may be performed on banked sera. Whole blood samples for PBMC testing for cell-mediated immunity will be collected on Days 0, 21, and 35 from a small subset of participants (< 100) representing both age strata and reasonably reflecting the demographic subgroups enrolled at selected study site(s) with the capacity to isolate PBMCs. These study site(s) will be identified prior to trial initiation.

A subset of approximately 900 participants 18 to ≤ 64 years of age at selected study sites will be asked to return approximately 14 days after the second crossover vaccination dose to provide a blood sample for anti-SARS-CoV-2 rS IgG to compare immunogenicity of the vaccine lot utilized for the crossover vaccination period to that of the earlier lot used for the initial vaccination period.

Aliquots of all collected samples from this study may be retained for use in future relevant research for a maximum of 25 years (starting from the date at which the last participant had the last study visit), unless local rules, regulations, or guidelines require different timeframes or different procedures, in accord with participant consent.

In the **Pediatric Expansion,** blood samples for serologic assessments (anti-NP antibodies, IgG antibody to SARS-CoV-2 S protein, MN, and hACE2 inhibition) will be collected from all pediatric participants before the initial set of vaccinations and immediately prior to the crossover set of vaccinations. Immune responses immediately following vaccinations will be obtained from all participants after the initial set of vaccinations but not after the crossover vaccinations. Testing will be performed on all adolescent participants. In the **Pediatric Expansion**, whole blood samples for PBMC will be collected at Days 0, 7 and 28 from a small subset comprised of 50 adolescent participants enrolled at selected study site(s) with the capacity to isolate PBMCs. These cells will be evaluated for cell-mediated immune responses to the initial set of vaccinations (Table 2). In summary, there are 7 scheduled blood draws for immunogenicity assessments at the Day 0, 21, 35, C1 (Day 180), and Months 12, 18, and 24 visits, and during unscheduled visits (ie Acute illness, Convalescent, and General). For participants in the CMI cohort only (N=50), additional blood will be collected to obtain PBMCs at the Day 0, 7 and 28 visits.

10.3 Safety Assessments

The timing and frequency of all safety assessments are listed in the SoA (Table 1 and Table 2). Solicited and unsolicited AEs will be graded for severity using the provided criteria. Recording of solicited (following the initial set of vaccinations only) and unsolicited AEs will be conducted by EDC. AESIs, including potential immune-mediated medical conditions (PIMMC) and AESIs specific to complications of potential disease enhancement for COVID-19 will also be monitored (see Appendix 2 for details).

The timing and frequency of safety assessments are described in Table 1 and Table 2.

10.3.1 Definitions

The definition of AEs, treatment-emergent adverse events (TEAEs), AESIs and SAEs is given below. The investigator and any qualified designees are responsible for detecting, documenting, and recording events that meet the definition of and AE, SAE or AESI and remain responsible for following up AEs that are serious, considered related to the trial vaccine or study procedures, or that causes the participant to discontinue the trial vaccine/study.

10.3.1.1 Adverse Events

An AE is defined as any untoward medical occurrence in a participant, or clinical investigation participant administered a pharmaceutical product, and which does not necessarily have a causal relationship with this treatment. An AE can therefore be any unfavorable and unintended sign, symptom or disease temporally associated with the use of a medicinal (investigational) product, regardless of whether related to the medicinal (investigational) product.

10.3.1.2 Events Meeting the AE Definition

- Any abnormal laboratory test results or other safety assessments (eg, vital signs measurements), including those that worsen from baseline, considered clinically

significant in the medical and scientific judgement of the investigator (ie, not related to progression of underlying disease).

- Exacerbation of a chronic or intermittent pre-existing condition including either an increase in frequency and/or intensity of the condition.
- New conditions detected or diagnosed after trial vaccine administration even though it may have been present before the start of the study.
- Signs, symptoms, or the clinical sequelae of a suspected drug-drug interaction.
- "Lack of efficacy" or "failure of expected pharmacological action" *per se* will not be reported as an AE or SAE. Such instances will be captured in the efficacy assessments. However, the signs, symptoms, and/or clinical sequelae resulting from lack of efficacy will be reported as AE or SAE if they fulfill the definition of an AE or SAE.

10.3.1.3 Serious Adverse Event

An SAE is defined as any untoward medical occurrence that:

- Results in death. The cause of death is the AE, death is an outcome.
- Is life-threatening. The term 'life-threatening' in the definition of 'serious' refers to an event in which the participant was at risk of death at the time of the event. It does not refer to an event, which hypothetically might have caused death, if it were more severe.
- Requires inpatient hospitalization or prolongs existing hospitalization. In general, hospitalization signifies that the participant has been detained (usually involving at least an overnight stay) at the hospital or emergency ward for observation and/or treatment that would not have been appropriate in the physician's office or outpatient setting. Complications that occur during hospitalization are AEs. If a complication prolongs hospitalization or fulfils any other serious criteria, the event is serious. When in doubt as to whether 'hospitalization' occurred or was necessary, the AE should be considered serious. Hospitalization for elective treatment of a pre-existing condition that did not worsen from baseline is not considered an SAE.
- Results in persistent or significant disability/incapacity. The term disability means a substantial disruption of a person's ability to conduct normal life functions. This definition is not intended to include experiences of relatively minor medical significance such as uncomplicated headache, nausea, vomiting, diarrhea, influenza, and accidental trauma (eg, sprained ankle) which may interfere with or prevent everyday life functions but do not constitute a substantial disruption.
- Is a congenital anomaly/birth defect.

- Is an important medical event. Important medical events that may not result in death, be life-threatening or require hospitalization may be considered a serious adverse drug experience, when based on appropriate medical judgement, they may directly jeopardize the participant or the participant may require medical or surgical intervention to prevent one of the outcomes listed in this definition. Examples of such events include invasive or malignant cancers, intensive treatment in an emergency room or at home for allergic bronchospasm, blood dyscrasias or convulsions that do not result in hospitalization, or development of drug dependency or drug abuse.
- Events of severe COVID-19 (see Section 7.1.4) constitute important medical events for this study. Events that may have, in a different hypothetical situation, been life-threatening, but did not pose an immediate life-threatening condition to the given study participant are not considered SAEs.

10.3.1.4 Treatment-Emergent Adverse Event

TEAEs are defined as any AE occurring or worsening on or after the first dose of trial vaccine.

10.3.1.5 Adverse Event of Special Interest

Participants will be assessed for diagnosis of an AESI at all study contacts. AESIs include PIMMCs, AEs specific to complications of COVID-19 (listed in Appendix 2), or other potential AEs that may be determined at any time by regulatory authorities as additional information concerning COVID-19 is obtained. Given the concern for “cytokine storm”, an AESI of cytokine release syndrome will be included as an AE specific to COVID-19. Listings of AESI are presented in Appendix 2.

An AESI must be reported as if it is an SAE (Section 10.3.6).

10.3.1.6 Medically Attended Adverse Events

An MAAE is defined as an AE that leads to an unscheduled visit to a healthcare practitioner.

10.3.1.7 Reactogenicity Symptoms

On vaccination days, participants will remain in clinic (or under observation) for at least 30 minutes to be observed for any immediate reaction. Site specific local (arm) and general systemic reactogenicity reactions including start and stop dates will be recorded following the initial set of vaccinations and the investigator will apply a standard toxicology grading at the subsequent study visit (Appendix 4). Any immediate reaction will be recorded as AEs on day of vaccination. Reactogenicity will not be collected following the crossover set of vaccinations.

Participants will utilize their eDiary to record reactogenicity following vaccination. All participants will record reactogenicity starting on the same day of the vaccinations and for a total of 7 days. Study site personnel will regularly review the eDiary for completeness. Should any reactogenicity event extend beyond 7 days after vaccination (toxicity Grade $\geq$ 1), then it will be

recorded as an AE with a start date that matches Day 7 of the reactogenicity event and followed to resolution per FDA guidelines for AE capture. Following receipt of either dose of vaccine, reactogenicity events that meet the criteria for nasal swab for COVID-19 (eg, fever, generalized myalgia, etc.), and that occur after completion of Day 4 of reactogenicity reporting via the eDiary in the **Adult Main Study**, should prompt the collection of a nasal swab and initiation of follow-up according to the directions for COVID-19 surveillance (Section 10.1). At any time after Day 0, severe COVID-19 should be reported as an SAE and managed as described in Section 10.1.3.

10.3.1.8 Pregnancy

A urine pregnancy test will be performed at screening and prior to each vaccination only for participants of childbearing potential. A positive urine pregnancy test at any time during the study will result in the participant not receiving any further vaccination and will initiate the prescribed follow-up for pregnancy outcome.

Pregnancy itself is not regarded as an AE unless there is a suspicion that the trial vaccine may have interfered with the effectiveness of a contraceptive medication. However, the outcome of all pregnancies (spontaneous miscarriage, elective termination, preterm birth, normal birth or congenital abnormality) must be followed up and documented even after the participant has completed the study.

All reports of congenital abnormalities/birth defects/preterm (< 37 weeks gestation) are SAEs. Spontaneous miscarriages should also be reported and handled as SAEs. Elective abortions without complications should not be handled as SAEs, but should be reported as a follow-up report for outcome of the pregnancy. All outcomes of pregnancy must be reported to ICON on a Pregnancy Outcomes Report Form.

Pregnancies must be reported to ICON Pharmacovigilance and Safety Services (PVSS) within 24 hours of awareness, using the reporting details provided in Section 10.3.6.

10.3.2 Time Period and Frequency for Collecting AE and SAE Information

All AEs reported or observed during the study will be recorded on the AE page of the eCRF.

Medical occurrences that begin prior to the first dose of trial vaccine will be recorded on the Medical History/Current Medical Conditions section of the eCRF not the AE section.

All unsolicited AEs of any severity will be collected from the time of first study vaccination through 49 days (28 days after the second injection of each set of vaccinations [initial and crossover]) (Table 1). Any relevant observations made prior to the first dose of trial vaccine are to be recorded on the AE eCRF but will not be considered TEAEs and will be reported separately from TEAEs.

MAAEs will be collected from the time of first study vaccination through 49 days (28 days after second injection of each set of vaccinations [initial and crossover]), and MAAEs attributed to

vaccine will be collected from the time of first study vaccination until completion of the EoS (Table 1).

All SAEs and AESIs will be collected from signing of informed consent until completion of the EoS (Table 1).

At any time after completion of the EoS visit, if an investigator learns of an SAE that could reasonably be considered related to trial vaccine, they should promptly notify the Sponsor.

10.3.3 Method of Detecting AEs, MAAEs and SAEs

Care will be taken not to introduce bias when detecting AEs, MAAEs and SAEs. Open-ended and non-leading verbal questioning of the participant is the preferred method to enquire about AE occurrences. AESIs will be inquired about according to the specific diseases listed in Appendix 2.

10.3.4 Recording of AEs and SAEs

When an AE/SAE occurs, it is the responsibility of the investigator to review all available documentation (eg, hospital progress notes, laboratory reports, and diagnostics reports) related to the event. The investigator will then record all relevant AE/SAE information in the eCRF. For an SAE of severe COVID-19 (see Section 7.1.4), every effort should be made to obtain a nasal swab to be sent to the study central lab. This swab may be recorded in the Acute Illness Visit eCRF. Participants with PCR-confirmed COVID-19 should subsequently be followed as per Section 10.1.2.

It is not acceptable for the investigator to send photocopies of the participant's medical records in lieu of completion of the AE/SAE eCRF page. There may be instances when copies of medical records for certain cases are requested. In this case, all participant identifiers, with the exception of the participant number, will be redacted on the copies of the medical records before submission. The investigator will attempt to establish a diagnosis of the event based on signs, symptoms, and/or other clinical information. Whenever possible, the diagnosis (not the individual signs/symptoms) will be documented as the AE/SAE.

The following variables will be recorded for each AE: verbatim/AE description and date for AE start and stop, severity (refer to Section 10.3.4.1), seriousness (Section 10.3.1.3), causality (Section 10.3.4.2), whether the AE caused the participant to not receive the second dose of study vaccine (Section 10.3.4.3), any other action taken (Section 10.3.4.4), and the outcome (Section 10.3.4.5). A new AE must be recorded if the severity of the AE changes.

Should an SAE have an outcome of death, the report should contain a comment regarding the co-involvement of progression of disease, if appropriate, and should assign main and contributory causes of death.

10.3.4.1 Assessment of Intensity (Severity)

The severity (or intensity) of an AE/SAE refers to the extent to which it affects the participant's daily activities and will be classified as mild, moderate, or severe using the following criteria:

- Mild (grade 1): These events require minimal or no treatment and do not interfere with the participant's daily activities.
- Moderate (grade 2): These events result in a low level of inconvenience or require minor therapeutic measures. Moderate events may cause some interference with normal functioning.
- Severe (grade 3): These events interrupt a participant's usual daily activity and may require systemic drug therapy or other treatment. Severe events are usually incapacitating.

If the severity of an AE/SAE changes, the most intense severity should be reported. An AE/SAE characterized as intermittent does not require documentation of the onset and duration of each episode.

An event is defined as 'serious' when it meets at least one of the regulatory criteria listed in Section 10.3.1.3.

An AE of severe intensity need not necessarily be considered serious. For example, nausea that persists for several hours may be considered severe nausea, but not an SAE. On the other hand, a stroke that results in only a limited degree of disability may be considered a mild stroke but would be an SAE.

10.3.4.2 Assessment of Causality

The investigator must assess the relationship between trial vaccine and each occurrence of each AE/SAE using their clinical judgement. A reasonable possibility of a causal relationship requires that there are facts, evidence, and/or biological plausibility to suggest a relationship, rather than that a relationship cannot be ruled out. Alternative causes, such as underlying disease(s), concomitant therapy, and other risk factors, as well as a temporal relationship of the event to trial vaccine administration will be considered and investigated. The investigator will also consult the IB and/or Product Information (for marketed products) as part of their assessment.

For each AE/SAE, the investigator must document in the medical notes that they have reviewed the AE/SAE and have provided an assessment of causality. There may be situations in which an SAE has occurred and the investigator has minimal information to include in the initial report. However, it is very important that the investigator always make an assessment of causality for every event before the initial transmission of the SAE data. The investigator may change their opinion of causality in light of follow-up information and send a SAE follow-up report with the updated causality assessment. The causality assessment is one of the criteria used when

determining regulatory reporting requirements. If an SAE cannot be determined to be Not Causally Related to trial vaccine, it must be classified as Related.

Term	Definition
Not Related	There is no reasonable possibility of relationship to trial vaccine. The AE does not follow a reasonable temporal sequence from administration of trial vaccine or can be reasonably explained by the participant's clinical state or other factors (eg, concurrent diseases, and concomitant medications).
Related	There is a reasonable possibility of relationship to trial vaccine. The AE follows a reasonable temporal sequence from administration of trial vaccine and cannot be reasonably explained by the participant's clinical state or other factors (eg, concurrent diseases, or concomitant medications), represents a known reaction to trial vaccine or other vaccines in its class, is consistent with the known pharmacological properties of the trial vaccine, and/or resolves with discontinuation of the trial vaccine (and/or recurs with re-challenge, if applicable).

The investigator should consider the following, before reaching a decision on causality assessment:

- Time relationship between trial vaccine injection and event's onset.
- Re-challenge following second vaccine, if applicable.
- Medical history.
- Study treatment.
- Mechanism of action of trial vaccine.
- Class effect.
- Concomitant treatments in use.
- Withdrawal of study treatment.
- Lack of efficacy/worsening of existing condition.
- Possible vaccine enhancement of COVID-19.
- Erroneous treatment with study medication or concomitant medication.
- Protocol-related process.

10.3.4.3 Action Taken with Trial Vaccine Due to AE

The action taken with trial vaccine should be recorded using one of the following:

- No action taken.
- Next dose delayed.
- Permanently discontinued/withdrawn from further study vaccination (with date).
- Not applicable.

10.3.4.4 Other Action Taken

Details of any other actions taken should be specified:

- Specific therapy/medication.

- Surgical or medical procedure.
- Prolonged hospitalization.

10.3.4.5 AE Outcome

Each AE should be rated according to one of the following outcomes:

- Recovered/resolved.
- Recovering/resolving.
- Not recovered/not resolved.
- Recovered with sequelae/resolved with sequelae.
- Fatal.
- Unknown.

10.3.5 Follow-up of AEs/SAEs

All AEs should be followed up until resolution, unless in the investigator's opinion, the AE is unlikely to resolve and has become a chronic underlying disease. SAEs, MAAEs or AESIs ongoing at the time of EoS should be reported according to Section 10.3.6.

10.3.6 Reporting of SAEs

All SAEs must be reported according to International Council for Harmonisation (ICH) GCP or local regulations, applying the regulation with the stricter requirements. Investigators and other study site personnel must inform appropriate ICON representatives of any SAE that occurs during the course of the study, from the time of informed consent until the EoS visit, regardless of whether it is judged to be causally related to trial vaccine or procedures. Notification must occur within 24 hours of when they become aware of it. The investigator should make every effort to obtain follow-up information on the outcome until the event is considered resolved, chronic and/or stable.

If a non-serious AE becomes serious, this and other relevant follow-up information must also be provided to ICON within 24 hours as described above. The date when the AE becomes serious should be notated in the eCRF or on the SAE form.

All SAEs will also be recorded in the eCRF. The investigator is responsible for informing the IEC/IRB of the SAE as per local requirements. Paper SAE forms should be completed at the study site and emailed within 24 hours of study site awareness of the event to the Central Receipt mailbox:

[redacted]

The report form should be attached to the email; a notification email of the event describing it in the email text is not sufficient. There may be situations when an SAE has occurred and the investigator has minimal information to include in the initial SAE report. However, it is very important that the investigator always makes an assessment of causality for every event prior to transmission of the SAE report form.

Minimum criteria for a reportable event are:

- Identifiable patient (participant number)
- A suspect product (ie, trial vaccine)
- An identifiable reporting source (investigator/study site identification), and
- An event or outcome that can be identified as serious.

Follow-up information on SAEs must also be reported by the investigator within the same time frames.

10.3.6.1 Safety Reporting to Sponsor

ICON PVSS will forward the SAE and Pregnancy reports to the Sponsor's safety representative(s) within 1 business day or 3 calendar days (whichever is earlier) of becoming aware of it.

10.3.6.2 Safety Reporting to Health Authorities, Independent Ethics Committees/Institutional Review Boards and Investigators

ICON will notify the Sponsor of any SAE and will perform follow-up activities with the concerned study site. Novavax will retain responsibility of expedited and periodic reporting to the US FDA and ICON for all other reporting according to national requirements Procedure and timelines for safety reporting are provided in the Safety Management Plan as agreed by ICON and the Sponsor. The investigator must comply with any applicable study site-specific requirements related to the reporting of SAEs (particularly deaths and SUSARs) to the IEC/IRB that approved the study. Investigators should provide written documentation of IEC/IRB notification for each report to the ICON PVSS. In accordance with ICH GCP, ICON PVSS will inform the investigators of findings that could adversely affect the safety of participant, impact the conduct of the study, or alter the IEC's/IRB's approval/favorable opinion to continue the study, as assessed by the Sponsor. In particular and in line with respective regulations, ICON PVSS will inform the investigators of SAEs. The investigator should place copies of Safety Reports in the Investigator Site File. National regulations with respect to Safety Report notifications to investigators will be taken into account. When specifically required by regulations and guidelines, the ICON PVSS will provide appropriate Safety Reports directly to the concerned lead IEC/IRB and will maintain records of these notifications. When direct reporting is not clearly defined by national or study site-specific regulations, the investigator will be responsible for promptly notifying the concerned IEC/IRB of any Safety Reports provided by the ICON PVSS and of filing copies of all related correspondence in the Investigator Site File.

10.3.6.3 24/7 Medical Emergency Coverage for Urgent Protocol-related Medical Questions

In a study-related health emergency, when assigned medical monitors for a study cannot be reached by a caller, for discussion of urgent medical-related questions an on-call physician can be reached 24/7 via an ICON Call-Center:

- [redacted]

(Chargeable telephone number allowing global reach from both landlines and mobile phones)

- [redacted]

On this internet page, a list of country-specific toll-free telephone numbers is provided. It should be noted that not all countries globally have access to toll-free numbers as indicated on the "24/7 Medical Help-desk" index. Countries without toll-free numbers need to dial the chargeable number as indicated above. Furthermore, there may be restrictions when dialing toll-free numbers from a mobile phone.

10.3.7 Laboratory Assessments

Screening clinical laboratory tests will be performed at a designated laboratory. The following parameters will be collected, as described in Table 6:

Table 6 Clinical Laboratory Tests

Laboratory Testing Profile	Tests Included
Pregnancy testing	Only for participants of childbearing potential:<br>• Urine pregnancy test at screening and prior to each vaccination

10.3.8 Physical Examination

Targeted physical examination, as indicated by participants' reports of AEs, will be performed at the visits specified in the SoA (Table 1 and Table 2).

Height and weight will be measured and BMI will be calculated at screening only, unless a participant's clinical condition changes such that reassessment of weight and BMI is medically indicated, which should then be recorded as part of an AE, as indicated below.

Examination at screening to include vital signs, height and weight (calculated BMI), and lymphatic assessment of upper extremities to allow for vaccination. Symptom-directed (targeted) physical examination will be performed at other scheduled time points, with the inclusion of vital signs and O_2 saturation at the Acute Illness Visit. Targeted physical examination after Day 21 is optional, as needed for AE evaluation, except for vaccination visits where it is mandatory.

Any clinically significant changes from baseline should be recorded as AEs.

10.3.9 Vital Signs

Vital sign measurements will be recorded at screening and prior to vaccination on Days 0 and 21, and at the crossover vaccinations. Vital sign measurements, recorded as continuous variables, will include oral temperature (or via forehead/ear reader), respiratory rate and diastolic and systolic blood pressure (BP) (after participant is seated for at least 5 minutes). Pulse rate may be measured by pulse oximeter. Blood pressure should not exceed medically acceptable limits to ensure

participants with uncontrolled hypertension are not included. Participants considered to have "white coat hypertension" should have a reduction in BP documented following a calming period. Body temperature should not exceed 38°C.

Vital sign measurements will be recorded as continuous variables prior to each vaccination. On vaccination days, vital sign measurements will be collected once before vaccination to ensure participant has controlled BP and heart rate and no evidence of fever prior to vaccination. If individual vital sign measurements are considered clinically significant by the investigator, vaccination may be withheld that day (or, as noted for BP, delayed briefly pending a calming period), and participants may return on a subsequent day for re-evaluation and vaccination, ideally, within the time window specified in the SoA (Table 1 and Table 2).

10.3.10 Overdose

A drug overdose is defined as the accidental or intentional use of a drug or medicine or an administration error in an amount that is higher than is normally used. Every overdose must be reported to ICON PVSS within 24 hours of awareness, using the details provided in Section 10.3.6 if the overdose was associated with an SAE. Other overdoses and those associated with non-serious AEs should be reported in the eCRF AE page. Only overdoses associated with a clinical SAE needs to be reported as an SAE. The quantity and duration of the excess dose should be documented in the eCRF.

Overdose in this study is specifically defined as any dose greater than the intended protocol dose (Section 9). In case of overdose, it is recommended that the participant be monitored for any signs or symptoms of adverse reactions or effects and appropriate symptomatic treatment be administered immediately. Note that administration of the "wrong" vaccine is a protocol deviation, but not, in the absence of associated AE, an SAE.

10.4 Other Assessments

10.4.1 Medical and Social History

Medical and social history will capture prior and concomitant medical conditions, recent vaccinations (≤ 90 days), and significant surgical procedures. Living and working or school conditions will be recorded to assess possible high-risk environments.

10.4.2 Demographics

Demographic information will be collected at screening only and will include date of birth (day, month, and year), sex, race, ethnicity, weight, height, and BMI (derived).

10.4.3 eDiary

The eDiary is a validated data collection tool that generates an audit trail. Solicited AEs of reactogenicity (following initial set of vaccinations only) will be collected via participant or parent(s)/caregiver(s) reporting in the eDiary utilizing smartphone application(s). Participants,

parent(s) or caregiver(s) who do not possess an appropriate device will be provided a device compatible with study requirements. All participants and parent(s)/caregiver(s) will be trained on the use of these applications at the initiation of the study, and a Help Desk will be available 24/7 for technical issues. For data entry issues, participants should contact the study site.

Study site personnel and central monitoring personnel will regularly review the eDiary for completeness.

Centralized monitoring of the electronic database will enable identification of any potential issues related to participant retention, including study participants who have missed regular reporting or non-compliant.

10.5 Post Blinded Crossover

In the **Adult Main Study**, following blinded crossover, follow-up as specified for the first 12 months of study will continue to collect by remote contact safety and efficacy endpoints after crossover. Follow-up during Months 12-24 will continue via remote contacts every 3-6 months through study completion (except when a visit is scheduled and replaces the remote contact), with the intent to capture 2 years follow-up on each participant. Once all participants have had 1 year of follow-up from enrollment, an analysis will be executed to examine durability effect of the vaccine on efficacy endpoints, taking into account the timing of the blinded crossover as described in Follmann et al, 2020.

In the **Pediatric Expansion**, following blinded crossover, follow-up with weekly remote contacts will continue to collect safety and COVID-19 diagnoses after crossover through Month 12. Follow-up during Months 12-24 will continue via remote contacts every 3 months through study completion (except when a visit is scheduled and replaces the remote contact) with the intent to capture ≥12 months follow-up on each participant following receipt of active vaccine. An analysis of immune responses and "effectiveness" data may be prepared for regulatory submission after all Day 35 data have been collected. Once all participants have had 1 year of follow-up from enrollment, planned analysis will be executed to examine durability effect of the vaccine on efficacy endpoints, taking into account the timing of the blinded crossover as described in Follmann et al, 2020. Antibody persistence will be determined by collecting samples for effectiveness assessment at 12, 18 and 24 months post initial set of vaccinations, taking into account the timing of the blinded crossover.

11 MEDICAL RESOURCE UTILIZATION

In the **Adult Main Study**, healthcare requirements, utilization and medical assessments from participants who become ill on study will be defined in a separate substudy protocol.

12 STATISTICAL CONSIDERATIONS

12.1 Sample Size and Power

The sample size for the original study design in the **Adult Main Study** was driven by the total number of cases expected to achieve statistical significance for the primary efficacy endpoint; a total of up to approximately 30,000 participants ≥ 18 years of age would be enrolled to provide a target of 144 symptomatic COVID-19 illness PCR-confirmed SARS-CoV-2 infections. With the current change of the study design to perform a single efficacy analysis for the initial vaccination phase when the blinded crossover is implemented, the estimated (through simulations) powers to reject the null hypothesis of VE lower bound of 95% CI ≤ 30% and achieving the point estimate of VE ≥ 50% simultaneously for the primary endpoint is summarized in **Error! Reference source not found.**.

Power calculations were performed by 10,000 simulated trials that were created under various assumptions of VEs and analyzed using methods described in the "efficacy analysis" section without covariates. All simulations were performed in SAS V9.4.

Table 7 Power Under Various Number of Primary Efficacy Endpoints and Vaccine Efficacy Assumptions

Number of Endpoints	Assumed Vaccine Efficacy	Power
20	80%	81%
	85%	93%
	90%	99%
	95%	>99%
25	80%	85%
	85%	95%
	90%	>99%
	95%	>99%
30	80%	94%
	85%	99%
	90%	>99%
	95%	>99%
35	80%	95%
	85%	99%
	90%	>99%
	95%	>99%
40	80%	98%
	85%	>99%
	90%	>99%
	95%	>99%

Table 7 Power Under Various Number of Primary Efficacy Endpoints and Vaccine Efficacy Assumptions

Number of Endpoints	Assumed Vaccine Efficacy	Power
45	80%	98%
	85%	>99%
	90%	>99%
	95%	>99%
50	80%	99%
	85%	>99%
	90%	>99%
	95%	>99%

Table 8 provides example statistical success boundaries (ie, minimum VE needed to demonstrate lower bound of two-sided 95% confidence interval [LBCI] above 30%).

Table 8 Example Minimum Vaccine Efficacy Needed to Demonstrate Lower Bound of the Confidence Interval > 30%*

Total Number of Endpoints	Maximum Number of Endpoints in Active Arm	Vaccine Efficacy (%)	95% CI
10	2	87.5	(41.1, 97.3)
15	4	81.8	(42.9, 94.2)
20	7	73.1	(32.5, 89.3)
25	9	71.9	(36.4, 87.6)
30	12	66.7	(30.8, 83.9)
35	14	66.7	(34.5, 83.0)
40	17	63.0	(30.9, 80.2)
45	19	63.5	(34.0, 79.8)
50	22	60.7	(31.4, 77.5)
55	24	61.3	(34.1, 77.3)
60	27	59.1	(32.0, 75.4)
65	30	57.1	(30.3, 73.7)
70	32	57.9	(32.7, 73.7)
75	35	56.2	(31.2, 72.2)
80	37	57.0	(33.3, 72.3)

* Lower bound of two-sided 95% confidence interval with 18,000 and 9,000 per protocol analysis set participants for the active and placebo groups, respectively.

The analyses for the **Adult Main Study** will be performed separately from analyses for the **Pediatric Expansion**. Only participants ≥ 18 years of age at the time of randomization will be included for analyses of data collected in the **Adult Main Study**.

A total of up to approximately 1,200 adult participants will be randomly selected for the immunogenicity assessment (IgG antibody to SARS-CoV-2 S protein, MN, and hACE2 inhibition) at Days 0 and 35. A random selection of 600 participants per adult age cohort will include approximately 400 vaccine and 200 placebo recipients. This random selection may be carried out in multiple subsets throughout the study and may also include other stratification factor(s) to achieve analytical efficiency.

Additionally, after blinded crossover, adult participants (approximately 900 in the 18 to ≤ 64 years of age group) enrolled at a pre-selected subset of study sites will be assessed for IgG antibodies to SARS-CoV-2 S protein as part of the analysis of immunobridging. Given the 2:1 randomization and if this ratio holds for this set of participants, this selection will include approximately 300 vaccine (received placebo initially) and 600 placebo (received active vaccine initially) recipients from the crossover (delayed) phase, where the 300 vaccine recipients receive a dose of vaccine from a different manufacturing lot than received by the 600 placebo recipients who received active vaccine during the initial set of vaccinations. The IgG antibody concentrations induced by the initial set of vaccinations for the 600 placebo recipients will be used in the evaluation, while the IgG antibody concentrations induced by the crossover set of vaccinations will be used for the 300 vaccine recipients.

Table 9 shows the power to conclude non-inferiority of the lot used for the crossover phase, defined as the upper bound of the one-sided 95% CI on the ratio of geometric mean IgG antibody concentrations between the 2 lots ($GMC_{INITIAL\ LOT}$ / $GMC_{CROSSOVER\ LOT}$) being ≤ 1.5. Calculation for power uses a two sample t-test assessing difference of 2 logarithmic (base 10) means from populations with common variance and assumes 10% of participants are non-evaluable per group.

Table 9 Power to Conclude Non-Inferiority of the Crossover Lot

Number of Evaluable Participants Per Lot Used	Standard Deviation of log10 IgG Antibody Concentration	Type I Error (one-sided)	Power to Show Non-inferiority
Initial lot: 540<br>Crossover lot: 270	0.45	0.025	>99%

Abbreviations: IgG = immunoglobulin G.

Additional 2-stage random samplings are planned to facilitate the case-cohort sampling design for assessing immune correlates of risk and protection to be conducted in collaboration with external CoVPN and OWS statistical groups. The analytical approach including sampling plan details will be documented in a separate SAP to be developed prior to the unblinding by the external statistics groups.

The sample size for the **Pediatric Expansion** is chosen to provide an adequate safety database of ≥2,000 pediatric recipients of investigational product to support licensure of the SARS-CoV-2 rS Matrix-adjuvanted vaccine in pediatric participants 12 to < 18 years of age. Recruitment of study participants will attempt to enroll a similar number of participants in the 12 to < 15 and 15 to < 18 year old age groups. The enrollment of approximately 3,000 adolescent participants with a 2:1 randomization to active vaccine or placebo will provide a total of approximately 2,000 pediatric participants exposed to active vaccine.

The analysis of efficacy in the **Pediatric Expansion** will be descriptive in nature using the same methods as the **Adult Main Study** but with no formal statistical hypothesis tested. Table 10 shows the estimated powers (through simulations) to demonstrate a lower bound for VE ≥ 0% given various assumptions of VE.

Table 10 Power Under Various Number of Primary Efficacy Endpoints and True Vaccine Efficacy Assumptions to Conclude VE > 0%

Number of Endpoints	Assumed Vaccine Efficacy	Power*
10	75%	56%
	80%	69%
	85%	82%
	90%	93%
	95%	99%
15	75%	80%
	80%	89%
	85%	96%
	90%	99%
	95%	>99%
20	75%	90%
	80%	96%
	85%	99%
	90%	>99%
	95%	>99%

* Power (based on simulation) to rule out VE ≤ 0% given sample sizes of 1,800 subjects in the SARS-CoV-2 rS group and 900 subjects in the placebo group using a two-sided 95% CI.

With 2,000 participants in the active vaccine group, there is a >90% probability of observing at least 1 participant with an AE if the true incidence of the AE is 0.12% and a 99% probability if the true incidence of the AE is 0.23%. With 2,000 participants receiving at least 1 vaccine dose, there is a >99% probability of observing at least 1 participant with an AE if the true incidence of the AE is at least 0.12%.

The blinded crossover ~6 months after completion of the initial vaccine series and 12 months follow-up after crossover will aim to collect ≥ 12 months safety data after receipt of the active vaccine.

A non-randomized non-inferiority (NI) analysis of immunogenicity will be performed between the adolescents in the **Pediatric Expansion** of the study and the 18 to < 26 years old adult population in the **Adult Main Study**.

A separate random sample of 750 participants aged 18 to < 26 years of age from the **Adult Main Study** will be drawn to provide approximately 400 participants for the NI analysis, accounting for the 2:1 randomization and 20% non-evaluability. Similarly, 750 participants will be randomly selected from the **Pediatric Expansion** for testing of neutralization titers, which will provide approximately 400 participants for the NI analysis, accounting for the 2:1 randomization and 20% non-evaluability.

For the effectiveness endpoint, successful demonstration of non-inferiority will require meeting the following 3 pre-specified criteria simultaneously,

1. upper bound of 2-sided 95% CI for the ratio of GMTs ($GMT_{18\text{-}<26yo}/GMT_{12\text{-}<18yo}$) < 1.5,
2. point estimate of the ratio of GMTs ≤ 1.22 (estimated as square root of 1.5)
3. upper bound of the 2-sided 95% CI for difference of seroresponse rates ($SRR_{18\text{-}<26yo}$ - $SRR_{12\text{-}<18yo}$) is < 10%

With 400 evaluable participants (500 accounting for 20% non-evaluability) in the active vaccine group randomly selected from each of the 18 to < 26 year old subset of participants in the **Adult Main Study** and the **Pediatric Expansion**, there is over 85% power (through simulations) to demonstrate the first 2 non-inferiority criteria when assuming an underlying GMT for the 18 to < 26 years of age group up to 1.1-fold higher than the 12 to < 18 years of age group.

The table below shows the power to conclude non-inferiority based on success criteria for GMT ratio ($GMT_{18\text{-}<26yo}/GMT_{12\text{-}<18yo}$).

Number of Evaluable Participants Per Age Group	Standard Deviation of log10 MN Antibody Titer	True GMT Ratio (18-<26yo / 12-<18yo)	Power to Show Non-inferiority
400	0.6	1.0	97.9%
		1.05	93.7%
		1.1	85.5%
		1.15	72.7%
		1.2	56.6%
		1.25	40.1%
		1.3	25.7%

The table below shows the power to demonstrate the third non-inferiority criterion given various assumptions for seroresponse rates and an evaluable sample of 400 in each of the 18 to < 26 and 12 to < 18 years of age groups. In the absence of an established correlate of protection for SARS-CoV-2 vaccines, seroresponse will be defined as ≥ 4-fold increase in neutralization titers (MN_{50}) at Day 35 relative to baseline titers. With this definition and assumed seroresponse rates (SRR) of 95% in the 18 to < 26 years of age group, there is over 80% power to demonstrate the third non-inferiority criterion for a difference as large as 4% lower in the 12 to < 18 years of age group. A descriptive assessment of immunogenicity will evaluate the same criteria in the 12 to < 15 and 15 to < 18 years of age groups separately.

Seroresponse rate 18 to < 26 years of age group	Seroresponse rate 12 to < 18 years of age group	Power to Show Non-inferiority
95%	90%	72.9%
94%	90%	85.2%
93%	90%	92.9%
92%	90%	96.9%
91%	90%	98.8%
99%	95%	99.2%
98%	95%	>99%
97%	95%	>99%
96%	95%	>99%

12.2 Analysis Sets

The following analysis sets are identified for analysis.

12.2.1 Intent-to-Treat (ITT) Analysis Set

In the **Adult Main Study** and **Pediatric Expansion**, the ITT analysis sets will include all participants who are randomized, regardless of protocol violations or missing data. The ITT analysis set will be used for participant disposition summaries and will be analyzed according to the treatment arm to which the participant was randomized.

12.2.2 Full Analysis Set (FAS)

In the **Adult Main Study** and **Pediatric Expansion**, the FAS will include all participants who are randomized and received at least 1 dose of study vaccine/placebo, regardless of protocol violations or missing data. Participants who are unblinded with an intention to receive other COVID-19 vaccines will be censored at the time of unblinding. The FAS population will be analyzed according to the treatment group to which they were randomized. The FAS analysis sets will be used for supportive analyses.

12.2.3 Safety Analysis Set

In the **Adult Main Study** and **Pediatric Expansion**, the safety analysis sets (for each age stratum) will include all participants who receive at least 1 dose of trial vaccine. Participants in the safety

analysis set will be analyzed according to the vaccine actually received. In cases where information is available that indicates that a participant received both active and placebo, the participant will be analyzed as part of the active group. Participants who unblind with intention to receive another COVID-19 vaccine (under EUA or otherwise licensed) or after unblinding due to dosing error (see Section 9.1) will be censored at the time of unblinding.

12.2.4 Per-Protocol Efficacy (PP-EFF) Analysis Set

In the **Adult Main Study** and **Pediatric Expansion**, the PP-EFF analysis sets (for each age stratum) will include all adult participants who receive the full prescribed initial regimen of trial vaccine and have no major protocol deviations that occur before the first COVID-19 PCR-positive episode (ie, participant will be censored at the time of the protocol deviation) and are determined to affect the efficacy outcomes, including baseline SARS-CoV-2 seropositivity or nasal swab PCR-positivity. Participants who are unblinded with an intention to receive other COVID-19 vaccines will be censored at the time of unblinding. Although the study will enroll participants regardless of SARS-CoV-2 serologic status at the time of initial vaccination, any participants with confirmed infection or prior infection due to SARS-CoV-2 at baseline, by nasal swab PCR or serology, will be excluded from the PP-EFF population. PP-EFF will be the primary set for all efficacy endpoints. Participants determined to have positive nasal swab PCR or serology immediately prior to the first crossover vaccination will be excluded from the post-crossover PP-EFF population.

A second per-protocol efficacy (PP-EFF-2) analysis set is defined to allow for evaluation of baseline serostatus analysis's impact on vaccine efficacy. The PP-EFF-2 analysis set will follow the same method described in the PP-EFF population with the exception that it will include all participants regardless of baseline serostatus.

The review and determination for exclusion from the PP-EFF and PP-EFF-2 analysis sets will be carried out in a blinded fashion by a study clinician based on all available information prior to unblinding.

12.2.5 Per-Protocol Immunogenicity (PP-IMM) Analysis Set

In the **Adult Main Study** and **Pediatric Expansion**, the PP-IMM analysis set will be determined for each study visit. Immediate immune reaction to study vaccine will be evaluated after the initial set of vaccinations. The PP-IMM analysis set will include participants that have at least a baseline and 1 serum sample result available after vaccination and have no major protocol violations that are considered clinically relevant to impact immunological measures at the visit in question. All participants in the PP-IMM analysis populations will be designated at time of vaccination within the immunogenicity subset. For participant visits on or after Day 21, participants must receive the second vaccination to be included in the PP-IMM analysis set. Prior exposed participants will be determined using baseline SARS-CoV-2 nasal swab or seropositivity at screening to assess if immune responses differ between previously exposed and unexposed individuals. Durability of

immune responses will be evaluated in participants who provide serologic data at Months 12, 18 and 24, taking into account when they received active vaccine and if/when they were infected with SARS-CoV-2, based on PCR or serology.

Adult participants in the PP-IMM population for the immunobridging analysis will be those at selected study sites who receive the two crossover vaccination doses and have blood drawn for anti-S IgG antibodies immediately prior to the first crossover vaccination dose and approximately 14 days after the second crossover vaccination dose.

The review and determination for exclusion from the PP-IMM analysis set will be carried out in a blinded fashion by a study clinician based on all available information prior to unblinding.

12.2.6 Participant Disposition

A CONSORT diagram and table displays will be generated to present the number of participants screened, the number enrolled and eligible for vaccination, the number randomized to each trial vaccine arm, the number receiving the first and second vaccination, the number of early terminations, the number completing the study, and the number eligible for analysis. Displays of participants included and excluded from each analysis population along with the reason for exclusion will be provided by trial vaccine arm. A review of participant disposition will also be summarized by age strata, race, ethnicity, sex at birth, and country.

12.3 Statistical Analyses

The SAP will include a more technical and detailed description of the statistical analyses. This section is a summary of the planned statistical analyses of the most important endpoints including primary and secondary endpoints. Primary and secondary efficacy and safety endpoints will be evaluated by key demographic characteristics, such as race, ethnicity, sex at birth, and country. In the **Adult Main Study**, these endpoints will also be analyzed by age stratum, while in the **Pediatric Expansion**, they may be analyzed by age group, even though no formal age sub-stratification is planned.

Primary analysis of the primary and the key secondary efficacy endpoints will be performed based on the data generated up to crossover or a cutoff date after initiation of the blinded crossover (whichever is earlier) for each individual participant, which is considered end of follow-up for the participant in the initial pre-crossover period. The analysis of data generated after the blinded crossover or the combined analyses of both pre- and post-blinded crossover will be performed using the approach described by Follmann et al [Follmann 2020]. Additional details on the analytical approach will be described in the Statistical Analysis Plan.

At the analysis of primary and key secondary efficacy endpoints, the study will remain blinded at the participant level for study site personnel and study participants until the end of the study (24 months after the first vaccination), while the Sponsor will be unblinded at the participant level to

prepare for regulatory submissions. There will be an unblinded statistician and programmer isolated (by firewall), from study personnel that prepare these analyses.

12.3.1 Primary Endpoint

In the **Adult Main Study**, the primary endpoint will be analyzed on the PP-EFF analysis set and supported by analysis of the FAS analysis set.

The VE is defined as VE (%) = (1 – RR) x 100, where RR = relative risk of incidence rates between the 2 trial vaccine groups (SARS-CoV-2 rS / Placebo). The RR will be estimated by exponentiating the treatment group coefficient from a Poisson regression analysis with robust error variance [Zou 2004]. The age strata will be included in the model as a covariate. To assess incidence rates rather than absolute counts of cases, accounting for differences in follow-up times starting with 7 days after the second vaccination among participants, an offset will be utilized in the Poisson regression. A two-sided, 95% confidence interval (CI) will be constructed around the estimate.

A super superiority of the vaccine efficacy at each analysis will be used to determine success of the primary endpoint. A hypothesis test with a one-sided Type I error of 2.5% will be conducted with the following hypotheses:

H_0: VE $\leq$ 0.30 (RR $\geq$ 0.70)

H_1: VE $>$ 0.30 (RR $<$ 0.70)

Rejection of the null hypothesis demonstrates a statistically significant VE with a lower bound of CI > 30%. In order to be considered for EUA by the FDA, a vaccine must show super superiority where there is a minimum VE of 50% and a lower bound of two-sided 95% confidence bound of at least 30%. Based upon the number of primary efficacy endpoints planned for analysis, a lower bound of more than 30% corresponds with a VE point estimate of at least 50%.

12.3.1.1 Efficacy

In the **Pediatric Expansion**, the primary endpoint will be analyzed on the PP-EFF analysis set and supported by analysis of the FAS analysis set. The statistical method used will be identical to the **Adult Main Study**. However, the analysis will be descriptive in nature with no formal statistical hypothesis tested.

The VE is defined as VE (%) = (1 – RR) x 100, where RR = relative risk of incidence rates between the 2 trial vaccine groups (SARS-CoV-2 rS / Placebo). The RR will be estimated by exponentiating the treatment group coefficient from a Poisson regression analysis with robust error variance [Zou 2004]. To assess incidence rates rather than absolute counts of cases, accounting for differences in follow-up times starting with 7 days after the second vaccination among participants, an offset will be utilized in the Poisson regression. A two-sided, 95% confidence interval (CI) will be constructed around the estimate.

In the **Pediatric Expansion**, formal non-randomized non-inferiority (NI) analysis of the primary effectiveness endpoint, neutralizing antibody to SARS-CoV-2 at Day 35, will be carried out using the PP-IMM analysis set. The analysis of the two pre-specified NI success criteria for ratio of GMTs comparing adolescents with the 18 to < 26 year old immunogenicity cohort from the **Adult Main Study** will be performed using the point estimate and upper bound of the two-sided 95% Confidence Interval (CI) on the ratio of GMTs between 2 age cohorts (adults 18 to <26 year old cohort in the **Adult Main Study**/adolescent cohort in the **Pediatric Expansion**). The ratio of GMTs between 2 age cohorts and the corresponding two-sided 95% CI will be calculated on log-transformed titers using the analysis of covariance (ANCOVA) with treatment group and baseline (Day 0) measurement as the covariate. The evaluation of the pre-specified NI success criterion for difference of seroresponse rates between 2 age cohorts (adolescents minus adults 18 to < 26 year old will be based on a 95% CI constructed with the method of Miettinen and Nurminen. A descriptive assessment of immunogenicity will evaluate the same criteria in the 12 to < 15 and 15 to < 18 years of age groups separately.

12.3.2 Secondary Endpoints

12.3.2.1 Adult Main Study and Pediatric Expansion

In the **Adult Main Study only**, the key secondary efficacy endpoint and other secondary efficacy endpoints will be analyzed using the same manner as the primary efficacy analysis described in protocol Section 12.3.1. The analysis of the key secondary endpoint will be carried out using a one-sided alpha of 0.025 only after the successful demonstration the primary endpoint to preserve the Type I error rate. All remaining secondary efficacy endpoints will also be performed using an unadjusted two-sided 0.05 alpha level.

For the moderate-to-severe efficacy endpoint, severe cases will be counted from potentially severe cases reviewed and confirmed by an external endpoint review committee in a blinded manner. Details of this endpoint committee will be in the Statistical Analysis Plan.

The secondary immunogenicity analyses will be performed using the PP-IMM analysis set and FAS.

For the immunobridging analysis in the **Adult Main Study**, the ratio of geometric mean IgG antibody concentrations will be computed at Day 35 for the initial lot versus the crossover lot using the data from the participants selected for analysis as described in Section 12.1. The 95% CI will be calculated from a statistical model described in Section 12.3.3.

In the **Pediatric Expansion**, analyses of secondary efficacy endpoints are descriptive in nature with no formal statistical hypothesis testing.

For the serum antibody levels specific for the SARS-CoV-2 S protein antigen(s) (IgG antibody to SARS-CoV-2 S protein and hACE2 inhibition) and MN, the geometric mean at each study visit, the geometric mean fold rise (GMFR) comparing to the baseline (Day 0) at each post-vaccination

study visit, and the GMFR comparing pre- and post-second dose, along with 95% CI will be summarized by trial vaccine group. The 95% CI will be calculated based on the t distribution of the log-transformed values for geometric means or GMFR, then back transformed to the original scale for presentation. Seroresponse rate (SRR) will also be calculated with exact 95% CI. Seroresponse is defined as achieving a 4-fold rise in antibody response from baseline at Day 35.

Similar summaries will be generated for the other immunogenicity endpoints and other assays if conducted.

In the **Pediatric Expansion**, descriptive analysis without a pre-specified NI margin will be carried out for the serum antibody levels specific for the SARS-CoV-2 S protein antigen(s) (IgG antibody to SARS-CoV-2 S protein and hACE2 inhibition) at Day 35 using the same statistical method as the primary endpoint. Evaluation of immune responses will also assess the 12 to < 15 and 15 to < 18 years age groups of the **Pediatric Expansion**. The secondary immunogenicity analyses will be performed using the PP-IMM analysis set and the FAS. The analysis of SCR will include calculation of the difference of SCR between groups with a 95% CI computed with the method of Miettinen and Nurminen.

12.3.3 Statistical Models

For analysis of the efficacy endpoints in the **Adult Main Study**, the RR and its CI will be estimated using Poisson regression with robust error variance [Zou 2004]. The generalized linear model with unstructured correlation matrix (robust error variances) will be used. The explanatory variables in the model will include the trial vaccine group. The dependent variable will be the incidence rate of the endpoint of interest. The robust error variances will be estimated using repeated statement and the participant identifier. The age strata will be included in the model as a covariate. To account for the censoring in the analysis, the offset will be defined as the natural log of the time from the start of follow-up (7 days post second vaccination) to the outcome of interest or to the end of study. Poisson distribution will be used with a logarithmic link function. The following is a sample of SAS code used to estimate the RR:

```
proc genmod data = <DATASET>;
      class armcd usubjid agestrata;
      model <OUTCOME> = armcd agestrata / dist = poisson link = log
      offset=<LN(TIMEVAR)>;
      repeated subject = usubjid/ type = unstr;
      estimate 'Beta' <armcd> 1 -1/ exp;
run;
```

A Cox proportional hazards model with the age strata as a covariate will also be developed as a supportive analysis to the Poisson regression. The model will follow the same explanatory and dependent variables as the Poisson model and will censor participants based on their follow-up time available.

For the **Pediatric Expansion**, the same Poisson regression and Cox proportional hazards models will be used, with exception that there is no age stratum included as covariate.

In the case where there are zero endpoints for one of the vaccine groups, the Poisson model will be substituted with an exact method to be detailed in the Statistical Analysis Plan.

For the immunobridging objective in the **Adult Main Study**, the two-sided 95% CI for the ratio of IgG antibody concentration (Initial Lot / Crossover Lot) will be computed using an analysis of variance (ANCOVA) model on the log-transformed antibody concentrations. The ANCOVA model will include vaccine group as a fixed effect and the baseline pre-vaccination concentration as a covariate.

Non-inferiority of the neutralization titer response for 12 to < 18 years of age group compared to the 18 to < 26 years of age group will be demonstrated if the upper-bound of the two-sided 95% CI for the ratio of GMTs ($GMT_{18\text{-}<26yo}/GMT_{12\text{-}<18yo}$) at Day 35 is < 1.5, the point estimate of the ratio of GMTs ≤ 1.22, and the upper bound of the two-sided 95% CI for difference of seroresponse rates ($SRR_{18\text{-}<26yo}$ - $SRR_{12\text{-}<18yo}$) is < 10%.

12.3.4 Handling of Missing Data

No imputation of missing primary or secondary endpoints will be *a priori,* to define the success of this study. All data recorded on the eCRF will be included in data listings and included in the clinical study report.

To assess the impact of missing values on the primary conclusions of this study, a tipping point analysis method will be conducted. The missing endpoint results in the control group will be imputed as non-endpoint, while participants in the active trial vaccine group with missing endpoint results will be imputed as an endpoint. If the primary endpoint is found to be significant, it will be assumed that the missing data has no impact on the conclusions of the study. If the primary endpoint is no longer significant a grid will be constructed of all possible ($mv+1$) by ($mp+1$) imputed outcomes for missing values by assigning imputed number of "endpoints" from 0 to the number in the vaccine group (mv) and 0 to the number in the placebo group (mp). For each possible imputed outcome, the overall VE and the corresponding CI will be constructed using the same statistical method used for the primary endpoint. The imputed data points in the grid will be evaluated against the H0: VE ≤ 30%. This grid will allow for an assessment of the impact of missing data on the primary conclusions of the trial.

Any further imputations required for reporting of AEs, medical history, and medications will be defined in the SAP.

12.3.5 Safety Analyses

In formal analyses, numbers and percentages (with 95% CIs based on the Clopper-Pearson method) of participants with solicited local and systemic AEs through 7 days after each vaccination of the initial set of vaccinations will be summarized by trial vaccine group and the maximum

toxicity grade over 7 days after each vaccination. The duration of solicited local and systemic AEs after each vaccination will also be summarized by trial vaccine group. Reactogenicity will not be collected following the crossover set of vaccinations.

Unsolicited AEs will be coded by preferred term and system organ class using the latest version of the Medical Dictionary for Regulatory Activities (MedDRA) and summarized by trial vaccine group as well as by severity and relationship to trial vaccine. AEs through 28 days after second injection of each set of vaccinations (initial and crossover); all MAAEs related to vaccine, SAE, or AESI through EoS will be listed separately and summarized by trial vaccine group. Participants who are unblinded to treatment assignment will be requested to report SAE, MAAE, AESIs and COVID-19 diagnoses by remote contact at the remaining prespecified time points following unblinding (see Table 1).

Participants who choose to be unblinded prior to the blinded crossover and receive EUA-authorized vaccine will be followed for safety (SAE, MAAE, AESI and COVID-19 diagnosis) by remote contact on the remaining schedule as specified in Table 1 and Table 2.

Vital sign measurements will be summarized by trial vaccine group at each time point using descriptive statistics.

Concomitant medications will be summarized by trial vaccine group and preferred drug name as coded using the WHO drug dictionary.

12.4 Interim Analysis

12.4.1 Adult Main Study and Pediatric Expansion

There are no formal interim analyses planned that require adjustment to Type I error.

Analyses planned before end of study include a single analysis of primary and secondary efficacy endpoints in the **Adult Main Study and** a subsequent analysis of data from the **Pediatric Expansion** at a cutoff point after initiation of the blinded crossover when participant data up to the crossover or cutoff date (whichever is earlier) has been accumulated and cleaned for all subjects. An analysis of safety and immunogenicity at Day 35 for adolescents (or at a later time point) may be conducted for the **Pediatric Expansion also prior to the end of the study.** For the **Adult Main Study** and **Pediatric Expansion**, after Month 12, an analysis will be performed to examine durability effect of the vaccine on efficacy endpoints as described in the next section. The analysis at Month 12 will also report on safety follow-up through 1 year.

12.5 Monitoring Potential Vaccine Harm

In the **Adult Main Study** , the DSMB will monitor the study for potential vaccine harm based on imbalance in the primary efficacy endpoint, ie, all mild, moderate or severe COVID-19 cases, and severe COVID-19 cases between SARS-CoV-2 rS with Matrix-M1 adjuvant versus placebo. In order to facilitate the timely identification of the severe COVID-19 cases, the cases will be

ascertained through both the efficacy surveillance and the SAE reporting. Monitoring for vaccine harm will be based on the Safety Analysis Set, defined as all randomized participants who received at least 1 dose of study vaccination. Participants will be analyzed according to the study vaccination they actually received regardless of the group to which they were randomized. For harm monitoring, cases will be counted starting on Day 0 after the first dose of study vaccination. Monitoring for harm will be performed at identification of each analysis-ready COVID-19 case once the 8th COVID-19 case has occurred, or the 8th severe COVID-19 case at any time post first vaccination (whichever is identified earlier). The 8th case was chosen because that is the minimum number of total cases required to reach the harm monitoring boundary if all 8 cases occur in the vaccine group.

The ICON unblinded statistician will continuously monitor the trial (ie, examine the data after each mild, moderate and severe COVID-19 case and each severe COVID-19 case, respectively) for early evidence of a potential elevated rate of mild, moderate and severe COVID-19 or severe COVID-19 in the SARS-CoV-2 rS with Matrix-M1 adjuvant group compared to the placebo group, in the Safety Analysis Set. The DSMB chair will be notified on a weekly basis as to the status of the number of cases observed and if the boundary conditions have been crossed. If the prespecified stopping boundary is reached for either mild, moderate and severe COVID-19 or severe COVID-19, then the ICON unblinded statistician will immediately inform the DSMB that the harm rules have been met. This monitoring guideline is chosen to allow stopping for prudence as early as possible, maximizing participant safety. In addition, IRBs/IECs will be notified if halting criteria are met and of any decisions made by the DSMB.

Potential vaccine harm is continuously monitored by evaluating number of mild, moderate and severe COVID-19 and severe COVID-19 cases separately, in the vaccine and placebo arms. The monitoring for each is implemented with exact one-sided binomial tests of H0: $p \leq 2/3$ versus H1: $p > 2/3$ (ie, H0: $RR \leq 1.0$ versus H1: $RR > 1.0$, where RR is relative risk of the rate in the SARS-CoV-2 rS with Matrix-M1 adjuvant group over the rate in the placebo group), where p is the probability that a case participant would be in the SARS-CoV-2 rS with Matrix-M1 adjuvant group given the total number of cases. The bounds for harm monitoring are based on the assumption that VE = 0% (corresponds to $p = 2/3$ or 0.67). The actual ratio, p, between the 2 treatment groups will be re-established once the final number of participants for the Safety Analysis Set have been determined.

For mild, moderate or severe COVID-19, each test is performed at one-sided type I error rate of 0.05.

For severe COVID-19, monitoring of severe COVID-19 cases will be performed using a similar approach as that for COVID-19, to allow early stopping if there is evidence of an elevated rate of severe disease in the vaccine versus placebo group. The monitoring for potential harm based on the number of severe COVID-19 cases will also continue at least until the final analysis of the

primary endpoint. Figure 1 presents example stopping boundaries based on the planned randomization ratio of 2:1 (see Appendix 3 for detailed list of example boundaries).

For the **Pediatric Expansion** portion of the study, monitoring of possible harm and any other potential safety issues will be overseen by the Sponsor, in close collaboration with the DSMB.

Figure 1 Example of Stopping Boundaries for Potential Vaccine Harm

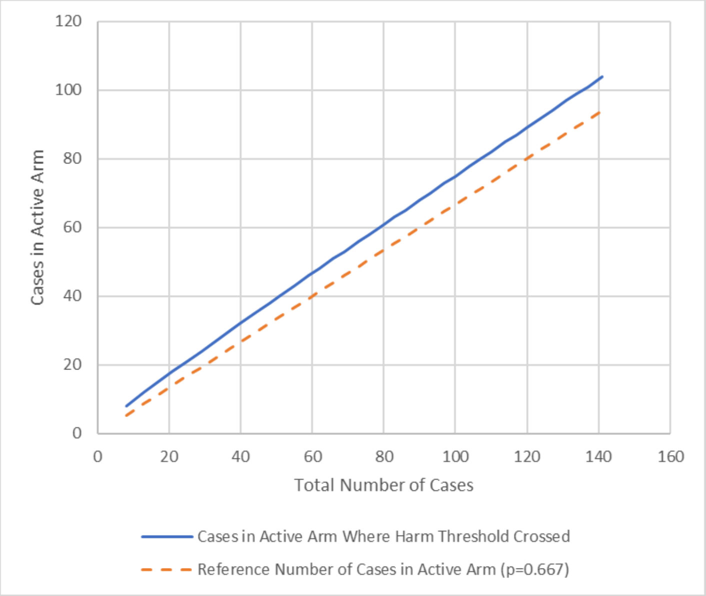

Stopping bounds for potential vaccine harm are based on exact binomial test conditional on the total number of cases, each test to be performed at one-sided alpha of 0.05.

12.6 Safety Monitoring

This protocol has extensive safety monitoring in place. Safety is monitored routinely by the ICON Medical Monitor, Novavax Pharmacovigilance and Safety Surveillance Physicians, Novavax Clinical Development Leads and routinely by the 2019nCoV-301 PSRT (for **Adult Main Study** only). In addition, for **Adult Main Study** and **Pediatric Expansion,** an independent DSMB periodically reviews study data, including unblinded study data if/when needed. The short-term safety of the adolescent cohort (ie, analysis of 7-day reactogenicity and safety in the first ~60 participants enrolled in an approximately even distribution across age groups 15 to < 18 years and 12 to < 15 years will be evaluated before enrollment of the remainder of the adolescent cohort will be reviewed by the DSMB. Likewise, analysis of 7-day reactogenicity and safety after receipt of the second vaccine dose in the first ~60 participants enrolled will be reviewed by the DSMB before the second vaccine dose is administered to the remainder of the adolescent participants. The safety data will be simultaneously shared and reviewed internally by the Sponsor.

Finally, the DSMB may recommend that the trial be terminated or that specific groups be withdrawn from the study, if any subgroup manifests serious or widespread adverse side effects. The DSMB will be informed immediately by the ICON unblinded statistician if the pre-specified

stopping boundary is met in the **Adult Main Study**, indicating that the vaccine causes harm by increasing the rate of mild, moderate or severe COVID-19. In addition, the DSMB will monitor the study for high vaccine efficacy or for futility to detect vaccine activity.

The 2019nCoV-301 PSRT is composed of the following members: medical and statistical representatives from the Sponsor, ICON and CoVPN network, as outlined in the PSRT Charter.

- Novavax study responsible Safety Physician
- Novavax Global Medical Lead, Clinical Development
- DAIDS medical officer representative
- Protocol chair and co-chairs
- BARDA Clinical Team Physician
- CoVPN Protocol Team Lead
- OWS Protocol Team Lead
- ICON Medical Monitor

The clinician members of the 2019nCoV-301 PSRT are responsible for decisions related to participant safety, as outlined in the PSRT Charter.

The Protocol Team clinic coordinator, clinical data manager, vaccine developer representative, clinical trial manager, and others may also be included in the 2019nCoV-301 PSRT meetings.

12.7 Data Safety and Monitoring Board

In the **Adult Main Study** and **Pediatric Expansion**, a centralized DSMB will be established in collaboration with NIH, NIAID, BARDA and Novavax according to the charter dictated by the participating groups. This group will then review interim unblinded data regularly in the **Adult Main Study** and **Pediatric Expansion** and make recommendations with respect to safety and emerging efficacy.

The NIAID DSMB assesses the effects of the study vaccine during the trial, provides other monitoring as described in Section 12.4, and may give advice to the 2019nCoV-301 Protocol Team leadership, the Oversight Group, and PSRT. More details on the role of and the data provided to the DSMB will be described in a DSMB Charter and SAP.

The DSMB will periodically review accumulating unblinded safety data by group. Prior to each meeting, the ICON unblinded statistician will provide the DSMB with data as described in SAP. Reports will be cumulative, generated from an up-to-date data file. Based on the reports, the DSMB will determine whether to recommend that the study should be continued, modified, or stopped, including for safety reasons.

The DSMB may recommend any steps to ensure the safety of study participants and the integrity of the trial. Furthermore, the DSMB may recommend that the trial be terminated or that specific groups be withdrawn from the study, if any subgroup manifests serious or widespread side effects.

To guarantee the unrestricted performance of its task, the DSMB may receive the individual study morbidity and mortality data from the ICON unblinded statistician.

Applicable to the **Adult Main Study**, the DSMB will be informed immediately by the ICON unblinded statistician if the pre-specified stopping boundary is met, indicating that the vaccine causes harm by increasing the rate of mild, moderate or severe COVID-19. Monitoring of possible harm and any other potential safety issues in the **Pediatric Expansion** will be overseen by the Sponsor, in close collaboration with the DSMB.

13 ETHICS

13.1 Independent Ethics Committee/Institutional Review Board

Prior to the start of the study, the investigator is responsible for ensuring that the protocol and consent form have been reviewed and approved by a relevant IEC/IRB. The IEC/IRB shall be appropriately constituted and perform its functions in accordance with FDA, ICH GCP and local requirements as applicable.

The IEC/IRB shall approve all protocol amendments (except for logistical or administrative changes), written informed consent documents and document updates, participant recruitment procedures (eg, advertisements), written information to be provided to the participants, IB, available safety information, information about payment and compensation available to participants, the investigator's curriculum vitae and/or other evidence of qualifications and any other documents requested by the IEC/IRB and Regulatory Authority (Competent Authority) as applicable.

13.2 Documentation of Informed Consent

The nature and purpose of the study shall be fully explained to each participant (or their legally responsible guardian). They must be informed that participation is voluntary.

Documentation of informed consent/assent forms (either written or via eConsent) must be obtained from each participant (or authorized representative) prior to any study procedures being performed. The process of obtaining informed consent must be documented in the participant’s source documents. The authorized person obtaining the informed consent must also sign the ICF, and a copy of the ICF must be provided to the participant or the participant’s legally authorized representative. Participants must be re-consented to the most current version of the ICF during their participation in the study.

Participants will be requested to provide the name and contact information for an emergency contact and to provide consent for serum banking for future testing to support establishment of correlates of protection against SARS-CoV-2 infection and disease.

The consent documents to be used for the study shall include all the elements of informed consent as outlined in accordance with FDA, ICH GCP and local requirements as applicable and be reviewed and approved by the appropriate IEC/IRB prior to use.

14 QUALITY CONTROL AND QUALITY ASSURANCE

14.1 Conduct of the Study

The Sponsor/designee shall implement and maintain quality control and quality assurance procedures with written standard operating procedures (SOPs) to ensure that the study is conducted and data are generated, documented and reported in compliance with the protocol, ICH GCP and applicable regulatory requirements.

This study shall be conducted in accordance with the provisions of the Declaration of Helsinki (October 2013), FDA (US Title 21 CFR, Part 312, Sections 312.50 and 312.56), European Union (EU) (536/2014) and with ICH GCP (CPMP/ICH/135/95 and updates).

The investigator will be responsible for the following:

1. Providing written summaries of the status of the study to the IRB/IEC annually or more frequently in accordance with the requirements, policies, and procedures established by the IRB/IEC.
2. Notifying the IRB/IEC of SAEs or other significant safety findings as required by IRB/IEC procedures.

The investigator may not deviate from the protocol without a formal protocol amendment having been established and approved by an appropriate IEC/IRB, except when necessary to eliminate immediate hazards to the participant or when the change(s) involve(s) only logistical or administrative aspects of the study. Any deviations may result in the participant having to be withdrawn from the study and render that participant non-evaluable.

The identification and reporting of serious breaches of ICH GCP or the protocol to the Regulatory Authorities and Ethics Committees will be conducted according to local SOPs and regulations.

14.2 Site Monitoring

The monitoring strategy for the study foresees a risk-based monitoring approach, in line with the relevant FDA and EMA recommendations, and will be described in detail by the study-specific risk-based Monitoring Plan.

Study site monitoring includes both source data review and source data verification. Study site monitors perform source data review of critical procedures to ensure that the safety and rights of participants are being protected and that the study is being conducted in accordance with the currently approved protocol, any other study agreements, ICH GCP, and all applicable regulatory requirements. Study site monitors perform source data verification of critical data to confirm transcription of data entered into the eCRF by authorized study site personnel are accurate, complete, and verifiable from source documents.

Monitoring details describing strategy (eg, risk-based initiatives in operations/quality such as Risk Management, Mitigation Strategies and Analytical Risk-Based Monitoring), methods, responsibilities and requirements, including handling of non-compliance issues and monitoring techniques (central, remote, or on-site monitoring) are provided in the Monitoring Plan.

The investigator, as part of his/her responsibilities, is expected to co-operate with ICON in ensuring that the study adheres to GCP requirements. The investigator must maintain accurate documentation (source data) that supports the information entered in the eCRF. The investigator may not recruit participants into the study until such time that a visit, or with the agreement of the Sponsor, attendance at the investigator meeting (or equivalent training), has been made by a Sponsor/ICON monitor to conduct a detailed review of the protocol, source documents and eCRF.

The investigator shall grant direct access of original source documents and study records to the ICON Site Monitor in order to conduct source data review, to ensure that the participants' well-being is maintained, data are being recorded in an adequate manner according to ALCOAC principles (ie, that they are attributable, legible, contemporaneous, original, accurate, and complete), that protocol and GCP adherence is satisfactory, and to verify accurate transcription of data to the eCRF.

15 DATA HANDLING AND RECORD KEEPING

15.1 Case Report Forms/Source Data Handling

All required study data must be entered by study site personnel in the eCRF or by study participants in the eDiary created for the study. These data collection tools are a validated EDC system that contains a system generated audit trail. Data required according to this protocol are recorded by study site personnel via data entry into the internet-based EDC software system or by study participant via the eDiary on their personal electronic device (smartphone). The investigator shall ensure that all data from participant visits are promptly entered into the eCRFs in accordance with the specific instructions given. The investigator must sign each eCRF to verify the integrity of the data recorded. All internal ICON and external study site personnel seeking access to the eCRF are supported by a Service Desk (if applicable). At the end of the study all data captured electronically will be provided to the investigator on CD ROM for archiving at the study site.

The investigator must maintain source documents, such as laboratory reports, consultation reports, and complete medical history and physical examination reports. All information in the eCRF must be traceable to the participant's source documents.

The investigator/institution shall provide direct access to source data/documents for study-related monitoring, audits, IEC/IRB review and regulatory inspection.

The Sponsor or designee is responsible for the data management of this study including quality checking of the data.

15.2 Data Protection

Participants will be assigned a unique identifier by the Sponsor. Any participant records or datasets that are transferred to the Sponsor will contain the identifier only; participant names or any information which would make the participant identifiable will not be transferred. The participant must be informed that their personal study-related data will be used by the Sponsor in accordance with local data protection law. The level of disclosure must also be explained to the participant. The participant must be informed that their medical records may be examined by Clinical Quality Assurance auditors or other authorized personnel appointed by the Sponsor, by appropriate IRB/IEC members, and by inspectors from regulatory authorities.

15.3 Dissemination of Clinical Study Data

Regardless of whether the study is completed or terminated prematurely, the Sponsor will ensure that clinical study reports are prepared and provided to regulatory agency(ies) as required by the applicable regulatory requirement(s). The Sponsor will also ensure that clinical study reports in marketing applications meet the standards of the ICH E3: Structure and Content of Clinical Study Reports. Where required by applicable regulatory requirements, an investigator signatory will be identified for the approval of the clinical study report. The investigator will be provided reasonable

access to statistical tables, figures, and relevant reports and will have the opportunity to review complete study results.

15.4 Retention of Essential Documents

The investigator/institution should maintain the study documents as specified in the ICH guidelines on GCP and as required by the applicable regulatory requirements. The investigator/institution should take measures to prevent accidental or premature destruction of these documents.

Essential documents should be retained until at least 2 years after the last approval of a marketing application in an ICH region and until there are no pending or contemplated marketing applications in an ICH region or until at least 2 years have elapsed since the formal discontinuation of clinical development of the trial vaccine or per local regulation, whichever is longer. These documents should be retained for a longer period, however, if required by applicable regulatory requirements or by an agreement with the Sponsor. It is the Sponsor's responsibility to inform the investigator/institution as to when these documents no longer need to be retained.

16 FINANCING AND INSURANCE

The investigator is required to provide financial disclosure information to allow the Sponsor to submit the complete and accurate certification or disclosure statements required under US Title 21 CFR Part 54 and local regulations. In addition, the investigator must provide to the Sponsor a commitment to promptly update this information if any relevant changes occur during the course of the investigation and for 1 year following the completion of the study. Neither the Sponsor nor designee nor the study site is financially responsible for further testing or treatment of any medical condition that may be detected during the screening process. In addition, in the absence of specific arrangements, neither the Sponsor nor designee nor the study site is financially responsible for further treatment of the disease under study.

17 PUBLICATION POLICY

The Sponsor shall retain the ownership of all data. When the study is complete the Sponsor shall arrange the analysis and tabulation of data. A clinical study report shall then be prepared, which may be used for publication, presentation at scientific meetings or submission to regulatory authorities.

The Sponsor will generally support publication of multicenter studies only in their entirety and not as individual study site data. In this case, a coordinating investigator will be designated by mutual agreement. Authorship will be determined by mutual agreement and in line with the International Committee of Medical Journal Editors authorship agreements. Authors will be provided reasonable access to all study data, statistical tables, figures and relevant reports and will have the opportunity to review complete study results. All proposed publications based on this study must be participant to the Sponsor's approval requirements.

The Sponsor assures that the key design elements of this protocol will be posted in a publicly accessible database such as clinicaltrials.gov. In addition, upon study completion and finalization of the study report, the results of this trial will be submitted for publication and/or posted in a publicly accessible database of clinical trial results.

18 CONFLICT OF INTEREST POLICY

The independence of this study from any actual or perceived influence, such as by the pharmaceutical industry, is critical. Therefore, any actual conflict of interest of persons who have a role in the design, conduct, analysis, publication, or any aspect of this study will be disclosed and managed. Furthermore, persons who have a perceived conflict of interest will be required to have such conflicts managed in a way that is appropriate to their participation in the study. The study leadership in conjunction with the Sponsor has established policies and procedures for all study group members to disclose all conflicts of interest and will establish a mechanism for the management of all reported dualities of interest.

19 SIGNATURE OF INVESTIGATOR

PROTOCOL TITLE: A Phase 3, Randomized, Observer-Blinded, Placebo-Controlled Study to Evaluate the Efficacy, Safety, and Immunogenicity of a SARS-CoV-2 Recombinant Spike Protein Nanoparticle Vaccine (SARS-CoV-2 rS) with Matrix-M1™ Adjuvant in Adult Participants ≥ 18 Years with a Pediatric Expansion in Adolescents (12 to < 18 Years)

PROTOCOL NUMBER: 2019nCoV-301

I agree to conduct Protocol 2019nCoV-301 in accordance with the terms and conditions of the protocol, ICH guidelines on GCP and with applicable regulatory requirements. All information pertaining to the study shall be treated in a confidential manner.

((Type name and job title))	Date (day/month/year)

20 REFERENCE LIST

Bengtsson KL, Song H, Stertman L, et al. Matrix-M adjuvant enhances antibody, cellular and protective immune responses of a Zaire Ebola/Makona virus glycoprotein (GP) nanoparticle vaccine in mice. Vaccine. 2016;34(16):1927-35.

Coleman CM, Venkataraman T, Liu YV, et al. MERS-CoV spike nanoparticles protect mice from MERS-CoV infection. Vaccine. 2017;35(12):1586-9.

Department of Health and Human Services (DHHS), Food and Drug Administration, Center for Biologics Evaluation and Research (US). Guidance for industry: Toxicity grading scale for healthy adult and adolescent volunteers enrolled in preventive vaccine clinical trials. September 2007 [cited 2020 Apr 01]. Available from: https://www.fda.gov/media/73679/download

Follmann D, Fintzi J, Fay MP, et al. Assessing durability of vaccine effect following blinded crossover in COVID-19 vaccine efficacy trials. medRxiv 2020.12.14.20248137; doi: https://doi.org/10.1101/2020.12.14.20248137. Preprint.

Guebre-Xabier M, Patel N, Tian JH, et al. NVX-CoV2373 vaccine protects cynomolgus macaque upper and lower airways against SARS-CoV-2 challenge. Vaccine. 2020 Oct 23;38(50):7892-6.

Habibzadeh P, Stoneman EK. The novel coronavirus: a bird's eye view. Int J Occup Environ Med. 2020;11(2):65-71.

Keech C, Albert G, Cho I, et al. Phase 1-2 Trial of a SARS-CoV-2 Recombinant Spike Protein Nanoparticle Vaccine. N Engl J Med. 2020 Sep 2. doi: 10.1056/NEJMoa2026920.

Lambert P-H, Ambrosino DM, Andersen SR, et al. Consensus summary report for CEPI/BC March 12-13, 2020 meeting: Assessment of risk of disease enhancement with COVID-19 vaccines. Vaccine. 2020 Jun 26;38(31):4783-4791.

Liu YV, Massare MJ, Barnard DL, et al. Chimeric severe acute respiratory syndrome coronavirus (SARS-CoV) S glycoprotein and influenza matrix 1 efficiently form virus-like particles (VLPs) that protect mice against challenge with SARS-CoV. Vaccine. 2011;29(38):6606-13. doi:10.1172/jci.insight.123158.

Mandolesi M, Sheward DJ, Hanke L, et al. SARS-CoV-2 protein subunit vaccination elicits potent neutralizing antibody responses. bioRxiv 2020:2020.07.31.228486.

Miettinen OS and Nurminen M. Comparative analysis of two rates. Stat Med. 1985;4(2):213 26.

Su S, Wong G, Shi W, et al. Epidemiology, genetic recombination, and pathogenesis of coronaviruses. Trends Microbiol. 2016;24(6):490-502.

Tian J-H, Patel N, Haupt R, et al. SARS-CoV-2 spike glycoprotein vaccine candidate NVX-CoV2373 elicits immunogenicity in baboons and protection in mice. bioRxiv 2020:2020.06.29.178509.

World Health Organization (WHO). Coronavirus disease 2019 (COVID-19) situation report-116. 15 May 2020. Retrieved 18 May 2020, from https://www.who.int/docs/defaultsource/coronaviruse/situation-reports/20200515-covid-19-sitrep-116.pdf?sfvrsn=8dd60956_2.

Zou G. A modified Poisson regression approach to prospective studies with binary data. Am J Epidemiol. 2004;159(7):702-6.

21 APPENDICES

Appendix 1: Protocol Change History

Protocol Version 9.0, 13 May 2021 (revised from Version 8.0, 16 April 2021)

The following is a summary of the changes made to the protocol.

Location of Change	Change/Modification in Version 9.0
Section 2 (Synopsis)	• Title of study was updated to reflect the change in age range.
Section 3 (Schedule of Assessments); Table 1 Schedule of Assessments in Adult Main Study	• Updated footnote 2; "Minimum Days Following Most Recent Vaccination" refers to initial vaccination 1 (Day 0).<br>• Added a column- Safety Follow-up Call<br>• Updated footnote 8,14,16 and 25<br>• Added footnote 26 – Participants that do no report symptoms in eDiary after blinded crossover until Month 12 should be contacted by sites remotely to collect SAE's, MAAE's or AESI's.<br>• Clarified that participants will be observed for 30 minutes for any immediate reactions on vaccination days.
Section 3 (Schedule of Assessments); Table 2 Schedule of Assessments in the Pediatric Expansion	• Added row; Endpoint Review form<br>• Added phone call columns for Day 14, Day 42 and Day 49 under Initial Vaccination Period.<br>• Added column 6 under Day 49 Initial Vaccination Period<br>• Changed column name – 56 M24<br>• Updated footnote 2; "Minimum Days Following Most Recent Vaccination" refers to initial vaccination 1 (Day 0).<br>• Updated footnote 9 ; 7 scheduled blood draws for immunogenicity assessments will be conducted at Day 0, 21, 35, C1 and Months 12, 18 and 24 visits. Additional blood samples will be collected from participants in CMI cohort to obtain PBMC at Day 0, 7 and 28.<br>• Updated footnote 20; Unscheduled Acute Illness Visit will be contacted by the study site to arrange an Unscheduled Convalescent Visit for participants with confirmed PCR positive test.<br>• Updated footnote 22; updated timeline for study site remote contact.<br>• Updated footnote 8, 14, 15, 18 and 23.<br>• Added footnote 24 clarifying unsolicited AEs, all MAAEs, and MAAEs since last visit will be collected through 49 days after the second injection of the initial and crossover sets of vaccinations.<br>• Changes made under Months following initial vaccinations.
Section 2 (Study Synopsis); Section 7.2.5 (Safety Endpoints)	• Updates made in safety endpoints for Paediatric Expansion.<br>• Made changes to clarify incidence and severity of MAAE's and unsolicited AE's after 49 days ie, 28 days after second injection of each set of vaccinations (initial and crossover).
Section 2 (Study Synopsis); Section 7.1.5.2 (Other Secondary Endpoints)	• Made changes to clarify incidence and severity of MAAE's and unsolicited AE's after 49 days ie, 28 days after second injection of each set of vaccinations (initial and crossover).
Section 2 (Study Synopsis); Section 8.1.1 (Enrollment);	Clarified in Pediatric Expansion<br>• Participants will be randomized in 2:1 ratio,

Section 8.2 (Discussion of Study Design)	• First ~60 adolescent participants after receiving the second dose will be reviewed for early safety data before dosing the remainder of the adolescent participants.<br>• Efforts will be made to enroll similar number of participants in both sub groups 12 to < 15 years of age and 15 to < 18 years of age. In Adult Main Study efforts will be made to enroll diverse population<br>• Safety data will be provided to DSMB after each early safety review period. Simultaneously safety data will be reviewed internally by the sponsor.<br>• Small language changes made in Table 3 and Table 4 footnotes.
Section 8.1.2 (Vaccine Administration)	• Added in Pediatric Expansion unblinded participants will not be eligible to receive further investigational product on this protocol. Participants who withdrew from blinded trial after receiving both doses will not be eligible for blinded crossover. Safety data will be collected remotely.
Section 2 (Study Synopsis); Section 8.1 (Study Design Description)	• Added to Unscheduled Convalescent Visits in Adult Main study, remote contact for safety follow-up may be conducted every 3 months after crossover if no other visits have been required<br>• Text added to the section to clarify ~60 adolescent participants will be reviewed for 7 days for reactogenicity data after receiving second dose, before the second dose is administered to the rest of the adolescent participants.<br>• Review of collection for immunogenicity will be supplemented by remote contacts on Days 7 (+3 days), 14 (+ 3 days), 28 (+3 days), 42 (+3 days) and 49 (+ 7 days) in the initial set of vaccinations<br>• Removed language that frequency of DSMB reviews in the Pediatric Expansion will be presented in the DSMB charter.
Section 2 (Study Synopsis); 8.1.3 (Safety Monitoring)	• Added safety follow up phone calls as a means to collect MAAE's, AESI's and SAE's conducted at 3 and 6 months.<br>• Any AE's, MAAE's and SAE's will be recorded by the parent/ caregiver on the memory aid under paediatric expansion.
Section 2 (Study Synopsis); Section 8.1 (Sample Size and Power); Section 8.8.3.3 (Discontinuation/Withdrawal by Sponsor (Study Halting Rules))	• Any SAE's in the first 60 adolescents within the first 7 days following the first or second initial vaccination.<br>• Any toxicity grade 3 or higher occurring in the first 60 participants within the first 7 days following the first and second initial vaccination.<br>• Any grade 3 (severe) unsolicited single AE occurring in the first 60 participants within the first 7 days following the first or second initial vaccination.
Section 2 (Study Synopsis) ; Section 8.1.4 (Immunogenicity Testing); Section 10.2 (Immunogenicity Assessments)	• Added under pediatric expansion, 7 scheduled blood draws for immunogenicity assessments will be conducted at Day 0, 21, 35, C1 and Months 12, 18 and 24 visits. Additional blood samples will be collected from participants in CMI cohort to obtain PBMC at Day 0, 7 and 28.
Section 2 (Study Synopsis); Section 8.1.5 (Prospective Surveillance of COVID-19); Section 10.1.1 (Active Surveillance for COVID-19)	• Clarified that for Pediatric Expansion, starting from Day 4 throughout the first 12 months of the study, parent(s)/caregiver(s) have to report any COVID-19 symptoms to the site as soon as possible after the onset of the symptoms.<br>• Removed language - No eDiary / FLU-PRO instrument, or self-swabbing will be collected from adolescent participants except for collection of 7-day reactogenicity data after every initial vaccination.<br>• Added text – eDiary will be used for collection of 7 day reactogenicity data after each initial vaccination for adolescent participants. EDiary collection will not be done for crossover vaccinations. Prospective surveillance will be conducted weekly via phone calls.

	• Updated endpoint collection will be obtained using SAE forms or electronic records. In person unscheduled visits will be replaced by remote reporting and testing, if participants are unable to report symptoms for >7 days, study site will contact to remind them.
Section 2 (Study Synopsis) ; 8.1.7 (Periods of Pediatric Expansion)	• Clarified that adolescent participants will be randomized in 2:1 ratio to receive 5 µg SARS-CoV-2 rS adjuvanted with 50 µg Matrix-M1 or placebo. Made small corrections in the language.<br>• Added text to clarify blinded cross over will begin after the last vaccination in the initial set of vaccinations.
Section 2 (Study Synopsis); Section 8.1 (Study Design Description); Section 12.5 (Safety Monitoring)	• Text added to the section to clarify 60 adolescent participants will be reviewed by the DSMB for 7 days for reactogenicity and safety data after receiving second dose, before the second dose is administered to the rest of the adolescent participants.<br>• First 60 adolescent participants will be enrolled in an approximately even distribution across age groups 15 to < 18 and 12 to < 15 years of age.
Section 2 (Study Synopsis) ; Section 8.1.9 (Safety Monitoring); Section 12.5 (Safety Monitoring)	• Added under Adult Main Study and Pediatric Expansion, centralized DSMB in collaboration with NIH, NIAID, BARDA and Novavax will be established to review interim unblinded data periodically and make recommendations with respect to safety and efficacy.;<br>• Short-term safety cohort will be reviewed by DSMB and internal sponsor simultaneously. If any subgroup manifests widespread AE's DMSB may recommend to terminate the trial or withdraw the specific group from the study.
Section 8.6 (Strategies for recruitment and retention)	• In Adult Main Study and Pediatric Expansion efforts will be made to enroll diverse population including minorities
Section 2 (Study Synopsis); Section 12.3.1 (Adult Main Study and Pediatric Expansion)	• Updated language to include subsequent analysis of data from pediatric expansion.
Section 2 (Study Synopsis); Section 12.6 (Data Safety and Monitoring Board)	• Clarified unblinded data will be reviewed regularly. Monitoring of possible harm or any other safety issue will be overseen by the sponsor in collaboration with DSMB.
Section 8.1.5 (Prospective Surveillance of COVID-19); Table 5 (Symptoms Suggestive of COVID-19)	• Updated suggestive symptoms of COVID-19
Section 8.1.11 (Participant Retention)	• Removed language regarding unscheduled visits and participant follow up.
Section 9.1 (Administration of study treatment)	• Updated text to clarify participant should receive originally randomized material if participant received wrong study material must be unblinded to offer a third dose ensuring they receive at least two doses of NVX-CoV2373
Section 10.3.2 (Time Period and Frequency of Collecting AE and SAE Information)	• Added text clarifying MAAE's will be collected from the time of first study vaccination through until 49 days (28 days after the second injection of each set of vaccinations [(initial and crossover]).
Section 10.3.8 (Physical Examination)	• Added text to clarify targeted physical examination after Day 21 is optional AE evaluation is mandatory. System directed physical examination will also include vital signs and O2 saturation at the Acute Illness Visit
Section 2 (Study Synopsis); Section 12.1 (Sample Size & Power)	Clarifications under Pediatric Expansion<br>• Efforts will be made to recruit similar number of adolescent participants in both age groups.

	• Analysis of efficacy in Pediatric Expansion is descriptive in nature, using same methods as the Adult Main Study with no formal statistical hypothesis tested.<br>• Added one more pre-specified criteria under NI analysis.<br>• 400 evaluable participants selected from each of the 18 to < 26 year old subset of participants in the Adult Main Study and the Pediatric Expansion, to demonstrate the first two non-inferiority criteria when assuming an underlying GMT, with definition of seroresponse as 4-fold rise in neutralization titers at Day 35 relative to baseline and assumed seroresponse rates (SRR) of 95%.
Section 2 (Study Synopsis); Section 12.2.1 (Primary Endpoint)	• Added text to clarify The analysis of the two pre-specified NI success criteria for ratio of GMTs will be performed using the point estimate and upper bound of the two-sided 95% Confidence Interval (CI) on the ratio of GMTs between 2 age cohorts
Section 2 (Study Synopsis); Section 12.2.2.1 (Adult Main Study and Pediatric Expansion)	• Added point to clarify for moderate to severe efficacy endpoint, cases will be reviewed and confirmed by an external endpoint committee in a blinded manner. Details of this endpoint will be in Statistical Analysis Plan.<br>• Changed method of calculation from SCR to SRR with 95% CI for serum antibody levels specific for the SARS-CoV-2 S protein antigen<br>• Evaluation of immune responses will also assess the 12 to < 15 and 15 to < 18 years age groups of the Pediatric Expansion.
Section 2 (Study Synopsis); Section 8.1 (Study Design Description); Section 12.6 (Data Safety and Monitoring Board)	• Removed language that frequency of DSMB reviews in the Pediatric Expansion will be presented in the DSMB charter.
General changes	• For clarity: changed 12 to 17 years to 12 to < 18 years; changed 15 to 17 years to 15 to < 18 years; changed 12 to 14 years to 12 to < 15 years; changed 18 to 25 to 18 to < 26; and changed 18-64 years to 18 to ≤ 64 years.<br>• Removed diary card as a means to collect data.<br>• Typographical errors were corrected without tracked changes.

Protocol Version 8.0, 16 April 2021 (revised from Version 7.0, 31 March 2021)

The following is a summary of the changes made to the protocol.

Location of Change	Change/Modification in Version 8.0
Section 2 (Synopsis)	• Title of study was updated<br>• Adult Main Study Updated to Main Study<br>• Under Main Study-<br>Changes and updates to key secondary objectives and endpoints, other secondary objectives and endpoints, and exploratory objectives and endpoints.<br>• Under Pediatric Expansion –<br>Changes and updates added to primary objectives and endpoints, secondary objectives and endpoints, and exploratory objectives and endpoints.
Section 3 (Schedule of Assessments); Table 2 Schedule of Assessments in the Pediatric Expansion	• Monthly Phone Call column updated to Regular Remote Contact.<br>• Added two rows-Monitoring for COVID-19 illness and Nasal swab (s) at clinic-anterior nares.

Section 7 (Study Objectives, endpoints and estimands); Section 7.1 (Main Study)	• Title of the Section updated to Main Study
Section 7 (Study Objectives, endpoints and estimands); Section 7.1.2.1 (Key Secondary Objective)	• Added key secondary objective for the Adult Main Study. To clarify SARA-CoV-2 not considered as a variant of concern or interest according to the CDC variant Classifications
Section 7 (Study Objectives, Endpoints and Estimands); Section 7.1.2.2 (Other Secondary Objectives)	• Added other secondary objectives for the Adult Main study
Section 7 (Study Objectives, endpoints and estimands); Section 7.1.3 (Exploratory Objective)	• Added exploratory objectives for the Adult Main Study. To clarify SARS-CoV-2 not considered as a variant of concern or interest according to the CDC variant Classifications.
Section 7 (Study Objectives, endpoints and estimands); Section 7.1.5.2 (Key Secondary Endpoint)	• Added key secondary endpoints for the Adult Main Study. To clarify SARS-CoV-2 not considered as a variant of concern or interest according to the CDC variant Classifications.
Section 7 (Study Objectives, endpoints and estimands); Section 7.1.6 (Other Secondary Endpoint)	• Added other secondary endpoints for the Adult Main Study.
Section 7 (Study Objectives, endpoints and estimands); Section 7.1.6 (Exploratory Endpoint)	• Added exploratory endpoints for the Adult Main Study.
Section 7 (Study Objectives, endpoints and estimands); Section 7.2.1 (Primary Objective)	• Added primary objectives for the Pediatric Expansion. To evaluate the efficacy of the 2-dose regimen, after completion of the second injection in the initial set of vaccinations.
Section 7 (Study Objectives, endpoints and estimands); Section 7.2.2 (Secondary Objective)	• Added secondary objectives for the Pediatric Expansion<br>• To evaluate the efficacy of the 2-dose regimen, after completion of the second injection in the initial set of vaccinations.<br>• To assess vaccine efficacy (VE) against ANY symptomatic SARS-CoV-2 infection<br>• To assess the durability of vaccine efficacy (measured by all defined efficacy endpoints) in adolescents after initial active vaccine recipients versus crossover (delayed) active vaccine recipients.
Section 7 (Study Objectives, endpoints and estimands); Section 7.2.3 (Exploratory Objective)	• Added secondary objectives for the Pediatric Expansion<br>• To evaluate the efficacy of the 2-dose regimen, after completion of the second injection in the initial set of vaccinations.<br>• To assess impact of vaccination on nasal viral load in nasal swabs of participants who develop symptoms of possible COVID-19.<br>• To assess impact of vaccination on asymptomatic SARS-CoV-2 PCR positivity and viral load at the time of the crossover set of vaccination.<br>• To describe sequences of the genetic material from SARS-CoV-2 viruses detected in COVID-19 cases to evaluate possible viral mutations that may be associated with breakthrough infections.

Section 7 (Study Objectives, endpoints and estimands); Section 7.2.4 (Primary Endpoints)	• Added primary endpoints for the Pediatric Expansion.<br>• First Episode of PCR-positive mild, moderate, or severe COVID-19 where severity is defined.<br>• Admission to an intensive care unit (ICU).<br>• Death<br>• Age group of Seronegative adult participants changed from 18-<65 to 18-25 years.<br>• Divided Endpoints into safety endpoints and effectiveness endpoints.
Section 7 (Study Objectives, endpoints and estimands); Section 7.2.5 (Secondary Endpoints)	• Added secondary endpoints for the Pediatric Expansion<br>• First episode of PCR-positive COVID-19, as defined under the primary endpoint, shown by gene sequencing to represent a variant not considered as a "variant of concern / interest" according to the CDC Variants Classification.<br>• First episode of PCR-positive moderate or severe COVID-19, as defined under the primary endpoint.<br>• ANY symptomatic SARS-CoV-2 infection, defined as: PCR- positive nasal swab and ≥ 1 of different symptoms<br>• Other Small changes made to existing secondary endpoints.
Section 7 (Study Objectives, endpoints and estimands); Section 7.2.6 (Exploratory Endpoints)	• Added exploratory endpoints for the Pediatric Expansion<br>• First episode of PCR- positive COVID-19 as defined under the primary endpoint, shown by gene sequencing to represent a "variant of concern / interest" according to the CDC Variants Classification.<br>• Quantitative PCR tests may be performed on nasal swabs collected from this trial to assess whether vaccination impacts viral shedding.<br>• Quantitative PCR tests performed on nasal swabs collected immediately prior to administration of blinded crossover vaccination to assess impact of initial vaccination on frequency of asymptomatic SARS-CoV-2 infection and level of viral shedding.<br>• Next generation sequencing of viral genomes detected in nasal swabs tested by PCR to describe the genetic evolution of circulating SARS-CoV-2 strains during the conduct of the study.
Section 8 (Overall Study Design and Plan); Section 8.1 (Study Design Description)	• Under Pediatric Expansion, number of adolescent participants enrolled updated from 30 to 60 (N=2940).<br>• Under Adult main study and Pediatric Expansion, Safety assessments will be collected via eDiary.<br>• Under Pediatric Expansion, updates added for weekly remote contact, In - person Unscheduled Acute Illness Visit and Unscheduled Convalescent Visit.<br>• Number of Adolescents to be enrolled = 60 in two age groups (15-17 years and 12-15 years), they will be enrolled first. and will followed for 7 days after the first vaccine dose for assessment of reactogenicity, which will be reviewed prior to enrollment of the remaining 2,940 adolescents.
Section 8 (Overall Study Design and Plan); Section 8.1 (Study Design Description)	• Under Pediatric Expansion age of pediatric participants changed to 12 to 17 years of age.<br>• Under pediatric expansion parents/caregivers will be asked to report symptoms of COVID-19 to the site on the onset of symptoms.<br>• Updated details for Unscheduled Acute Illness visits and Unscheduled Convalescent Visit and their conditions and evaluations.<br>• Number of adolescent patients to be enrolled initially and reviewed changed.<br>• Added Study Vaccination Pause Rules for Pediatric Expansion.
Section 10 (Study Assessments and	• Added under Pediatric Expansion, once all participants have had 1 year of follow-up from enrollment, an analysis will be executed to examine durability

procedures); Section 10.5 (Post Blinded Crossover)	of the vaccine on efficacy endpoints, taking into account the timing of the blinded crossover as described in Follmann et al, 2020.
Section 12 (Statistical Considerations); Section 12.1 (Sample Size and Power)	• Updated details of Table: Estimated Vaccine Efficacy and 95% CI for Various Scenarios of Endpoints Observed<br>• Analyses of efficacy in the Pediatric Expansion will use the same methods as the Adult Main Study.
Section 12 (Statistical Considerations); Section 12.3.1 (Primary Endpoint)	• Added under Pediatric Expansion Primary Endpoint –<br>• In the **Pediatric Expansion**, the primary endpoint will be analyzed on the PP-EFF analysis set and supported by analysis of the FAS analysis set. The statistical methods will be identical to those used for Adult Main Study.
Section 12 (Statistical Considerations); Section 12.4 (Interim Analysis)	• Clarified no analysis planned that require adjustment to type I error.<br>• Before EoS, only primary and secondary efficacy endpoints will be included in Adult Main Study.<br>• In Pediatric Expansion after month 12 durability will be examined based on efficacy endpoints.
Section 19 (Signature of Investigator)	• Changes made in Title

Abbreviations: PCR = polymerase chain reaction; PP-IMM = Per-Protocol Immunogenicity; SARS-CoV-2 rS = severe acute respiratory syndrome coronavirus 2 recombinant spike protein nanoparticle vaccine; CI= confidence interval.

Protocol Version 7.0, 31 March 2021 (revised from Version 6.0, 02 March 2021)

The following is a summary of the changes made to the protocol.

Location of Change	Change/Modification in Version 7.0
Section 1 (General Information)	• Added National Coordinating Investigator for Mexico.
Section 2 (Study Synopsis); Safety Assessments; Section 8.1 (Study Design Description)	• Updates were made in safety assessments for both the Adult Main Study and Paediatric Expansion, during the second 12 months of follow up after the initial set of vaccinations instead of monthly participants will be queried every 3 months via remote contact or thru scheduled visits.
Section 2 (Study Synopsis); Immunogenicity Assessments; Section 3 (Table 2 – Footnote 16); Section 8.1 (Study Design Description); Section 10.2 (Immunogenicity Assessments)	• Clarified that in the Pediatric Expansion, whole blood samples for PBMC will be collected at Days 0, 7 and 28 from a small subset comprised of 50 adolescent participants (not the first 50 participants) enrolled at selected study site(s) with the capacity to isolate PBMCs.
Section 2 (Study Synopsis); Prospective Surveillance of COVID-19 (Adult Main Study); Section 8.1 (Study Design Description); Section 10.1 (Efficacy Assessments)	• Clarified that passive surveillance of safety and efficacy would be made by remote contacts or at the scheduled visits during Months 12 to 24.<br>• Added respiratory rate to the targeted physical examination at the in-person Unscheduled Acute Illness Visit.
Section 2 (Study Synopsis); Timing of Trial Visits; Section 3 (Table 1 –	• Text added to the section to clarify participants who requested to be unblinded/or are continuing in the study for Safety Follow-up will have visits replaced by remote contact (phone, email, text) every 90 days for first year of

footnote 25); Section 8.1 (Study Design Description)	participation and every 180 days for second year of participation. No eDiary, nasal swabs or blood samples will be collected.
Section 2 (Participant Retention); Section 8.1 (Study Design Description)	• Clarified that safety follow-up after blinded crossover continued at scheduled visits for participants who did not request unblinding or at regular intervals if visits were not mandated.
Section 2 (Statistical Methods and Sample Size Calculation); Section 12.1 (Sample Size and Power)	• The conduct of two formal interim analyses of efficacy and futility were replaced with a single analysis of efficacy.
Section 2 (Per-Protocol Immunogenicity Analysis Set); Section 12.2.5	• Added that the The PP-IMM analysis set will also exclude participants who have a PCR-positive nasal swab between baseline up to the visit analyzed.
Section 2 (Primary Endpoint); Section 12.3.1	• Replaced alpha level adjusted confidence interval to 95% confidence interval.<br>• Replaced alpha level adjusted hypothesis test to a hypothesis test with a one-sided Type I error of 2.5%.<br>• Replaced two-sided alpha adjusted confidence bound with a two-sided 95% confidence interval.
Section 2 (Other Secondary Endpoints); Section 7.1.5.2	• Clarified 300 participants 18-64 years of age in the IgG analysis were active vaccine recipients.
Section 2 (Pediatric Expansion – Effectiveness); Section 12.3.1 (Primary Endpoint)	• Clarified that the non-inferiority analysis of the primary effectiveness endpoint included a comparison with the 18-64 year old immunogenicity cohort from the Adult Main Study.
Section 2 (Secondary Endpoints – Adult Main Study); Section 12.3.2; Section 12.3.3 (Statistical Models)	• Replaced one-sided alpha of 0.05 to 0.025.<br>• Clarified that the bridging analysis was an immunobridging analysis comparing the initial lot (from the initial set of vaccinations) to the crossover lot.
Section 2 (Statistical Analyses); Section 12.3	• New section added describing the unblinding of study site and Sponsor personnel at the time of the primary and secondary endpoint analyses.
Section 2 (Interim Analyses); Section 12.4	• Stated that no formal interim analyses were planned, which resulted in the removal of text from this section.
Section 2 (Post Blinded Crossover); Section 10.5	• Clarified that follow-up during Months 12 to 24 will continue via remote contacts every 3 months through study completion, except when a visit is scheduled and replaces the remote contact.
Section 2 (Data and Safety Monitoring Board); Section 12.7	• Removed language that the DSMB will monitor the study for high vaccine efficacy or for futility to detect vaccine activity.
Section 3 (Table 1)	• Added respiratory rate to footnote 15.<br>• Clarified footnote 24 to remove monthly remote contacts with remote contacts at Months 15 and 21.<br>• Clarified that no remote contacts were needed when in-person visits were scheduled.
Section 3 (Table 2)	• Clarified that from Months 3 to 12, study sites will perform monthly remote contacts with the participants' parent(s)/caregiver(s) to collect safety assessments and every 3 months from Months 12 to 24.
Section 9.6 (Procedure for Breaking the Randomization Code)	• Removed language pertaining to the planned interim analyses.

Section 12.1 (Sample size and Power)	• Replaced Table 7, which originally described the power for the planned interim and final analyses, with a table describing the power of a single efficacy analysis.<br>• Added Table 8 describing the example statistical success boundaries for the single efficacy analysis.<br>• Removed language describing an interim and final analysis.<br>• Clarified that the lot bridging subset analysis was an immunobridging study comparing IgG levels (in approximately 900 participants 18 to 64 years of age at select sites) between approximately 300 vaccine (received placebo initially) and 600 placebo (received active vaccine initially) recipients from the crossover (delayed) phase, where the 300 vaccine recipients receive a dose of vaccine from a different lot than received by the 600 placebo recipients who received active vaccine during the initial set of vaccinations. The IgG antibody concentrations induced by the initial set of vaccinations for the 600 placebo recipients will be used in the evaluation, while the IgG antibody concentrations induced by the crossover set of vaccinations will be used for the 300 vaccine recipients.<br>• Revised standard deviation of log10 neutralization antibody titer calculation estimate from 0.60 to 0.42.
Section 12.3.3 (Statistical Models)	• Added the following text: In the case where there are zero endpoints for one of the vaccine groups, the Poisson model will be substituted with an exact method to be detailed in the Statistical Analysis Plan.
General changes	• Language pertaining to interim, final, or futility analyses were removed from the protocol as only a single efficacy analysis will be performed in the study.<br>• Typographical errors were corrected without tracked changes.

Abbreviations: PCR = polymerase chain reaction; PP-IMM = Per-Protocol Immunogenicity; SARS-CoV-2 rS = severe acute respiratory syndrome coronavirus 2 recombinant spike protein nanoparticle vaccine; CI= confidence interval.

Protocol Version 6.0, 02 March 2021 (revised from Version 5.0, 02 February 2021)

The following is a summary of the changes made to the protocol.

Location of Change	Change/Modification in Version 6.0
Title Page; Sponsor Signature Page; General Information; Section 2 (Synopsis –Title of Study); Section 19 (Signature of Investigator)	• Title revised to add with Pediatric Expansion in Adolescents (12 to <18 Years) and School Age Children (6 to 11 Years)
Section 2 (Synopsis –Study Centers)	• Increased number of study centers to 160
Section 2 (Synopsis – Planned Study Period)	• Changed the study end period to 2023
Section 2 (Synopsis – Adult Main Study–Primary Endpoint); Section 7.1.4 (primary endpoint)	• Primary endpoint was revised for clarity to first episode of PCR-positive mild, moderate or severe COVID-19.
Section 2 (Synopsis – Adult Main Study–Key Secondary Objective);	• Revised key secondary objective by removing the text related to the level of severity of the PCR-confirmed symptomatic COVID-19 illness and to further

Section 7.1.2.1 (Key secondary objective)	clarify that SARS-CoV-2 variant, which is the cause of COVID-19 condition has to be genetically similar (ie, "matched") to that contained in the vaccine.
Section 2 (Synopsis – Adult Main Study–Key Secondary Endpoints); Section 7.1.5.1 –Key Secondary Endpoints)	• The term "nasal swab" was removed to align with the primary endpoint.<br>• The key secondary endpoint was re-phrased to align with the re-defined primary endpoint and key secondary objective.
Section 2 (Synopsis – Adult Main Study–Secondary Objectives); Section 7.1.2.2 (secondary objective)	• Section heading was updated to add "Other" Secondary Objectives<br>• Added to secondary objective to evaluate the efficacy of a 2-dose regimen of SARS-CoV-2 rS adjuvanted with Matrix-M1 compared to placebo against PCR-confirmed moderate-to-severely symptomatic COVID-19 illness diagnosed ≥ 7 days after completion of the second vaccination in the initial set of vaccinations of adult participants ≥ 18 years of age.<br>• Corrected SARS-CoV NP to SARS-CoV-2 NP
Section 2 (Synopsis – Adult Main Study–Other Secondary Endpoints); Section 7.1.5.2 – Other Secondary Endpoints)	• Text added to describe the First episode of PCR-positive moderate or severe COVID-19, as defined under the primary endpoint.
Section 2 (Synopsis – Adult Main Study–Exploratory Objectives); Section 7.1.3 (Key Exploratory Objectives)	• Added to exploratory objectives to evaluate the efficacy of study vaccine compared to placebo against PCR-confirmed symptomatic COVID-19 illness due to SARS-CoV-2 variant genetically divergent compared to that contained in the vaccine (ie, "unmatched" genetic variants), diagnosed ≥ 7 days after Completion of the second vaccination in the initial set of vaccinations of adult participants ≥ 18 years of age.
Section 2 (Synopsis – Adult Main Study–Exploratory Endpoints); Section 7.1.6 – Exploratory Endpoints)	• Exploratory endpoints were aligned with the re-defined primary endpoint to add first episode of PCR-positive COVID-19, as defined under the primary endpoint, shown by gene sequencing to be divergent to the vaccine antigen (ie, "unmatched" genetic variants).<br>• Revised for clarity text related to the future assessment of the next-generation sequencing of viral genomes.
Section 2 (Synopsis – Pediatric Expansion – Objectives); 7.2 Pediatric Expansion –7.2.1 Primary Objectives)	• Headings and sub-heading were added for the Pediatric Expansion
Section 2 (Synopsis – Pediatric Expansion – 7.2.1 Primary Objectives)	• Added primary objectives for the pediatric expansion.
Section 2 (Synopsis – Pediatric Expansion – 7.2.4 Primary Endpoints)	• Added primary endpoints for the pediatric expansion.
Section 2 (Synopsis – Pediatric Expansion – 7.2.2 Secondary Objectives)	• Added secondary objectives for the pediatric expansion.
Section 2 (Synopsis – Pediatric Expansion – 7.2.5 Secondary Endpoints)	• Added secondary endpoints for the pediatric expansion.
Section 2 (Synopsis – Pediatric Expansion 7.2.3 Exploratory Objective)	• Added exploratory objectives for the pediatric expansion.

Section 2 (Synopsis – Pediatric Expansion – 7.2.6 Exploratory Endpoints)	• Added exploratory endpoints for the pediatric expansion.
Section 2 (Synopsis –Study Design); 8.1Study Design Description	• Updated to include specifications related to Adult Main Study and Pediatric Expansion.
Section 2 (Synopsis – Study Design)	• New sub-headings added under study design- Enrollment; Trial Vaccinations; Safety Assessments; Immunogenicity Assessments; Prospective Surveillance of COVID-19 (Adult Main Study only); Timing of Trial Visits; Safety Monitoring; and Participant Retention
Section 2 (Synopsis – Enrollment); 8.1 (Study Design– Enrollment)	• Aligned with the objectives and endpoints, to update enrollment for both Adult Main Study and Pediatric Expansion, as applicable.<br>• Title of Table 3 was added to include Adult Main Study in the Section 8.1 (Study Design– Enrollment)
Section 2 (Synopsis – Trial Vaccinations) 8.1 (Study Design– Trial Vaccinations)	• Aligned with the objectives and endpoints to include information regarding trial vaccinations of both Adult Main Study and Pediatric Expansion separately.<br>• Vaccine administration site-related text of alternating deltoid muscles were removed.
Section 2 (Synopsis – Safety Assessments); 8.1 (Study Design– Safety Assessments)	• Aligned with the objectives and endpoints to include information regarding safety assessments of both Adult Main Study and Pediatric Expansion separately.
Section 2 (Synopsis – Immunogenicity Assessments); 8.1 (Study Design– Immunogenicity Assessments)	• Text aligned with the objectives and endpoints to incorporate immunogenicity assessments applicable for Adult Main Study and Pediatric Expansion.
Section 2 (Synopsis – Prospective Surveillance of COVID-19 (Adult Main Study only); 8.1 (Study Design– Prospective Surveillance of COVID-19 (Adult Main Study only)	• Prospective surveillance of COVID-19 was added to clarify that it will only be conducted in the Adult Main Study. For the Pediatric Expansion, COVID-19 diagnoses will be collected as an important medical event reported as an SAE, regardless of severity.
Section 2 (Synopsis – Timing of Trial Visits); 8.1 (Study Design– Timing of Trial Visits)	• Regarding the Adult Main Study, the text was updated to clarify that additional subsequent study visits for blood draws and the crossover set of vaccinations (approximately 21 days apart) will occur after acquisition of safety and efficacy data on SARS-CoV-2 rS with Matrix-M1 adjuvant sufficient to support application for Emergency Use Authorization (EUA) in adults. The Pediatric Expansion visits were added
Section 2 (Synopsis – Safety Monitoring); 8.1 (Study Design– Safety Monitoring)	• Safety monitoring of the protocol was updated Novavax Clinical Development Leads and a parenthetical note was added to the 2019nCoV-301 Protocol Safety Review Team (PSRT) clarifying that it is related to the Adult Main Study only.<br>• More information was added regarding the short-term safety assessment of the pediatric cohorts.
Section 2 (Synopsis – Participant Retention); 8.1 (Study Design– Participant Retention)	• Aligned with the objectives and endpoints to revise as per both the age groups in the respective studies.<br>• Text revised to clarify eligibility of participants to receive further vaccination if unblinded depending upon when the unblinding occur.

	• Text revised to clarify that participants in the Pediatric Expansion who are unblinded will not be eligible to received subsequent vaccinations or for the blinded crossover.
Section 2 (Synopsis – Number of Participants)	• Text aligned with the objectives and endpoints to include both Adult Main Study and Pediatric Expansion.<br>• The Pediatric Expansion incorporated a plan to enroll up to approximately 3,000 adolescent participants 12 to < 18 years of age and approximately 3,000 school-age participants 6 to 11 years of age who will be randomized initially 2:1 to receive active vaccine or placebo.
Section 2 (Synopsis – Inclusion Criteria); 8.3.1 (Inclusion Criteria)	• Text aligned with the objectives and endpoints to include inclusion criteria for both Adult Main Study and Pediatric Expansion.
Section 2 (Synopsis – Exclusion Criteria); 8.3.2 (Inclusion Criteria)	• Text aligned with the objectives and endpoints to include exclusion criteria for both Adult Main Study and Pediatric Expansion.
Section 2 (Synopsis –Test Product, Dose and Mode of Administration)	• Text related to vaccine administration to the deltoid muscles was removed.
Section 2 (Synopsis – Statistical Methods and Sample Size Calculation)	• Text aligned with the objectives and endpoints to include statistical methods and sample size calculation respective to both Adult Main Study and Pediatric Expansion.<br>• The added text described the sample size in the Pediatric Expansion.
Section 2 (Synopsis – Analysis Sets); 12.2.4 (Per-Protocol Efficacy [PP-EFF] Analysis Set)	• Add second per-protocol efficacy 2 (PP-EFF-2) analysis set, which included all participants regardless of baseline serostatus.
Section 2 (Synopsis – Primary Endpoint);	• Sub-heading added- Adult Main Study and Pediatric Expansion.<br>• Safety was added as another sub-heading to Pediatric Expansion, to further include information regarding the process to assess safety in this cohort.<br>• Another sub-heading was added to describe how Effectiveness will be assessed.
Section 2 (Synopsis – Secondary Endpoints)	• Sub-heading added- Adult Main Study and Pediatric Expansion.<br>• Under Pediatric Expansion, information was added, which clarified that descriptive analysis without a pre-specified NI margin will be carried out for the serum antibody levels specific for the SARS-CoV-2 S protein antigen(s) (IgG antibody to SARS-CoV-2 S protein and hACE2 inhibition) at Day 35 using the same statistical method as the primary endpoint. The secondary immunogenicity analyses will be performed using the PP-IMM analysis set and the FAS.
Section 2 (Synopsis – Safety Analyses); 12.3.5 (Safety Analysis)	• Sub-heading added- Adult Main Study and Pediatric Expansion. The text was updated as applicable.<br>• Removed reference related to PSRT review of accumulating safety data because the accumulating safety data will be reviewed by the ICON Medical Monitor.
Section 2 (Synopsis – Interim Analyses)	• Sub-heading added- Adult Main Study and updated accordingly.
Section 2 (Synopsis – Monitoring Potential Vaccine Harm)	• Sub-heading added- Adult Main Study
Section 2 (Synopsis – Post Blinded Crossover)	• Sub-heading added- Adult Main Study and text added to clarify that follow-up will be continued to be collected by remote contact.

	• Sub-heading added- Expansion. The text was added to clarify that following blinded crossover, follow-up with monthly remote contacts will continue to collect safety and COVID-19 diagnoses after crossover.
Section 2 (Synopsis – Data and Safety Monitoring Board)	• The text was updated to include Adult Main Study and Pediatric Expansion, wherever applicable.<br>• Information regarding the frequency of DSMB reviews was added in the Pediatric Expansion to determine and present in the DSMB Charter.
Section 2 (Synopsis – Date of the Protocol)	• The date of the protocol was updated to 02 March 2021.
Section 3 (Schedule of Assessments)	• Added information on the contents of Table 1 and Table 2 of the schedule of assessments.<br>• Revised the title of Table 1 to include Adult Main Study.<br>• Added Table 2 - Schedule of Assessments in the Pediatric Expansion.
Section 4.1 (List of Tables)	• Table numbers were updated.
Section 6.4 (Study Rationale)	• Study rationale was aligned with the changes made for the purpose of the study (Section 6.1) to clarify that adult participants ≥ 18 years of age were included in the main study.<br>• Likewise, the Pediatric Expansion was added to evaluate the safety and effectiveness of SARS-CoV-2 rS with Matrix-M1 adjuvant in adolescents (12 to < 18 years) and school-age children (6 to 11 years).
Section 8.1 (Study Design Description)	• Added Table 4 describing the trial vaccine groups for the Pediatric Expansion.
Section 8.2 (Discussion of Study Design)	• Removed statement regarding the consideration to be paid to enroll individuals at increased risk of COVID-19 by virtue of interpersonal interactions.
Section 8.4 (Prohibited Medications)	• Added that any vaccine received within 4 days prior to the first study vaccination or planned receipt of any vaccine before Day 49 (ie, 28 days after second vaccination).<br>• Updated text regarding seasonal flu vaccination, which may be received ≥ 14 days prior to or ≥ 7 days after either study vaccination.
Section 9.6 (Procedure for Breaking the Randomization Code)	• Removed text related the blinded trial, and remaining text updated as applicable for the Adult Main Study.<br>• Added that Participants in the Pediatric Expansion who are unblinded between the first (Day 0) and second (Day 21) dose of trial vaccine will not be eligible to receive further investigational product on this protocol. Similarly, participants who choose to withdraw from the blinded trial after having received both doses of blinded study vaccine will not be eligible for blinded crossover.
Section 10 (Study Assessments and procedures)	• As the study also includes a Pediatric Expansion, so applicable information regarding assent form was updated.
Section 10.1 (Efficacy Assessments)	• Parenthetical note was added to the title- Adult Main Study Only. Underlying text updated accordingly.
Section 10.2 (Immunogenicity Assessments)	• Added that in the Pediatric Expansion, blood samples for serologic assessments (anti-NP antibodies, IgG antibody to SARS-CoV-2 S protein, MN, and hACE2 inhibition) will be collected from all pediatric participants before the initial set of vaccinations and immediately prior to the crossover set of vaccinations and how PBMC samples will be collected.

Section 10.3.1.3 (Serious Adverse Events)	• Added that in the Pediatric Expansion, all COVID-19 diagnoses will be reported as SAEs, regardless of severity.
Section 10.5 (Post Blinded Crossover)	• Section updated to incorporate information as per both Adult Main Study and Pediatric Expansion, as applicable.<br>• Added that in the Pediatric Expansion, following blinded crossover, follow-up with monthly remote contacts will continue to collect safety and COVID-19 diagnoses after crossover.
Section 11 (Medical Resource Utilization)	• Text was updated as per Adult Main Study.
Section 12.1 (Sample Size and Power)	• Text was updated as per Adult Main Study.<br>• Further clarified that the analyses for the Adult Main Study (including interim analyses and final analysis) will be performed separately from analyses for the Pediatric Expansion. Only participants > 18 years of age will be included for analyses of data collected in the Adult Main Study.<br>• Clarified that the random selection of 600 participants per age cohort may be carried out in multiple subsets throughout the study and may also include other stratification factors to achieve analytical efficacy.<br>• Table 7 – Values of the new lot was updated to 270 and Power to Show Equivalence of the 2 Manufacturing Lot Processes was changed to >99%.<br>• Added that the sample size for the Pediatric Expansion is chosen to provide an adequate safety database of ≥3,000 pediatric recipients of investigational product to support licensure of the SARS-CoV-2 rS Matrix-adjuvanted vaccine in pediatric participants 6-17 years of age.<br>• Added probability of observing AEs in the pediatric population with the proposed samples size.<br>• Added the process in which non-inferiority analysis will be performed between the 2 age cohorts in the Pediatric Expansion of the study and the 18-64 years old adult population in the Adult Main Study.
Section 12.3.1 (Primary Endpoint)	• Updated as applicable for the Adult Main Study and Pediatric Expansion.<br>• Added that in the Pediatric Expansion, details on the formal non-inferiority (NI) analysis of the primary effectiveness endpoint.
Section 12.3.2 (Secondary Endpoint)	• Updated as applicable for the Adult Main Study and Pediatric Expansion.<br>• Added that in the Pediatric Expansion, descriptive analysis without a pre-specified NI margin.
Section 12.3.3 (Statistical Models)	• Statistical models were used to analyze efficacy endpoint in the Adult Main Study.
Section 12.3.5 (Safety Analysis)	• Added that, participants who choose to be unblinded prior to the blinded crossover and receive EUA-authorized vaccine will be followed for safety (SAE, MAAE, AESI and COVID-19 diagnosis) by remote contact on the remaining schedule as specified in Table 1 and Table 2.
Section 12.4 (Interim Analysis)	• Following parenthetical note was added to the 2 age strata- 18-64 years of age, ≥65 years of age.<br>• Statement regarding the UBCI for the secondary endpoint was removed.
Section 12.5 (Monitoring Potential Vaccine Harm)	• Clarified by adding text- in the Adult Main Study.
Section 12.6 (Safety Monitoring)	• Novavax Clinical Development Leads was added to the other safety monitoring committees.<br>• 2019nCoV-301 PSRT routinely monitors only for Adult Main Study.

	• Added description of the age de-escalation process and assessment of reactogenicity/safety.
Section 12.7 (Data Safety and Monitoring Board)	• Added that the frequency of DSMB reviews of Pediatric Expansion data is to be determined and presented in the DSMB Charter.<br>• The statement regarding ICON unblinded statistician immediately informing the DSMB if the pre-specified stopping boundary meets is specific to the Adult Main Study and is updated accordingly.
Section 13.2 (Documentation of Informed Consent)	• Added documentation of assent forms for the Pediatric Expansion.
Section 21 (Appendix 1)	• Added that Protocol Version 6.0, 02 March 2021 (revised from Version 5.0, 02 February 2021<br>• Added that the following is a summary of the changes made to the protocol.
General Changes	• Revised text for consistency across the protocol.<br>• Text was revised wherever applicable with regards to Adult Main Study and Pediatric Expansion throughout the protocol.

Abbreviations: AE = adverse event; AESI = adverse event of special interest; COVID-19 = coronavirus disease 2019; DSMB = Data and Safety Monitoring Board; EUA = Emergency Use Authorization; FAS = full analysis set; hACE2 = human angiotensin-converting enzyme 2; IgG = immunoglobulin G; MAAE = medically attended adverse event; MN = microneutralization assay; NI = non-inferiority; NP = nucleocapsid; PBMC = peripheral blood mononuclear cell; PCR = polymerase chain reaction; PP-IMM = Per-Protocol Immunogenicity; PSRT = Protocol Safety Review Team; S = spike; SAE = serious adverse event; SARS-CoV-2 = severe acute respiratory syndrome coronavirus 2; SARS-CoV-2 rS = severe acute respiratory syndrome coronavirus 2 recombinant spike protein nanoparticle vaccine; UBCI = upper bound confidence interval.

Protocol Version 5.0, 02 February 2021 (revised from Version 4.0, 11 January 2021)

The following is a summary of the changes made to the protocol.

Location of Change	Change/Modification in Version 5.0
Section 2 (Synopsis – Secondary Objective); Section 7.2.2	• Added a secondary objective assessing the immunogenicity of a new lot of SARS-CoV-2 rS with Matrix-M1 adjuvant in comparison to the lot utilized in the initial set of vaccinations (ie, immunobridging).
Section 2 (Synopsis – Primary Endpoint); Section 7.4	• Revised definition of tachypnea under Moderate COVID-19 from 20 to 29 breaths per minute to 24 to 29 breaths per minute to better define moderate disease.
Section 2 (Synopsis – Other Secondary Endpoints); Section 7.5.2	• Added a secondary endpoint for the added secondary objective.
Section 2 (Synopsis – Study Design); Section 8.1	• Added statement that enrollment of participants will focus on those for whom vaccines authorized for Emergency Use are not or not anticipated to be recommended or available during the early months of the trial to reduce the number of dropouts during the study.<br>• Clarified that the blinded crossover will commence after demonstration of statistically significant vaccine efficacy and satisfactory safety sufficient to support application for EUA.<br>• Clarified that immune responses immediately following vaccinations will be obtained from all participants after the initial set of vaccinations but not after the crossover vaccinations.<br>• Added that blood samples will be obtained approximately 14 days after the second crossover vaccination dose from approximately 900 participants 18-64 years of age at selected study sites to compare immunogenicity of the vaccine lot utilized for the

Location of Change	Change/Modification in Version 5.0
	crossover vaccination period to that of the earlier lot used for the initial vaccination period.<br>• Clarified that participants who choose to be unblinded prior to the blinded crossover to receive EUA-authorized vaccine will be censored in the final efficacy analysis.<br>• Added that participants who, upon unblinding for the purpose of receiving EUA-authorized vaccine prior to 26 January 2021, learn they had received active vaccine are censored at the time of unblinding but may receive the second dose of Novavax trial vaccine. Participants unblinded between the first (Day 0) and second (Day 21) dose of trial vaccine after 26 January 2021 will not be eligible to receive further investigational product on this protocol. Similarly, participants who choose to withdraw from the blinded trial after having received both doses of blinded study vaccine will not be eligible for blinded crossover.<br>• Added that safety follow-up of the above participants will include remote contacts (eg, phone, email or text to collect SAE, MAAE, AESI and COVID-19 diagnoses at the time points indicated for scheduled visits. No eDiary, nasal swabs or blood samples for immunogenicity will be obtained. Receipt of another COVID-19 vaccine under EUA should be recorded as a concomitant medication.
Section 2 (Synopsis – Safety Analysis Set); Section 12.2.3	• Added that participants who receive another COVID-19 vaccine under EUA will be censored at the time of receipt.
Section 2 (Synopsis – Per-Protocol Efficacy and Immunogenicity Analysis Sets); Section 12.2.4	• Added that participants in the PP-IMM population for the immunobridging analysis will be those at selected study sites who receive the 2 crossover vaccination doses and have blood drawn for anti-SARS-CoV-2 rS IgG antibodies immediately prior to the first crossover vaccination dose and approximately 14 days after the second crossover vaccination dose.
Section 2 (Synopsis – Secondary Endpoints); Section 12.3.2	• Added the statistical analysis of the lot bridging endpoint.
Section 2 (Synopsis – Safety Analysis); Section 12.3.5	• Added that participants who choose to be unblinded prior to the blinded crossover and receive EUA-authorized vaccine will be followed for safety (SAE, MAAE, AESI and COVID-19 diagnosis) by remote contact on the remaining schedule and no further eDiary entries, nasal swabs or blood draws for immunogenicity will be required.
Section 2 (Synopsis – Post Blinded Crossover)	• Clarified the timings of the post-blinded crossover period.
Section 3 (Schedule of Assessments)	• Added a parenthetical note and footnote to the collection of blood for SARS-CoV-2 vaccine immunogenicity at the C2 visit to instruct the study sites to collect blood from a subset of approximately 900 participants at approximately 14 days after the second crossover vaccination dose.

Location of Change	Change/Modification in Version 5.0
Section 6.4 (Study Rationale)	• Clarified that prior to the second dose of vaccine (Day 21), participants may offered the opportunity to be unblinded so that those who received placebo may receive EUA-authorized vaccine, if they choose.<br>• Clarified that participants who choose to be unblinded prior to the blinded crossover to receive EUA-authorized vaccine will be censored in the final efficacy analysis.<br>• Added that participants who, upon unblinding for the purpose of receiving EUA-authorized vaccine prior to 26 January 2021, learn they had received active vaccine are censored at the time of unblinding but may receive the second dose of Novavax trial vaccine. Participants unblinded between the first (Day 0) and second (Day 21) dose of trial vaccine after 26 January 2021 will not be eligible to receive further investigational product on this protocol. Similarly, participants who choose to withdraw from the blinded trial after having received both doses of blinded study vaccine will not be eligible for blinded crossover.
Section 8.1 (Study Design Description)	• Updated Table 2 to clarify that the number of randomized participants is an estimate due to the availability of EUA-authorized vaccines.<br>• Clarified footnote 2 in Table 2 that the blinded crossover will commence after demonstration of statistically significant vaccine efficacy and satisfactory safety sufficient to support application for EUA.
Section 8.2 (Discussion of Study Design)	• Clarified that the blinded crossover will commence after demonstration of statistically significant vaccine efficacy and satisfactory safety sufficient to support application for EUA.<br>• Added statement that enrollment of participants will focus on those for whom vaccines authorized for Emergency Use are not or not anticipated to be recommended or available during the early months of the trial to reduce the number of dropouts during the study.
Section 8.2.2 (Trial Vaccine After the End of Study)	• Clarified that the blinded crossover will commence after demonstration of statistically significant vaccine efficacy and satisfactory safety sufficient to support application for EUA.<br>• Added that participants who, upon unblinding for the purpose of receiving EUA-authorized vaccine prior to 26 January 2021, learn they had received active vaccine are censored at the time of unblinding but may receive the second dose of Novavax trial vaccine. Participants unblinded between the first (Day 0) and second (Day 21) dose of trial vaccine after 26 January 2021 will not be eligible to receive further investigational product on this protocol. Similarly, participants who choose to withdraw from the blinded trial after having received both doses of blinded study vaccine will not be eligible for blinded crossover.<br>• Added that safety follow-up of the above participants will include remote contacts (eg, phone, email or text to collect SAE, MAAE, AESI and COVID-19 diagnoses at the time points indicated for scheduled visits. No eDiary, nasal swabs or blood samples for immunogenicity will be obtained. Receipt of another COVID-19 vaccine under EUA should be recorded as a concomitant medication.
Section 8.4 (Prohibited Medications)	• Added that EUA-authorized vaccines received by participants outside this protocol must be recorded as concomitant medications.
Section 8.6 (Strategies for Recruitment and Retention)	• Added statement that enrollment of participants will focus on those for whom vaccines authorized for Emergency Use are not or not anticipated to be recommended or available during the early months of the trial to reduce the number of dropouts during the study.<br>• Clarified that the blinded crossover will commence after demonstration of statistically significant vaccine efficacy and satisfactory safety sufficient to support application for EUA.

Location of Change	Change/Modification in Version 5.0
Section 8.8.3.1 (Discontinuation/ Withdrawal by Participant)	• Added cross-reference for clarity.
Section 9.4 (Prior Vaccinations and Concomitant Therapy)	• Clarified that vaccines included EUA-authorized vaccines.
Section 9.6 (Procedure for Breaking the Randomization Code)	• Added that participants who, upon unblinding for the purpose of receiving EUA-authorized vaccine prior to 26 January 2021, learn they had received active vaccine are censored at the time of unblinding but may receive the second dose of Novavax trial vaccine. Participants unblinded between the first (Day 0) and second (Day 21) dose of trial vaccine after 26 January 2021 will not be eligible to receive further investigational product on this protocol. Similarly, participants who choose to withdraw from the blinded trial after having received both doses of blinded study vaccine will not be eligible for blinded crossover.
Section 10.2 (Immunogenicity Assessments)	• Added that blood samples will be obtained approximately 14 days after the second crossover vaccination dose from approximately 900 participants 18-64 years of age at selected study sites to compare immunogenicity of the vaccine lot utilized for the crossover vaccination period to that of the earlier lot used for the initial vaccination period.
Section 10.2 (Post Blinded Crossover)	• Section added to match information in synopsis specifying the collection of safety and efficacy endpoints after crossover.
Section 12.1 (Sample Size and Power)	• Added number of primary endpoints needed to conduct each formal interim analysis of efficacy and futility.<br>• Revised sample size language for immunogenicity assessments to accommodate addition of lot bridging analysis.<br>• Added power calculation for lot bridging analysis.
Section 12.3.3 (Statistical Models)	• Corrected error in SAS code used to estimate RR, the relative risk of incidence rates between the 2 trial vaccine groups.<br>• Added statistical model for assessing the lot bridging objective.
Section 12.4 (Interim Analysis)	• Added number of primary endpoints needed to conduct each formal interim analysis of efficacy and futility.
General Changes	• Revised text for consistency across the protocol.

Abbreviations: AESI = adverse event of special interest; C2 = Second crossover vaccination dose; COVID-19 = coronavirus disease 2019; eDiary = electronic patient-reported outcome diary application; EUA = Emergency Use Authorization; IgG = immunoglobulin G; MAAE = medically attended adverse event; PP-IMM = Per-Protocol Immunogenicity; RR = relative risk of incidence rates between the two trial vaccine groups; SAE = serious adverse event; SARS-CoV-2 = severe acute respiratory syndrome coronavirus 2; SARS-CoV-2 rS = severe acute respiratory syndrome coronavirus 2 recombinant spike protein nanoparticle vaccine.

Protocol Version 4.0, 11 January 2021 (revised from Version 3.0, 16 November 2020)

The following is a summary of the changes made to the protocol.

Location of Change	Change/Modification in Version 4.0
Universal Change	A blinded crossover design was added to the protocol to enable all study participants to receive active vaccine following authorization for Emergency Use in the US based on demonstration of statistically significant vaccine efficacy and satisfactory safety in an analysis of the primary endpoint. That is, initial recipients of placebo will receive SARS-CoV-2 rS with Matrix-M1 adjuvant and initial recipients of SARS-CoV-2 rS

Location of Change	Change/Modification in Version 4.0
	with Matrix-M1 adjuvant will receive placebo. The timing of the 2 blinded crossover visits is dependent on the rate of endpoint accrual and the timing of regulatory authorization for Emergency Use.
Section 2 (Synopsis – Primary Objective); Section 7.1	• Clarified that the primary 2-dose regimen of SARS-CoV-2 rS with Matrix-M1 adjuvant comprises a 2-dose regimen.<br>• Clarified that the primary objective was based only on the initial set of vaccinations, not the crossover set of vaccinations.
Section 2 (Synopsis – Key Secondary Objective); Section 7.2.1	• Clarified that the primary 2-dose regimen of SARS-CoV-2 rS with Matrix-M1 adjuvant comprises a 2-dose regimen.<br>• Clarified that the key secondary objective was based only on the initial set of vaccinations, not the crossover set of vaccinations.
Section 2 (Synopsis – Secondary Objective); Section 7.2.2	• Removed nursing homes and healthcare providers and added essential retail workers from examples of living or working conditions involving known frequent exposure to SARS-CoV-2.<br>• Added a secondary objective assessing the durability of vaccine efficacy in active vaccine recipients in both the initial and crossover vaccination periods.<br>• Due to the addition of the crossover period, the Month 3 and 6 visits were removed.<br>• Clarified that the secondary objectives pertained to either the initial vaccination period (reactogenicity) or both the initial and crossover vaccination periods (other safety and immunogenicity objectives).
Section 2 (Synopsis – Exploratory Objectives); Section 7.3	• Clarified that the exploratory objective for cell-mediated immunity pertained only to the initial vaccination period.<br>• Added an exploratory objective on the impact of vaccination on asymptomatic SARS-CoV-2 infection and viral load at the time of the crossover vaccination period.
Section 2 (Synopsis – Key Secondary Endpoint and Other Secondary Endpoints); Sections 7.5.1 and 7.5.2	• Removed language pertaining to the duration of symptoms from the other secondary endpoint as it does not belong in the endpoint definition.
Section 2 (Synopsis – Other Secondary Endpoints); Section 7.5.2	• Due to the addition of the crossover period, the Month 3 and 6 visits were removed.<br>• Clarified that the secondary endpoints pertained to either the initial vaccination period (reactogenicity) or both the initial and crossover vaccination periods (other safety and immunogenicity objectives).
Section 2 (Synopsis – Exploratory Endpoints); Section 7.6	• Clarified that the exploratory endpoint of cell-mediated immunity pertained only to Days 0 and 35 of the initial vaccination period.<br>• Added an exploratory endpoint on the impact of vaccination on asymptomatic SARS-CoV-2 PCR positivity and viral load to the initial vaccination period.<br>• Removed parenthetical text in the exploratory immunogenicity endpoint.
Section 2 (Synopsis – Study Design); Section 8.1	• Removed nursing homes and healthcare providers and added essential retail workers from examples of living or working conditions involving known frequent exposure to SARS-CoV-2.<br>• Added statement regarding the addition of a blinded crossover design.<br>• Clarified that reactogenicity will only be assessed after the initial vaccination period.<br>• Clarified that other safety and immunogenicity assessments will be assessed after the initial and crossover vaccination periods.

Location of Change	Change/Modification in Version 4.0
	• Added that participants who test positive immediately prior to the crossover vaccination period may contribute to the immunogenicity analyses at Months 12, 18 and 24.<br>• Clarified that cell-mediated immunity assessments will be assessed at Days 0, 21, and 35 of the initial vaccination period.<br>• Added that active and passive surveillance of COVID-19 will continue in the crossover vaccination period through 24 months after the initial vaccination period.<br>• Clarified that during the in-person Unscheduled Acute Illness Visit, participants will undergo a targeted physical examination as indicated by the participant's symptoms.<br>• Clarified that medically attended swabs collected at the Unscheduled Acute Illness Visit will be processed at the study site for shipment to the central laboratory.<br>• Added statement that during the first 4 days after the second vaccination when solicited systemic reactogenicity symptoms may be similar to those of COVID-19, investigators should use their clinical judgement to decide if an Unscheduled Acute Illness visit is warranted.<br>• Added that other clinical or laboratory evaluations that may be performed at the discretion of study personnel to inform need for isolation or additional medical care will be outside the scope of the study protocol.<br>• Clarified the timing of the study visits based on the addition of the crossover vaccination period.<br>• Added that participants who experience a positive PCR episode of COVID-19, ie, represent an endpoint, will be eligible to receive the crossover vaccine if they remain in follow-up.<br>• Added statement that the addition of the crossover design will help participants to remain in the study.
Section 2 (Synopsis – Number of Participants)	• Added the randomization schema in the description.
Section 2 (Synopsis – Exclusion Criteria); Section 8.3.2	• Revised the note to Exclusion Criterion 1 to state that verbal report of well-controlled HIV will suffice as documentation.
Section 2 (Synopsis – Test Product, Dose and Mode of Administration); Section 9.1 (Administration of Study Treatment)	• Added statement that in the event of an error in administration of the wrong study material for the first vaccine injection, the participant will be administered the same study material for the second vaccine and will be analyzed in the treatment group received rather than the group to which they were randomized.<br>• Added statement regarding vaccination in the crossover vaccination period.
Section 2 (Synopsis – Duration of Treatment); Section	• Clarified that the duration of the study is approximately 24 months after the initial set of vaccinations.
Section 2 (Synopsis – Full Analysis Set); Section 12.2.2	• Clarified that participants who are unblinded with an intention to receive other COVID-19 vaccines will be censored at the time of unblinding.
Section 2 (Synopsis – Per-Protocol Efficacy and Immunogenicity Analysis Sets); Section 12.2.4	• Clarified that participants who are unblinded with an intention to receive other COVID-19 vaccines will be censored at the time of unblinding.

Location of Change	Change/Modification in Version 4.0
	• Clarified that participants determined to have positive nasal swab PCR or serology immediately prior to the first crossover vaccination will be excluded from the post-crossover PP-EFF population.<br>• Added that immediate immune reaction to study vaccine will be evaluated after the initial set of vaccinations.<br>• Added that durability of immune responses will be evaluated in participants who provide serologic data at Months 12, 18 and 24, taking into account when they received active vaccine and if/when they were infected with SARS-CoV-2, based on PCR or serology.
Section 2 (Synopsis – Safety Analyses);<br>Section 12.3.5	• Clarified that reactogenicity will only be assessed after the initial vaccination period.<br>• Clarified that other safety assessments will be assessed after the initial and crossover vaccination periods.
Section 2 (Synopsis – Monitoring Potential Vaccine Harm);<br>Section 12.5	• Removed statement that only cases of mild, moderate, or severe COVID-19 reported on or after Day 4 will be included in the case count as this language was kept in error as the previous sentence states that the cases will be counted from Day 0.
Section 2 (Synopsis – Post Blinded Crossover)	• New section added to describe follow-up in the crossover vaccination period.
Section 3 (Schedule of Assessments)	• Updated Schedule of Assessments based on addition of crossover design, which created initial and crossover vaccination periods and removed the Month 3 and 6 visits.<br>• Added 2 visits for the crossover vaccination period, 21 days apart, to accommodate vaccine administration during the crossover vaccination period.<br>• Clarified that reactogenicity will only be assessed after the initial vaccination period.<br>• Clarified that other safety and immunogenicity assessments will be assessed after the initial and crossover vaccination periods.
Section 8.1 (Study Design Description)	• Updated Table 2 to add the crossover vaccination period.<br>• Added less definitive language around the 3:1 representation of the respective age stratum and for enrollment of at least 25% of participants > 65 years of age to avoid potential protocol deviations.
Section 8.2 (Discussion of Study Design)	• Updated section based on the addition of the crossover design.<br>• Removed nursing homes and healthcare providers and added essential retail workers from examples of living or working conditions involving known frequent exposure to SARS-CoV-2.
Section 8.2.2 (Trial Vaccine After the End of the Study)	• Updated section based on the addition of the crossover design, including the anticipated incentive for participants to remain in the study.
Section 8.6 (Strategies for Recruitment and Retention)	• Removed nursing homes and healthcare providers and added essential retail workers from examples of living or working conditions involving known frequent exposure to SARS-CoV-2.<br>• Added statement that the addition of the crossover design will help participants to remain in the study.
Section 9.2.2 (Storage)	• Added storage conditions for placebo (0.9% sodium chloride for injection).
Section 9.4 (Prior Vaccinations and Concomitant Therapy)	• Clarified that concomitant medications will be collected through 49 days of the initial vaccination period.

Location of Change	Change/Modification in Version 4.0
Section 9.5 (Blinding and Randomization of Study Treatment)	• Added statement to ensure blinding is maintained during the crossover vaccination period.
Section 9.6 (Procedure for Breaking the Randomization Code)	• Clarified that the data will also be unblinded to the ICON unblinded statistician for the planned interim analyses prior to study completion.
Section 10.1.1 (Active Surveillance for COVID-19)	• Added that active and passive surveillance of COVID-19 will continue in the crossover vaccination period through 24 months after the initial vaccination period.<br>• Added statement that during the first 4 days after the second vaccination when solicited systemic reactogenicity symptoms may be similar to those of COVID-19, investigators should use their clinical judgement to decide if an Unscheduled Acute Illness visit is warranted.
Section 10.1.3 (Nasal Swabs for Virus Detection)	• Added that nasal swabs for virus detection will be obtained at the first crossover vaccination visit.
Section 10.2 (Immunogenicity Assessments)	• Added that blood samples will be obtained during the immediate period after the first set of vaccinations (Day 0, 21 and 35), but only in the long-term (Months 12, 18 and 24) follow-up of all subjects.<br>• Clarified that cell-mediated immunity assessments will be assessed at Days 0, 21, and 35 of the initial vaccination period.
Section 10.3 (Safety Assessments)	• Clarified that reactogenicity will only be assessed after the initial vaccination period.<br>• Clarified that other safety and immunogenicity assessments will be assessed after the initial and crossover vaccination periods.
Section 10.3.8 (Physical Examination)	• Clarified that participants will undergo a targeted physical examination as indicated by the participant's symptoms.
Section 10.4.3 (eDiary)	• Clarified that the eDiary will collect solicited AEs following the initial set of vaccinations only.
Section 12.3 (Statistical Analysis)	• Added text clarifying that the primary analysis of the primary and key secondary efficacy endpoints will be performed prior to the blinded crossover vaccination period.
Section 20 (Reference List)	• Add reference based on assessing durability of vaccine effect following blinded crossover.
General Changes	• Revised text for consistency across the protocol.

Abbreviations: AE = adverse event; COVID-19 = coronavirus disease 2019; eDiary = electronic patient-reported outcome diary application; HIV = human immunodeficiency virus; PCR = polymerase chain reaction; SARS-CoV-2 = severe acute respiratory syndrome coronavirus 2; SARS-CoV-2 rS = severe acute respiratory syndrome coronavirus 2 recombinant spike protein vaccine; US = United States.

Protocol Version 3.0, 16 November 2020 (revised from Version 2.0, 02 October 2020)

The following is a summary of the changes made to the protocol.

Location of Change	Change/Modification in Version 3.0
Section 2 (Primary Endpoint); Section 7.4	• Simplified the primary endpoint by deleting "with each symptom reported for at least 2 consecutive days."
Section 2 (Synopsis - Study Design)	• Added that the procedure for maintaining the blind for all vaccinations is described in the Pharmacy Manual for clarity.

Location of Change	Change/Modification in Version 3.0
	• Clarified that unblinded study site personnel who manage unblinded product can administer the product if qualified to do so.<br>• Clarified that the initiation of the study was Day 0.<br>• Corrected the number of participants in the Immunogenicity Population to 1,200.<br>• Clarified that participants will be provided with an oral thermometer on Day 0 and instructed to monitor body temperature throughout the first 12 months of the study.<br>• Clarified that starting on Day 4, through the first 12 months of the study, suspected symptoms of COVID-19 are to be reported in the eDiary.<br>• Added that medically attended swabs collected at the Unscheduled Acute Illness Visit will be processed according to procedures described in the Laboratory Manual for clarity.<br>• Added definition of mild exercise for participants measuring oxygen saturation for clarity.<br>• Clarified that PCR-positive nasal swabs are to be confirmed at the central laboratory.<br>• Clarified that an Unscheduled General Visit may be conducted by study personnel in the event of a general medical issue other than COVID-19 symptomatology.<br>• Clarified that for participants who terminate the study early, an EoS telephone visit will occur to collect maximum safety data and blood sample, if possible.
Section 2 (Number of Participants and Statistical Methods and Sample Size Calculations)	• Clarified that up to approximately 30,000 participants were to be enrolled in the study.
Section 2 (Synopsis - Exclusion Criteria); Section 8.3.2	• Revised Exclusion Criterion 1b to define undetectable HIV RNA as RNA < 50 copies/mL for clarity.<br>• Clarified that the criteria for meeting Exclusion Criterion 5 is judged to cause significant immunocompromise.<br>• Clarified that the criteria for meeting Exclusion Criterion 8 is chemotherapy judged to cause significant immunocompromise.
Section 2 (Synopsis - Reference Therapy, Dose and Duration of Administration)	• Added the injection volume (0.5 mL) of normal saline (placebo) for clarity.
Section 2 (Synopsis - Statistical Methods and Sample Size Calculations)	• Revised wording of analysis sets to clarify that the PP analysis sets for efficacy and immunogenicity were the primary analysis sets of the study, the ITT analysis set was for participant disposition, and the FAS was for supportive analyses.<br>• Clarified that participants receiving both vaccine and placebo would be analyzed as part of the vaccine group.<br>• Separated the PP-EFF and PP-IMM definitions for clarity and provided for context to the PP-IMM definition for consistency with the protocol.<br>• Added that the review and determination for exclusion from the PP analysis set will be carried out in a blinded fashion by a study clinician prior to unblinding for the interim analysis based on all available information from the locked database.
Section 2 (Synopsis – Primary Endpoint); Section 12.3.1	• Removed country as a covariate based on comments received from the FDA.<br>• Added the definition of VE per FDA guidance.
Section 2 (Synopsis – Secondary Endpoint)	• Changed the alpha for the key secondary endpoint from two-sided alpha of 0.05 to one-sided alpha of 0.025 per FDA comments.

Location of Change	Change/Modification in Version 3.0
	• Added that secondary immunogenicity analyses will also be performed using the FAS.
Section 2 (Synopsis – Interim Analyses)	• Added that the nominal alpha to be spent for the final analysis will be recalculated using the Lan-DeMets alpha spending function based on the actual numbers of events used for the interim analyses and the numbers of endpoints to be used for the final analysis.<br>• Removed language regarding additional interim analyses requested by the DSMB.
Section 2 (Synopsis - Monitoring Potential Vaccine Harm); Section 12.5	• Clarified language pertaining to harm monitoring starting on Day 0 for consistency across the protocol.
Section 2 (Data and Safety Monitoring Board); Section 12.7	• Added mild to rate of moderate or severe COVID-19 for consistency across the protocol.
Section 3 (Schedule of Assessments)	• Added assessment of vital signs at the Screening visit for consistency across the protocol.<br>• Clarified that starting on Day 4, through the first 12 months of the study, suspected symptoms of COVID-19 are to be reported in the eDiary.<br>• Added cross-references to Appendix 2 for AESIs for clarity.
Section 5 (List of Abbreviations)	• The list of abbreviations was updated based on changes made to the synopsis/body of the protocol.
Section 6.5 (Rationale for Dose Selection)	• Clarified that unblinded study site personnel who manage unblinded product can administer the product if qualified to do so.
Section 8.1 (Study Design Description)	• Clarified that up to approximately 30,000 participants (up to 20,000 in the SARS-CoV-2 rS with Matrix-M1 adjuvant group and up to 10,000 in the placebo group).<br>• Added that the procedure for maintaining the blind for all vaccinations is described in the Pharmacy Manual for clarity.<br>• Clarified that unblinded study site personnel who manage unblinded product can administer the product if qualified to do so.<br>• Clarified that the initiation of the study was Day 0.<br>• Corrected the number of participants in the Immunogenicity Population to 1,200.<br>• Clarified that participants will be provided with an oral thermometer on Day 0 and instructed to monitor body temperature throughout the first 12 months of the study.<br>• Clarified that starting on Day 4, through the first 12 months of the study, suspected symptoms of COVID-19 are to be reported in the eDiary.<br>• Added definition of mild exercise for participants measuring oxygen saturation for clarity.<br>• Clarified that PCR-positive nasal swabs are to be confirmed at the central laboratory.<br>• Clarified that an Unscheduled General Visit may be conducted by study personnel in the event of a general medical issue other than COVID-19 symptomatology.<br>• Clarified that for participants who terminate the study early, an EoS telephone visit will occur to collect maximum safety data and blood sample, if possible.<br>• Added mild to rate of moderate or severe COVID-19 for consistency across the protocol.
Section 8.2 (Discussion of Study Design)	• Clarified that up to approximately 30,000 participants were to be enrolled in the study.

Location of Change	Change/Modification in Version 3.0
Section 8.8.1 (Trial Vaccine Discontinuation)	• Added that participants who discontinue due to an AE should be followed to resolution of the AE or determination that it is a chronic condition.
Section 8.8.2 (Study Temporary Discontinuation/Vaccine Pause)	• Added cross-reference to section on vaccine-enhanced disease for clarity.
Section 9.1 [Administration of Study Treatment(s)]; Section 9.5 [Blinding and Randomization of Study Treatment(s)]	• Clarified that unblinded study site personnel who manage unblinded product can administer the product if qualified to do so.
Section 9.2.1 (Packaging and Labelling)	• Clarified that 0.9% sodium chloride for injection will be used for placebo.
Section 9.4 (Prior Vaccinations and Concomitant Therapy)	• Clarified that non-study medications, therapies, or vaccines will be recorded in the eCRF.
Section 9.6 (Procedure for Breaking the Randomization Code)	• Added that the IWRS will also notify the PSRT that the randomization code has been broken.
Section 9.7 (Study Treatment Accountability)	• Added that all used and unused trial vaccines will be reconciled and retained or destroyed according to applicable regulations for consistency with the Pharmacy Manual.
Section 10.1.1 (Active Surveillance for COVID-19)	• Added that symptoms of severe COVID-19 should be reported as an SAE (important medical event) beginning on Day 0 following the first study vaccine administration and appropriate medical care should be sought.<br>• Clarified that starting on Day 4, through the first 12 months of the study, suspected symptoms of COVID-19 are to be reported in the eDiary.
Section 10.1.2 (Unscheduled Acute Illness and Convalescent Visits)	• Added that medically attended swabs collected at the Unscheduled Acute Illness Visit will be processed according to procedures described in the Laboratory Manual for clarity.<br>• Clarified that PCR-positive nasal swabs are to be confirmed at the central laboratory.
Section 10.1.3 (Nasal Swabs for Virus Detection)	• Added that participants who experience an SAE of severe COVID-19 any time after Day 0 should, if at all possible, have a nasal swab obtained (by site personnel or other healthcare personnel) to be sent by the study site to the study central laboratory. Such a swab, if obtained, will constitute the medically attended nasal swab recorded on the Acute Illness Visit form.<br>• Clarified that starting on Day 4, through the first 12 months of the study, suspected symptoms of COVID-19 are to be reported in the eDiary.<br>• Clarified that participants will be instructed on the procedure for arranging transport of swabs to the central lab.
Section 10.1.4 (FLU-PRO)	• Clarified that starting on Day 4, through the first 12 months of the study, suspected symptoms of COVID-19 are to be reported in the eDiary.<br>•
Section 10.1.5 (Oxygen Saturation Monitoring)	• Added definition of mild exercise for participants measuring oxygen saturation for clarity.<br>•

Location of Change	Change/Modification in Version 3.0
Section 10.2 (Immunogenicity Assessments)	• Corrected the number of participants in the Immunogenicity Population to 1,200.
Section 10.3 (Safety Assessments); Section 10.3.1.5 (Adverse Event of Special Interest)	• Clarified that AESIs specific to complications of potential disease enhancement for COVID-19 will also be monitored.
Section 10.3.1.3 (Serious Adverse Events)	• Added that events of severe COVID-19 constitute important medical events for this study.
Section 10.3.1.7 (Reactogenicity Symptoms)	• Clarified that reactogenicity events extending beyond 7 days after vaccination will be recorded as an AE with a start date that matches Day 7.<br>• Added that at any time after Day 0, severe COVID-19 should be reported as an SAE and managed accordingly.
Section 10.3.4 (Recording of AEs and SAEs)	Added that participants who experience an SAE of severe COVID-19 any time after Day 0 should, if at all possible, have a nasal swab obtained (by site personnel or other healthcare personnel) to be sent by the study site to the study central laboratory. Such a swab, if obtained, will constitute the medically attended nasal swab recorded on the Acute Illness Visit form.
Section 10.3.9 (Vital Signs)	• Added that blood pressure should not exceed medically acceptable limits to ensure participants with uncontrolled hypertension are not included for clarity.<br>• Added that participants considered to have "white coat hypertension" should have a reduction of in blood pressure documented following a calming period.
Section 10.4.1 (Medical and Social History)	• Added that living and working or school conditions will be recorded to assess possible high-risk environments.
Section 10.4.3 (eDiary)	• Clarified that study site personnel and central monitoring personnel will regularly review the eDiary for completeness.
Section 12.1 (Sample Size and Power)	• Clarified that up to approximately 30,000 participants were to be enrolled in the study.<br>• Corrected the number of participants in the Immunogenicity Population to 1,200, including the breakdown of randomly selected participants per country and age cohort.<br>• Deleted the immunogenicity sample size assumptions.<br>• Deleted the specifics of the random selection of participant for the additional 2-stage random samplings to assess immunogenicity.
Section 12.2 (Analysis Sets)	• Revised wording of analysis sets to clarify that the PP analysis sets for efficacy and immunogenicity were the primary analysis sets of the study, the ITT analysis set was for participant disposition, and the FAS was for supportive analyses.<br>• Clarified that participants receiving both vaccine and placebo would be analyzed as part of the vaccine group.<br>• Separated the PP-EFF and PP-IMM definitions for clarity and clarified the PP-IMM definition.
Section 12.3.2 (Secondary Endpoints)	• Changed the alpha for the key secondary endpoint from two-sided alpha of 0.05 to one-sided alpha of 0.025 per FDA comments.<br>• Added that secondary immunogenicity analyses will also be performed using the FAS.<br>• Deleted the analysis of covariance model for non-randomized comparisons of subgroups for the immunogenicity analysis.

Location of Change	Change/Modification in Version 3.0
Section 12.3.3 (Statistical Models)	• Removed country as a covariate based on comments received from the FDA.
Section 12.4 (Interim Analysis)	• Added that the nominal alpha to be spent for the final analysis will be recalculated using the Lan-DeMets alpha spending function based on the actual numbers of events used for the interim analyses and the numbers of endpoints to be used for the final analysis.<br>• Corrected the nominal alpha for the second interim analysis at 75% to 0.00916.<br>• Removed language regarding additional interim analyses requested by the DSMB.
Section 12.6 (Safety Monitoring)	• Clarified the composition of the PSRT.
Section 14.2 (Site Monitoring)	• Removed text pertaining to local studies conducted in Japan.
Section 20 (Reference List)	• Updated reference list based on changes made to the protocol.
Appendix 2	• Added table numbers to the AESIs of PIMMC and of complications of COVID-19 for cross-reference purposes.<br>• Added reference for Coalition for Epidemic Preparedness Innovations/Brighton Collaboration Consensus Meeting.
General Changes	• Revised text for consistency across the protocol.

Abbreviations: AE = adverse event; AESI = adverse event of special interest; BMI = body mass index; COVID-19 = coronavirus disease 2019; DSMB = Data and Safety Monitoring Board; eDiary = electronic patient-reported outcome diary application; EoS = end of study; HIV = human immunodeficiency virus; IWRS = Interactive web response system; PCR = polymerase chain reaction; PSRT = Protocol Safety Review Team; RNA = ribonucleic acid; SAE = serious adverse event; SARS-CoV-2 rS = severe acute respiratory syndrome coronavirus 2 recombinant spike protein vaccine.

Protocol Version 2.0, 02 October 2020 (revised from Version 1.0, 21 August 2020)

The following is a summary of the changes made to the protocol.

Location of Change	Change/Modification in Version 2.0
Title Page	• Matrix-M1™ adjuvant was added to test product because SARS-CoV-2 rS is an adjuvanted vaccine and the 2 products are co-formulated together.
Section 2 (Synopsis - Principal Investigator)	• The Principal Investigator was revised from "To Be Changed" to "This is a multicenter study."
Section 2 (Synopsis - Study Centers)	• The number of study sites was decreased from 300 to approximately 125, and the location of sites outside of North America were condensed to globally (if needed).
Section 2 (Synopsis - Planned Study Period)	• The start of the planned study period was changed from September to October 2020.
Section 2 (Synopsis - Primary Objective); Section 7.1	• Text was added to the primary objective for clarity.
Section 2 (Synopsis - Key Secondary Objective); Section 7.2.1	• Text was added to the key secondary objective for consistency with the text in the primary objective.
Section 2 (Synopsis – Other Secondary Objectives); Section 7.2.2	• Revised name of section to delineate from key secondary objective section.<br>• Added new secondary objective assessing VE against any symptomatic SARS-CoV-2 infection.

Location of Change	Change/Modification in Version 2.0
	• Deleted secondary objective on VE of high-risk participants because it was redundant to a subsequent secondary objective.<br>• Added definition of high-risk participants to secondary objective for clarity.<br>• Clarified wording on secondary objectives of efficacy, immunogenicity, safety, mortality, and anti-SARS-CoV-2 NP antibodies.<br>• Clarified that all unsolicited AEs and MAAEs would be assessed from Day 0 through Day 49 and MAAEs attributed to vaccine would be assessed through the EoS.<br>• Added specificity to the secondary objective assessing medical resource utilization.<br>• Added new secondary objective assessing VE against SARS-CoV-2 infection determined by anti-SARS-CoV NP antibodies.
Section 2 (Synopsis - Exploratory Objectives); Section 7.3	• Revised wording of NIH objective for clarity.<br>• Added 2 exploratory objectives pertaining to nasal virus load and sequencing genetic material from SARS-CoV-2 viruses.
Section 2 (Synopsis - Primary Endpoint); Section 7.4	• Text was added to clarify that the primary endpoint would be assessed at the first episode of mild, moderate, or severe COVID-19 with each symptom reported for at least 2 consecutive days.<br>• Removed 48-hour durations for headache and nausea, vomiting or diarrhea based on the change in the endpoint text requiring each symptom to be reported for at least 2 consecutive days.
Section 2 (Synopsis - Key Secondary Endpoint); Section 7.5.1	• Text was added to clarify the key secondary endpoint for consistency with the primary endpoint and to cross-reference the same information that is included in the primary endpoint for moderate and severe COVID-19.
Section 2 (Synopsis - Other Secondary Endpoints); Section 7.5.2	• Added new secondary endpoint corresponding to the new secondary objective assessing VE against any symptomatic SARS-CoV-2 infection.<br>• Revised secondary endpoints based on changes made to secondary objectives.<br>• Added secondary endpoint corresponding to the description of the course, treatment, and severity of COVID-19.<br>• Clarified that the injection site reactions of swelling and induration are synonymous, separated arthralgia and myalgia and added systemic reactogenicity reactions of headache and nausea/vomiting.<br>• Added secondary endpoints of unsolicited AEs, MAAEs, SAEs, and AESIs to correspond with the associated secondary objective.<br>• Removed secondary endpoint pertaining to severity of reactogenicity as this endpoint is covered under the other reactogenicity endpoint.<br>• Added secondary endpoint on death due to any cause to correspond with mortality objective.<br>• Added secondary endpoint on antibodies to SARS-CoV-2 NP to correspond with new secondary objective.
Section 2 (Synopsis - Exploratory Endpoints); Section 7.6	• The designated subset of 1,600 Immunogenicity Population participants for NIAID testing was increased to approximately 4,500 participants based on feedback from the CoVPN and OWS external statistics groups.<br>• Added clarifying text to the NIAID endpoint.<br>• Added 2 exploratory endpoints corresponding to the added secondary objectives pertaining to nasal virus load and sequencing genetic material from SARS-CoV-2 viruses.
Section 2 (Synopsis - Study Design)	• Clarified the age strata as 18 to 64 years and ≥ 65 years.

Location of Change	Change/Modification in Version 2.0
	• Removed text implying that the Phase 3 study could commence with the younger age cohort (18 to 64 years) based on feedback from OWS. OWS requires that both age strata be initiated concurrently.<br>• Added that 25% of the study population will be ≥ 65 years.<br>• Further defined the high-risk participant population and added the targeted percentage of older participants based on OWS feedback.<br>• Clarified that the selected dose level of SARS-CoV-2 rS with Matrix-M1 adjuvant was based on optimal safety and immunogenicity data from nonclinical and early clinical data.<br>• Clarified that the eDiary would collect solicited AEs of reactogenicity, AEs, and COVID-19 symptomatology, participants will be trained on the eDiary at the initiation of the study, and a Help Desk will be available for eDiary technical issue and the study site for data entry issues.<br>• Clarified the timing of safety assessments during the first 12 months of follow-up post final vaccination and during the second 12 months of follow-up post final vaccination.<br>• Defined the serologic assessments in the study.<br>• The number of participants in the Immunogenicity Population was corrected to 1,600 and text was added to clarify the representative study population.<br>• Added that participants who have a PCR-positive nasal swab indicating SARS-CoV-2 infection at baseline will have SARS-CoV-2 S protein immune responses analyzed, but that these data will not contribute to the primary immunogenicity or efficacy analyses.<br>• Clarified the recording of other relevant symptoms in the eDiary and noted that participants will be contacted by phone if they are not compliant with it.<br>• Added instructions on nasal swab maintenance and shipping.<br>• Removed text regarding providing all participants with pulse oximeters at enrollment.<br>• Added Unscheduled Illness and Unscheduled Convalescent Visits to monitor participants with PCR-confirmed COVID-19 and added Unscheduled General Visit for all other medical issues.<br>• Clarified the EoS visit.<br>• Added text encouraging participants to continue in the study to follow safety and reported COVID-19 cases.<br>• Added text summarizing the safety monitoring in the study.<br>• Clarified the requirements for vaccination of placebo participants in case the Novavax vaccine or another product are approved by regulatory authorities.
Section 2 (Synopsis - Inclusion Criteria); Section 8.3.1	• Removed males or females from Inclusion Criterion 1 as the text is not needed.<br>• Added text to Inclusion Criterion 1 to specify enrollment of participants who are at substantial risk of exposure or infection with SARS-CoV-2 to ensure enough endpoint events are captured for the primary analysis.<br>• Clarified the definition of female participants of childbearing potential in Inclusion Criterion 3.

Location of Change	Change/Modification in Version 2.0
Section 2 (Synopsis - Exclusion Criteria); Section 8.3.2	• Clarified Exclusion Criteria 1, 3, 5, and 7.<br>• Revised Exclusion Criterion 4 to exclude participants who received influenza vaccine or any other adult vaccine within 4 days prior to or within 7 days after each study vaccination to avoid confounding reactogenicity observations.<br>• Revised Exclusion Criterion 5 to specify conditions requiring ongoing immunomodulatory therapy and revised the definition of diabetes mellitus.<br>• Removed prior Exclusion Criterion 8 as it is captured under other conditions posing a health risk to participants.<br>• Revised new Exclusion Criterion 8 to broaden the study population by allowing participants with a history of active cancer beyond 1 year instead of 5 years.<br>• Revised Exclusion Criterion 9 to removed latex allergy as a contraindication because there is no latex in contact with the product/injection.<br>• Revised Exclusion Criterion 10 to broaden the study population by allowing participants who plan to become pregnant 3 months after the second vaccination consistent with the respective Inclusion Criterion 3.
Section 2 (Synopsis - Test Product, Dose and Mode of Administration)	• Changed "up to" 2 IM injections to 2 IM injections to clarify that planned vaccination consists of 2 doses.<br>• Added "+ 7 days" to the Day 21 vaccination day.
Section 2 (Synopsis - Reference Therapy, Dose and Duration of Administration)	• Added "normal saline" as the definition of placebo for clarity.
Section 2 (Synopsis - Statistical Methods and Sample Size Calculations)	• The addition of the second interim analysis increased the target number of PCR-confirmed SARS-CoV-2 infections from 141 to 144.<br>• Briefly summarized the 2 formal interim analyses of efficacy and futility that will be conducted based on the accumulation of approximately 50% and 75% of the total anticipated primary endpoints.
Section 2 (Synopsis [Intent-to-Treat Efficacy (ITT-EFF) and Immunogenicity (ITT-IMM) Analysis Sets]); Section 12.2.1	• Revised the Analysis Sets for ITT-EFF and ITT-IMM due to changes made in the PP-EFF and PP-IMM Analysis Sets.
Section 2 (Synopsis [Full Analysis Efficacy (FAS-EFF) and Immunogenicity (FAS-IMM) Analysis Sets]); New Section 12.2.2	• Added FAS-EFF and FAS-IMM Analysis Sets for supportive analyses.
Section 2 (Synopsis [Per-Protocol Efficacy (PP-EFF) and Immunogenicity (PP-IMM) Analysis Sets]); Section 12.2.4	• Revised PP-EFF and PP-IMM Analysis Sets to clarify that no major protocol deviations were to occur prior to the first COVID-19 positive episode and to exclude participants with confirmed infection or prior infection due to SARS-CoV-2 at baseline from the PP populations.
Section 2 (Synopsis - Primary Endpoint); Section 12.3.1	• Added country and age strata in the primary endpoint analysis.<br>• Added that cases are to be counted starting 7 days after the second vaccination (Day 28).<br>• Clarified hypothesis testing for VE including revision of statistical methodology.

Location of Change	Change/Modification in Version 2.0
Section 2 (Synopsis - Secondary Endpoints); Section 12.3.2	• Updated the key secondary and other efficacy endpoint analyses.<br>• Clarified the immunogenicity assessments to be analyzed.<br>• Removed the analysis of SCR.<br>• Added country and vaccine lot to the secondary endpoint analyses.
Section 2 (Synopsis - Safety Analyses); Section 12.3.5	• Added that safety data will be reviewed weekly by the 2019nCoV-301 PSRT to detect possible signals of solicited and unsolicited AEs.
Section 2 (Synopsis - Interim Analyses); Section 12.4	• Added second planned interim analysis and revised timing of both planned interim analyses from 66.7% (original planned analysis) to 50% and 75%, respectively of the targeted total number of cases.<br>• Updated the statistical methods around this analysis based on feedback from the DSMB.<br>• Added text regarding the DSMB's ability to request additional safety and efficacy analyses during the study based on feedback received from the DSMB and OWS.
Section 2 (Synopsis - Monitoring Potential Vaccine Harm); Section 12.5	• Added new section to monitor the study for potential vaccine harm on imbalance in the primary efficacy endpoint.
Section 2 (Synopsis - Data and Safety and Monitoring Board); Section 12.7	• Changed section title from Data Monitoring Committee to Data and Safety Monitoring Board.<br>• Removed duplicate text regarding the treatment of placebo participants with an approved vaccine.<br>• Added text regarding additional functions of the DSMB.

Location of Change	Change/Modification in Version 2.0
Section 3 (Schedule of Assessments)	• Added Unscheduled Illness and Unscheduled Convalescent Visits to monitor participants with PCR-confirmed COVID-19 and changed Unscheduled visit to Unscheduled General Visit for all other medical issues.<br>• Added minimum days following most recent vaccination to Months 6, 12, 18, and 24 visits.<br>• Clarified medical history to medical and social history.<br>• Removed prior/concomitant medications, vital sign measurements, blood sampling for SARS-CoV-2 anti-NP antibodies, and any MAAE attributed to vaccine assessments from the screening visit.<br>• Added blood sampling for SARS-CoV-2 anti-NP antibodies to the Day 0 visit<br>• Added prior/concomitant medications; targeted physical examination; blood sampling for SARS-CoV-2 anti-NP antibodies and immunogenicity; and unsolicited AEs, MAAEs, MAAEs since last visit, MAAEs attributed to vaccine, SAEs, and AESIs to the Unscheduled Acute Illness and Unscheduled Convalescent Visits.<br>• Added footnote at Month 3 visit limiting assessment of unsolicited AEs and MAAEs to Day 49 for clarity.<br>• Added nasal swab at clinic to the Unscheduled Acute Illness Visit.<br>• Added Endpoint Review assessments to the Unscheduled Convalescent Visit.<br>• Added prior/concomitant medications and MAAEs attributed to vaccine to the 18- and 24-Month visits for clarity.<br>• Added text describing when the participant should nasal self-swab and assess oxygen saturation during the study.<br>• Updated nasal swab collection area to anterior nares only.<br>• Updated SoA list of abbreviations.<br>• Updated SoA footnotes based on changes made to the synopsis/body of the protocol.
Section 5 (List of Abbreviations)	• The list of abbreviations was updated based on changes made to the synopsis/body of the protocol.
Section 6.1 (Background)	• Removed "NVXCoV2373" terminology since NVX-CoV2373 represents both the antigen (SARS-CoV-2 rS) and adjuvant (Matrix-M1) and is not used elsewhere in this document for clarity.<br>• Changed "up to" 2 IM injections to 2 IM injections to clarify that planned vaccination consists of 2 doses.<br>• Added "+ 7 days" to the Day 21 vaccination day.<br>• Removed repetitive text regarding the treatment of placebo participants with an approved vaccine.
Section 6.2 (Nonclinical Summary)	• Updated dose-titration studies in various animal models.<br>• Added journal reference to Phase 1 clinical trial.
Section 6.3 (Clinical Summary)	• Clarified Phase 1 (Part 1) text and added information on the Phase 2 (Part 2) study.

Location of Change	Change/Modification in Version 2.0
Section 6.4 (Study Rationale)	• Clarified Phase 1 (Part 1) text and added journal reference for Phase 1 study.<br>• Added summary of Phase 2 (Part 2) reactogenicity data.<br>• Removed text implying that the Phase 3 study could commence with the younger age cohort (18 to 64 years) based on feedback from OWS. OWS requires that both age strata be initiated concurrently.<br>• Changed "up to" 2 IM injections to 2 IM injections to clarify that planned vaccination consists of 2 doses.<br>• Added "+ 7 days" to the Day 21 vaccination day.<br>• Added text regarding the treatment of placebo participants with an approved vaccine.
Section 6.5 (Rationale for Dose Selection)	• Added journal reference for Phase 1 study.
Section 6.6 (Benefit – Risk Assessment)	• Updated Benefit-Risk section to add that antigen is not produced using infectious SARS-CoV-2.<br>• Added mitigation approach to AEs related to hypersensitivity with text from the IB.
Section 6.6.2 (Overall Benefit – Risk Conclusion)	• Added Phase 2 (Part 2) safety data that showed a similar reactogenicity profile between younger and older participants, with both local and systemic reactogenicity events generally occurring less frequently in older adults.
Section 8.1 (Study Design Description)	• Clarified the age strata as 18 to 64 years and ≥ 65 years.<br>• Removed text implying that the Phase 3 study could commence with the younger age cohort (18 to 64 years) based on feedback from OWS. OWS requires that both age strata be initiated concurrently.<br>• Clarified that the majority of participants will be consented, enrolled and vaccinated on Day 0.<br>• Further defined the high-risk participant population and added the targeted percentage of older participants based on OWS feedback.<br>• Specified race and ethnicity to ensure a diverse study population.<br>• Clarified that the selected dose level of SARS-CoV-2 rS with Matrix-M1 adjuvant was based on optimal safety and immunogenicity data from nonclinical and early clinical data.<br>• Clarified that the eDiary would collect solicited AEs of reactogenicity, AEs, and COVID-19 symptomatology, participants will be trained on the eDiary at the initiation of the study, and a Help Desk will be available for eDiary technical issue and the study site for data entry issues.<br>• Clarified the timing of safety assessments during the first 12 months of follow-up post final vaccination and during the second 12 months of follow-up post final vaccination.<br>• Defined the serologic assessments in the study.<br>• The number of participants in the Immunogenicity Population was corrected to 1,600 and text was added to clarify the representative study population.<br>• Added that participants who have a PCR-positive nasal swab indicating SARS-CoV-2 infection at baseline will have SARS-CoV-2 S protein immune responses analyzed, but that these data will not contribute to the primary immunogenicity or efficacy analyses.<br>• Clarified the recording of other relevant symptoms in the eDiary and noted that participants will be contacted by phone if they are not compliant with it.<br>• Added instructions on nasal swab maintenance and shipping.

Location of Change	Change/Modification in Version 2.0
	• Removed text regarding providing all participants with pulse oximeters at enrollment.<br>• Added Unscheduled Illness and Unscheduled Convalescent Visits to monitor participants with PCR-confirmed COVID-19 and added Unscheduled General Visit for all other medical issues.<br>• Clarified the EoS visit.<br>• Added text encouraging participants to continue in the study to follow safety and reported COVID-19 cases.<br>• Added text summarizing the safety monitoring in the study.<br>• Clarified the requirements for vaccination of placebo participants in case the Novavax vaccine or another product are approved by regulatory authorities.<br>• Removed duplicate text regarding the treatment of placebo participants with an approved vaccine.<br>• Added text regarding additional functions of the DSMB.
Section 8.2 (Discussion of Study Design)	• Revised text for consistency with the changes made to the Study Design section for clarity.
Section 8.2.2 (Trial Vaccine After the End of Study)	• Revised text regarding the treatment of placebo participants with an approved vaccine for consistency with similar text in the Study Design section.
Section 8.4 (Prohibited Medications)	• Added criteria for prohibiting administration of seasonal influenza vaccine during the study.
Section 8.6 (Strategies for Recruitment and Retention)	• Updated section for consistency with the Study Design section and added specifics regarding the percentage of high-risk participants that should be targeted for enrollment into the study.
Old Section 8.8 (Unscheduled Visits)	• Moved section to Efficacy Assessments (Section 10.1.2).
New Section 8.8 (Trial Vaccine Discontinuation and Participant Discontinuation/ Withdrawal)	• Section title was revised to include participant discontinuation and withdrawal.<br>• Subsections were added to clarify reasons for trial vaccine discontinuation, temporary discontinuation/vaccine pause, and withdrawal of participants by participants, investigator, and Sponsor.
Section 9 (Study Treatment)	• Revised text for clarity.<br>• Moved Study Treatment Accountability section to end of section.
Section 10.1 (Efficacy Assessments)	• Revised text for consistency with Study Design section and added descriptions of the Unscheduled Acute Illness and Unscheduled Convalescent Visits for assessment and monitoring of confirmed cases of symptomatic COVID-19.<br>• Added description of nasal swabs for virus detection,<br>• Added text on when participants will be instructed on the FLU-PRO questionnaire.<br>• Added text on the number of participants who completed the FLU-PRO for historical reference.
Section 10.2 (Immunogenicity Assessments)	• Defined the serologic assessments in the study.<br>• The number of participants in the Immunogenicity Population was corrected to 1,600 and text was added to clarify the representative study population.
Section 10.3.1.2 (Events Meeting the AE Definition)	• Signs, symptoms, or clinical sequelae of a suspected overdose were removed from this section because overdose was already described in Section 10.3.10 of the protocol.

Location of Change	Change/Modification in Version 2.0
Section 10.3.1.7 (Reactogenicity Symptoms)	• Added cross-reference to FDA toxicity criteria for assessment of severity, which can be found in Appendix 4.
Section 10.3.2 (Time Period and Frequency for Collecting AE and SAE Information)	• Clarified that all AEs reported or observed during the study will be recorded on the AE page of the eCRF.<br>• Clarified that MAAEs attributed to vaccine (not MAAEs) would be assessed through the EoS.
Section 10.3.4.1 [Assessment of Intensity (Severity)]	• Replaced severity criteria used to assess reactogenicity with standard severity criteria for unsolicited AEs.
Section 10.3.6.2 (Safety Reporting to Health Authorities, Independent Ethics Committees/ Institutional Review Boards and Investigators)	• Text revised to state that Novavax will have responsibility for safety reporting to the US FDA and ICON for all other reporting.
Section 10.3.8 (Physical Examination)	• Added text that weight and BMI would be re-assessed if clinically indicated.
Section 10.3.9 (Vital Signs)	• Corrected body temperature that should not be exceeded from 30°C to 38°C.
Old Section 10.4 (Study Halting Rules)	• The contents of this section were moved to revised Trial Vaccine Discontinuation and Participant Discontinuation/Withdrawal.
Section 10.3.10 (Overdose)	• Clarified the reporting of overdose as either an AE or SAE.
New Section 10.4 (Other Assessments)	• New section added to describe assessments not related to efficacy and safety, including description of the eDiary.
Section 11 (Medical Resource Utilization)	• Title and content of section was revised to focus on medical resource allocation for consistency with the efficacy endpoints of the study.
Section 12.1 (Sample Size and Power)	• The addition of the second interim analysis increased the target number of PCR-confirmed SARS-CoV-2 infections from 141 to 144.<br>• Added second planned interim analysis and revised timing of both planned interim analyses from 66.7% (original planned analysis) to 50% and 75%, respectively of the targeted total number of cases.<br>• Updated the estimated power calculations based on the changes made to the planned interim analyses.<br>• Described the rationale for the sample size of the Immunogenicity Population.<br>• Added description of two-stage random samplings to facilitate the case-cohort sampling design.
Section 12.3.3 (Statistical Models)	• Added country and age strata as covariates.<br>• Revised that cases were to be counted starting 7 days (not 14 days) after the second vaccination.<br>• Added Cox proportional hazards model description.
Section 12.3.4 (Handling of Missing Data)	• Section was revised for clarity.

Location of Change	Change/Modification in Version 2.0
Section 12.6 (Safety Monitoring)	• Added new section describing the safety monitoring in the study.
Section 14.2 (Site Monitoring)	• Removed text pertaining to local studies conducted in Japan.
Section 15.1 (Case Report Forms/Source Data Handling)	• Revised text for clarity.<br>• Removed laboratory test text since no clinical laboratory tests will be performed in the study.
Section 17 (Publication Policy)	• Added text providing authors reasonable access to all study data, statistical tables, figures and relevant reports to review complete study results.
Section 19 (Signature of Investigator)	• Section was updated for clarity.
Section 20 (Reference List)	• Updated reference list based on changes made to the protocol.
Section 21 (Appendices)	• Added an appendix (Appendix 1) to list the changes to the protocol.<br>• Added an appendix (Appendix 3) providing example stopping bounds for vaccine harm monitoring.<br>• Added an appendix (Appendix 4) providing the FDA grading scale for local and general reactogenicity.
General Changes	• Matrix-M adjuvant changed to Matrix-M1 adjuvant, where applicable because that is the specific adjuvant used in the trial.<br>• Corrected use of abbreviations in text by defining abbreviations at first use and applying abbreviations they have been defined.<br>• Revised text for consistency across the protocol.

Abbreviations: AE = adverse event; AESI = adverse event of special interest; BMI = body mass index; COVID-19 = coronavirus disease 2019; CoVPN = COVID-19 Prevention Network; DSMB = Data and Safety Monitoring Board; eDiary = electronic patient-reported outcome diary application; EoS = end of study; FAS-EFF = Full Analysis Set – Efficacy; FAS-IMM = Full Analysis Set – Immunogenicity; FDA = United States Food and Drug Administration; FLU-PRO = InFLUenza Patient-Reported Outcome (questionnaire); IB = Investigator's Brochure; IM = intramuscular; ITT-EFF = Intent-to-Treat – Efficacy; ITT-IMM = Intent-to-Treat – Immunogenicity; MAAE = medically attended adverse event; NP = nucleocapsid; OWS = Operation Warp Speed; PP-EFF = Per Protocol – Efficacy; PP-IMM = Per Protocol – Immunogenicity; PCR = polymerase chain reaction; PSRT = Protocol Safety Review Team; SAE = serious adverse event; SAP = statistical analysis plan; SARS-CoV-2 = severe acute respiratory syndrome coronavirus 2; SARS-CoV-2 rS = severe acute respiratory syndrome coronavirus 2 recombinant spike protein vaccine; SCR = seroconversion response; SoA = Schedule of Assessments; VE = vaccine efficacy.

Appendix 2: Listings of Adverse Events of Special Interest

Because it has been hypothesized that immunizations with or without adjuvant may be associated with autoimmunity, regulatory authorities have requested that Novavax instruct investigators to be especially vigilant regarding the PIMMC listed below (Table 11). Note that this regulatory request is not specific to Novavax's SARS-CoV-2 rS or Matrix-M1 adjuvant; and there is no current evidence to suggest that the trial vaccines in this protocol are, or are not, associated with these illnesses. The list is not intended to be exhaustive, nor does it exclude the possibility that other diagnoses may be AESI.

Table 11 Potential Immune-Mediated Medical Conditions

Categories	Diagnoses (as MedDRA Preferred Terms)
Neuroinflammatory Disorders:	Acute disseminated encephalomyelitis (including site-specific variants: eg, non-infectious encephalitis, encephalomyelitis, myelitis, myeloradiculomyelitis), cranial nerve disorders including paralyses/paresis (eg, Bell's palsy), generalized convulsion, Guillain-Barre syndrome (including Miller Fisher syndrome and other variants), immune-mediated peripheral neuropathies and plexopathies (including chronic inflammatory demyelinating polyneuropathy, multifocal motor neuropathy and polyneuropathies associated with monoclonal gammopathy), myasthenia gravis, multiple sclerosis, narcolepsy, optic neuritis, transverse myelitis, uveitis.
Musculoskeletal and Connective Tissue Disorders:	Antisynthetase syndrome, dermatomyositis, juvenile chronic arthritis (including Still's disease), mixed connective tissue disorder, polymyalgia rheumatic, polymyositis, psoriatic arthropathy, relapsing polychondritis, rheumatoid arthritis, scleroderma (including diffuse systemic form and CREST syndrome), spondyloarthritis (including ankylosing spondylitis, reactive arthritis [Reiter's Syndrome] and undifferentiated spondyloarthritis), systemic lupus erythematosus, systemic sclerosis, Sjogren's syndrome.
Vasculitides:	Large vessels vasculitis (including giant cell arteritis such as Takayasu's arteritis and temporal arteritis), medium sized and/or small vessels vasculitis (including polyarteritis nodosa, Kawasaki's disease, microscopic polyangiitis, Wegener's granulomatosis, Churg–Strauss syndrome [allergic granulomatous angiitis], Buerger's disease [thromboangiitis obliterans], necrotizing vasculitis and ANCA-positive vasculitis [type unspecified], Henoch-Schonlein purpura, Behcet's syndrome, leukocytoclastic vasculitis).
Gastrointestinal Disorders:	Crohn's disease, celiac disease, ulcerative colitis, ulcerative proctitis.
Hepatic Disorders:	Autoimmune hepatitis, autoimmune cholangitis, primary sclerosing cholangitis, primary biliary cirrhosis.
Renal Disorders:	Autoimmune glomerulonephritis (including IgA nephropathy, glomerulonephritis rapidly progressive, membranous glomerulonephritis, membranoproliferative glomerulonephritis, and mesangioproliferative glomerulonephritis).
Cardiac Disorders:	Autoimmune myocarditis/cardiomyopathy.

Table 11 Potential Immune-Mediated Medical Conditions

Categories	Diagnoses (as MedDRA Preferred Terms)
Skin Disorders:	Alopecia areata, psoriasis, vitiligo, Raynaud's phenomenon, erythema nodosum, autoimmune bullous skin diseases (including pemphigus, pemphigoid and dermatitis herpetiformis), cutaneous lupus erythematosus, morphoea, lichen planus, Stevens-Johnson syndrome, Sweet's syndrome.
Hematologic Disorders:	Autoimmune hemolytic anemia, autoimmune thrombocytopenia, antiphospholipid syndrome, thrombocytopenia.
Metabolic Disorders:	Autoimmune thyroiditis, Grave's or Basedow's disease, new onset Hashimoto thyroiditis[a], diabetes mellitus type 1, Addison's disease.
Other Disorders:	Goodpasture syndrome, idiopathic pulmonary fibrosis, pernicious anemia, sarcoidosis.

Abbreviations: ANCA = anti-neutrophil cytoplasmic antibody; IgA = immunoglobulin A; MedDRA = Medical Dictionary for Regulatory Activities.

AEs specific to COVID-19 are listed below (Table 12). The list is not intended to be exhaustive, nor does it exclude the possibility that other diagnoses may be AESI.

Table 12 Adverse Events Representing Complications Specific to of COVID-19[1]

Categories	Diagnoses (as MedDRA System Organ Class/Preferred Term)
Respiratory/Infectious Disorders:	ARDS, pneumonitis, septic shock-like syndrome.
Cardiac Disorders:	Acute cardiac injury, arrhythmia.
Coagulopathy	Deep vein thrombosis, myocardial infarction, stroke.
Renal Disorders:	Acute kidney injury.
Hematologic Disorder	Thrombocytopenia, septic shock-like syndrome.
Inflammatory Disorders:	Cytokine Release Syndrome related to COVID-19 infection[2], multisystem inflammatory syndrome in children (MIS-C).
Neurologic Disorders:	Generalized convulsions.

Abbreviations: ARDS = acute respiratory distress syndrome; COVID-19 = coronavirus disease 2019; DAIDS = Division of AIDS; MedDRA = Medical Dictionary for Regulatory Activities.

1. COVID-19 manifestations associated with more severe presentation and decompensation with consideration of enhanced disease potential. The current listing is based on Coalition for Epidemic Preparedness Innovations /Brighton Collaboration Consensus Meeting (12/13 March 2020) and expected to evolve as evidence accumulates [Lambert 2020].
2. Cytokine release syndrome related to COVID-19 infection is a disorder characterized by nausea, headache, tachycardia, hypotension, rash, and/or shortness of breath [DAIDS, 2017].

Appendix 3: Example Stopping Bounds for Vaccine Harm Monitoring

Example stopping bounds for harm monitoring based on the randomization ratio. Each test is based on exact binomial test given the total number of cases at one-sided alpha = 0.05 under H0: p = 0.67 (corresponds to VE = 0).

Placebo Cases	Active Cases	Total Cases	Proportion of Cases in Active Group	One-sided Lower Exact 95% CI
0	8	8	1.000	0.688
1	12	13	0.923	0.684
2	15	17	0.882	0.674
3	18	21	0.857	0.671
4	21	25	0.840	0.670
5	24	29	0.828	0.671
6	27	33	0.818	0.672
7	30	37	0.811	0.674
8	32	40	0.800	0.668
9	35	44	0.795	0.670
10	38	48	0.792	0.672
11	40	51	0.784	0.668
12	43	55	0.782	0.671
13	46	59	0.780	0.673
14	48	62	0.774	0.670
15	51	66	0.773	0.672
16	53	69	0.768	0.669
17	56	73	0.767	0.671
18	58	76	0.763	0.669
19	61	80	0.763	0.671
20	63	83	0.759	0.669
21	65	86	0.756	0.668
22	68	90	0.756	0.670
23	70	93	0.753	0.668
24	73	97	0.753	0.670
25	75	100	0.750	0.669
26	78	104	0.750	0.670
27	80	107	0.748	0.669
28	82	110	0.745	0.668
29	85	114	0.746	0.670
30	87	117	0.744	0.669
31	89	120	0.742	0.668
32	92	124	0.742	0.669
33	94	127	0.740	0.668

Placebo Cases	Active Cases	Total Cases	Proportion of Cases in Active Group	One-sided Lower Exact 95% CI
34	97	131	0.740	0.670
35	99	134	0.739	0.669
36	101	137	0.737	0.668
37	104	141	0.738	0.670

Appendix 4: FDA Toxicity Grading Scale for Local and General Systemic Reactogenicity

**Local Reaction to Injectable Product**				
**Clinical Abnormality**	**Mild (Grade 1)**	**Moderate (Grade 2)**	**Severe (Grade 3)**	**Potentially Life Threatening (Grade 4)**
Pain	Does not interfere with activity	Repeated use of nonnarcotic pain reliever >24 hours or interferes with activity	Any use of narcotic pain reliever or prevents daily activity	ER visit or hospitalization
Tenderness	Mild discomfort to touch	Discomfort with movement	Significant discomfort at rest	ER visit or hospitalization
Erythema/redness[a]	2.5 – 5 cm	5.1 – 10 cm	> 10 cm	For all participants: Necrosis or exfoliative dermatitis
Induration/swelling[b]	2.5 – 5 cm and does not interfere with activity	5.1 – 10 cm or interferes with activity	> 10 cm or prevents daily activity	For all participants: Necrosis
**Systemic (General)**				
**Clinical Abnormality**	**Mild (Grade 1)**	**Moderate (Grade 2)**	**Severe (Grade 3)**	**Potentially Life Threatening (Grade 4)**
Fever (°C)<br>(°F)	38.0 – 38.4<br>100.4 – 101.1	38.5 – 38.9<br>101.2 – 102.0	39.0 – 40<br>102.1 – 104	> 40<br>> 104
Nausea/vomiting	No interference with activity or 1 – 2 episodes/24 hours	Some interference with activity or >2 episodes/24 hours	Prevents daily activity, or requires outpatient IV hydration	ER visit or hospitalization for hypotensive shock
Headache	No interference with activity	Repeated use of nonnarcotic pain reliever >24 hours or some interference with activity	Significant; any use of narcotic pain reliever or prevents daily activity	ER visit or hospitalization
Fatigue/Malaise	No interference with activity	Some interference with activity	Significant; prevents daily activity	ER visit or hospitalization
Myalgia	No interference with activity	Some interference with activity	Significant; prevents daily activity	ER visit or hospitalization
Arthralgia	No interference with activity	Some interference with activity	Significant; prevents daily activity	ER visit or hospitalization

Abbreviations: DHHS = Department of Health and Human Services; ER = emergency room; FDA = United States Food and Drug Administration.

[a] In addition to grading the measured local reaction at the greatest single diameter, the measurement should be recorded as a continuous variable.

[b] Induration/swelling should be evaluated and graded using the functional scale as well as the actual measurement.

[c] Oral temperature; no recent hot or cold beverages.

Source: [DHHS 2007]. Reactogenicity table applies to both adults (≥18 years) and adolescents (12 to <18 years).

**Protocol Title:**	A Phase 3, Randomized, Observer-Blinded, Placebo-Controlled Study to Evaluate the Efficacy, Safety, and Immunogenicity of a SARS-CoV-2 Recombinant Spike Protein Nanoparticle Vaccine (SARS-CoV-2 rS) with Matrix-M1™ Adjuvant in Adult Participants ≥ 18 years with a Pediatric Expansion in Adolescents (12 to <18 Years)
**Protocol Number:**	2019nCoV-301
**Protocol Version, Date:**	Version 9.0; May 14, 2021
**ICON ID:**	2088-0040
**Document Version, Date:**	Final 4.0; October 01, 2021
**Prepared by:**	[redacted]
**On behalf of:**	Novavax, Inc.<br>21 Firstfield Road<br>Gaithersburg, MD 20878<br>United States

Confidentiality statement:

- The information provided in this document is strictly confidential.
- The recipients of the SAP must not disclose the confidential information contained within this document or any related information to other persons without the permission of the sponsor.
- In addition, the recipient of the SAP must keep this confidential document in a controlled environment which prevents unauthorized access to the document.

DocUUID : d222bbbe-1732-48af-b4cf-1e79e47eea3b

Signature Page

Prepared at ICON Clinical Research by:

[redacted]

Signature	Date

Reviewed at ICON Clinical Research by:

[redacted]

Signature	Date

Approved at SPONSOR by:

[redacted]

Signature	Date

DocUUID : d222bbbe-1732-48af-b4cf-1e79e47eea3b

Revision History

Version	Date	Revisions
0.1	12-April-2021	Initial draft
1.0	27-April-2021	Final Version with input from Sponsor
1.1	24-May-2021	To align with Protocol Version 9.0
2.0	26-May-2021	Updated after Sponsor review
2.1	22-September-2021	Updated after Sponsor review and removal of GPHS
3.0	28-September-2021	Updated after Sponsor review
4.0	01-October-2021	Final Version

Contents

1. Introduction 8

2. Planned Analysis 8

2.1. Interim Analyses 8
2.2. Final Analyses 8

3. Study Objectives 8

3.1. Primary objectives 8
3.2. Secondary objectives 9
3.3. Exploratory objectives 10

4. Study Design 10

4.1. Overview and Key Features 10
4.2. Randomization and blinding 11
4.3. Study treatments and assessments 12

5. Study Endpoints 19

5.1. Primary endpoints 19

5.1.1. Safety Endpoints: 20

5.1.2. Effectiveness Endpoint 21

5.2. Secondary Endpoints 21
5.3. Exploratory Endpoints 22

6. Sample Size and Power 22

7. Analysis Sets 24

7.1. Intention-To-Treat (ITT) analysis set 24
7.2. Full Analysis Set (FAS) 24
7.3. Safety (Safety) Analysis set 24
7.4. Per-Protocol Efficacy (PP-EFF) 24
7.5. Per-Protocol Efficacy Analysis Set 2 (PP-EFF-2) 25
7.6. Per-Protocol Immunogenicity (PP-IMM) analysis sets 25
7.7. Protocol deviations/violations and exclusions from analysis sets 26

8. Statistical Considerations and Analysis 26

8.1. Endpoint Review Committee for Severe Cases 26
8.2. Derived Variables 27
8.3. Handling of missing data and outliers 29

8.3.1. Missing data analysis methods 29

9. Statistical Methods 30

9.1. General statistical conventions 30

DocUUID : d222bbbe-1732-48af-b4cf-1e79e47eea3b

9.2. Participant disposition 31
9.3. Protocol deviations 32
9.4. Demographics and baseline characteristics 32
9.4.1. Demographics 32
9.4.2. Baseline and disease characteristics 32
9.4.3. Medical history 32
9.4.4. Prior and concomitant medications 33
9.5. Extent of exposure 33
9.5.1. Treatment duration 33
9.5.2. Treatment compliance 33
9.6. Efficacy Analyses 34
9.6.1. Analysis methods 34
9.6.2. Analysis of primary efficacy endpoint(s) 36
9.6.3. Analysis of secondary efficacy and immunogenicity endpoints 37
9.7. Analysis of exploratory endpoint(s) 39
9.8. Safety Analysis 39
9.8.1. Adverse events 40
9.8.2. Vital signs 41
9.8.3. Physical examinations 41
9.8.4. Electrocardiograms 41
9.9. Subgroup Analysis 41
10. Changes to Planned Analysis from Study Protocol 42
11. References 42
12. Appendices 44
12.1. Appendix 1: Baseline Comorbidity Mappings and Medical Subgroupings to MedDRA terms 44
12.1.1. Preferred Terms 44
12.1.2. Higher Level Group Terms 49

DocUUID : d222bbbe-1732-48af-b4cf-1e79e47eea3b

List of Abbreviations

Abbreviation	Meaning
AE	Adverse Event
AESI	Adverse Event of Special Interest
AMI	Acute Myocardial Infarction
ARDS	Acute Respiratory Distress Syndrome
ATC	Anatomical Therapeutic Chemical
BiPAP	Bilevel positive Airway Pressure
BMI	Body Mass Index
BP	Blood Pressure
CI	Confidence Interval
CMI	Cell-Mediated Immunity
CONSORT	Consolidated Standards of Reporting Trials
COVID	Corona Virus Disease
CoVPN	COVID-19 Prevention Network
CPAP	Continuous Positive Airway Pressure
CPH	Cox Proportional Hazard
CSR	Clinical Study Report
CT	Computerized Tomography
DBP	Diastolic Blood Pressure
DSMB	Data and Safety Monitoring Board
DVT	Deep Vein Thrombosis
ECMO	Extracorporeal Membrane Oxygenation
ELISA	Enzyme-linked Immunosorbent Assay
EU	European Union
FAS	Full Analysis Set
FDA	Food and Drug Administration
FLU-Pro	InFLUenza Patient-Reported Outcome
GCP	Good Clinical Practice
GMEU	Geometric Mean ELISA Units
GMFR	Ratio of GMEUs
GMT	Geometric Mean Titers
H0	Null Hypothesis
hACE2	human Angiotensin-Converting Enzyme 2
HIV	Human Immunodeficiency Virus
ICF	Informed Consent Form
ICH	International Council for Harmonisation of Technical Requirements for Pharmaceuticals for Human Use
ICU	Intensive Care Unit
ID	Identification
IFN	Interferon
IL	interleukin
IRT	Interactive Response Technology
ITT	Intent-to-treat
LN	Natural Logarithm
LRTI	Lower Respiratory Tract Infection
MAAE	Medically Attended Adverse Event
MedDRA	Medical Dictionary for Regulatory Affairs

DocUUID : d222bbbe-1732-48af-b4cf-1e79e47eea3b

Abbreviation	Meaning
MIS-C	Multisystem Inflammatory Syndrome in Children
MLE	Maximum Likelihood Estimator
MN	Microneutralization
NIAID	National Institute of Allergy and Infectious Diseases
NIH	National Institutes of Health
NIPPV	Non-invasive Positive Pressure Ventilation
NIV	Non-invasive Ventilation
NP	Nucleoprotein
O_2	Oxygen
OWS	Operation Warp Speed
PBMC	Peripheral Blood Mononuclear Cell
PCR	Polymerase Chain Reaction
PE	Pulmonary Embolism
PI	Principal Investigator
PIMMC	Potential Immune-mediated Medical Conditions
PP-EFF	Per-protocol Efficacy
PP-IMM	Per-protocol Immunogenicity
PT	Preferred Term
RNA	Ribonucleic Acid
SAE	Serious Adverse Event
SAP	Statistical Analysis Plan
SARS-CoV-2	Severe Acute Respiratory Syndrome Coronavirus 2
SAS	Statistical Analysis Software
SCR	Seroconversion Rate
SBP	Systolic Blood Pressure
SD	Standard Deviation
SOC	System Organ Class
SSG	Statistical Support Group
TEAE	Treatment Emergent Adverse Event
TLF	Table, Listing, and Figure
TNF	Tumor Necrosis Factor
TTE	Time to Event
US	United States
VoC	Variant of Interest
VoI	Variant of Concern
VE	Vaccine Efficacy

1. Introduction

The purpose of this Statistical Analysis Plan (SAP) is to provide detailed descriptions of the statistical methods, data derivations, and data displays regarding the final analysis for the study protocol 2019nCoV-301 version 9.0 “A Phase 3, Randomized, Observer-Blinded, Placebo-Controlled Study to Evaluate the Efficacy, Safety, and Immunogenicity of a SARS-CoV-2 Recombinant Spike Protein Nanoparticle Vaccine (SARS-CoV-2 rS) with Matrix-M1™ Adjuvant in Adult Participants ≥ 18 years with a Pediatric Expansion in Adolescents (12 to <18 Years) “ dated May 14, 2021. The table of contents and templates for the TLFs will be produced in a separate document.

Beginning with version 6.0 of the protocol, the study consists of an Adult Main Study and a Pediatric Expansion. In version 8.0, the School-Age Children (6-11 Years) are taken out the study. Version 9.0 reflects further changes subsequent to feedback from the FDA Center for Biologics Evaluation and Research (CBER). This SAP will only cover the Pediatric Expansion Study, with a separate SAP for the Adult Main Study conducted in conjunction with Version 9.0 of the protocol.

Any deviations from this SAP will be described and justified in the Clinical Study Report (CSR).

The preparation of this SAP has been based on International Conference on Harmonisation (ICH) E9 Statistical Principles for Clinical Trials and E6 Good Clinical Practice (GCP) guidelines.

All data analyses and generation of TLFs will be performed using SAS 9.4® or higher.

2. Planned Analysis

2.1. Interim Analyses

There are no interim analyses that require adjustment to Type I error.

2.2. Final Analyses

Analyses planned before end of study include a single analysis of primary and secondary efficacy endpoints at a cutoff point after initiation of the blinded crossover when participant data up to the crossover or cutoff date (whichever is earlier) has been accumulated and cleaned for all subjects. In addition, an analysis of safety and immunogenicity at Day 35 for adolescents (or at a later time point) may be conducted for the Pediatric Expansion. After Month 12, an analysis will be performed to examine durability of the vaccine on efficacy endpoints as described in the next section. The analysis at Month 12 will also report on safety follow-up through 1 year.

3. Study Objectives

3.1. Primary objectives

- To evaluate the efficacy of a 2-dose regimen of SARS-CoV-2 rS adjuvanted with Matrix-M1 compared to placebo against polymerase chain reaction (PCR)-confirmed symptomatic coronavirus disease 2019 (COVID-19) illness diagnosed ≥ 7 days after completion of the second injection in the initial set of vaccinations of adolescent participants 12 to <18 years of age years of age.

- To describe the safety experience for the vaccine versus placebo in adolescent participants (12 to <18 years of age) based on solicited short-term reactogenicity by toxicity grade for 7 days following each vaccination (Days 0 and 21) after the initial set of vaccinations.

- To assess overall safety through 49 days (28 days after second injection of each set of vaccinations [initial and crossover]) by comparing vaccine versus placebo for all unsolicited AEs and MAAEs.

- To assess the frequency and severity of MAAEs attributed to vaccine, AESIs, or SAEs through the EoS and to compare vaccine versus placebo after each set of vaccinations (initial and crossover).

- To assess all-cause mortality in vaccine versus placebo recipients after each set of vaccinations (initial and crossover).

- To assess non-inferiority of the neutralizing antibody response for all adolescent participants seronegative to anti-SARS-CoV-2 NP antibodies at baseline, compared with that observed in seronegative adult participants 18 to < 26 years of age from the Adult Main Study (Immunogenicity Population participants before crossover).

3.2. Secondary objectives

- To evaluate the efficacy of a 2-dose regimen ofSARS-CoV-2 rS adjuvanted with Matrix-M1compared to placebo against PCR-confirmedsymptomatic COVID-19 illness due to SARS- CoV-2 variant not considered as a "variant of concern / interest" according to the CDC Variants Classification, diagnosed ≥ 7 days aftercompletion of the second injection in the initial set of vaccinations of adolescent participants 12to <18 years of age.

- To evaluate the efficacy of a 2-dose regimen ofSARS-CoV-2 rS adjuvanted with Matrix-M1compared to placebo against PCR-confirmedmoderate-to-severely symptomatic COVID-19 illness diagnosed ≥ 7 days after completion of the second vaccination in the initial set of vaccinations of adolescent participants 12 to <18 years of age.

- To assess VE against ANY symptomatic SARS-CoV-2 infection.

- To assess VE according to race and ethnicity.

- To assess the durability of VE (measured by all defined efficacy endpoints) in adolescents after initial active vaccine recipients versus crossover (delayed) active vaccine recipients.

- To monitor occurrence and severity of COVID-19 cases by following participant-reported symptoms.

- To assess the neutralizing antibody response to SARS-CoV-2 for adolescent participants by subsets with and without anti-NP antibodies at baseline, compared with that observed in adult participants 18 to <26 years of age from the **Adult Main Study** (Immunogenicity Populationparticipants before crossover).

- To assess the anti-spike immunoglobulin G (IgG) antibody response and human angiotensin converting enzyme 2 (hACE2) inhibiting antibody response at Day 35 for adolescent participants by subsets with and without detectable anti-NP antibodies at baseline, compared with that observed in

DocUUID : d222bbbe-1732-48af-b4cf-1e79e47eea3b

adult participants 18 to <26 years of age from the **Adult Main Study** (Immunogenicity Population participants before crossover).

- To assess the durability of immune response (anti-rS IgG antibody, hACE2 inhibition, and microneutralization [MN] titers) at 12, 18 and 24 months of study in all adolescent participants, and for subsets with and without detectable anti-NP antibodies at baseline or prior to crossover set of vaccinations, with that observed in adult participants 18 to <26 years of age from the Adult Main Study (Immunogenicity Population participants before crossover).

- To assess the proportion of adolescent participants (vaccine versus placebo recipients) with SARS-CoV-2 infection determined by anti-SARS-CoV-2 NP antibodies, including specifically asymptomatic infection, across the 2 years of study follow-up.

- To assess the VE against SARS-CoV-2 infection determined by anti-SARS-CoV-2 NP antibodies in adolescent participants, regardless of whether the infection was symptomatic.

3.3. Exploratory objectives

- To evaluate the efficacy of study vaccine compared to placebo against PCR-confirmed symptomatic COVID-19 illness due to a SARS-CoV-2 variant considered as a "variant of concern / interest" according to the CDC Variants Classification, diagnosed ≥ 7 days after completion of the second vaccination in the initial set of vaccinations of adolescent participants 12 to < 18 years of age.

- To assess cell-mediated response in a cohort of 50 adolescent participants at baseline and at Days 7 and 28: Type 1 T helper (Th1) or Type 2 T helper (Th2) predominance.

- To assess impact of vaccination on nasal viral load in nasal swabs of adolescent participants who develop symptoms of possible COVID-19.

- To assess impact of vaccination on asymptomatic SARS-CoV-2 PCR positivity and viral load in adolescent participants at the time of the crossover set of vaccinations.

- To describe sequences of the genetic material from SARS-CoV-2 viruses detected in COVID-19 cases in adolescent participants to evaluate possible viral mutations that may be associated with breakthrough infections.

4. Study Design

4.1. Overview and Key Features

The Pediatric Expansion Study is part of the 2019nCoV-301 phase 3, multicenter, multinational, randomized, observer-blinded, placebo-controlled clinical study that will evaluate the efficacy, safety, and immunogenicity of a SARS-CoV-2 vaccine in adolescent participants (12- <18 years of age). Participants will then be randomized in a 2:1 ratio via block randomization to receive 2 IM injections of SARS-CoV-2 rS + Matrix-M1 adjuvant or normal saline placebo as described in Table 1.

DocUUID : d222bbbe-1732-48af-b4cf-1e79e47eea3b

Table 1 Pediatric Expansion - Trial Vaccine Groups

Trial Vaccine Group	Estimated Number of Randomized Participants	Vaccination Periods			
		Initial		Crossover[1]	
		2 Vaccinations		2 Vaccinations	
		Day 0	Day 21 (+ 7 days)	Day 0	Day 21 (+ 7 days)
SARS-CoV-2 rS (5 µg) + Matrix-M1 adjuvant (50 µg)	12-<18 years: 2,000	Active vaccine	Active vaccine	Placebo	Placebo
Placebo (normal saline)	12-<18 years: 1,000	Placebo	Placebo	Active vaccine	Active vaccine

1. Crossover will occur ~6 months after the completion of the initial set of vaccinations.

Participants in the Pediatric Expansion will be enrolled in the US only. The full enrollment will be contingent upon the review of the early safety data (i.e., 7 days of reactogencity and overall safety post-dose 1). Among the first approximately 60 sentinel adolescents (15 to <18 years of age, N=~30, and 12 to <15 years of age, N=~30) enrolled will be dosed with active vaccine or placebo and data will be collected through Day 7 before the remainder of the planned adolescent participants (N = ~2,940) are enrolled. Likewise, administration of the second vaccine dose to the full participant population will be contingent upon the review of early safety data (ie, 7 days of reactogenicity and overall safety post-dose 2) in the first ~60 enrolled adolescents before dosing the remainder of the adolescent participants. Safety data will be provided to and reviewed by the Data and Safety Monitoring Board (DSMB) after each dose of the early safety data review period (ie 7 days after each vaccination in the sentinel group). Simultaneously, safety data will be reviewed internally by the Sponsor.

After approximately 6 months of follow-up after the completion of the initial set of vaccinations (or earlier depending upon feasibility, EUA or otherwise licensed vaccine availability and the dynamics of the pandemic), pediatric participants will be scheduled for administration of another set of vaccinations with 2 injections of the alternate study material 21 days apart (“blinded crossover”). That is, the initial recipients of placebo prior to crossover will then receive SARS-CoV-2 rS with Matrix-M1 adjuvant and initial recipients of active vaccine of SARS-CoV-2 rS with Matrix-M1 adjuvant will receive placebo of normal saline. The same procedure for vaccine administration followed for the initial set of vaccinations will be followed at the time of the blinded crossover to ensure that the integrity of the blinded study is maintained.

4.2. Randomization and blinding

At each clinical site, participants who are eligible to enter the double-blind treatment period will be randomized to initially receive 2 IM injections of 5 µg SARS-CoV-2 rSadjuvanted with 50 µg Matrix-M1 or

normal saline placebo on Days 0 and 21 + 7 days. in a 2:1 ratio via block, via the FlexAdvantage Interactive Response Technology (IRT) system. Randomization will follow a permuted block randomization method where the block size is allowed to vary randomly. No stratification by site will be conducted, however, at the time of randomization of a participant at a site, a full block will be assigned to the site in order to maintain treatment assignment balance in the planned ratio at each site and allow for site and region effects to be assessed. For the blinded crossover part of the study, subjects initially randomized to placebo will be scheduled to receive SARS-CoV-2 rS with Matrix-M1 adjuvant and subjects initially randomized to SARS-CoV-2 rS with Matrix-M1 adjuvant will be scheduled to receive placebo. Handling of mis-dosed and mixed-dose during the initial vaccination period will be done by the clinical and site team in order to avoid overdosing subjects with study product.Study staff at each site (coordinators and PI) along with the participants, and sponsor representatives will be blinded throughout the course of the trial. The sponsor will be blinded up to the time of analysis of the initial treatment period. After the analysis the sponsor will be unblinded to facilitate regulatory submissions. Unblinded personnel at ICON are designated as per the document “STUDY SPECIFIC PROCEDURE FOR UNBLINDING PLAN PRIOR TO DATABASE RELEASE” maintained by ICON.

Formal unblinding of the trial will be done after database lock and after identification and approval of the study analysis sets. Emergency unblinding procedures may be taken if the knowledge of the product administered is essential for the medical management of the participant by the principal investigator. Additionally, unblinding by the study site may be conducted if the participant is eligible for and desires to obtain an Emergency Use Authorized (EUA) or otherwise licensed vaccine. Individual, emergency participant unblinding will be conducted through the IRT system. Notification to sponsor and medical monitor will be made for each unblinding and a record will be kept at each study site for all broken treatment codes and the reason for the need to break the treatment assignment. This will also be documented in the IRT system and in the clinical database.

4.3. Study treatments and assessments

A full vaccination will consist of two, 0.5 mL intramuscular injections at Day 0 and Day 21 (+ 7 days) and again during the crossover portion of the trial (the second injection 21 + 7 days after the first in the series). The trial vaccine of 0.5 mL includes active ingredients of 5 µg SARS-CoV-2 rS adjuvanted with 50 µg Matrix-M1.

The total study duration is approximately 25 months consisting of 4 periods:

- Screening period starting up to 7 days prior to initial injection up to the point of initial injection. (maximum of 7 days).
- Double-blind treatment period starting at initial injection through the day prior to first crossover injection; or Month 12 if no crossover doses received.

DocUUID : d222bbbe-1732-48af-b4cf-1e79e47eea3b

- Double-blind crossover treatment period beginning the day of first crossover injection through month 12.
- Long-term follow-up period beginning at month 12 through month 24.

The pediatric participants who test positive for COVID-19 anti-NP antibodies and/or PCR-positive nasal swab at baseline, indicating previous and/or current SARS-CoV-2 infection, will have subsequent SARS-CoV-2 S protein immune responses analyzed but will not contribute to the primary efficacy/effectiveness analyses.

Those who test positive (PCR or anti-NP) immediately prior to the crossover vaccination series may contribute to the immunogenicity analyses at Months 12, 18 and 24. Immediate immunogenicity analyses will not be performed subsequent to the crossover vaccination period.

Results from the anti-NP positive and/or PCR-positive nasal swab participants prior to either sets of vaccinations will be assessed and reported separately.

The whole blood samples for PBMC will be collected at Days 0, 7 and 28 from a small subset comprised of 50 adolescent participants enrolled at selected study site(s) with the capacityto isolate PBMCs. These cells will be evaluated for cell-mediated immune responses to the initial set of vaccinations.

Active surveillance for COVID-19 illness will be conducted through the first 12 months of the study. Parent(s)/caregiver(s) of participants will be asked to report symptoms of COVID-19 to the site as soon as possible after symptoms onset, or during the weekly remote contact. Fever and other symptoms of COVID-19 (including date of onset, duration, etc.) will be collected in a paper memory aid and will be reported to the sites by the parent(s)/caregiver(s) either as a spontaneous phone call or during the weekly remote contact during the first 12 months. If the parent(s)/caregiver(s) report symptoms compatible with COVID-19 by spontaneous contact or during the weekly contact, the study site will schedule an in-person Unscheduled Acute Illness Visit. During the first 4 days after vaccination when solicited systemic reactogenicity symptoms may be similar to those of COVID-19, investigators are directed to use their clinical judgement to decide if an Unscheduled Acute Illness visit is warranted. Active surveillance for COVID-19 will continue after the blinded crossover through the first 12 months of study. Passive surveillance of safety and efficacy via remote contacts or the scheduled visits will continue during Months 12 to 24.

Overall safety assessments from Day 0 through the first 12 months of follow-up after the initial set of vaccinations will include parent(s)/caregiver(s)-recorded solicited (local and systemic reactogenicity) events through 7 days following each injection in the initial set of vaccinations collected via eDiary. Unsolicited AEs and MAAEs will be collected through 49 days, ie, 28 days after second injection of the

initial and crossover sets of vaccinations and MAAEs attributed to vaccine, AESIs, and SAEs will be recorded by the parent/caregiver on the memory aid and reported to the sites by weekly remote contact with the parent(s)/caregiver(s) through Month 12, and every 3 months via remote contacts (ie, Months 15 and 21) or during the scheduled visits (ie, Months 12, 18, and 24) during the second year (total of 24 months).

Blood samples for serologic assessments (anti-NP antibodies, IgG antibody to SARS-CoV-2 S protein, MN, and hACE2 inhibition) will be collected from all pediatric participants before the initial set of vaccinations and at selected subsequent time points thereafter, including immediately prior to the crossover set of vaccinations.

Immune responses immediately following vaccinations will be obtained from all participants after the initial set of vaccinations but not immediately after the crossover vaccinations (Visit C2).

Persistence of the immune responses will be measured at 12, 18, and 24 months after the initial set of vaccinations. Testing will be performed on all adolescent participants for IgG and hACE2 but only a randomly selected set of participants will be tested for MN to support the effectiveness endpoint.

Participants unblinded between the first (Day 0) and second (Day 21) dose of trial vaccine will not be eligible to receive further investigational product on this protocol. Similarly, participants who choose to withdraw from the blinded trial after having received both doses of blinded study vaccine will not be eligible for blinded crossover. Receipt of another COVID-19 vaccine (under EUA or otherwise licensed) and date of administration should be recorded as a concomitant medication and participant censored at the time of unblinding for the purpose of receiving another COVID-19 vaccine.

A detailed description of procedures and assessments to be conducted during this study is summarized in the Schedule of Study Assessments in Table 2 below.

DocUUID : d222bbbe-1732-48af-b4cf-1e79e47eea3b

Table 2 Schedule of Assessments in the Pediatric Expansion

Study Period:	Screening Period	Initial Vaccination Period								Crossover Vaccination period		Regular remote contact	Unscheduled visit[4]			Months Following Initial Vaccination		
													Acute Illness[3]	Convalescent[4]	General[5]			
**Study Day:**	–7 to 0	0[1]	7	14	21	28	35	42	49	C1	C2	56-M24[21]	–	–	–	12[21]	18[21]	24[21]
**Window (days)[2]:**	–	0	+ 3	+ 3	+ 7	+3	+ 7	+ 3	+ 7	+ 7	+ 7	+3	–	–	–	± 15	± 30	± 30
**Minimum Days Following Most Recent Vaccination[2]:**	–	0	–	–	21	–	14	–	28	180	21	–	–	–	–	360	540	720
**Study Visit:**	Screening	1	2	PC	3	4	5	PC	6	7	8	Phone call	Acute Illness[22]	Convalescent[22]	General[22]	9	10	EoS[6, 22]
Informed consent	X																	
Medical and social history[7]	X																	
Inclusion/exclusion criteria	X	X[8,9]			X[8,9]													
Demographics[10]	X																	
Prior/concomitant medications		X[8,9]	X	X	X[8,9]	X	X	X	X	X[8,9]	X[9]	X[11]	X	X	X	X[11]	X[11]	X[11]
Vital sign measurements (including body temperature)	X	X[12]			X[12]					X[12]	X[12]		X					
Urine pregnancy test[13]	X	X[9]			X[9]					X[9]	X[9]							
Targeted physical examination[14]	X	X[9]			X[9]		X			X[9]	X		X	X	X	X		
Randomization		X																
Blood sampling for SARS-CoV-2 (anti-NP) antibodies		X[9]					X			X[9]			X	X	X	X	X	X
Vaccination		X			X					X	X							
Reactogenicity[15,16]		X			X					X	X							
Blood sampling for SARS-CoV-2 vaccine immunogenicity (IgG antibody to SARS-CoV-2 S protein, MN, hACE2 inhibition)		X[9]			X[9]		X			X[9]			X	X		X	X	X
Blood sampling for whole blood, CMI[17]		X[9]	X			X[9]												
Safety follow-up phone call			X	X		X		X	X			X						

Study Period:	Screening Period	Initial Vaccination Period								Crossover Vaccination period		Regular remote contact	Unscheduled visit[4]			Months Following Initial Vaccination		
													Acute Illness[3]	Convalescent[4]	General[5]			
**Study Day:**	–7 to 0	0[1]	7	14	21	28	35	42	49	C1	C2	56-M24[21]	–	–	–	12[21]	18[21]	24[21]
**Window (days)[2]:**	–	0	+ 3	+ 3	+ 7	+3	+ 7	+ 3	+ 7	+ 7	+ 7	+3	–	–	–	± 15	± 30	± 30
**Minimum Days Following Most Recent Vaccination[2]:**	–	0	–	–	21	–	14	–	28	180	21	–	–	–	–	360	540	720
**Study Visit:**	Screening	1	2	PC	3	4	5	PC	6	7	8	Phone call	Acute Illness[22]	Convalescent[22]	General[22]	9	10	EoS[6,22]
Monitoring for COVID-19 illness[18]		From 4 days after initial vaccination, collected in paper memory aid and reported to the sites by the parent(s)/caregiver(s) via remote contact																
Nasal swab(s) at clinic – anterior nares[3]		X[9]								X[9]			X					
All unsolicited AEs since prior visit[23]		X	X	X	X	X	X	X	X	X	X	X	X	X	X			
All MAAEs[23]		X	X	X	X	X	X	X	X	X	X	X	X	X	X			
Any MAAE attributed to vaccine		X	X	X	X	X	X	X	X	X	X	X	X	X	X	X	X	X
SAEs	X	X	X	X	X	X	X	X	X	X	X	X	X	X	X	X	X	X
AESI[19]	X	X	X	X	X	X	X	X	X	X	X	X	X	X	X	X	X	X
Endpoint Review form[4]														X				
EoS form[20]																		X

Abbreviations: AE = adverse event; AESI = adverse event(s) of special interest; BMI = body mass index; BP = blood pressure; C1 = first vaccination visit of the crossover vaccination period; C2 = second vaccination visit of the crossover vaccination period; CMI = cell-mediated immunity; COVID-19 = coronavirus disease 2019; eDiary = electronic patient-reported outcome diary application; EoS = end of study; EUA = Emergency Use Authorization; FDA = United States Food and Drug Administration; hACE2 = human angiotensin-converting enzyme 2; IgG = immunoglobulin G; M24 = Month 24; MAAE = medically attended adverse event; MN = microneutralization; NP = nucleoprotein; O_2 = oxygen; PBMC = peripheral blood mononuclear cells; PC = phone call; PCR = polymerase chain reaction; PIMMC = potential immune-mediated medical conditions; S = spike; SAE = serious adverse event; SARS-CoV-2 = severe acute respiratory syndrome coronavirus 2.

1. If screening and randomization occur on the same day (ie, Day 0), study visit procedures should not be duplicated.
2. Days relative to vaccination are only estimates because the window allowance is not inclusive. Should a study pause occur, visits/windows will be adjusted to allow participants to continue without protocol deviation. Visit schedules following the vaccinations are calculated relative to the day the vaccinations were received. The first blinded crossover visits (C1) will be conducted after approximately 6 months (or earlier depending upon feasibility, EUA vaccine availability for this age group, and the dynamics of the pandemic) of follow-up after completion of the initial set of vaccinations has occurred. For Months 12, 18 and 24 visits, “Minimum Days Following Most Recent Vaccination” refers to initial vaccination 2 (Day 21).
3. At the in-person Unscheduled Acute Illness Visit, participants will be queried regarding AE symptoms, concomitant medications taken for these symptoms, undergo a targeted physical examination (to include O_2 saturation and respiratory rate), if indicated by signs and symptoms, and have obtained by the study personnel a medically attended nasal swab, and a blood sample for serologic testing.

4. Study participants whose medically attended nasal swabs are confirmed to be PCR-positive for SARS-CoV-2 at the Unscheduled Acute Illness Visit will be contacted by the study site to arrange an Unscheduled Convalescent Visit. The Unscheduled Convalescent Visit will occur approximately 1 month (or as soon thereafter, as feasible) after the onset of the PCR-confirmed case of COVID-19 at the Unscheduled Acute Illness Visit to assess status of AEs, record the clinical course of the disease on the Endpoint Form and obtain a blood sample for convalescent serologic testing.
5. An Unscheduled General Visit will be conducted by study personnel in the event of a general medical issue other than COVID-19 symptomatology.
6. EoS visit. Should participants decide to terminate early, an EoS telephone visit will occur to collect the maximum safety data and blood sample, if possible.
7. Including prior and concomitant medical conditions, recent vaccinations (≤ 90 days), and significant surgical procedures.
8. Should participants start specific medications or have specific diagnoses that would have been exclusionary at baseline, consultation with the ICON Medical Monitor or Sponsor is required.
9. Performed prior to each vaccination. There are 7 scheduled blood draws for immunogenicity assessments at the Day 0, 21, 35, C1 (Day 180), and Months 12, 18, and 24 visits, and during unscheduled visits (ie Acute illness, Convalescent, and General). For participants in the CMI cohort only (N=50), additional blood will be collected to obtain PBMCs at the Day 0, 7 and 28 visits.
10. Screening only. Including date of birth (day, month, and year), sex, race, ethnicity, weight, height, and BMI (derived).
11. Only those medications associated with any MAAE attributed to vaccine, potential AESI, or SAE will be recorded. For day of second dose, recording is prior to each vaccination.
12. On vaccination days, vital sign measurements will be collected once before vaccination to ensure participant has controlled BP and heart rate and no evidence of fever prior to vaccination.
13. For participants of childbearing potential (excluding participants who are premenarche or surgically sterile), a urine pregnancy test will be performed at screening and prior to each vaccination. A positive urine pregnancy test at any of the vaccination visits will result in the participant not receiving any further vaccination.
14. Examination at screening to include height and weight. Targeted physical examination after Day 21 is optional, as needed for AE evaluation, except for vaccination visits where it is mandatory.
15. On vaccination days (either at initial set of vaccinations or at the crossover set of vaccinations), participants will remain in clinic for at least 30 minutes to be monitored for any immediate reaction. Any immediate reaction will be noted as AEs on day of vaccination.
16. Parent(s)/caregiver(s) of the participants will utilize an eDiary to record reactogenicity following the initial set of vaccinations. Parent(s)/caregiver(s) of the participants will record reactogenicity starting on the same day of the vaccinations and for an additional 6 days (not counting vaccination day). Parent(s)/caregiver(s) of the participants will be provided with a thermometer and instructed to monitor the participant body temperature daily throughout the reactogenicity period and to record temperature and relevant symptoms daily in their eDiary. Study site personnel will regularly review the eDiary for completeness. Should any reactogenicity event extend beyond 7 days after vaccination (toxicity grade ≥ 1), then it will be recorded as an AE with the same start date as the reactogenicity event and followed to resolution per FDA guidelines for dataset capture. 7-day reactogenicity will not be captured after the crossover set of vaccinations.
17. Subset of 50 adolescent participants enrolled at pre-identified study site(s) with the capability to process blood samples for PBMC.
18. Parent(s)/caregiver(s) of the participants will be instructed to report COVID-19 symptoms / diagnosis to the sites as soon as they are aware or at the weekly remote contact. Starting on Day 4, throughout the first 12 months of the study, when fever or other specified symptoms (see Table 5 in protocol for symptoms suggestive of COVID-19) are reported the study site to contact the participant to schedule the in-person Unscheduled Acute Illness Visit and medically-attended nasal swab.
19. AESI: To include PIMMC (listed in Appendix 2, Table 11 in protocol), or any newly identified complications of COVID-19 (listed in Appendix 2, Table 12 in protocol) or other potential AESI followed through 24 months after participants' initial set of vaccinations.
20. EoS form will be completed for all participants, including those who are terminated early.
21. From Day 0 to Month 12, study sites will perform weekly remote contacts (phone, email, text) with the participants' parent(s)/caregiver(s) to collect SAEs, MAAEs attributed to vaccine, AESIs or COVID-19 illness except during weeks in which an in-clinic visits is to be performed (eg, Day 21 or Day 35 visits), and every 3 months from Months 12-24 (ie, at Months 15 and 21), in addition to conducting in-person visits for blood draws at Month 12, 18 and 24 (ie, no remote contact needed when in-person visits are scheduled).

DocUUID : d222bbbe-1732-48af-b4cf-1e79e47eea3b

22. Participants that requested to be unblinded and/or are continuing in the study for Safety Follow-up will have visits replaced by remote contact (phone, email, text), at a minimum, every 90 days (± 30 days) from last dose received during the first year of participation. During the second year (ie, Months 12-24), contact will be continued every 180 days (± 30 days). **No eDiary, nasal swabs or blood samples will be collected during safety follow-up**. MAAEs, SAEs, AESI, and COVID- 19 diagnoses will be collected during the remote contacts. Receipt of another COVID-19 vaccine used under EUA should be recorded as a concomitant medication. The End of Study page is not to be completed for these participants until the end of their participation in the study.
23. Through 49 days, ie, 28 days after second injection of the initial and crossover sets of vaccinations.

DocUUID : d222bbbe-1732-48af-b4cf-1e79e47eea3b

5. Study Endpoints

5.1. Primary endpoints

First episode of PCR-positive mild, moderate, or severe COVID-19, where severity is defined as:

Mild COVID-19 (≥ 1 of the following):

- Fever (defined by subjective or objective measure, regardless of use of anti-pyretic medications)
- New onset cough
- ≥ 2 additional COVID-19 symptoms:
    - New onset or worsening of shortness of breath or difficulty breathing compared to baseline.
    - New onset fatigue.
    - New onset generalized muscle or body aches.
    - New onset headache.
    - New loss of taste or smell.
    - Acute onset of sore throat, congestion or runny nose.
    - New onset nausea, vomiting or diarrhea.

OR Moderate COVID-19 (≥ 1 of the following):

- High fever (≥ 38.4°C) for ≥ 3 days (regardless of use of anti-pyretic medications, need not be contiguous days).
- Any evidence of significant lower respiratory tract infection (LRTI):
    - Shortness of breath (or breathlessness or difficulty breathing) with or without exertion (greater than baseline).
    - Tachypnea: 24 to 29 breaths per minute at rest.
    - SpO_2: 94% to 95% on room air.
    - Abnormal chest X-ray or chest computerized tomography (CT) consistent with pneumonia or LRTI.
- Adventitious sounds on lung auscultation (eg, crackles/rales, wheeze, rhonchi, pleural rub, stridor).

OR Severe COVID-19 (≥ 1 of the following):

- Tachypnea: ≥ 30 breaths per minute at rest.
- Resting heart rate ≥ 125 beats per minute.
- SpO_2: ≤ 93% on room air or PaO_2/FiO_2 < 300 mmHg.
- High flow oxygen (O_2) therapy or non-invasive ventilation (NIV)/non-invasive positive pressure ventilation (NIPPV) (eg, continuous positive airway pressure [CPAP] or bilevel positive airway pressure [BiPAP]).
- Mechanical ventilation or extracorporeal membrane oxygenation (ECMO).
- One or more major organ system dysfunction or failure to be defined by diagnostic testing/clinical syndrome/interventions, including any of the following:
    - Acute respiratory failure, including acute respiratory distress syndrome (ARDS).

- Acute renal failure.
- Acute hepatic failure.
- Acute right or left heart failure.
- Septic or cardiogenic shock (with shock defined as systolic blood pressure [SBP] < 90 mm Hg OR diastolic blood pressure [DBP] < 60 mm Hg).
- Acute stroke (ischemic or hemorrhagic).
- Acute thrombotic event: acute myocardial infarction (AMI), deep vein thrombosis (DVT), pulmonary embolism (PE).
- Requirement for: vasopressors, systemic corticosteroids, or hemodialysis.

- Multisystem Inflammatory Syndrome in Children (MIS-C), as per the CDC definition:
  - An individual aged <21 years with fever(>38.0°C for ≥24 hours, or report of subjective fever lasting ≥24 hours), laboratory evidence of inflammation (including, but not limited to, one or more of the following: an elevated C-reactive protein (CRP), erythrocyte sedimentation rate (ESR), fibrinogen, procalcitonin, d-dimer, ferritin, lactic acid dehydrogenase (LDH), or interleukin 6 (IL-6), elevated neutrophils, reduced lymphocytes and low albumin), and evidence of clinically severeillness requiring hospitalization, with multisystem (>2) organ involvement (cardiac, renal, respiratory, hematologic, gastrointestinal, dermatologic or neurological); AND
  - No alternative plausible diagnoses; AND
  - Positive for current or recent SARS-CoV-2 infectionby RT-PCR, serology, or antigen test; or COVID-19 exposure within the 4 weeks prior to the onset ofsymptoms.
- Admission to an intensive care unit (ICU).
- Death.

5.1.1. Safety Endpoints:

- Reactogenicity incidence, duration and severity (mild, moderate or severe) recorded by parent(s)/caregiver(s) on their electronic patient-reported outcome diary application (eDiary) on days of vaccination and subsequent 6 days (total 7 days after each vaccineinjection in the initial set of vaccinations).
  - Reactogenicity endpoints include injection sitereactions:
    - Pain.
    - Tenderness.
    - Erythema.
    - Swelling/induration.
  - Systemic reactions:
    - Fever.
    - Malaise.
    - Fatigue.
    - Arthralgia.
    - Myalgia.
    - Headache.
    - Nausea/vomiting.
- Incidence and severity of MAAEs through 49 days ie, 28 days after second injection of each set of vaccinations (initial and crossover).
- Incidence and severity of unsolicited AEs through 49 days, ie, 28 days after second injection of each set of vaccinations (initial and crossover).
- Incidence and severity of MAAEs attributed to study vaccine, SAEs and AESIs through Month 12.
- Incidence and severity of SAEs (including COVID-19 diagnoses), MAAEs attributed to study vaccine and AESIs during Month 12 through Month 24 or the EoS.

DocUUID : d222bbbe-1732-48af-b4cf-1e79e47eea3b

- Death due to any cause.

5.1.2. Effectiveness Endpoint

Neutralizing antibody response at Day 35 for all adolescent participants seronegative to anti-SARS- CoV-2 NP antibodies at baseline, compared with that observed in seronegative adult participants 18 to <26 years of age from the **Adult Main Study** (Immunogenicity Population participants before crossover).

5.2. Secondary Endpoints

- First episode of PCR-positive COVID-19, as defined under the primary endpoint, shown by gene sequencing to represent a variant not considered as a “variant of concern / interest” according to the CDC Variants Classification[1].
- First episode of PCR-positive moderate or severe COVID-19 as defined under the primary endpoint.
- ANY symptomatic SARS-CoV-2 infection, defined as: PCR-positive nasal swab **and** ≥ 1 of any of the following symptoms:
    - Fever.
    - New onset cough.
    - New onset or worsening of shortness of breath or difficulty breathing compared to baseline.
    - New onset fatigue.
    - New onset generalized muscle or body aches.
    - New onset headache.
    - New loss of taste or smell.
    - Acute onset of sore throat, congestion or runny nose.
    - New onset nausea, vomiting or diarrhea.
- Proportion of adolescent participants reporting SARS- CoV-2 infection (COVID-19) from Day 28 through endof Year 1, with severity classification as defined in the **Adult Main Study** (mild, moderate, or severe).
- Neutralizing antibody response at Day 35 for adolescent participants by age strata and with and without anti- SARS-CoV-2 NP antibodies at baseline, compared with that observed in adult participants 18 to <26 years of age from the **Adult Main Study** (Immunogenicity Population participants before crossover).
- Antibodies to SARS-CoV-2 NP at Days 0 and 35, andat Months 12, 18 and 24 will be used to determine natural infection and to determine the incidence of asymptomatic infection acquired during study follow-up.
- Serum IgG levels to SARS-CoV-2 S protein, hACE2 inhibition titers 14 days after second injection of the initial vaccination series (Day 35) in adolescent participants and subsets with and without anti-NP antibodies at baseline.
- Serum IgG levels to SARS-CoV-2 S protein, MN and hACE2 inhibition titers at Months 12, 18 and 24.
- Description of course, treatment and severity ofCOVID-19 reported after a PCR-confirmed case

[1] https://www.cdc.gov/coronavirus/2019-ncov/cases-updates/variant-surveillance/variant-info.html

via the Endpoint Form.

- Antibodies to SARS-CoV-2 NP, regardless of whether the infection was symptomatic.

5.3. Exploratory Endpoints

- First episode of PCR-positive COVID-19, as defined under the primary endpoint, shown by gene sequencing to represent a “variant of concern / interest” according to the CDC Variants Classification.
- Th1 or Th2 responses, eg, interleukin [IL]-2, IL-4, IL-5, IL-13, tumor necrosis factor alpha (TNF-α), interferon gamma (IFN-γ) in whole blood and/or harvested peripheral blood mononuclear cell (PBMCs).
- Quantitative PCR tests may be performed on nasal swabs collected from this trial to assess whether vaccination impacts viral shedding.
- Quantitative PCR tests performed on nasal swabs collected immediately prior to administration of blinded crossover vaccination to assess impact of initial vaccination on frequency of asymptomatic SARS-CoV-2 infection and level of viral shedding.
- Next-generation sequencing of viral genomes detected in nasal swabs tested by PCR to describe the genetic evolution of circulating SARS-CoV-2 strains during the conduct of the study.

6. Sample Size and Power

The sample size for the **Pediatric Expansion** is chosen to provide an adequate safety database of ≥2,000 recipients of investigational product to support licensure of the SARS-CoV-2 rS Matrix-adjuvanted vaccine in pediatric participants 12-<18 years of age. Recruitment of study participants will attempt to enroll a similar number of participants in the 12 to < 15 and 15 to < 18 year old age groups. With 2,000 participants in the active vaccine group, there is a >90% probability of observing at least 1 participant with an AE if the true incidence of the AE is 0.12% and a 99% probability if the true incidence of the AE is 0.23%. With 2,000 participants receiving at least 1 vaccine dose, there is a >99% probability of observing at least 1 participant with an AE if the true incidence of the AE is at least 0.12%.

The enrollment of approximately 3,000 adolescent participants with a 2:1 randomization to active vaccine or placebo will provide a total of approximately 2,000 pediatric participants exposed to active vaccine.

The analysis of efficacy in the **Pediatric Expansion** will be descriptive in nature using the same methods as the **Adult Main Study** but with no formal statistical hypothesis tested.

The blinded crossover approximately 6 months after completion of the initial vaccine series and 12 months follow-up after crossover will aim to collect ≥12 months safety data after receipt of the active vaccine.

A non-randomized non-inferiority (NI) analysis of immunogenicity will be performed between all the adolescents in the **Pediatric Expansion** and the 18-<26 years old adult population in the **Adult Main**

DocUUID : d222bbbe-1732-48af-b4cf-1e79e47eea3b

Study. A separate random sample of 750 participants aged 18-<26 years of age from the **Adult Main Study** will be drawn to provide approximately 400 participants for the NI analysis, accounting for the 2:1 randomization and (500 participants accounting for)20% non-evaluability. Similarly, 750 participants will be randomly selected from the **Pediatric Expansion** for testing of neutralization titers, which will provide approximately 400 participants for the NI analysis, accounting for the 2:1 randomization and 20% non-evaluability. Successful demonstration of non-inferiority will require meeting the following 3 pre-specified criteria simultaneously,

1. upper bound of 2-sided 95% CI for the ratio of GMTs ($GMT_{18\text{-}<26yo}$ /$GMT_{12\text{-}<18yo}$) < 1.5,
2. point estimate of the ratio of GMTs ≤ 1.22 (estimated as square root of 1.5)
3. upper bound of the 2-sided 95% CI for difference of seroresponse rates ($SRR_{18\text{-}<26yo}$ - $SRR_{12\text{-}<18yo}$) is < 10%

Assuming a standard deviation of $\log_{10}$ neutralization antibody titer of 0.6, there is over 85% power (through simulations) to demonstrate the first 2 non-inferiority criteria when assuming an underlying GMT for the 18-<26 years of age group up to 1.1-fold higher than the 12-<18 years of age group.

Below shows sample size justification on assumed GMT Ratio and standard deviation of $Log_{10}MN$.

Table 3 Power to conclude non-inferiority based on success criteria for GMT ratio ($GMT_{18\text{-}<26yo}/GMT_{12\text{-}17yo}$)

Number of Evaluable Participants Per Age Group	Standard Deviation of $\log_{10}$MN Antibody Titer	True GMT Ratio (18-<26yo / 12-17yo)	Power to Show Non-inferiority
400	0.6	1.0	97.9%
		1.05	93.7%
		1.1	85.5%
		1.15	72.7%
		1.2	56.6%
		1.25	40.1%
		1.3	25.7%

The table below shows the power to demonstrate the third non-inferiority criterion given various assumptions for seroresponse rates and an evaluable sample of 400 in each of the 18 to < 26 and 12 to < 18 years of age groups. In the absence of an established correlate of protection for SARS-CoV-2 vaccines, seroresponse will be defined as ≥ 4-fold increase in neutralization titers (MN_{50}) at Day 35 relative to baseline titers. With this definition and assumed seroresponse rates (SRR) of 95% in the 18 to < 26 years of age group, there is over 80% power to demonstrate the third non-inferiority criterion for a difference as large as

4% lower in the 12 to < 18 years of age group. A descriptive assessment of immunogenicity will evaluate the same criteria in the 12 to < 15 and 15 to < 18 years of age groups separately.

Seroresponse rate 18 to < 26 years of age group	Seroresponse rate 12 to < 18 years of age group	Power to Show Non-inferiority
95%	90%	72.9%
94%	90%	85.2%
93%	90%	92.9%
92%	90%	96.9%
91%	90%	98.8%
99%	95%	99.2%
98%	95%	>99%
97%	95%	>99%
96%	95%	>99%

7. Analysis Sets

Prospective approval of protocol deviations to recruitment and enrollment criteria, also known as protocol waivers or exemptions is not permitted

7.1. Intention-To-Treat (ITT) analysis set

The ITT analysis set will include all participants who are randomized, regardless of protocol violations or missing data. The ITT analysis set will be used for participant disposition summaries and will be analyzed according to the treatment arm to which the participant was randomized.

7.2. Full Analysis Set (FAS)

The FAS will include all participants who are randomized and received at least 1 dose of study vaccine/placebo, regardless of protocol violations or missing data. Participants who are unblinded with an intention to receive other COVID-19 vaccines will be censored at the time of unblinding. Participants in the blinded-crossover phase will be censored at the time of the receipt of crossover vaccination. The FAS population will be analyzed according to the treatment group to which they were randomized. The FAS analysis sets will be used for supportive analyses. When the efficacy endpoints are analyzed using FAS, baseline SARS-CoV-2 seropositivity or nasal swab PCR-positivity will be ignored.

7.3. Safety (Safety) Analysis set

The safety analysis set will include all participants who receive at least 1 dose of trial vaccine. Participants in the safety analysis set will be analyzed according to the treatment actually received. In cases where information is available that indicates that a participant received both active and placebo vaccine during the initial or crossover period, the participant will be analyzed as part of the active group.

7.4. Per-Protocol Efficacy (PP-EFF)

The PP-EFF analysis set will include all participants who receive the full prescribed regimen of trial vaccine and have no major protocol deviations that occur before the first COVID-19 positive episode (ie, participant will be censored at the time of the protocol deviation) and are determined to affect the efficacy outcomes, including baseline SARS-CoV-2 seropositivity or nasal swab PCR-positivity. Participants who are unblinded with an intention to receive other COVID-19 vaccines will be censored at the time of unblinding. Although the study will enroll participants regardless of SARS-CoV-2 serologic status at the time of initial vaccination, any participants with confirmed infection or prior infection due to SARS-CoV-2 at baseline, by nasal swab PCR or serology, will be excluded from the PP-EFF population. PP-EFF will be the primary set for all efficacy endpoints. Participants determined to have positive nasal swab PCR or serology immediately prior to the first crossover vaccination will be excluded from the post-crossover PP-EFF population.

7.5. Per-Protocol Efficacy Analysis Set 2 (PP-EFF-2)

A second per-protocol efficacy (PP-EFF-2) analysis set is defined to allow for evaluation of baseline serostatus impact on vaccine efficacy. The PP-EFF-2 analysis set will follow the same method describe in the PP-EFF population with the exception that it will include all participants regardless of baseline serostatus (based on anti-NP). The analysis of vaccine efficacy using this population will be dependent on whether there exists an endpoint event for the relevant analysis where the subject has a baseline positive anti-NP result.

The review and determination for exclusion from the PP-EFF and PP-EFF-2 Analysis sets will be carried out in a blinded fashion by a study clinician based on all available information prior to unblinding.

7.6. Per-Protocol Immunogenicity (PP-IMM) analysis sets

The PP-IMM analysis set will be determined for each study visit and may be assay specific (i.e., Serum vs PBMC, and within Serum, IgG, MN, hACE2). The PP-IMM analysis set will include participants that have at least a baseline and 1 sample result available after vaccination and have no major protocol violations that are considered clinically relevant to impact immunological measures prior to the visit in question. The PP-IMM analysis set will also exclude participants who have a PCR positive nasal swab between baseline up to the visit analyzed. All participants in the PP-IMM analysis population will be designated at time of vaccination within the immunogenicity subset. For participant visits on or after Day 21, participants must receive the second vaccination to be included in the PP-IMM analysis set. Prior exposed participants will be determined using baseline SARS-CoV-2 nasal swab or seropositivity at screening to assess if immune responses differ between previously exposed and unexposed individuals. This analysis set will be labelled PP-IMM-2. Durability of immune responses will be evaluated in participants who

DocUUID : d222bbbe-1732-48af-b4cf-1e79e47eea3b

provide serologic data at Months 12, 18 and 24, taking into account when they received active vaccine and if/when they were infected with SARS-CoV-2, based on PCR or serology.

The review and determination for exclusion from PP-IMM and PP-IMM-2 analysis sets will be carried out in a blinded fashion by a study clinician prior to unblinding for the analysis based on all available information from the locked database.

7.7. Protocol deviations/violations and exclusions from analysis sets

Protocol deviation data will be captured in the clinical trial management system (CTMS). Extracts of all protocol deviations will be provided to biostatistics periodically and at the time of the analysis. All protocol deviations and other intercurrent events impacting exclusion of participants from the analysis sets will be identified prior to the analysis, through clinical review input provided by the sponsor, using the following sources of information:

- Supportive participant listings provided by the ICON statistician based upon data recorded in the clinical trial management system.

Furthermore, deviations from the protocol will be classified as major or minor. Classification of major (key) and minor (non-key) protocol deviations is determined prior to participant enrollment and is outlined in detail in the protocol deviation criteria documentation managed by the clinical trial management team.

The following protocol deviations or intercurrent events will be programmed to verify completeness of the the protocol deviation data captured in the CTMS, prior to compilation, review and approval of final list of exclusions from analysis sets:

- o Window for Day 35 blood draw, being at least 14 days from Dose 2 and at most 28 days after Dose 2
- o Dose 2 not administered but the subject provides a Day 35 blood draw

8. Statistical Considerations and Analysis

8.1. Endpoint Review Committee for Severe Cases

Potentially severe cases of symptomatic PCR positive COVID-19 will be reviewed by an external Independent Medical Review Committee (IMRC) established by the ICON Independent DMC and Adjudication (IDEA) group. The IMRC will consist of physicians who have clinical and research experience (e.g., medical review and/or clinical trial experience) in infectious disease. The committee's structure, responsibilities and operation is specified within a charter. Potentially severe cases include COVID-19 reported as serious adverse events, programmatically identified endpoints consisting of at

DocUUID : d222bbbe-1732-48af-b4cf-1e79e47eea3b

least one pulse oximeter reading ≤ 93%, and episodes identified as severe on the Endpoint Assessment CRF. Patient profiles (as outlined in the charter) will be provided to the committee members for review according to the process outlined in the charter. These patient profiles include demographics, medical history, adverse events, concomitant medications, reported daily symptoms, and the Endpoint Assessment CRF. The external reviewers will document the criteria used for their clinical review of the cases.

The results of the review will be to confirm the case as severe or rule that the case is not severe. The outcome of the review for each case is stored in an electronic medical review system. A file will be exported from the system and provided to the ICON Biostatistics group for use in analysis. Cases that are ruled as not severe by the committee when the investigator has entered a severe grading will be further reviewed and documented by Novavax clinician(s) prior to unblinding to determine the severity to use in analysis. Cases that are ruled as severe by the IMRC but not severe by the investigator will be analyzed as severe in the analysis.

8.2. Derived Variables

The below table provides the list of derived variables for demographic and baseline characteristics, various duration derivations, baseline derivation and other important derivations applicable for this study. Only PCR test results from the University of Washington Virology Laboratory (central laboratory for nasal swab testing) will be used for analysis.

Table 4 Derived Variables

Variables	Formula
**Demographic and Baseline Characteristics**	
Body Mass Index	Weight (kg) / [height (m)]^2
**Derivation of Durations**	
Study day at any visit	Date of interest – date of first dose of trial vaccine. One day is added if the difference is ≥ 0.
Duration of any events	End Date of Event – Start date of event + 1
**Endpoints**	
Start of illness episode	The earlier of the date of positive PCR date from the University of Washington Virology Laboratory or start of symptoms as reported on the COVID-19 Endpoint CRF will be used as the start of the illness episode.

DocUUID : d222bbbe-1732-48af-b4cf-1e79e47eea3b

Variables	Formula
First episode of COVID-19 disease (Primary endpoint)	Minimum date of the following events (both events must occur): date of PCR positive result from the University of Washington Virology Laboratory ; date of the start of mild, moderate, or severe COVID-19 disease from the COVID-19 Endpoint assessment CRF.
Start of moderate or severe COVID-19 disease (Secondary endpoint)	Minimum date of the following events (all events must occur): date of PCR positive result from the University of Washington Virology Laboratory; date of the start of moderate or severe COVID-19 disease from the COVID-19 Endpoint assessment CRF.
Start of any symptomatic COVID-19 infection	Minimum date of the following events (both events must occur): date of PCR positive result from the University of Washington Virology Laboratory; date of the start of symptoms for any confirmed COVID-19 infection regardless of severity as reported on the COVID-19 Endpoint assessment CRF.
Time to COVID-19 disease	Start date of COVID-19 disease as defined in above categories – start of observation period.<br>The start of observation period is defined for the primary and secondary endpoints in the FAS and PP-EFF analysis sets as the date of second injection + 7 days.<br>An additional observation period for the primary and secondary endpoints is defined for the FAS and PP-EFF analysis sets as the date of first vaccination + 1 day.
Variant of Concern (VoC) / Variant of Interest (VoI)	VoI/VoC are defined as strains not immunologically similar to the prototype strain used to manufacture the vaccine. These are defined by the CDC at the web page https://www.cdc.gov/coronavirus/2019-ncov/cases-updates/variant-surveillance/variant-info.html and referenced in section 3.2 of this SAP. The VoI/VoC listed at the time of data extract will be used for analysis. University of Washington will identify infective strains in PCR positive subjects.
**Other Derivations**	
Vaccine Efficacy	1 – relative risk * 100 (expressed as percent)
Relative Risk	Risk in the vaccinated sample / Risk in the unvaccinated sample. Estimated by various methods through Poisson regression or Cox Proportional Hazards Regression, as defined in this SAP.
Geometric mean EU/Geometric mean titers	Individual values for a subject will be transformed via the natural log, averaged along with summary statistics, and then back transformed using Euler's number (e) to obtain the geometric mean in the original scale.
Geometric mean fold rise (GMFR)	Measures the changes in immunogenicity titers within subjects. The mean of the subject's log ratio of values of post dose assay relative to baseline (usually) is back-transformed to calculate the GMFR.
Seroconversion Rate (SCR)	Defined as percentage of subjects with a value ≥ 4 folder difference between Day 35 and Day 0. Seroconversion is a binary variable defined as 1 if the post-vaccination value is ≥ 4times the baseline value. Otherwise, the variable equals 0.
Incident rate in a group	Total number of events experienced within the group / (the total number of person-years contributed by the group) * multiplication factor. Where the factor will equal the numerical number of person-years.

Variables	Formula
Person-year	The number of years a participant contributes to the study defined as the end date of the observation of the subject minus the start date of the observation of the subject + 1 day. (e.g. the start date of observation for SAEs is the date of first vaccine dose to the date the subject has completed the study or terminated early from the study). The end date may be a censoring date as specified for the analysis set.
Incidence in a group	The proportion of the participants experiencing the outcome of interest compared to the total number of participants at risk of experiencing the outcome of interest. (e.g. the incident rate of SAEs after vaccination are the number of participants with an SAE reported after vaccination divided by the number of participants that received the vaccination).

8.3. Handling of missing data and outliers

The extent and pattern of missing data for primary and secondary efficacy endpoints will be summarized separately by treatment groups. No imputations will be conducted for missing efficacy data. No data are excluded due to being an outlier.

8.3.1. Missing data analysis methods

Any data missing from this study to assess the primary endpoints will be assumed to be missing completely at random and no imputation methods will be considered.

8.3.1.1. Handling of missing or incomplete dates

Imputation of missing or incomplete dates that relate to adverse events and concomitant medications should not result in the end date falling after the date of data extraction or date or date of death (if applicable).

<u>Imputation rules for missing or partial adverse event start/stop dates</u>

- If the AE start date day is missing (month and year provided) then set the date to the first of the month, unless the month and year are the same as a dosing event. In this case, set the date to the date of the dosing event.
- If the AE start date month is missing (year is provided) then set the month and day to January 1, unless the year is the same as the year of a dosing event. In this case, set the date to the earliest dosing event in that year.
- If the AE end date day is missing (month and year provided) then set the date to the last day of the month.
- If the AE end date month is missing (year is provided) then set the date to December 31.

- If the year of the AE start date or AE end date are missing, then a query to the site must be made to gather additional information. If the end date and start date are both missing, then no imputation will be done. If the start date remains missing but the end date is before first dose date, then the AE will be considered before treatment. If the end date is after the first dose, then the AE will be considered to have been treatment emergent.

Imputation rules for missing or partial medication start/stop dates

Start Date:

- If only day is missing, use the first day of the month.
- If day and month are missing, use the first day of the year.
- If day, month, and year are missing use the first day of the year with the same year as the first dose.

End Date:

- If only day is missing, use the last day of the month.
- If day and month are missing, use the last day of the year.
- If day, month, and year are missing assign 'continuing' status to the stop date.

9. Statistical Methods

9.1. General statistical conventions

Unless otherwise stated, all statistical testing will be two-sided and will be performed using a significance (alpha) of 0.05. Two-sided 95% confidence intervals (CI) will be provided when relevant.

Continuous variables will be summarized using descriptive statistics, including number of participants (n), mean, median, standard deviation (SD), minimum, and maximum. Means and medians will be rounded to one more decimal place than was is reported in the data. Standard deviations will be rounded to two more decimal places than what is reported in the data.

Immunology data consisting of Elisa Unit (EU) and titer data will be summarized using geometric means, also known as geometric mean EUs/titers (GMEU/GMT), geometric mean fold rise (GMFR) and seroconversion rate (SCR). Immunology results below the lower limit of quantification (LLOQ) will be summarized and reported using 0.5*LLOQ. Immunology results above the upper limit of quantification (ULOQ) will be summarized and reported using the ULOQ.

For categorical variables, summaries will include counts of participants and percentages. Percentages will be rounded to one decimal place. Confidence intervals surrounding proportions may be constructed using a normal approximation for larger samples and exact methods for smaller samples, whichever is appropriate for the data.

DocUUID : d222bbbe-1732-48af-b4cf-1e79e47eea3b

For summary purposes, baseline will be defined as the last available pre-dose value. All summaries will be presented by treatment group and stratification variable where appropriate, unless otherwise specified.

All participant data, including those derived, will be presented in individual participant data listings. Unless otherwise stated, unscheduled visit results will be included in date/time chronological order, within participant listings only. All listings will be sorted by investigational site, participant ID, date/time and visit. The participant's sex and age will be stated on each listing. The treatment group will be presented when the study is unblinded at the subject-level; or if listings are provided to persons designated as able to view subject-level unblinded output. Unless otherwise stated, data listings will be based on all participants randomized (ITT Analysis set).

Unscheduled and out of window data points will be reviewed during a review of protocol deviations, prior to unblinding, and a determination will be made as to whether that data point will be used in the analysis. Documentation and approval by the sponsor will be generated prior to unblinding.

9.2. Participant disposition

Participant disposition information will be summarized by treatment group, race, ethnicity, sex at birth, country, and overall. A CONSORT diagram will be constructed that details the number of participants screened, the number enrolled and eligible for vaccination, the number randomized to each trial vaccine arm, the number receiving the first and second vaccination, the number of early terminations, the number completing the study, and the number eligible for analysis.

Completeness of visit data will be tabulated, showing at each timepoint the proportion of completed visits vs expected visits.

The primary reason for early withdrawal will also be tabulated.

The number of participants randomized will be used as the denominator for the proportion calculation. Participant disposition will be listed along with inclusion or exclusion criteria that were not met for randomization.

The number and percent of participants in each analysis set will also be tabulated. A listing of each participant excluded from an analysis set will be listed as well as the reason why they were excluded from the analysis set.

A summary of unblinded participants along with the reason for unblinding will be summarized by treatment group and baseline serostatus.

Treatment misallocations occur when a participant received a different treatment than they were randomized to. In these cases:

DocUUID : d222bbbe-1732-48af-b4cf-1e79e47eea3b

- If a participant was randomized but not treated, then they will be reported under their randomized treatment group the participant disposition summaries.
- If a participant was treated but not randomized, then by definition they will be excluded from the efficacy analyses since randomized treatment is missing but will be reported under the treatment they actually received for all safety analyses.
- If a participant was randomized but took a different treatment, then they will be reported under their randomized treatment group for all efficacy analyses in the FAS but will be reported under the treatment they actually received for the first dose for all safety analyses and efficacy analyses in the PP-EFF analysis set, as well as the PP-IMM analysis set.
- If a participant was randomized and received both active and placebo product, the participant will be classified as active study drug for safety analyses and as randomized for efficacy analyses in the FAS. These participants will be excluded from the PP-EFF and PP-IMM analysis sets.

9.3. Protocol deviations

The number of participants excluded from ITT, FAS, Safety, and Per-protocol analysis sets and reasons for exclusion will be summarized by treatment group and overall.

Analysis set membership details will be listed, including reason for exclusion from each analysis set (on randomized participants).

Protocol deviation data are captured in the clinical trial management system and will be provided as an external data source to the Biostatistics team. A summary table that categorizes the type of deviation and the category (e.g., major or minor) will be generated for the ITT analysis set. Protocol deviations leading to exclusion of subjects from analysis sets will be summarized in the final clinical study report.

9.4. Demographics and baseline characteristics

9.4.1. Demographics

Age, height, weight, and other continuous demographic variables at baseline will be summarized descriptively. Sex, primary race, ethnicity, and other categorical variables will be summarized using the ITT and PP analysis sets.

9.4.2. Baseline and disease characteristics

Results for baseline SARS-CoV-2 serostatus will be summarized using frequency counts and proportions for qualitative results and geometric mean titers for quantitative results will be presented for the ITT and Per Protocol analysis sets.

9.4.3. Medical history

A summary of prior and concomitant medical conditions, recent vaccinations (≤ 90 days), and significant surgical procedures will be presented by system organ class (SOC) and preferred term (PT) using the

DocUUID : d222bbbe-1732-48af-b4cf-1e79e47eea3b

Medical Dictionary for Regulatory Affairs (MedDRA) version 24.0. Medical history data will be used to classify baseline comorbidities and high-risk medical groups in various subgroup analysesA mapping of MedDRA coded terms to comorbidities classifications and baseline medical histories of interest will be provided in Appendix 1 of the SAP.

9.4.4. Prior and concomitant medications

Medications used in this study will be coded by using the World Health Organization Drug Dictionary Enhanced (WHODrug version Mar2021 B3).

Prior medications are defined as those medications with a start date prior to signing the informed consent form (ICF).

Concomitant medications will include all medications (including vaccines) taken by the participant from the time of signing the ICF through Day 49 of the initial vaccination period and all medications taken by the participant for treatment of a reportable SAE, MAAE or AESI from Day 50 through end of study (or through the early termination visit if prior to that time).

Prior and concomitant medications will be summarized descriptively using frequency tables by ATC class and preferred name by treatment group on the FAS and presented separately for the following groups:

- Concomitant medications continued after initial study drug dose
- Prior and concomitant use of any prohibited medications as described in protocol section 8.4. This list is currently inclusive of only medications for the prevention of COVID-19 and influenza vaccinations received within 4 days prior to or 7 days after either study vaccination.

Details for imputing missing or partial start and/or stop dates of medication are described in section 8.3.1.1.

9.5. Extent of exposure

9.5.1. Treatment duration

Study drug exposure will be summarized as the number of participants receiving a total of 1 and 2 doses of vaccine product by study period (initial and crossover) and will be presented by treatment group on the safety, FAS, and PP analysis sets.

9.5.2. Treatment compliance

A full course of study product administration consists of two doses of vaccine in each study vaccination period (initial and crossover). A summary table detailing the total number of doses received will be presented along with the reason a second dose was not administered or missed. A summary of

DocUUID : d222bbbe-1732-48af-b4cf-1e79e47eea3b

participants receiving the second dose outside the dosing window, 21 to 28 days post first dose, will be presented.

9.6. Efficacy Analyses

This section addresses separately the analyses to be conducted on the primary and secondary efficacy variables.

Analysis of the primary and the secondary efficacy endpoints will be performed based on the data generated prior to the blinded crossover using the PP-EFF analysis set. Secondary efficacy endpoints may not be analyzed if there are not a sufficient number of relevant cases. If an analysis is not performed, it will be noted in the clinical study report. The analysis of data generated after the blinded crossover or the combined analyses of both pre- and post-blinded crossover may be performed using the approach described by Follmann et al [Follmann 2020, Fintzi 2021] provided there is a sufficient number of relevant cases.

9.6.1. Analysis methods

Estimate Vaccine Efficacy

A Poisson regression model utilizing robust error variance and an offset to account for variable follow-up time will be used to estimate the relative risk and vaccine efficacy. The outcome variable will be defined as participants experiencing a positive diagnosis and symptoms of COVID-19 disease as defined in the primary and secondary efficacy objectives of the trial. The main explanatory variable will be the treatment arm (active vaccine or placebo). A repeated measure by participant ID assuming an unstructured variance model will be included to obtain the robust error variance method as described in Zou, 2004. The offset is defined as the log of the follow-up time variable, where follow-up time is defined as the number of days from the start of the observation period to diagnosis of COVID-19 or end of the observation period. The observation period will end after reaching the first occurrence of any of the following events: a positive endpoint definition is reached, the subject terminates from the study early, the subject dies, the subject completes the study as scheduled, the study is stopped early, start of a PCR-confirmed illness episode, any positive PCR result after screening, participant enters the blinded crossover, occurrence of major protocol deviation, or the subject is unblinded to treatment assignment. Any subject with a censoring date occurring prior to the start of the observation period will be excluded from the respective analysis set. The offset will allow the modeling of rate data rather than count data and will account for the variability of follow-up time each participant will contribute to the trial. The relative risk due to treatment effects will be gathered from exponentiating the treatment group variable coefficient. Subtracting the relative risk estimate from 1 will provide the estimated vaccine efficacy due to active vaccine. A two-sided confidence interval around the estimate will also provide the Maximum Likelihood

Estimator (MLE) 95% confidence interval around the vaccine efficacy. Due to the potential for sparse numbers of cases among potential covariates, some covariates may not be able to be evaluated and will not be considered in the model.

The following sample of SAS code will be used to construct the Poisson regression model:

```
proc genmod data = <DATASET>;
      class armcd usubjid agestrata;
      model <OUTCOME> = armcd
            / dist = poisson link = log offset=<LN(TIMEVAR)>;
      repeated subject = usubjid/ type =
      unstr; estimate 'Beta' <ARMCD> 1 -1/
      exp;
run;
```

The Poisson model assumes the response variable is a count and therefore each participant will take on values of 0 or 1 where 1 indicates the occurrence of the outcome of interest, that each observation is independent of each other, the mean equals the variance measure, and the log of the mean rate is a linear function of the explanatory variables.

Provided that the Poisson model converges, a Cox proportional hazard (CPH) model using the same dependent and explanatory variables will be developed as a supportive analysis. Ties will be handled with the Efron approximation method. Inference based assessments will use Wald test statistics. The time from 7-days post second vaccination to the event of interest or end of observation period (as defined earlier) will be used as the predictive variable. Vaccine efficacy will be defined as 1 – the hazard ratio of the treatment group. Participants will be censored if no outcome of interest was experienced by the end of the observation period. A sample of SAS code to be used in building the CPH model is as follows:

```
proc phreg data=TTE;
      class armcd(ref=”placebo”);
      model TIME*CENSOR(1)=ARMCD
             /risklimits ties=EFRON;
      run;
```

In the case where there are zero endpoints for one of the vaccine groups or the total number of endpoints in both treatment groups combined is less than 5, a Poisson model will be substituted with an exact conditional binomial method. This method conditions on the total number of events across the treatment groups where the number of events in the active group are generated from a binomial distribution with success parameter equal to:

$$\pi = \frac{\#\ events\ in\ active\ group}{\#\ total\ events\ in\ both\ groups\ combined}$$

If T_i represents follow-up time in group i, and n_i represents number of events in group i, then the rate of events in group i is $\frac{n_i}{T_i}$. The vaccine efficacy is estimated as:

$$VE = 1 - \left[\frac{\frac{n_A}{T_A}}{\frac{n_P}{T_P}}\right] = 1 - \frac{\pi}{(\frac{T_A}{T_P}) * (1 - \pi)}$$

where T_A is observed time in the active group = A and n_A is the number of events in the active group, with the corresponding values for the placebo group = P being T_P and n_P . The exact Clopper-Pearson confidence interval $(\hat{p}_{LB}, \hat{p}_{UB})$ is obtained on the observed proportion $[\hat{p} = n_A/(n_A + n_P)]$ of events in the active group over the total number of events across both groups, and back-translated to present in terms of vaccine efficacy (VE).

$$(VE\ Lower\ Bound, VE\ Upper\ Bound) = (1 - \frac{\hat{p}_{UB}}{(\frac{T_A}{T_P}) * (1 - \hat{p}_{UB})}, 1 - \frac{\hat{p}_{LB}}{(\frac{T_A}{T_P}) * (1 - \hat{p}_{LB})})$$

<u>Non-inferiority of Effectiveness</u>

Non-inferiority of immune response based on neutralization titers for adolescents 12 -< 18 years of age compared to adults 18 -< 26 years of age will be demonstrated if all 3 criteria stated in Section 6 are met. Additional details are provided in section 9.6.3.1 below.

9.6.1.1. Multiplicity

The pediatric expansion part of this study will be not be adjusted for multiplicity.

9.6.2. Analysis of primary efficacy endpoint(s)

The primary endpoint will be analyzed on the PP-EFF analysis set and supported by analysis of the FAS analysis set. The statistical method used will be identical to the Adult Main Study. However, the analysis will be descriptive in nature with no formal statistical hypothesis tested.

The VE is defined as VE (%) = (1 – RR) x 100, where RR = relative risk of incidence rates betweenthe 2 trial vaccine groups (SARS-CoV-2 rS / Placebo).

The primary analysis will be conducted in the PP-EFF and FAS analysis sets. Additionally, the primary efficacy endpoint may be evaluated in the PP-EFF2 population among the seropositive and seronegative participants.

DocUUID : d222bbbe-1732-48af-b4cf-1e79e47eea3b

The primary efficacy analysis will be conducted with data collected up to the blinded crossover. The subject's follow-up time for primary efficacy will be censored at the time of crossover and other events defined in section 9.6.1 above.

9.6.3. Analysis of secondary efficacy and immunogenicity endpoints

The secondary efficacy endpoints are descriptive in nature with no formal statistical hypothesis testing. Baseline PCR positive samples as well as PCR positive samples from the Acute Illness Visit are sent to the University of Washington Virology Laboratory for gene sequencing.

Vaccine efficacy will be calculated for the first episode of PCR positive, moderate or severe, COVID-19 disease due to a strain shown by gene sequencing to represent a non-"Variant of Concern/interest" according to CDC classification, starting at least 7 days post vaccination 2 in the initial vaccination period. A non-Variant of Concern/Interest is any PCR-sequenced sample where the strain does not meet the definition of Variant of Concern/Interest presented in Table 4. PCR positive samples with sufficient viral load are chosen for sequencing. The classification of variants is conducted by the University of Washington Virology Laboratory and provided for analysis.

For the serum antibody levels specific for the SARS-CoV-2 S protein antigen(s) (IgG antibody to SARS-CoV-2 S protein and hACE2 inhibition) and MN, the geometric mean at each study visit, the geometric mean fold rise (GMFR) comparing to the baseline (Day 0) at each post-vaccination study visit, and the GMFR comparing pre- and post-second dose, along with 95% CI will be summarized by trial vaccine group. The 95% CI will be calculated based on the t-distribution of the log-transformed values for geometric means or GMFR, then back transformed to the original scale for presentation. Seroconversion (SCR) will also be calculated with exact 95% CI. SCR is defined as achieving a 4-fold rise in antibody response from baseline.

Similar summaries will be generated for the other immunogenicity endpoints and other assays if conducted.

A descriptive analysis will be carriedout for the serum antibody levels specific for the SARS-CoV-2 S protein antigen(s) (IgG antibodyto SARS-CoV-2 S protein and hACE2 inhibition) at Day 35. The secondary immunogenicity analyses will be performed using the PP- IMM analysis set and the FAS. The analysis of SCR will include calculation of the difference of SCR between groups with a 95% CI computed with the method of Miettinen and Nurminen.

9.6.3.1. Effectiveness Analysis

A formal non-randomized non-inferiority (NI) analysis of the primary effectiveness endpoint, neutralizing antibody to SARS-CoV-2 at Day 35, will be carried out using the PP-IMM analysis set. The NI analysis of adolescents compared with the 18-<26 year old immunogenicity cohort from the Adult Main Study will be

DocUUID : d222bbbe-1732-48af-b4cf-1e79e47eea3b

performed using the point estimate and upper bound of the two-sided 95% Confidence Interval (CI) on the ratio of GMTs between 2 age cohorts (adults 18-<26 year old cohort in the Adult Main Study/adolescent cohort in the Pediatric Expansion) against the pre-specified success criteria. The ratio of GMTs between 2 age cohorts and the corresponding two-sided 95% CI will be calculated on log-transformed titers using the analysis of covariance (ANCOVA) with age cohort and baseline (Day 0) measurement as the covariate. In addition, difference of seroresponse rates between the two age cohorts will be computed using a definition of seroresponse as 4-fold rise in neutralization titers at Day 35 relative to Day 0. The two-sided 95% CI on this difference of seroresponse rates will be computed based on the method of Miettinen and Nurminen.

Successful demonstration of non-inferiority will require meeting the following 3 pre-specified criteria simultaneously,

- upper bound of 2-sided 95% CI for the ratio of GMTs ($GMT_{18\text{-}<26yo}/GMT_{12\text{-}<18yo}$) < 1.5,
- point estimate of the ratio of GMTs ≤ 1.22 (estimated as square root of 1.5)
- upper bound of the 2-sided 95% CI for difference of seroresponse rates ($SRR_{18\text{-}<26yo}$ - $SRR_{12\text{-}<18yo}$) is < 10%

The following is a sample of SAS code to compute the ratio of GMT between the two age cohorts:

```
proc mixed data= <DATASET>;
  class <age cohort>;
  model log(<D35 titer>) = log(<baseline titer>) <age
  cohort>;
  lsmeans <age cohort>/cl diff e alpha=0.05;
run;
```

9.6.3.2. Durability of vaccine efficacy

Dependent on the number and nature of endpoint cases in the post-crossover period, Cox regression may be used to estimate a smoothly varying vaccine efficacy index by time since vaccination as described in Fintzi and Follmann (2021). This will be conducted using SAS PROC PHREG with a time-varying vaccine efficacy that is a log-linear function of time since vaccination with time index the number of days since the start of the study.

Briefly, the hazard ratio for vaccine compared to placebo is assumed to be $exp(B0 + B1\, t)$ where t is the time since vaccination. Other functions such as $exp(B0 + B1\, t + B2\, t^2)$ may also be used if warranted. Since cases are not counted until 7 days after the second dose of the initial and crossover immunization periods, volunteers are not at risk during these periods. Participants who have an event during the pre-crossover period contribute only during that phase. Participants who avoid an event during the pre-crossover period and successfully complete the crossover immunization event free, also contribute for the

post crossover period. For each person we record the times of study entry, the time last seen (i.e. censoring or disease) and if applicable, the start and end of crossover.

These analyses may be performed for the primary endpoint and for other related endpoints such as severe disease and moderate/severe disease if enough numbers of endpoints are accrued.

9.7. Analysis of exploratory endpoint(s)

Vaccine efficacy will be calculated for the first episode of PCR positive, symptomatic, COVID-19 disease due to a strain shown by gene sequencing to represent a “Variant of Concern/interest” according to CDC classification, starting at least 7 days post vaccination 2 in the initial vaccination period. A Variant of Concern/Interest is any PCR-sequenced sample where the strain meets the definition of Variant of Concern/Interest presented in Table 4. The selection of PCR positive samples for gene sequencing is described in Section 9.6.3. The analysis approach will use the same approach as the primary objective and key secondary efficacy endpoints in the FAS and PP-EFF analysis sets.

Th1 or Th2 responses, eg, interleukin [IL]-2, IL-4, IL-5, IL-13, tumor necrosis factor alpha (TNF-α), interferon gamma (TFN-γ) in whole blood and/or harvested peripheral blood mononuclear cell (PBMCs) will be evaluated descriptively over time in plots and tables. This will be conducted for PP-IMM analysis set, subset for the subjects who have PBMCs collected. The population will be labelled as the CMI Subset.

Quantitative PCR tests may be performed on nasal swabs collected from this trial to assess whether vaccination impacts viral shedding.

9.8. Safety Analysis

This section describes the safety analyses that will be conducted on the treatment and follow-up periods (i.e., the safety analyses on all data collected during the treatment and follow-up periods and all data collected in participants who dropped-out during the treatment and follow-up periods).

All definitions relative to safety endpoints are detailed in section 8.2.

Safety analyses will be conducted on the safety analysis set and will be performed for all safety variables specified below.

An initial safety review will be performed in the first ~60 subjects of unsolicited AEs and reactogenicity events through day 7 post dose 1. This review will be conducted prior to the dosing of the remaining subjects in the cohort. Likewise, administration of the second vaccine dose to the full participant population will be contingent upon the review of early safety data (ie, 7 days of reactogenicity and overall safety post-dose 2) in the same ~60 early-enrolled adolescents before dosing the remainder of the

adolescent participants. Safety data will be provided to and reviewed by the Data and Safety Monitoring Board (DSMB) after each early safety data review period (ie 7 days after each vaccination in the sentinel group). Simultaneously, safety data will be reviewed internally by the Sponsor.

All unblinded safety data analyses will be summarized by treatment group. Comparison of proportions of subjects experiencing each event may be inaccurate due to variable rates of follow-up time during the initial vaccination period and the crossover vaccination period. Therefore, adverse event rates will be displayed instead of proportions. AE rates are defined as the total number of events of interest experienced divided by the months of follow-up time. An appropriate multiplier will be applied across any summary tables depending on the observed rate to assist in the display.

Solicited adverse event(s) collected via the eDiary reported after the date of consent withdrawal or end of study will not be included in the analysis. No statistical tests will be performed on the safety endpoints.

9.8.1. Adverse events

All adverse events (AE) will be classified by Primary System Organ Class (SOC) and Preferred Term (PT) according to the Medical Dictionary for Regulatory Activities (MedDRA) Version 24.0.

In summaries by SOC and PT, adverse events will be sorted by descending rates within each SOC and PT according to the incident rate in the entire study. In summaries by PT, AEs will be sorted by decreasing rate according to the incident rate in the entire study.

AEs will be classified as treatment emergent (TEAE), or post-treatment defined as any AE that was newly developed at or after the first dose date of study vaccine. Unsolicited Adverse events will be collected up to 28 days after the second vaccination for the initial and crossover portion of the trial. Reactogenicity events will only be captured for the initial set of vaccinations and will not be collected following the crossover. Reactogenicity events will be reported as the number of subjects experiencing the event along with an exact 95% confidence interval instead of rates because follow-up time will be constant for all subjects.

Details for imputing missing or partial start dates of adverse events are described in section 8.3.1.1.

AE summary tables will be presented for the following groupings of AEs:

- Reactogenicity (Local and Systemic) from vaccination to day 7 post vaccination, overall, by severity, and vaccination sequence (first vs second dose).
- Medically attended adverse events (MAAEs) up to 49 days post first vaccination (initial and crossover) and attributed to vaccine and after day 49 through the end of study or until crossover. Tables will also be presented by severity.

DocUUID : d222bbbe-1732-48af-b4cf-1e79e47eea3b

- All unsolicited AEs through 49 days post first vaccination (initial and crossover), presented overall and by severity.
- All unsolicited AEs through 49 days post first dose (initial and crossover), presented overall and by severity.
- All MAAEs attributed to study vaccine, SAEs, and AESIs through Month 12.
- All MAAEs attributed to study vaccine, SAEs, and AESIs from crossover to the end of study.
- Death due to any cause
- AEs leading to study and treatment discontinuation

All AEs will be summarized by SOC, PT and treatment group. In addition, an overall summary for categories above will be prepared by treatment group, sex, race, ethnicity, high risk category/status, and overall.

9.8.1.1 Potential Immune Mediated Medical Conditions (PIMMC)

For the analysis of PIMMC, three approaches will be taken. First, PIMMC will be summarized based on site reporting as captured in flags on the Adverse Event CRF. Second, a summary will be created based on Protocol Defined Criteria as presented in Table 11 in Appendix 2 of the protocol. Third, a summary will be created based on a combination of these two approaches.

9.8.2. Vital signs

Visit values for vital sign measurements (pulse rate, resting systolic and diastolic blood pressure, temperature, respiratory rate) will be summarized by treatment group at each visit using descriptive statistics. Visit values will be calculated as the mean of all available measurements per parameter.

9.8.3. Physical examinations

All baseline physical examination abnormalities will be listed. Any abnormal findings or worsening findings post baseline will be reported as adverse events as per the protocol and will be analyzed following the safety section above.

9.8.4. Electrocardiograms

Not Applicable

9.9. Subgroup Analysis

The analysis may be conducted to determine if there is a difference in efficacy and safety of the vaccine by:

- Age group (12-<15 years of age, 15-<18 years of age)
- Gender
- Race
- Ethnicity

These analyses may be conducted in the efficacy, immunogenicity, and safety analysis sets as appropriate for the type of analysis being conducted and will be displayed in the results. All analyses would be descriptive. Many of these subgroups have been defined as part of the primary and secondary analyses described previously in this SAP.

10. Changes to Planned Analysis from Study Protocol

The protocol definition of obesity is not inclusive (>) of 30 kg/m2, however the standard definition of obesity as defined be the CDC is inclusive (≥) of 30 kg/m2. This SAP used the CDC standard definition to ensure results are consistent with other studies and understanding of the definition.

Removed censoring of unblinded subjects from the Safety Analysis Set definition.

11. References

Development and Licensure of Vaccines to Prevent COVID-19; Guidance for Industry, June 2020, https://www.fda.gov/media/139638/download.

Emergency Use Authorization for Vaccines to Prevent COVID-19; Guidance for Industry, October 2020, https://www.fda.gov/media/142749/download.

Fintzi, J. and Follmann, D., 2021. Assessing Vaccine Durability in Randomized Trials Following Placebo Crossover. [online] arXiv.org. Available at: <https://arxiv.org/abs/2101.01295> [Accessed 15 February 2021].

Follmann D, Fintzi J, Fay MP, et al. Assessing durability of vaccine effect following blinded crossover in COVID-19 vaccine efficacy trials. medRxiv 2020.12.14.20248137; doi: https://doi.org/10.1101/2020.12.14.20248137. Preprint.

US Census Bureau. “2010 Census Regions and Divisions of the United States.” The United States Census Bureau, 20 Aug. 2018, www.census.gov/geographies/reference-maps/2010/geo/2010-census-regions-and-divisions-of-the-united-states.html.

Yan, X., Lee, S., and Li, N. (2009), “Missing data handling methods in medical device clinical trials,” Journal of Biopharmaceutical Statistics, 19(6), 1085-1098.

DocUUID : d222bbbe-1732-48af-b4cf-1e79e47eea3b

Zou G. A modified Poisson regression approach to prospective studies with binary data. Am J Epidemiol. 2004;159(7):702-6.

12. Appendices

12.1. Appendix 1: Baseline Comorbidity Mappings and Medical Subgroupings to MedDRA terms

12.1.1. Preferred Terms

Medical Hx Category	Medical Hx Subcategory	Comorbidity	MedDRA Preferred Term (PT)	MedDRA PT Code
Blood Disorders	HIV/AIDS		AIDS related complex	10001513
Blood Disorders	HIV/AIDS		Acute HIV infection	10000807
Blood Disorders	HIV/AIDS		HIV-associated neurocognitive disorder	10078164
Blood Disorders	HIV/AIDS		Asymptomatic HIV infection	10003581
Blood Disorders	HIV/AIDS		HIV infection CDC Group I	10020162
Blood Disorders	HIV/AIDS		HIV infection CDC Group II	10020163
Blood Disorders	HIV/AIDS		HIV infection CDC Group III	10020164
Blood Disorders	HIV/AIDS		HIV infection CDC group IV	10061519
Blood Disorders	HIV/AIDS		HIV infection	10020161
Blood Disorders	HIV/AIDS		Enterocolitis AIDS	10014894
Blood Disorders	HIV/AIDS		HIV infection CDC Group IV subgroup A	10020166
Blood Disorders	HIV/AIDS		HIV infection CDC Group IV subgroup B	10020167
Blood Disorders	HIV/AIDS		HIV infection CDC Group IV subgroup C1	10020168
Blood Disorders	HIV/AIDS		HIV infection CDC Group IV subgroup C2	10020169
Blood Disorders	HIV/AIDS		HIV infection CDC Group IV subgroup D	10020170
Blood Disorders	HIV/AIDS		HIV infection CDC Group IV subgroup E	10020171
Blood Disorders	HIV/AIDS		HIV-2 infection	10020194
Blood Disorders	HIV/AIDS		HIV infection CDC category A	10075708
Blood Disorders	HIV/AIDS		HIV infection CDC category B	10075709
Blood Disorders	HIV/AIDS		HIV infection CDC category C	10075710
Blood Disorders	HIV/AIDS		AIDS related complication	10061624
Blood Disorders	HIV/AIDS		End stage AIDS	10049076
Blood Disorders	HIV/AIDS		HIV wasting syndrome	10050309
Blood Disorders	HIV/AIDS		AIDS retinopathy	10053227
Blood Disorders	HIV/AIDS		HIV infection WHO clinical stage I	10064446
Blood Disorders	HIV/AIDS		HIV infection WHO clinical stage II	10064447
Blood Disorders	HIV/AIDS		HIV infection WHO clinical stage III	10064448
Blood Disorders	HIV/AIDS		HIV infection WHO clinical stage IV	10064456
Blood Disorders	HIV/AIDS		HIV peripheral neuropathy	10065681
Blood Disorders	HIV/AIDS		AIDS cholangiopathy	10067092

DocUUID : d222bbbe-1732-48af-b4cf-1e79e47eea3b

Medical Hx Category	Medical Hx Subcategory	Comorbidity	MedDRA Preferred Term (PT)	MedDRA PT Code
Blood Disorders	HIV/AIDS		HIV cardiomyopathy	10069658
Blood Disorders	HIV/AIDS		HIV enteropathy	10069691
Blood Disorders	HIV/AIDS		HIV associated nephropathy	10070737
Blood Disorders	HIV/AIDS		Perinatal HIV infection	10071049
Blood Disorders	HIV/AIDS		HIV viraemia	10077716
Blood Disorders	HIV/AIDS		HIV lipodystrophy	10078720
Blood Disorders	HIV/AIDS		CSF HIV escape syndrome	10081027
Blood Disorders	HIV/AIDS		Papular pruritic eruption of HIV	10082425
Blood Disorders	HIV/AIDS		HIV meningoencephalitis	10083411
Blood Disorders	Sickle cell disease		Sickle cell anaemia	10040641
Blood Disorders	Sickle cell disease		Sickle cell anaemia with crisis	10040642
Blood Disorders	Sickle cell disease		Sickle cell disease	10040644
Blood Disorders	Sickle cell disease		Sickle cell trait	10040650
Blood Disorders	Sickle cell disease		Retinopathy sickle cell	10038935
Blood Disorders	Sickle cell disease		Thalassaemia sickle cell	10043395
Blood Disorders	Sickle cell disease	Chronic Kidney Disease	Sickle cell nephropathy	10084204
Cardiac Disorders	Atrial fibrillation		Atrial fibrillation	10003658
Cardiac Disorders	Congestive heart failure		Cardiac failure congestive	10007559
Cardiac Disorders	Coronary artery disease	Cardiovascular disease	Coronary artery disease	10011078
Cardiac Disorders	Hypertension		Hypertension	10020772
Cardiac Disorders	Valvular heart disease		Aortic valve disease mixed	10002912
Cardiac Disorders	Valvular heart disease		Aortic valve disease	10061589
Cardiac Disorders	Valvular heart disease		Mitral valve disease	10061532
Cardiac Disorders	Valvular heart disease		Tricuspid valve disease	10061389
Cardiac Disorders	Valvular heart disease		Cardiac valve disease	10061406
Cardiac Disorders	Valvular heart disease		Mitral valve disease mixed	10027724
Cardiac Disorders	Valvular heart disease		Pulmonary valve disease	10061541
Cardiac Disorders	Valvular heart disease		Degenerative aortic valve disease	10075846
Cardiac Disorders	Valvular heart disease		Degenerative mitral valve disease	10075847
Cardiac Disorders	Valvular heart disease		Degenerative tricuspid valve disease	10078909
Endocrine and Metabolic Disorders	Diabetes Mellitus		Diabetes mellitus inadequate control	10012607

Medical Hx Category	Medical Hx Subcategory	Comorbidity	MedDRA Preferred Term (PT)	MedDRA PT Code
Endocrine and Metabolic Disorders	Diabetes Mellitus	Diabetes Mellitus Type 2	Diabetes mellitus	10012601
Endocrine and Metabolic Disorders	Diabetes Mellitus		Diabetes complicating pregnancy	10012596
Endocrine and Metabolic Disorders	Diabetes Mellitus		Type 1 diabetes mellitus	10067584
Endocrine and Metabolic Disorders	Diabetes Mellitus	Diabetes Mellitus Type 2	Type 2 diabetes mellitus	10067585
Endocrine and Metabolic Disorders	Diabetes Mellitus		Steroid diabetes	10081755
Endocrine and Metabolic Disorders	Diabetes Mellitus		Diabetes with hyperosmolarity	10012631
Endocrine and Metabolic Disorders	Diabetes Mellitus		Increased insulin requirement	10021664
Endocrine and Metabolic Disorders	Diabetes Mellitus		Insulin resistant diabetes	10022491
Endocrine and Metabolic Disorders	Diabetes Mellitus		Ketosis-prone diabetes mellitus	10023392
Endocrine and Metabolic Disorders	Diabetes Mellitus		Monogenic diabetes	10075980
Endocrine and Metabolic Disorders	Diabetes Mellitus		Neonatal diabetes mellitus	10028933
Endocrine and Metabolic Disorders	Diabetes Mellitus		Pancreatogenous diabetes	10033660
Endocrine and Metabolic Disorders	Diabetes Mellitus	Diabetes Mellitus Type 2	Insulin-requiring type 2 diabetes mellitus	10053247
Endocrine and Metabolic Disorders	Diabetes Mellitus		Diabetes mellitus malnutrition-related	10050197
Endocrine and Metabolic Disorders	Diabetes Mellitus		Decreased insulin requirement	10052340
Endocrine and Metabolic Disorders	Diabetes Mellitus		Type 3 diabetes mellitus	10072659
Endocrine and Metabolic Disorders	Diabetes Mellitus		Acquired lipoatrophic diabetes	10073667
Endocrine and Metabolic Disorders	Diabetes Mellitus		Latent autoimmune diabetes in adults	10066389
Endocrine and Metabolic Disorders	Diabetes Mellitus		Fulminant type 1 diabetes mellitus	10072628
Endocrine and Metabolic Disorders	Diabetes Mellitus		New onset diabetes after transplantation	10082630
Endocrine and Metabolic Disorders	Hyperlipidemia		Hypercholesterolaemia	10020603
Endocrine and Metabolic Disorders	Hyperlipidemia		Hyperchylomicronaemia	10020606
Endocrine and Metabolic Disorders	Hyperlipidemia		Hyperlipidaemia	10062060
Endocrine and Metabolic Disorders	Hyperlipidemia		Hypertriglyceridaemia	10020869
Endocrine and Metabolic Disorders	Hyperlipidemia		Remnant hyperlipidaemia	10038316
Endocrine and Metabolic Disorders	Hyperlipidemia		Cholesterosis	10051914

Medical Hx Category	Medical Hx Subcategory	Comorbidity	MedDRA Preferred Term (PT)	MedDRA PT Code
Endocrine and Metabolic Disorders	Hyperlipidemia		Acquired mixed hyperlipidaemia	10071236
Endocrine and Metabolic Disorders	Hyperlipidemia		Autoimmune hyperlipidaemia	10071577
Liver Disorders	Chronic hepatitis		Hepatitis alcoholic	10019728
Liver Disorders	Chronic hepatitis		Autoimmune hepatitis	10003827
Liver Disorders	Chronic hepatitis		Hepatitis chronic active	10019755
Liver Disorders	Chronic hepatitis		Chronic hepatitis	10008909
Liver Disorders	Chronic hepatitis		Hepatitis chronic persistent	10019759
Liver Disorders	Chronic hepatitis		Hepatitis	10019717
Liver Disorders	Chronic hepatitis		Hepatitis toxic	10019795
Liver Disorders	Chronic hepatitis		Lupus hepatitis	10067737
Liver Disorders	Chronic hepatitis		Alloimmune hepatitis	10080576
Liver Disorders	Chronic liver disease		Liver disorder	10024670
Liver Disorders	Cirrhosis		Cirrhosis alcoholic	10009208
Liver Disorders	Cirrhosis		Biliary cirrhosis	10004659
Liver Disorders	Cirrhosis		Hepatic cirrhosis	10019641
Liver Disorders	Cirrhosis		Lupoid hepatic cirrhosis	10025129
Liver Disorders	Cirrhosis		Cardiac cirrhosis	10054936
Liver Disorders	Cirrhosis		Cryptogenic cirrhosis	10063075
Liver Disorders	Cirrhosis		Pseudocirrhosis	10076501
Neurologic Disorders	Parkinson disease		Cogwheel rigidity	10009848
Neurologic Disorders	Parkinson disease		Freezing phenomenon	10060904
Neurologic Disorders	Parkinson disease		Hypokinetic dysarthria	10082243
Neurologic Disorders	Parkinson disease		On and off phenomenon	10030312
Neurologic Disorders	Parkinson disease		Parkinsonian crisis	10048868
Neurologic Disorders	Parkinson disease		Parkinsonian gait	10056242
Neurologic Disorders	Parkinson disease		Parkinsonian rest tremor	10056437
Neurologic Disorders	Parkinson disease		Parkinsonism	10034010
Neurologic Disorders	Parkinson disease		Parkinsonism hyperpyrexia syndrome	10071243
Neurologic Disorders	Parkinson disease		Parkinson's disease	10061536
Neurologic Disorders	Parkinson disease		Reduced facial expression	10078576
Neurologic Disorders	Parkinson disease		Vascular parkinsonism	10068100
Neurologic Disorders	Stroke		Basal ganglia stroke	10071043
Neurologic Disorders	Stroke		Brain stem stroke	10068644
Neurologic Disorders	Stroke		Cerebellar stroke	10079062
Neurologic Disorders	Stroke		Embolic stroke	10014498
Neurologic Disorders	Stroke		Haemorrhagic stroke	10019016
Neurologic Disorders	Stroke		Haemorrhagic transformation stroke	10055677

Medical Hx Category	Medical Hx Subcategory	Comorbidity	MedDRA Preferred Term (PT)	MedDRA PT Code
Neurologic Disorders	Stroke		Ischaemic stroke	10061256
Neurologic Disorders	Stroke		Lacunar stroke	10076994
Neurologic Disorders	Stroke		Perinatal stroke	10073945
Neurologic Disorders	Stroke		Pseudostroke	10078090
Neurologic Disorders	Stroke		Spinal stroke	10082031
Neurologic Disorders	Stroke		Stroke in evolution	10059613
Neurologic Disorders	Stroke		Thrombotic stroke	10043647
Neurologic Disorders	Stroke		Vertebrobasilar stroke	10082484
Respiratory Disorders	Asthma	Chronic lung disease	Asthma	10003553
Respiratory Disorders	Asthma	Chronic lung disease	Asthma exercise induced	10003557
Respiratory Disorders	Asthma	Chronic lung disease	Asthma late onset	10003559
Respiratory Disorders	Asthma	Chronic lung disease	Status asthmaticus	10041961
Respiratory Disorders	Asthma	Chronic lung disease	Childhood asthma	10081274
Respiratory Disorders	Asthma	Chronic lung disease	Cough variant asthma	10063076
Respiratory Disorders	Asthma	Chronic lung disease	Asthmatic crisis	10064823
Respiratory Disorders	Asthma	Chronic lung disease	Occupational asthma	10070836
Respiratory Disorders	Chronic obstructive lung disease	Chronic lung disease	Chronic obstructive pulmonary disease	10009033
Respiratory Disorders	Chronic obstructive lung disease	Chronic lung disease	Asthma-chronic obstructive pulmonary disease overlap syndrome	10077005
Urologic Disorders	Chronic Kidney Disease	Chronic Kidney Disease	Renal failure	10038435
Urologic Disorders	Chronic Kidney Disease	Chronic Kidney Disease	Chronic kidney disease	10064848
Urologic Disorders	Chronic Kidney Disease	Chronic Kidney Disease	End stage renal disease	10077512
Urologic Disorders	Chronic Kidney Disease	Chronic Kidney Disease	Renal impairment	10062237
Urologic Disorders	Chronic Kidney Disease	Chronic Kidney Disease	Diabetic end stage renal disease	10012660
Urologic Disorders	Chronic Kidney Disease	Chronic Kidney Disease	Prerenal failure	10072370
Urologic Disorders	Chronic Kidney Disease	Chronic Kidney Disease	Pancreatorenal syndrome	10056277
Urologic Disorders	Chronic Kidney Disease	Chronic Kidney Disease	Postrenal failure	10059345

DocUUID : d222bbbe-1732-48af-b4cf-1e79e47eea3b

Medical Hx Category	Medical Hx Subcategory	Comorbidity	MedDRA Preferred Term (PT)	MedDRA PT Code
Urologic Disorders	Chronic Kidney Disease	Chronic Kidney Disease	Scleroderma renal crisis	10062553
Urologic Disorders	Kidney stones and obstruction		Hydronephrosis	10020524
Urologic Disorders	Kidney stones and obstruction		Stag horn calculus	10041900
Urologic Disorders	Kidney stones and obstruction		Nephrocalcinosis	10029146
Urologic Disorders	Kidney stones and obstruction		Nephrolithiasis	10029148
Urologic Disorders	Kidney stones and obstruction		Hydrocalyx	10073745
Urologic Disorders	Kidney stones and obstruction		Malignant urinary tract obstruction	10080015
Urologic Disorders	Kidney stones and obstruction		Pelvi-ureteric obstruction	10034232
Urologic Disorders	Kidney stones and obstruction		Retrograde migration of renal calculi	10083032

12.1.2. Higher Level Group Terms

Medical Hx Category	MedDRA Higher Level Group Term (HLGT)	MedDRA HLGT Code
Cancer	Leukaemias	10024324
Cancer	Skin neoplasms malignant and unspecified	10040900
Cancer	Endocrine neoplasms malignant and unspecified	10014713
Cancer	Haematopoietic neoplasms (excl leukaemias and lymphomas)	10018865
Cancer	Miscellaneous and site unspecified neoplasms malignant and unspecified	10027655
Cancer	Reproductive neoplasms female malignant and unspecified	10038594
Cancer	Gastrointestinal neoplasms malignant and unspecified	10017991
Cancer	Respiratory and mediastinal neoplasms malignant and unspecified	10038666
Cancer	Renal and urinary tract neoplasms malignant and unspecified	10038364
Cancer	Reproductive neoplasms male malignant and unspecified	10038597
Cancer	Metastases	10027476
Cancer	Lymphomas non-Hodgkin's T-cell	10025321
Cancer	Nervous system neoplasms malignant and unspecified NEC	10029211
Cancer	Lymphomas NEC	10025323
Cancer	Soft tissue neoplasms malignant and unspecified	10072990
Cancer	Skeletal neoplasms malignant and unspecified	10040778
Cancer	Lymphomas non-Hodgkin's B-cell	10025320
Cancer	Ocular neoplasms	10030054
Cancer	Mesotheliomas	10027412
Cancer	Plasma cell neoplasms	10035227

Medical Hx Category	MedDRA Higher Level Group Term (HLGT)	MedDRA HLGT Code
Cancer	Hepatobiliary neoplasms malignant and unspecified	10019815
Cancer	Breast neoplasms malignant and unspecified (incl nipple)	10006291
Cancer	Lymphomas non-Hodgkin's unspecified histology	10025322
Cancer	Reproductive and genitourinary neoplasms gender unspecified NEC	10038588
Cancer	Lymphomas Hodgkin's disease	10025319
Cancer	Neoplasm related morbidities	10068775
