# Supplementary material for: Safety, Immunogenicity, and Efficacy of the NVX-CoV2373 COVID-19 Vaccine in Adolescents
Source: JAMA Netw Open. Author manuscript; Available in PMC 2023 Sep 28. (PMC10536880; doi:10.1001/jamanetworkopen.2023.9135)
Supplement: Supplementary file 4 [file NIHMS1927823-supplement-Supplement_4_Data_Sharing_Statement.pdf]

Data Sharing Statement

Áñez. Safety, Immunogenicity, and Efficacy of the NVX-CoV2373 COVID-19 Vaccine in Adolescents. *JAMA Netw Open*. Published April 26, 2023.
doi:10.1001/jamanetworkopen.2023.9135

Data
Data available: No
